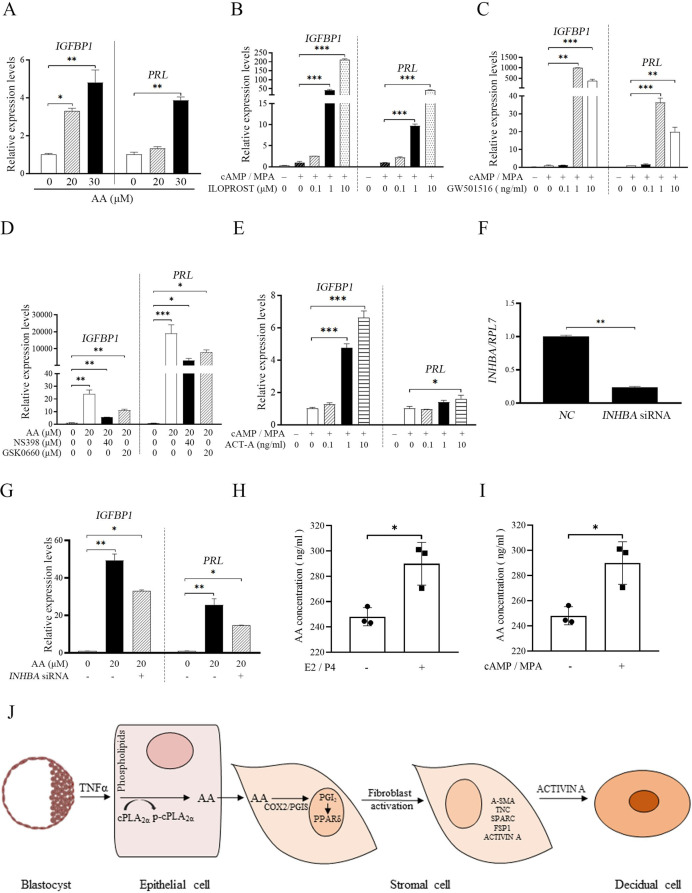# Correction: Embryo-derive TNF promotes decidualization via fibroblast activation

**DOI:** 10.7554/eLife.112145

**Published:** 2026-05-26

**Authors:** Si-Ting Chen, Wen-Wen Shi, Yu-Qian Lin, Zhen-Shan Yang, Ying Wang, Meng-Yuan Li, Yue Li, Ai-Xia Liu, Yali Hu, Zeng-Ming Yang

**Keywords:** Human, Mouse

Chen S-T, Shi W-W, Lin Y-Q, Yang Z-S, Wang Y, Li M-Y, Li Y, Liu A-X, Hu Y, Yang Z-M. 2023. Embryo-derive TNF promotes decidualization via fibroblast activation. *eLife* **12**:e82970. doi: 10.7554/eLife.82970.

Published 17 July 2023

After publication we noticed three errors in the published paper.

1. The TUBULIN bands for loading control in Figure 2G was erroneously duplicated in Figure 5H. The error occurred during panel assembly. The published Source data containing the original TUBULIN blot images for Figure 5H are however correct.

We have corrected the figure by replacing the duplicated TUBULIN bands in Figure 5H.

2. The IP bands in Figure 3B was erroneously duplicated in Figure 7E. This error occurred during panel assembly. Additionally, during revision Figure 7E was updated to include IP bands whilst the Source data not similarly updated to include the original IP blot images.

We have corrected the Figure 7E to show the correct IP bands and also updated the Source data for this Figure to include the previously inadvertently omitted original IP blot image.

3. The histogram in Figure 8I was erroneously duplicated in Figure 8H. This error also occurred during Figure assembly. Below we show the original data for these two histograms:

**Table:** 

Figure 8H					Figure 8I			
			AA concentration(ng/ml)					AA concentration(ng/ml)
mesc-1D	E2/ P4	-	260.61		4003-4D	cAMP/ MPA	-	256.24
			248.58					244.57
			268.19					243.26
		+	298.20				+	298.20
			291.72					270.54
			302.02					301.06

We sincerely apologize for this inadvertent mistake during figure assembly.

The corrected Figure 5 (with updated panel H, tubulin bands) is shown here:

**Figure 1:**
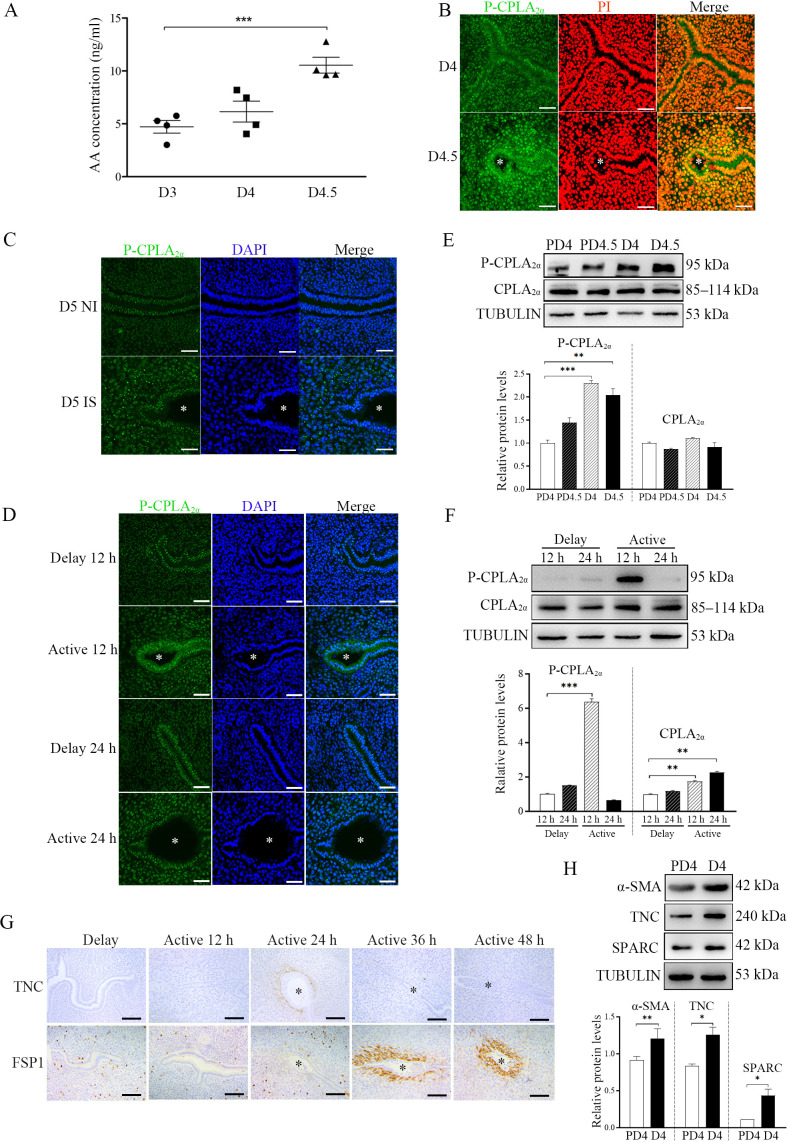


The originally published Figure 5 is shown here for reference:

**Figure 2:**
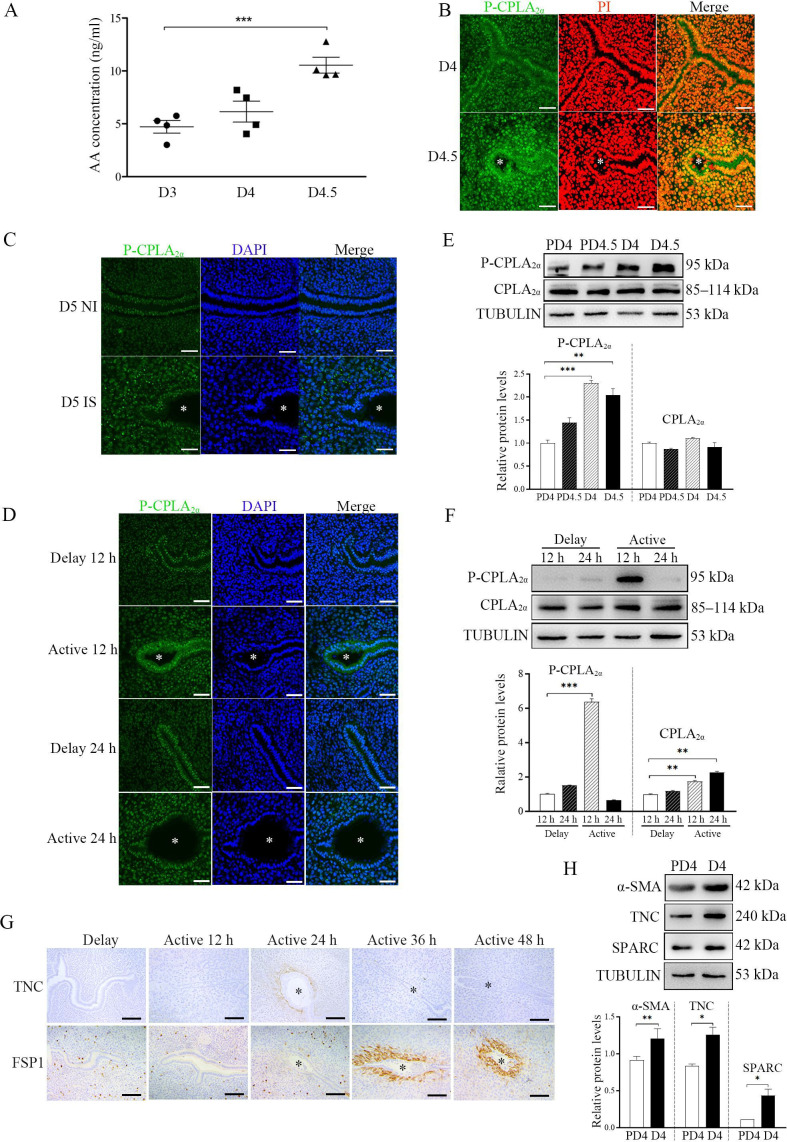


The corrected Figure 7 (with updated panel E, IP bands) is shown here:

**Figure 3:**
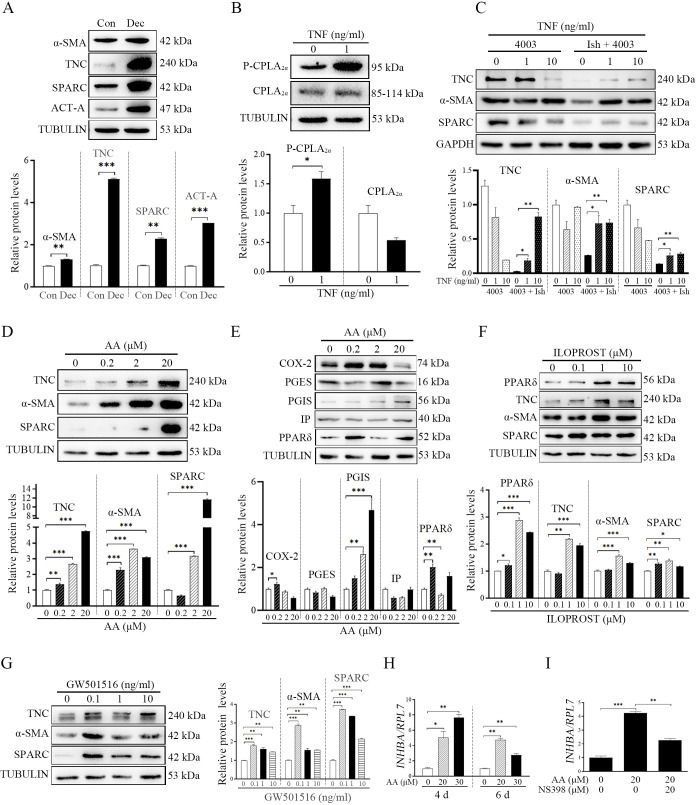


The originally published Figure 7 is shown here for reference:

**Figure 4:**
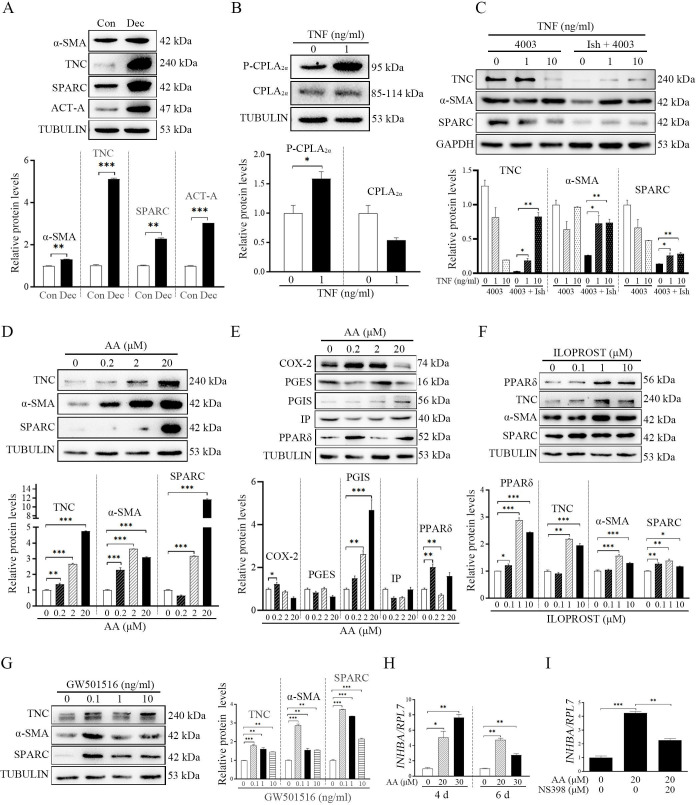


The corrected Figure 8 (with updated panel H) is shown here:

**Figure 5:**
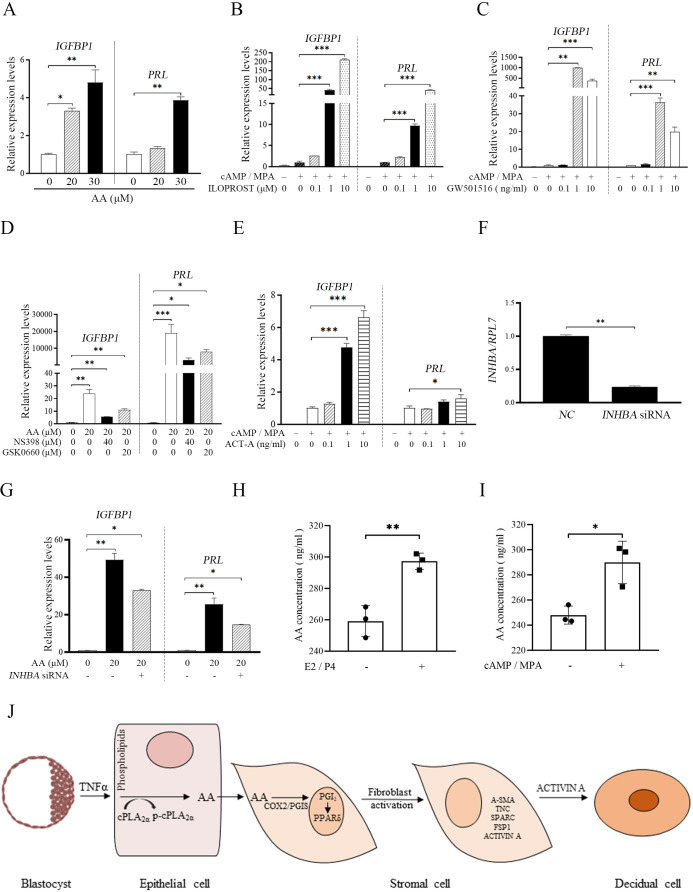


The originally published Figure 8 is shown here for reference:

**Figure 6:**