# Supplementary figures and images for: Embryo-derive TNF promotes decidualization via fibroblast activation (part 1 of 2)
Source: eLife. 2023 Jul 17;12:e82970. doi: 10.7554/eLife.82970 (PMC10374279; doi:10.7554/eLife.82970)

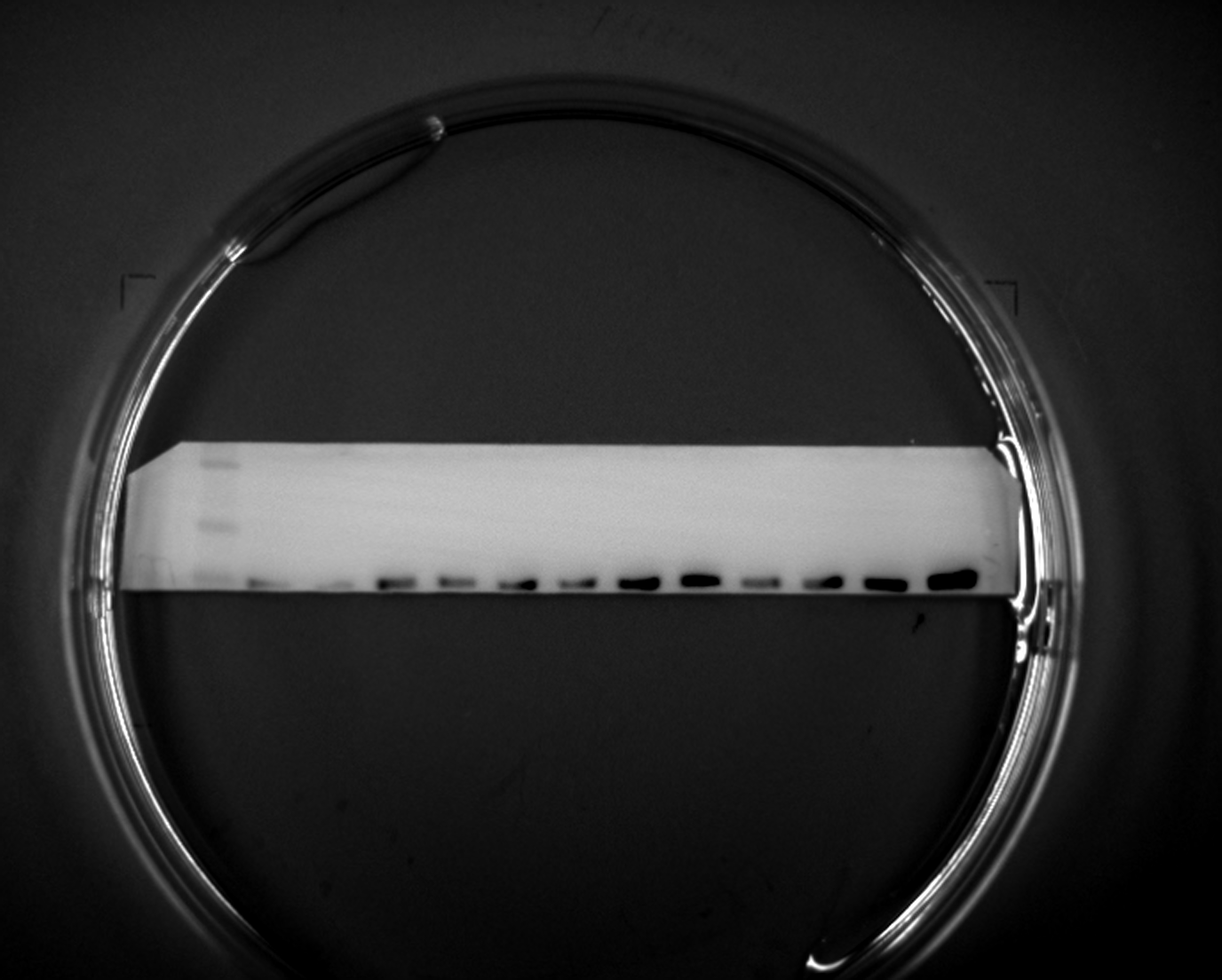

Supplement: Figure 1—source data 1. [file elife-82970-fig1-data1.zip › Figure_1-source_data_1/Figure_1-source_data_1_Figure_1B_SPARC.tif]

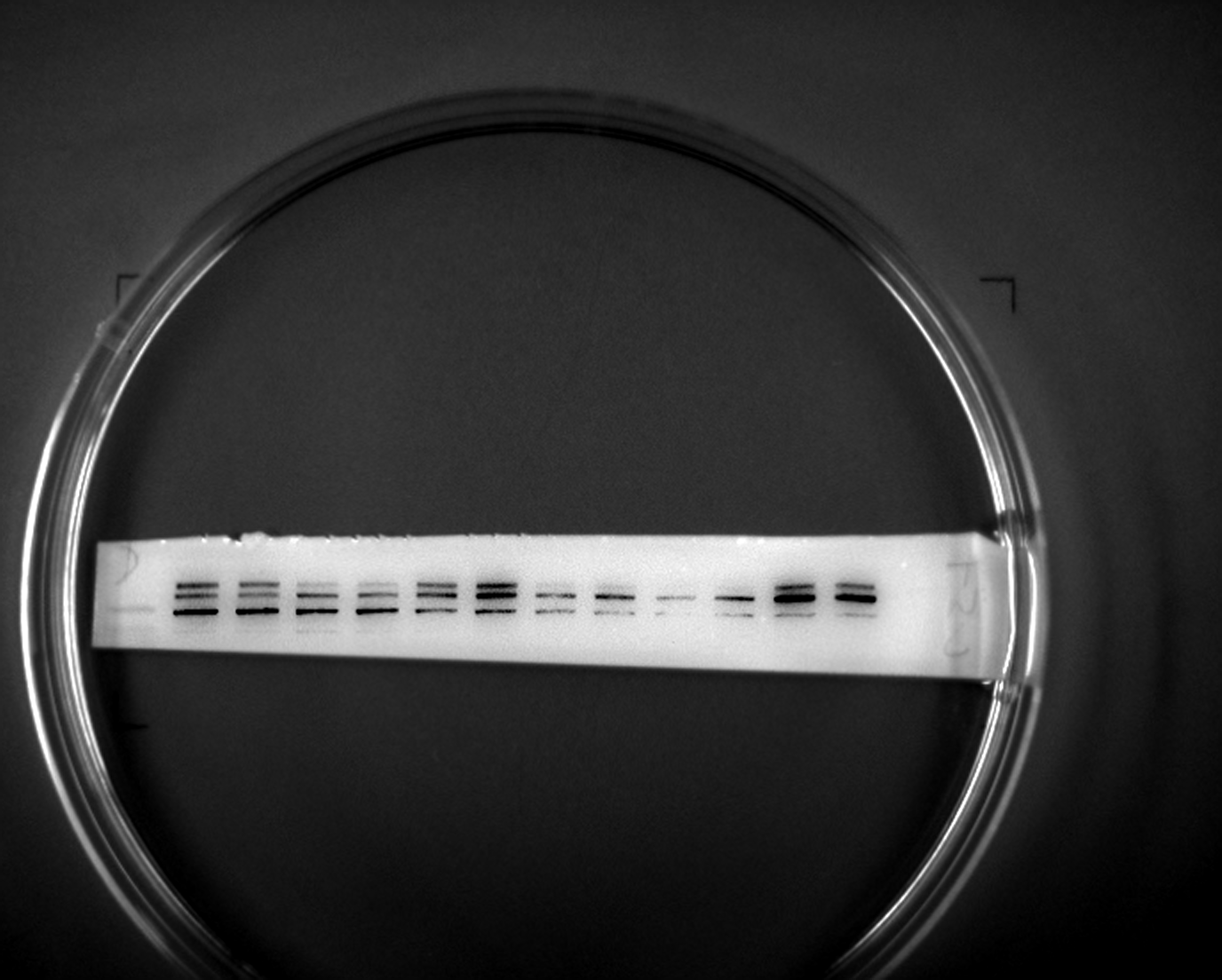

Supplement: Figure 1—source data 1. [file elife-82970-fig1-data1.zip › Figure_1-source_data_1/Figure_1-source_data_1_Figure_1B_TNC.tif]

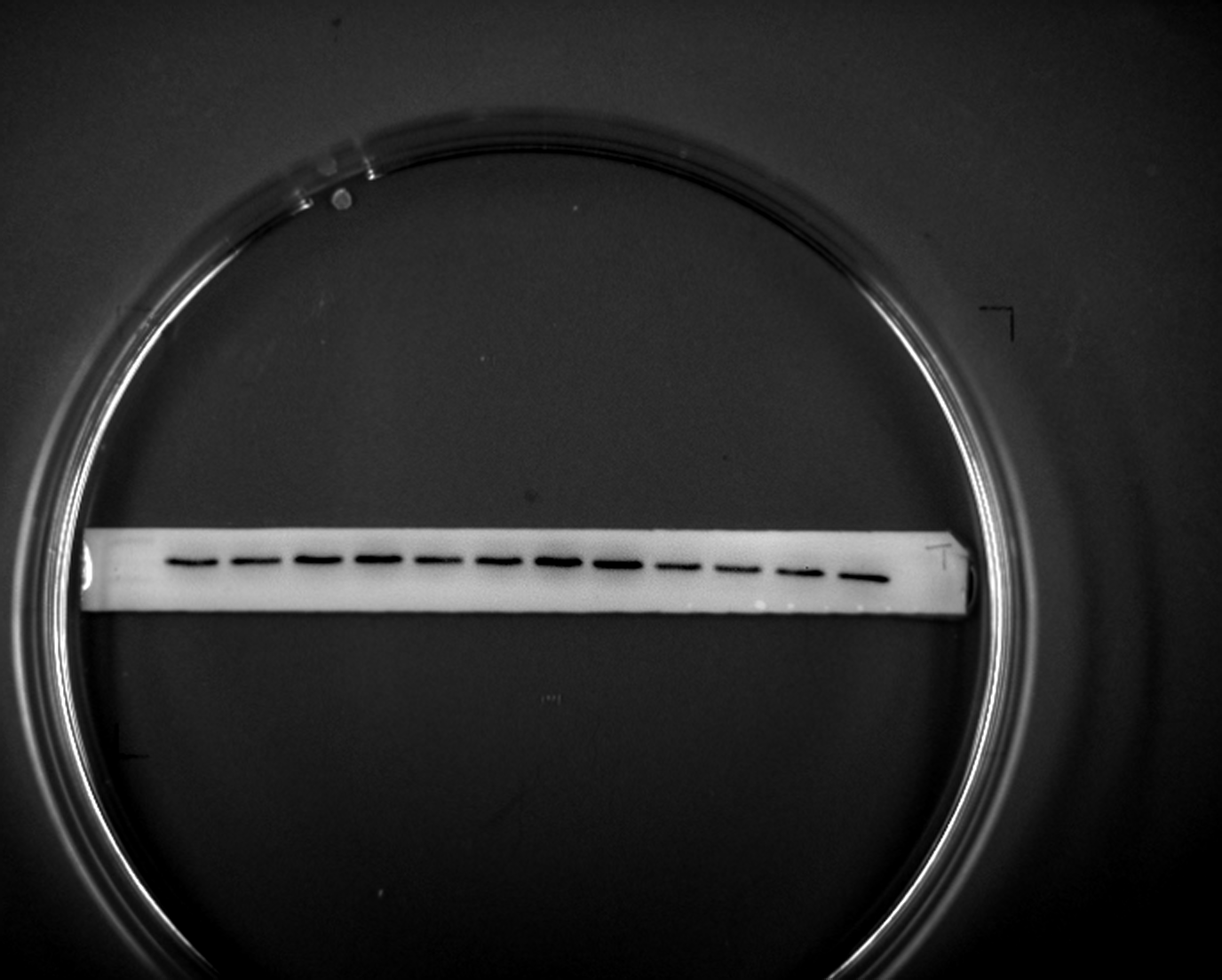

Supplement: Figure 1—source data 1. [file elife-82970-fig1-data1.zip › Figure_1-source_data_1/Figure_1-source_data_1_Figure_1B_TUBULIN.tif]

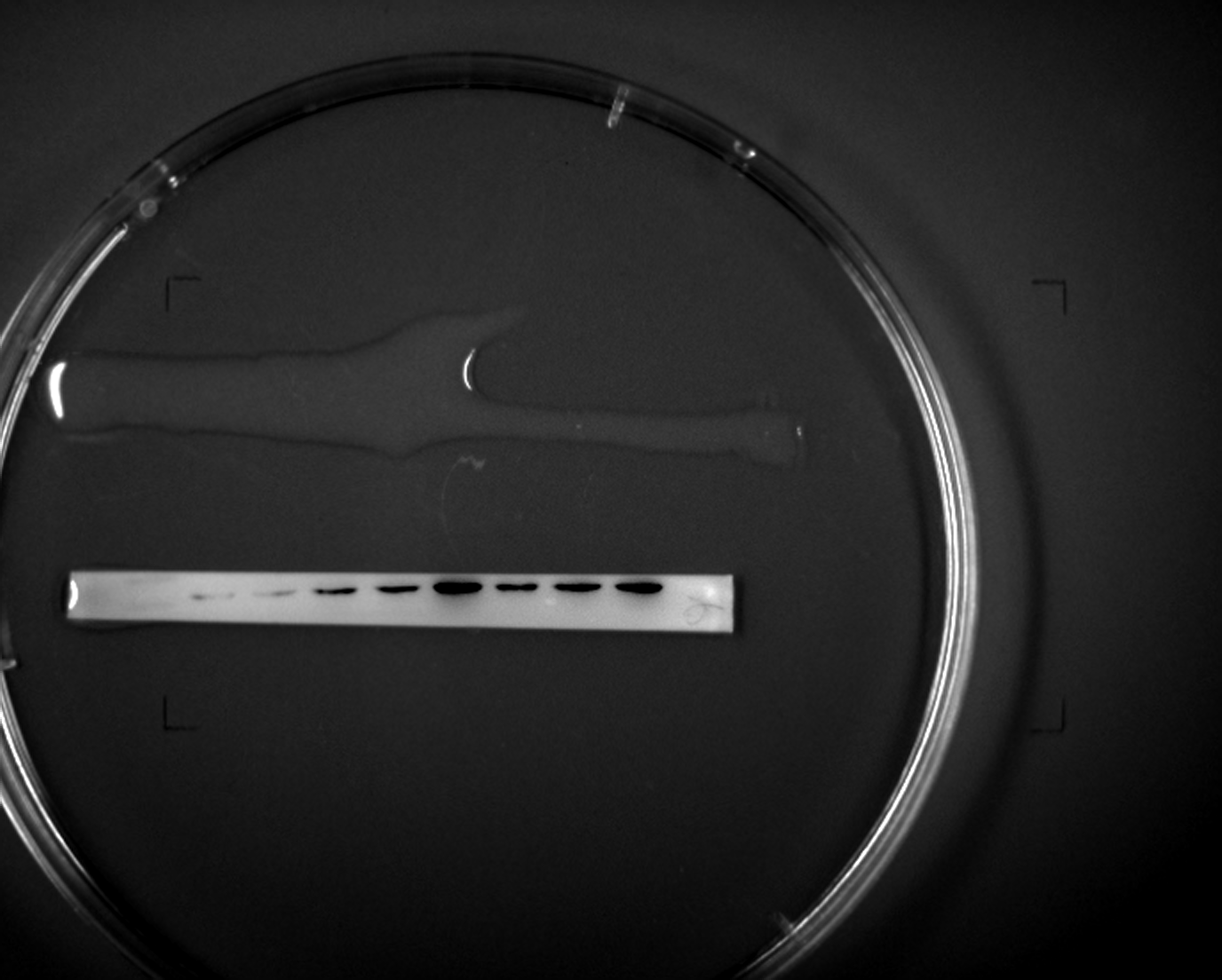

Supplement: Figure 1—source data 1. [file elife-82970-fig1-data1.zip › Figure_1-source_data_1/Figure_1-source_data_1_Figure_1B_a┴-SMA.tif]

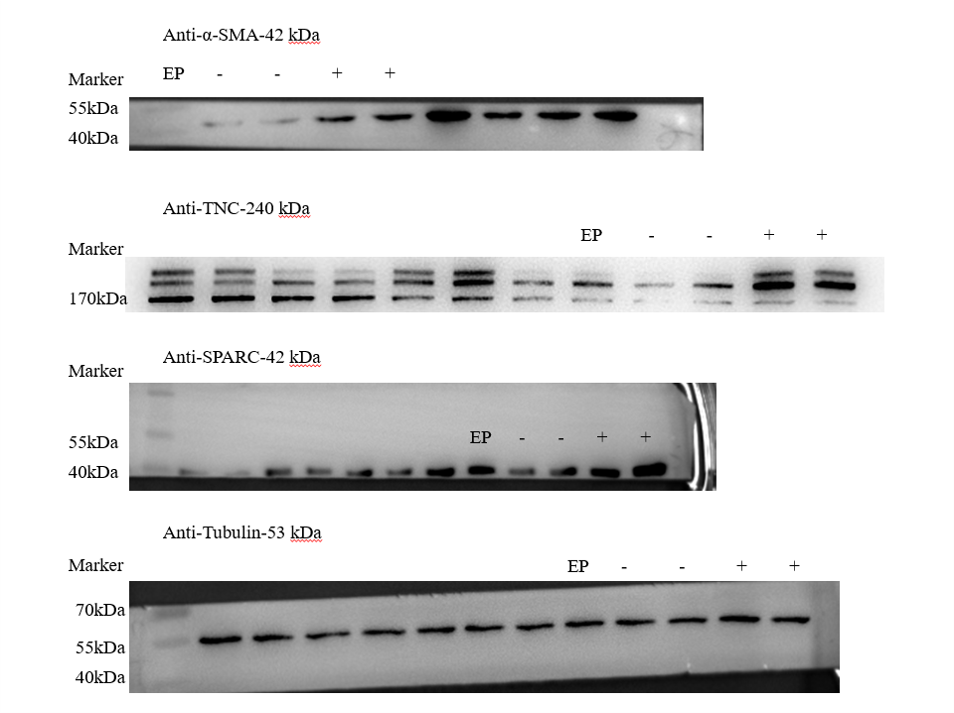

Supplement: Figure 1—source data 2. [file elife-82970-fig1-data2.zip › Figure_1-source_data_2/Figure_1-source_data_2-1B.png]

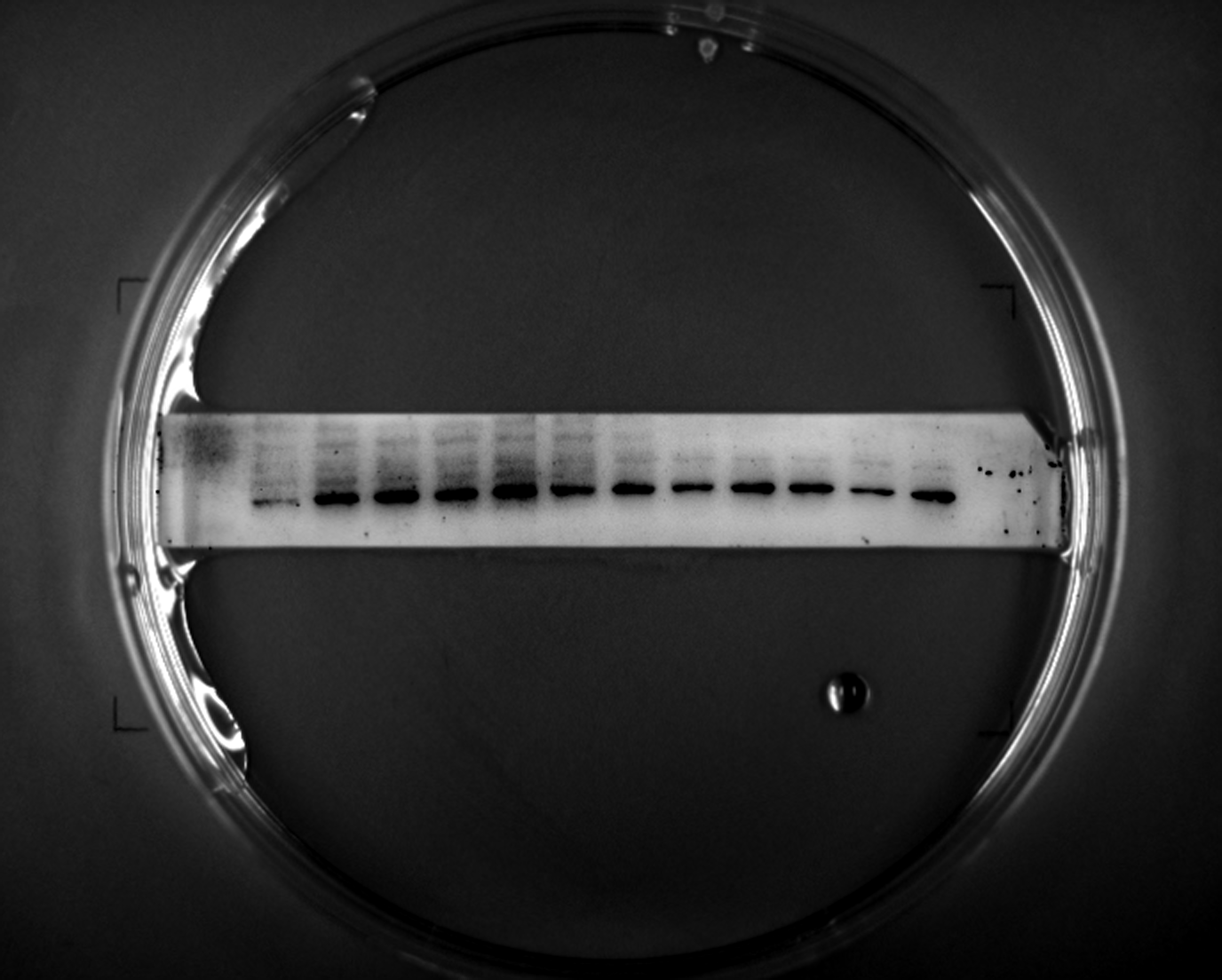

Supplement: Figure 2—source data 1. [file elife-82970-fig2-data1.zip › Figure_2-source_data_1/Figure_2-source_data_1_Figure_2A_BMP2.tif]

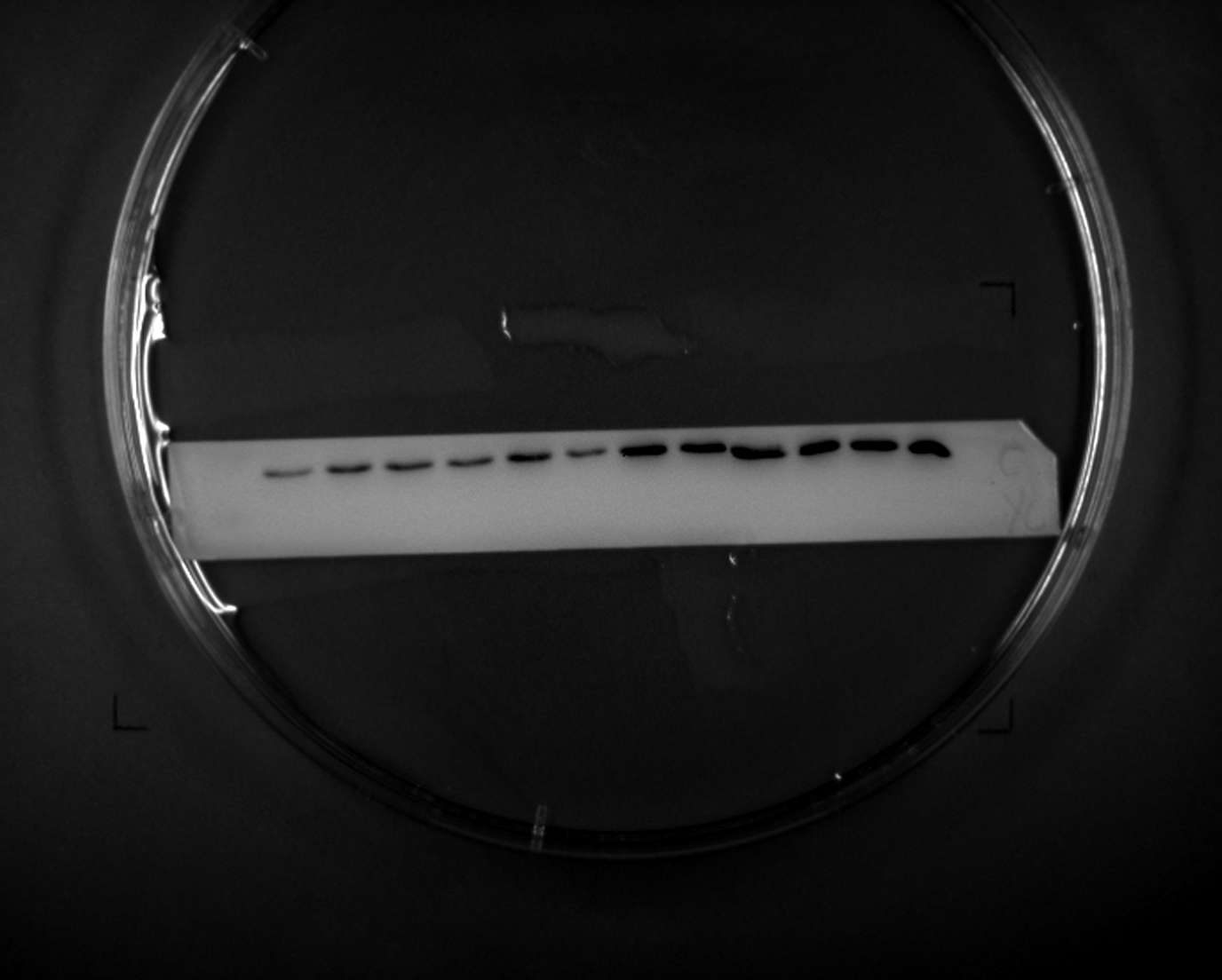

Supplement: Figure 2—source data 1. [file elife-82970-fig2-data1.zip › Figure_2-source_data_1/Figure_2-source_data_1_Figure_2A_CYCLIN D3.tif]

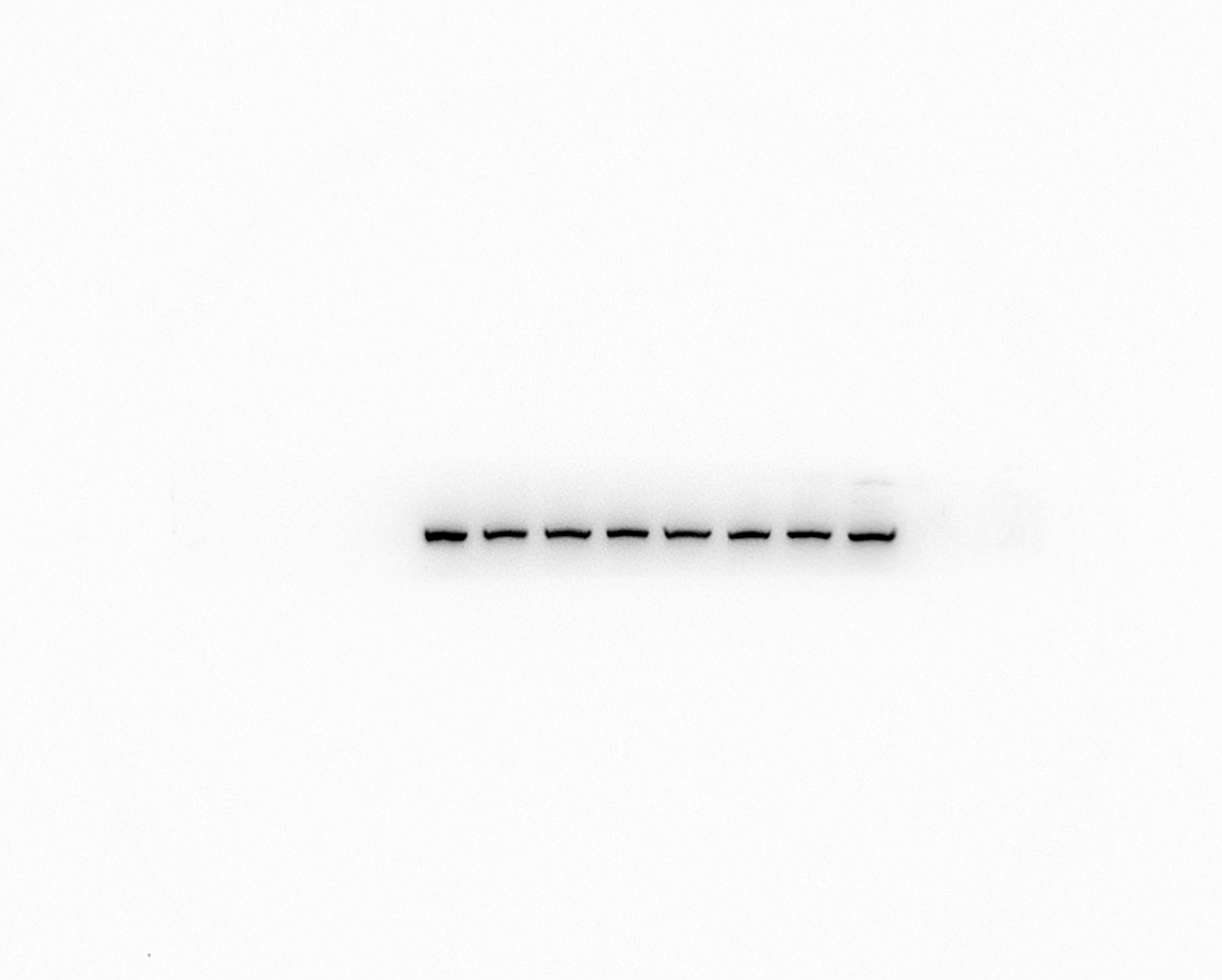

Supplement: Figure 2—source data 1. [file elife-82970-fig2-data1.zip › Figure_2-source_data_1/Figure_2-source_data_1_Figure_2A_E2F8.tif]

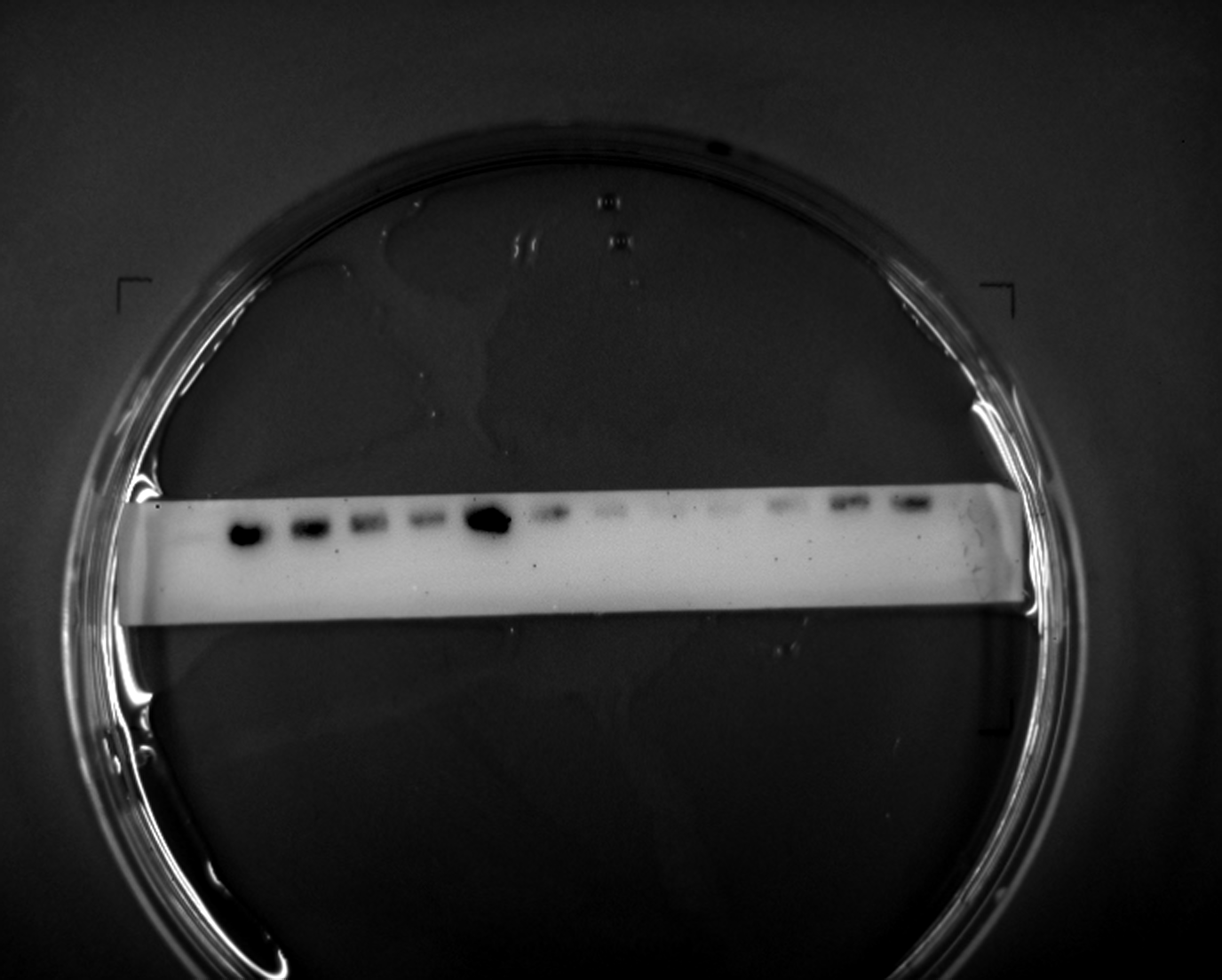

Supplement: Figure 2—source data 1. [file elife-82970-fig2-data1.zip › Figure_2-source_data_1/Figure_2-source_data_1_Figure_2A_WNT4.tif]

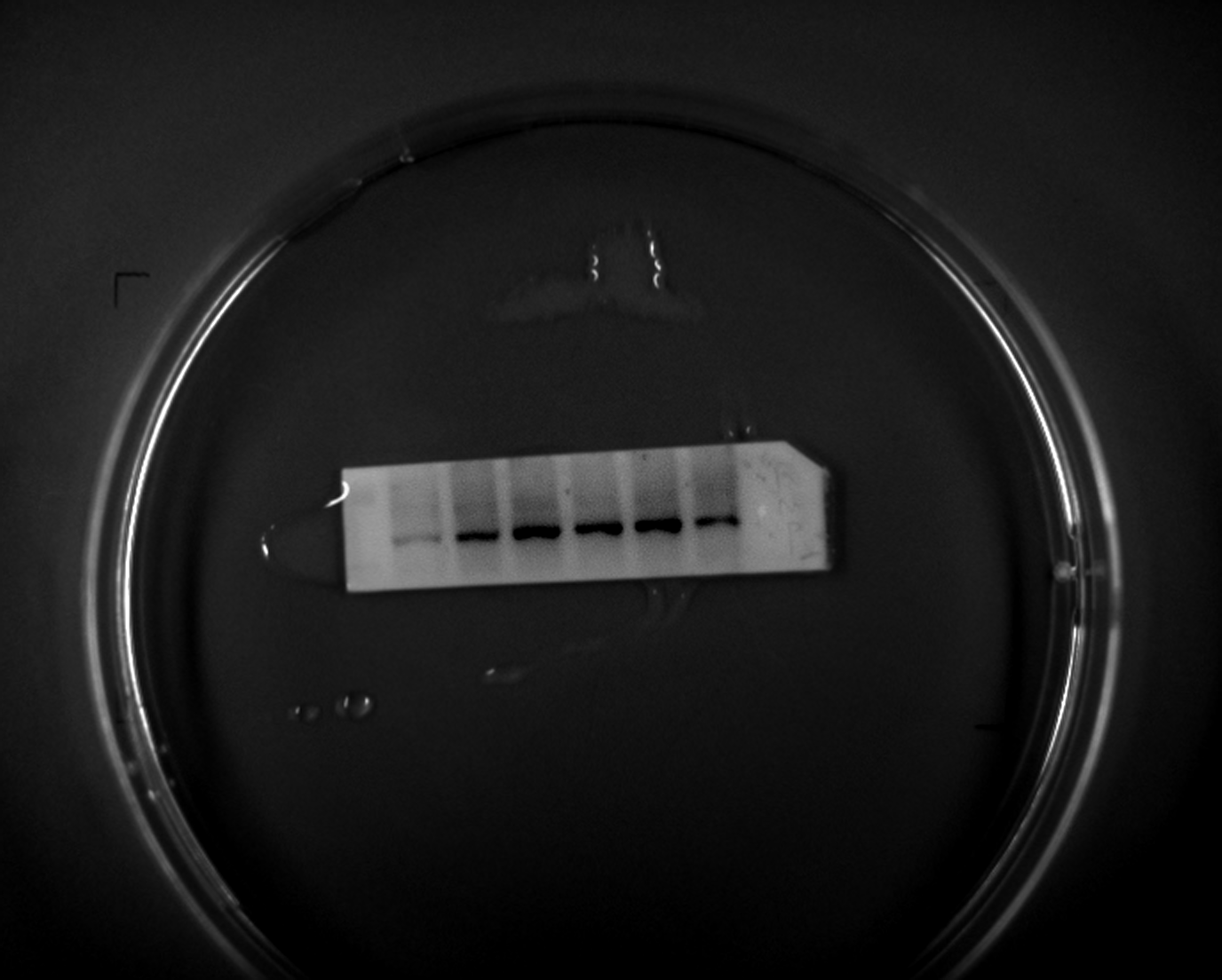

Supplement: Figure 2—source data 1. [file elife-82970-fig2-data1.zip › Figure_2-source_data_1/Figure_2-source_data_1_Figure_2B_BMP2.tif]

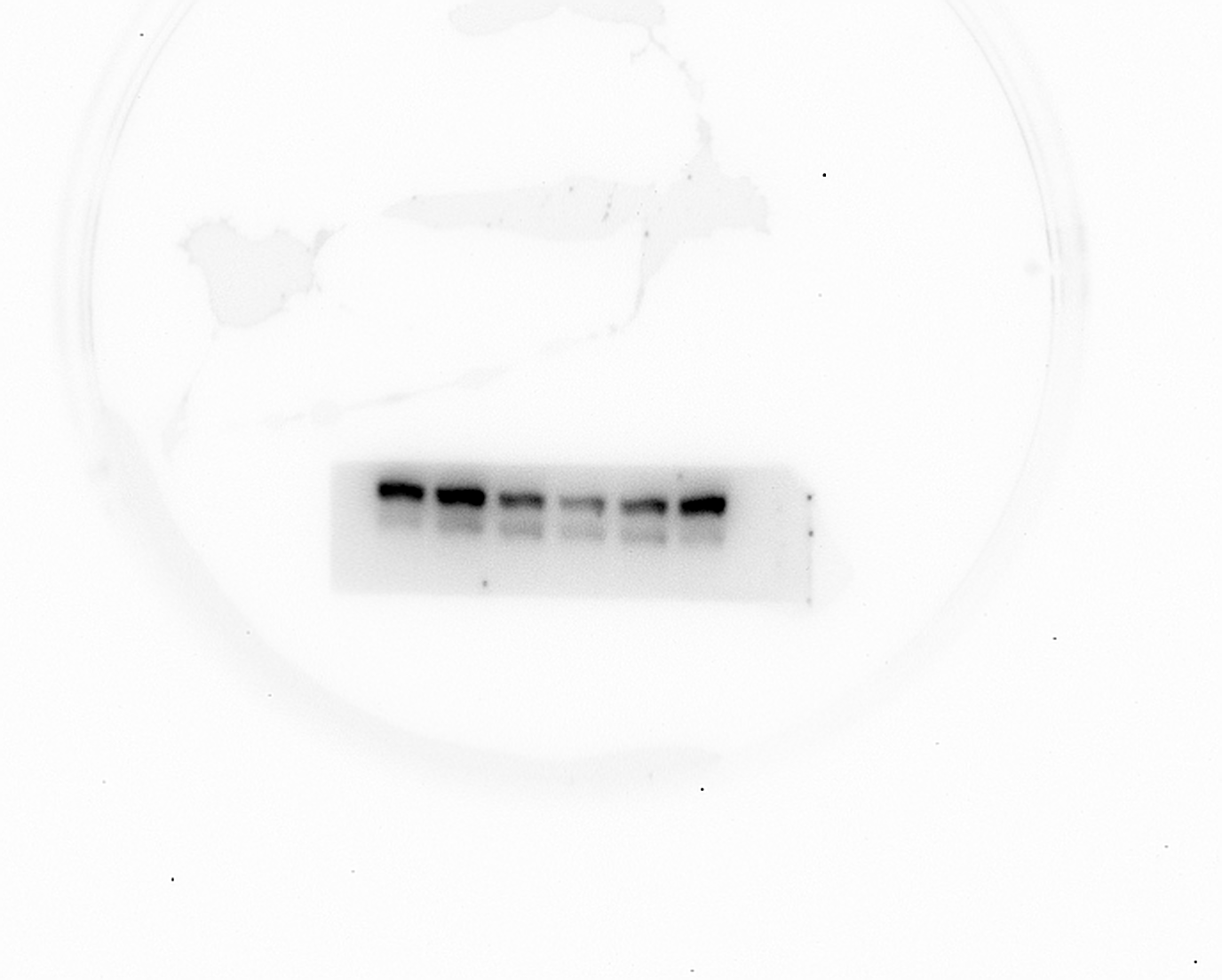

Supplement: Figure 2—source data 1. [file elife-82970-fig2-data1.zip › Figure_2-source_data_1/Figure_2-source_data_1_Figure_2B_CYCLIN D3.tif]

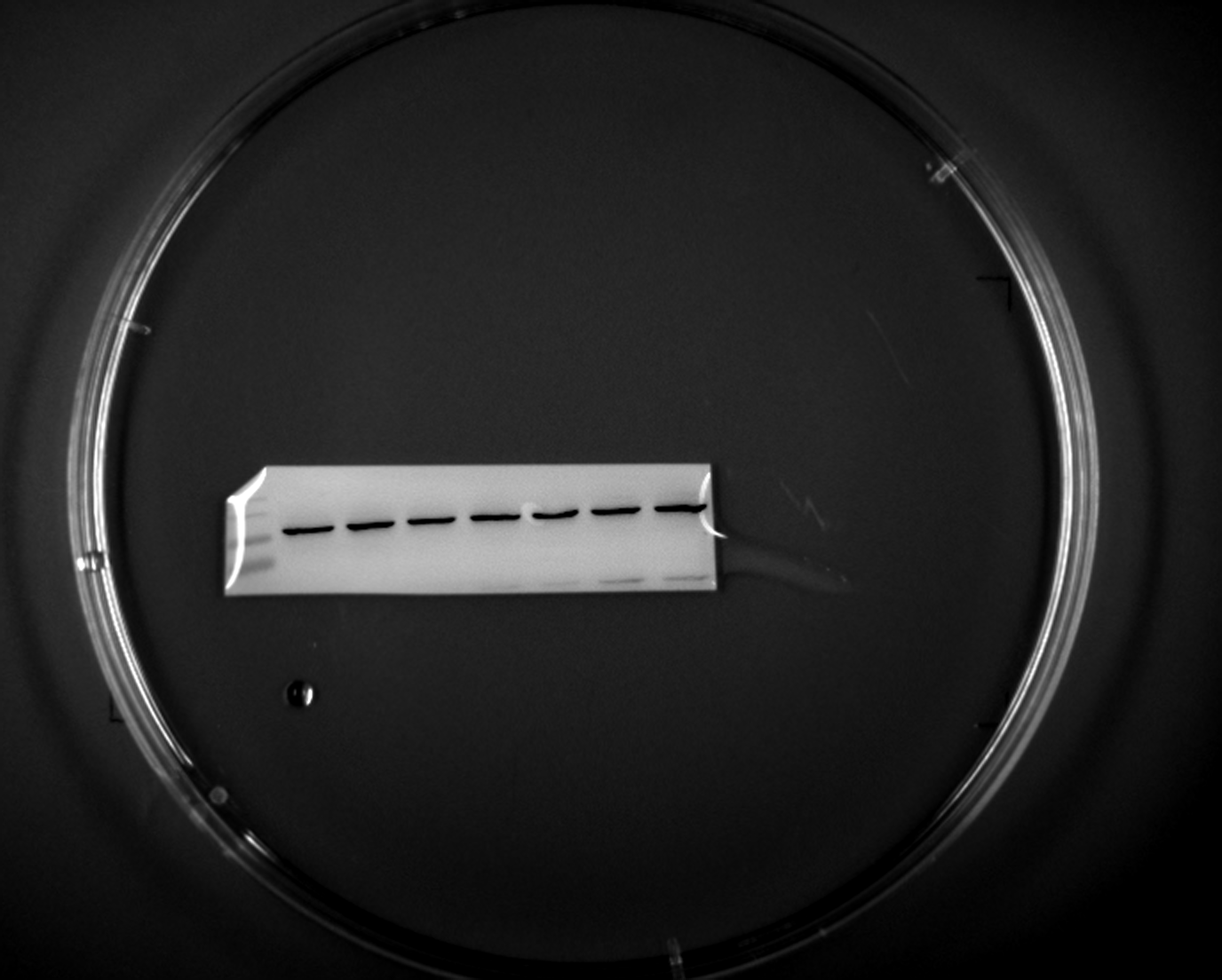

Supplement: Figure 2—source data 1. [file elife-82970-fig2-data1.zip › Figure_2-source_data_1/Figure_2-source_data_1_Figure_2B_E2F8.tif]

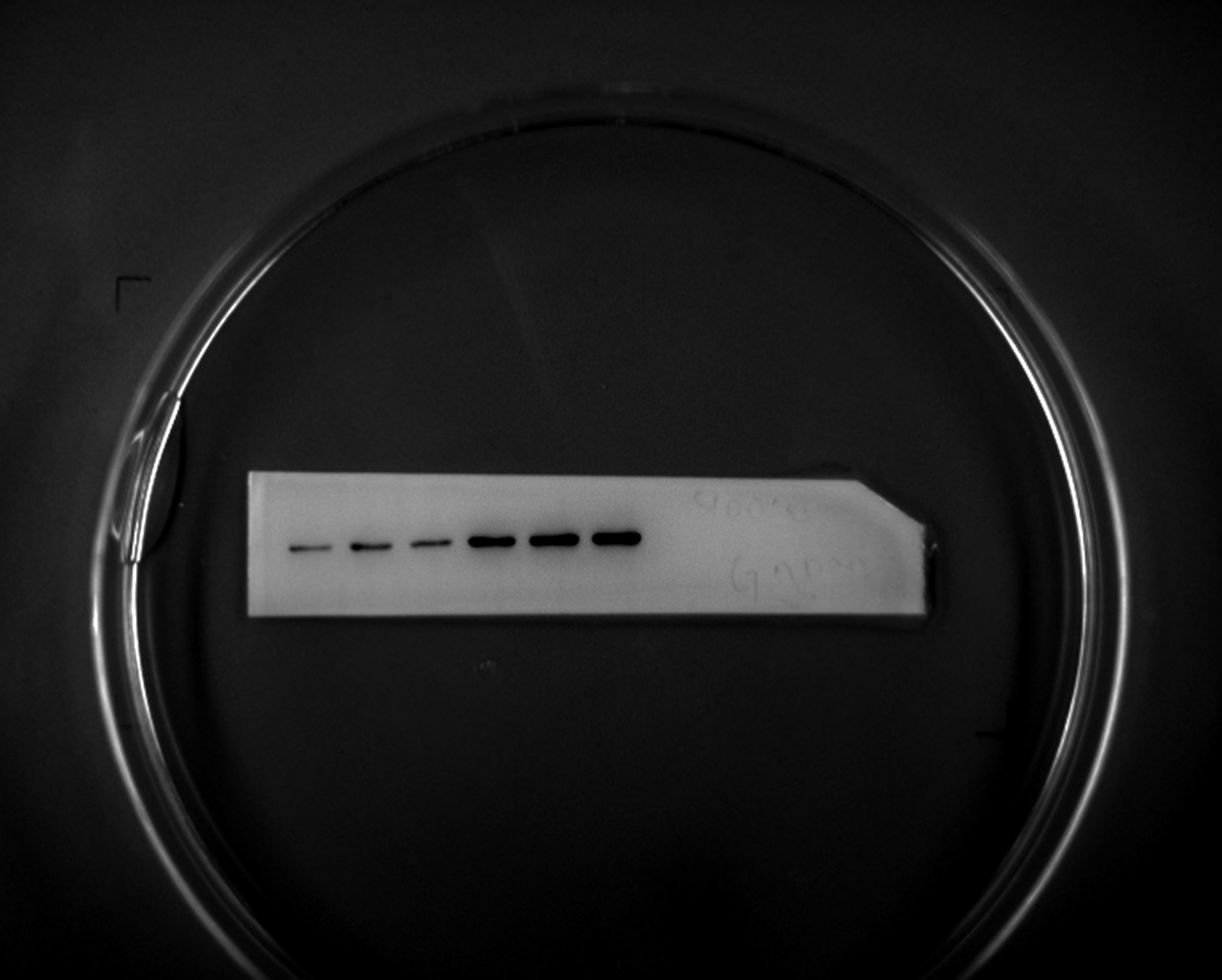

Supplement: Figure 2—source data 1. [file elife-82970-fig2-data1.zip › Figure_2-source_data_1/Figure_2-source_data_1_Figure_2B_TUBULIN.tif]

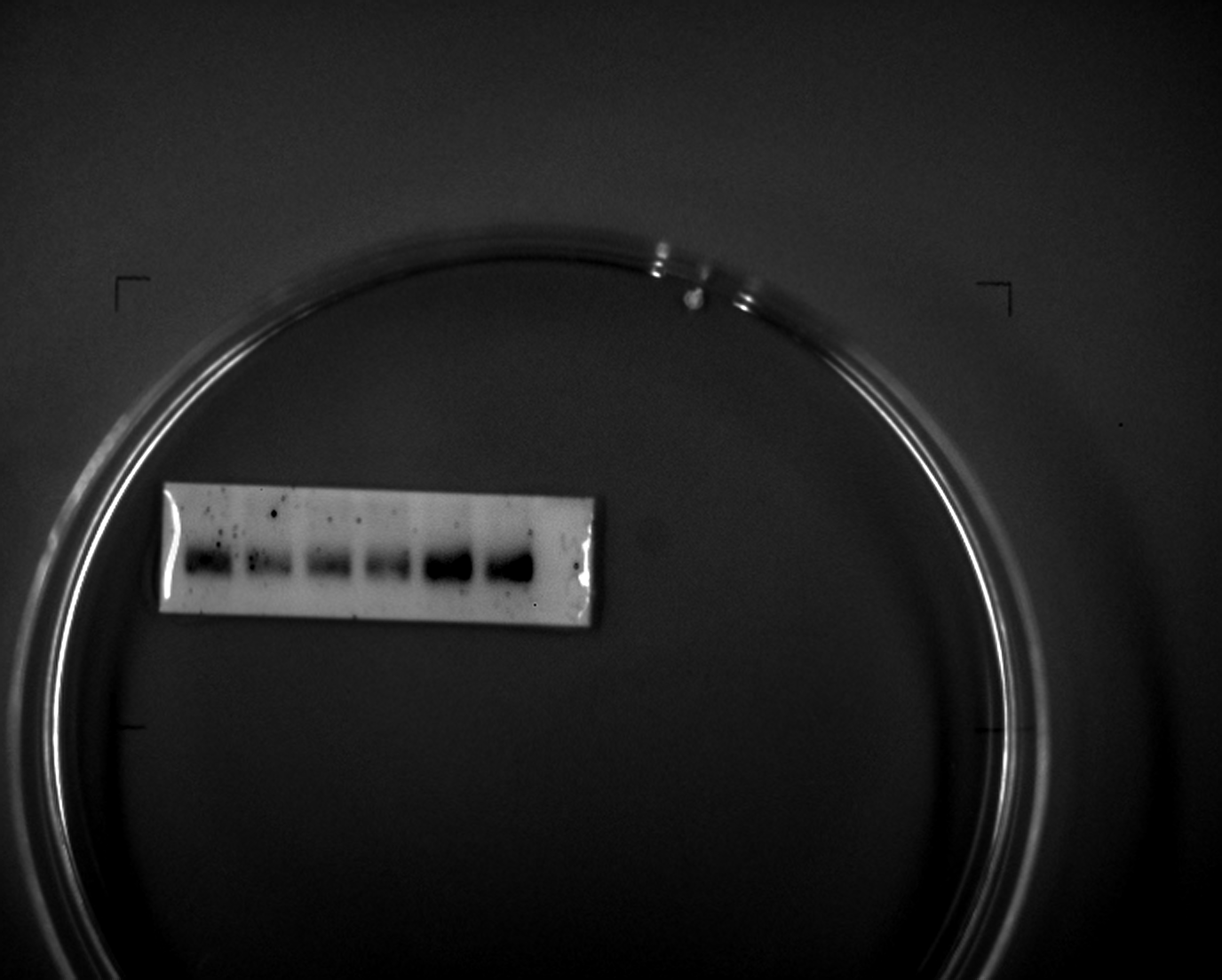

Supplement: Figure 2—source data 1. [file elife-82970-fig2-data1.zip › Figure_2-source_data_1/Figure_2-source_data_1_Figure_2B_WNT4.tif]

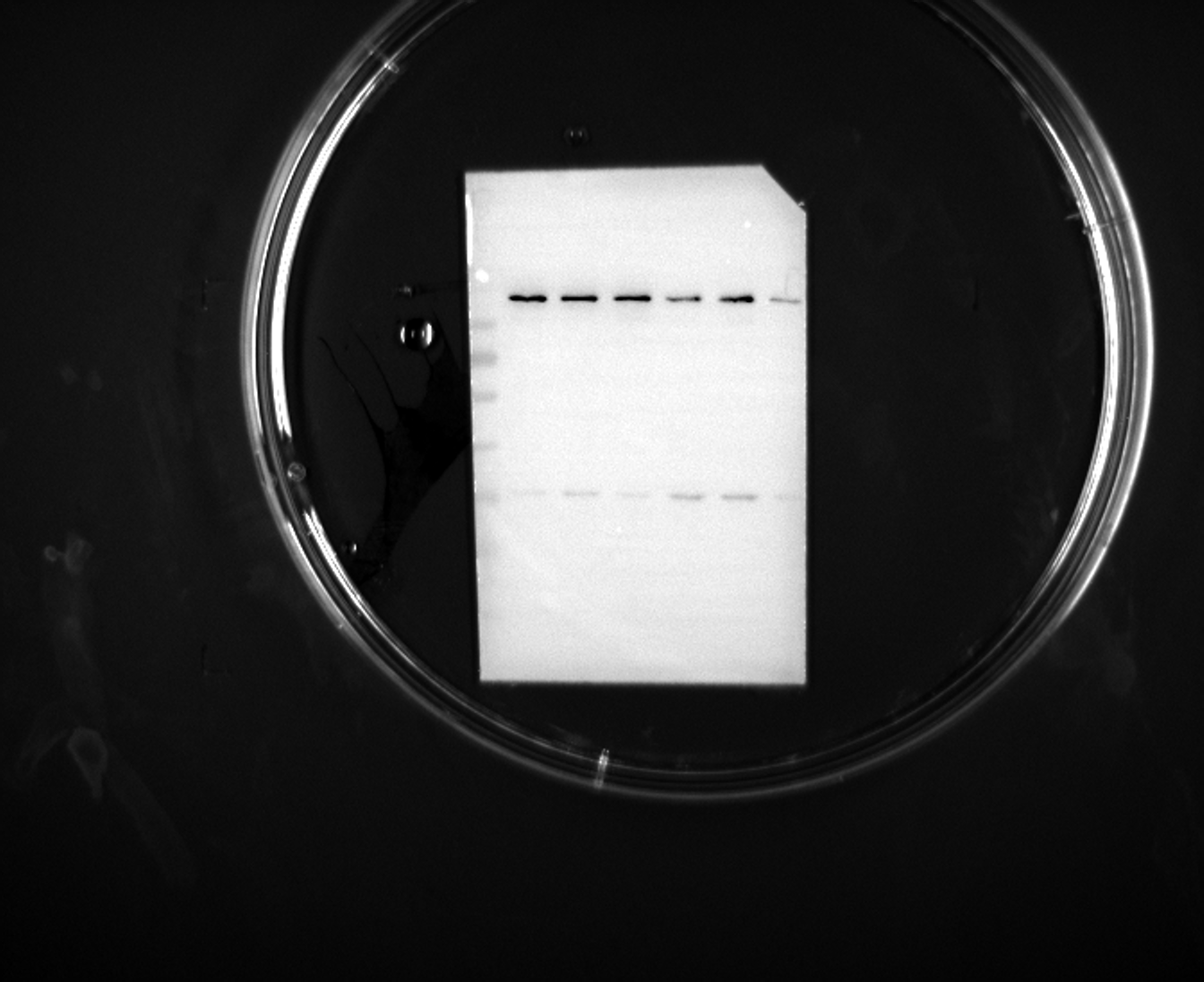

Supplement: Figure 2—source data 1. [file elife-82970-fig2-data1.zip › Figure_2-source_data_1/Figure_2-source_data_1_Figure_2C_BMP2.Tif]

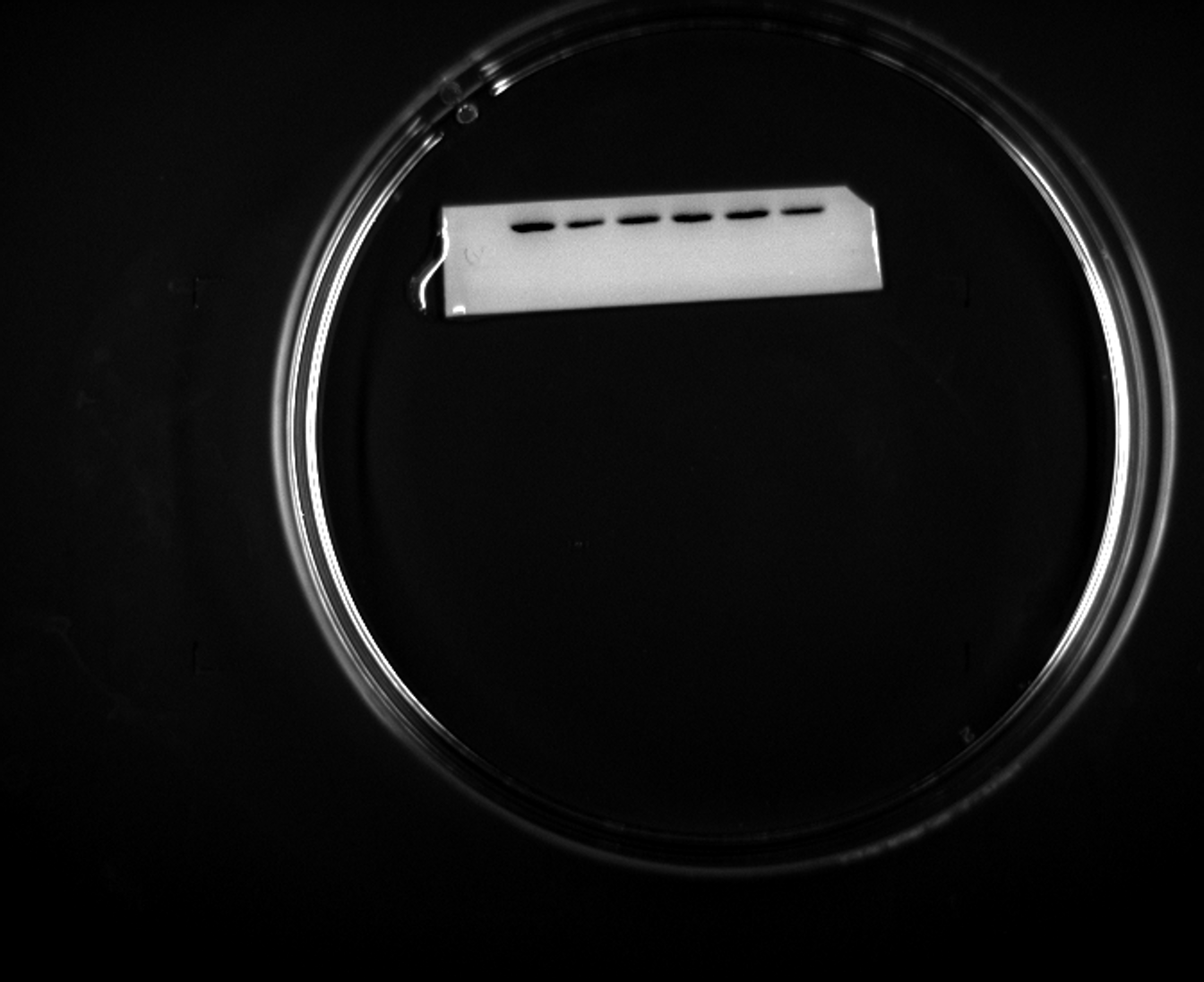

Supplement: Figure 2—source data 1. [file elife-82970-fig2-data1.zip › Figure_2-source_data_1/Figure_2-source_data_1_Figure_2C_CYCLIND3.Tif]

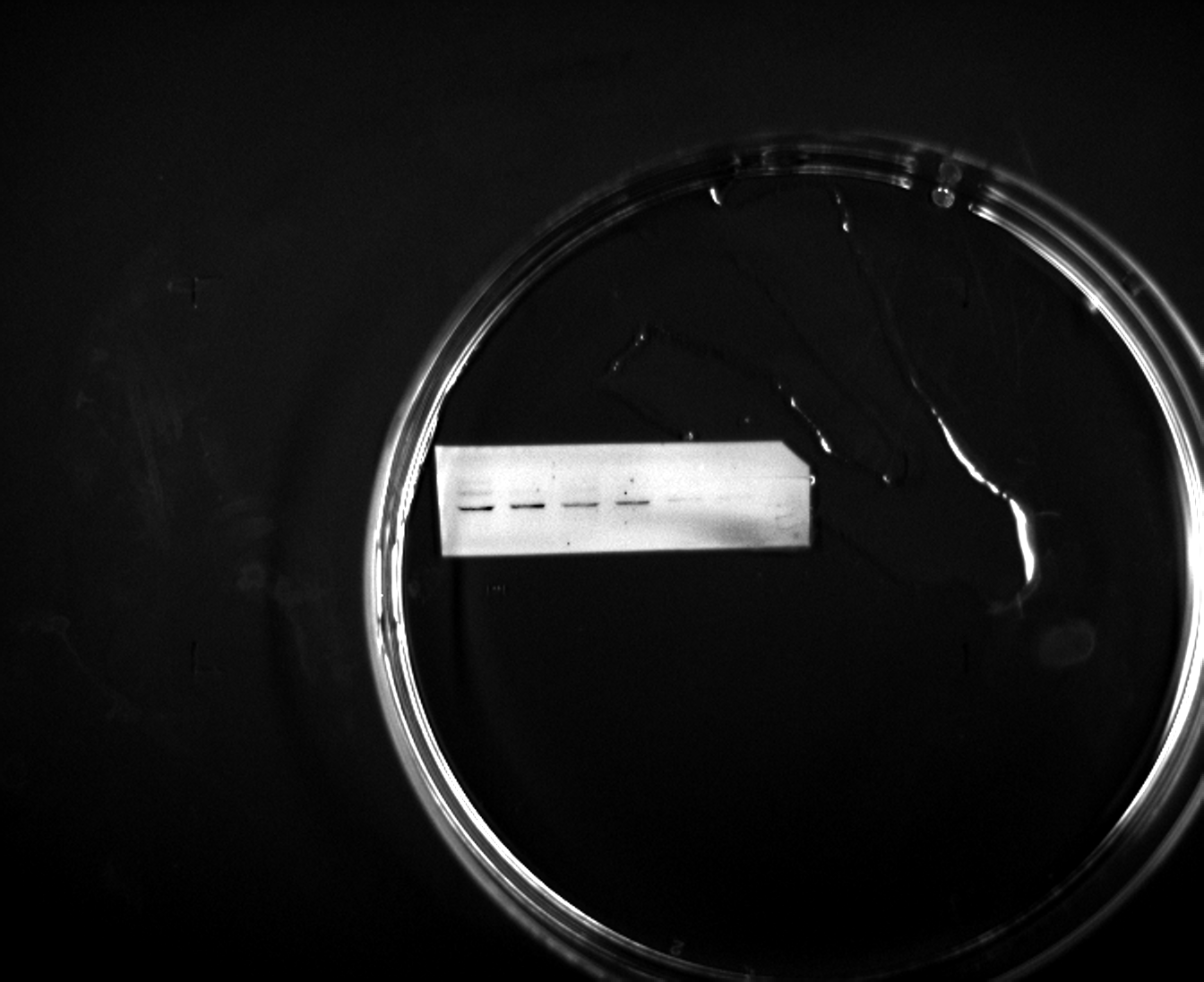

Supplement: Figure 2—source data 1. [file elife-82970-fig2-data1.zip › Figure_2-source_data_1/Figure_2-source_data_1_Figure_2C_E2F8.Tif]

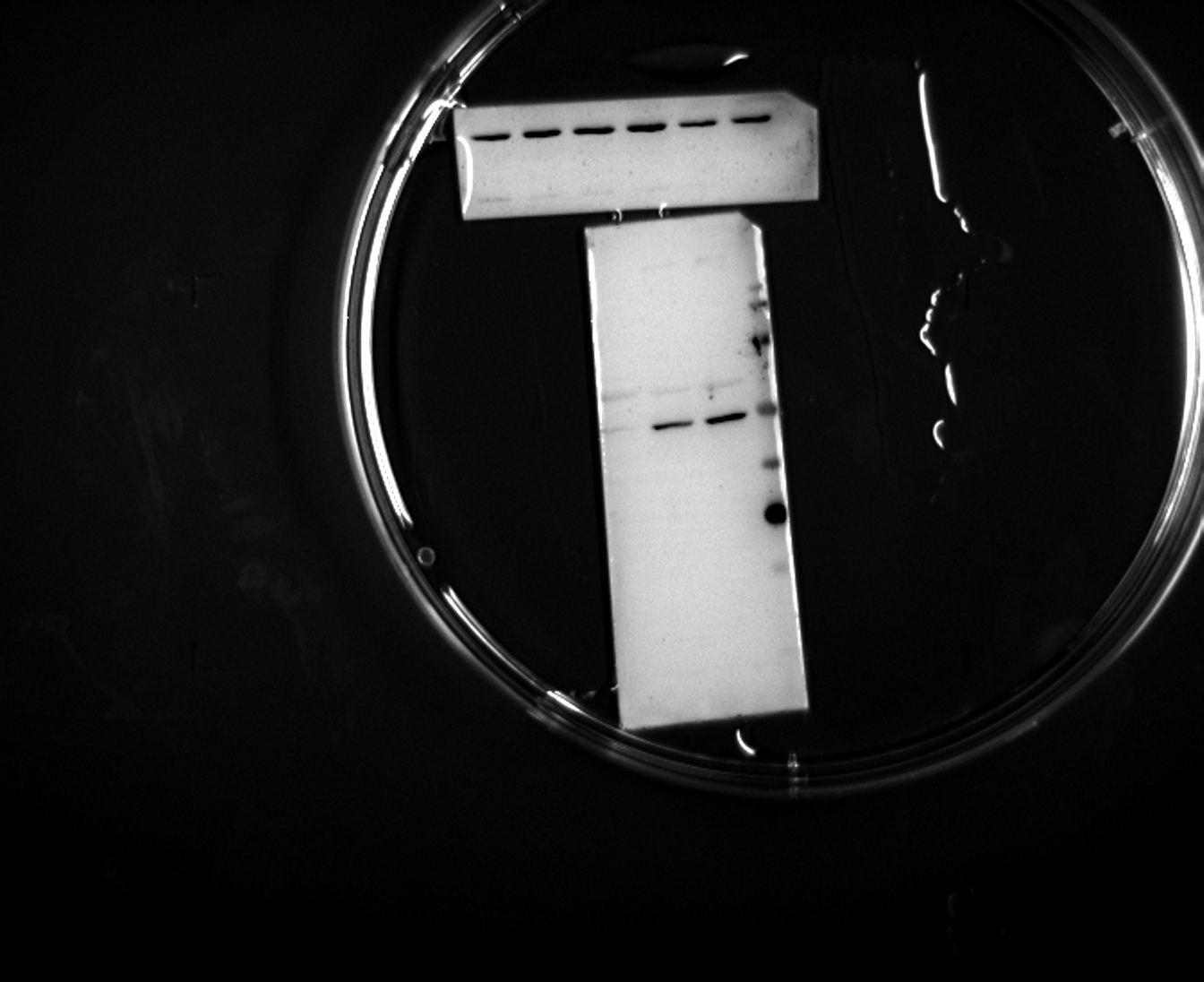

Supplement: Figure 2—source data 1. [file elife-82970-fig2-data1.zip › Figure_2-source_data_1/Figure_2-source_data_1_Figure_2C_GAPDH.Tif]

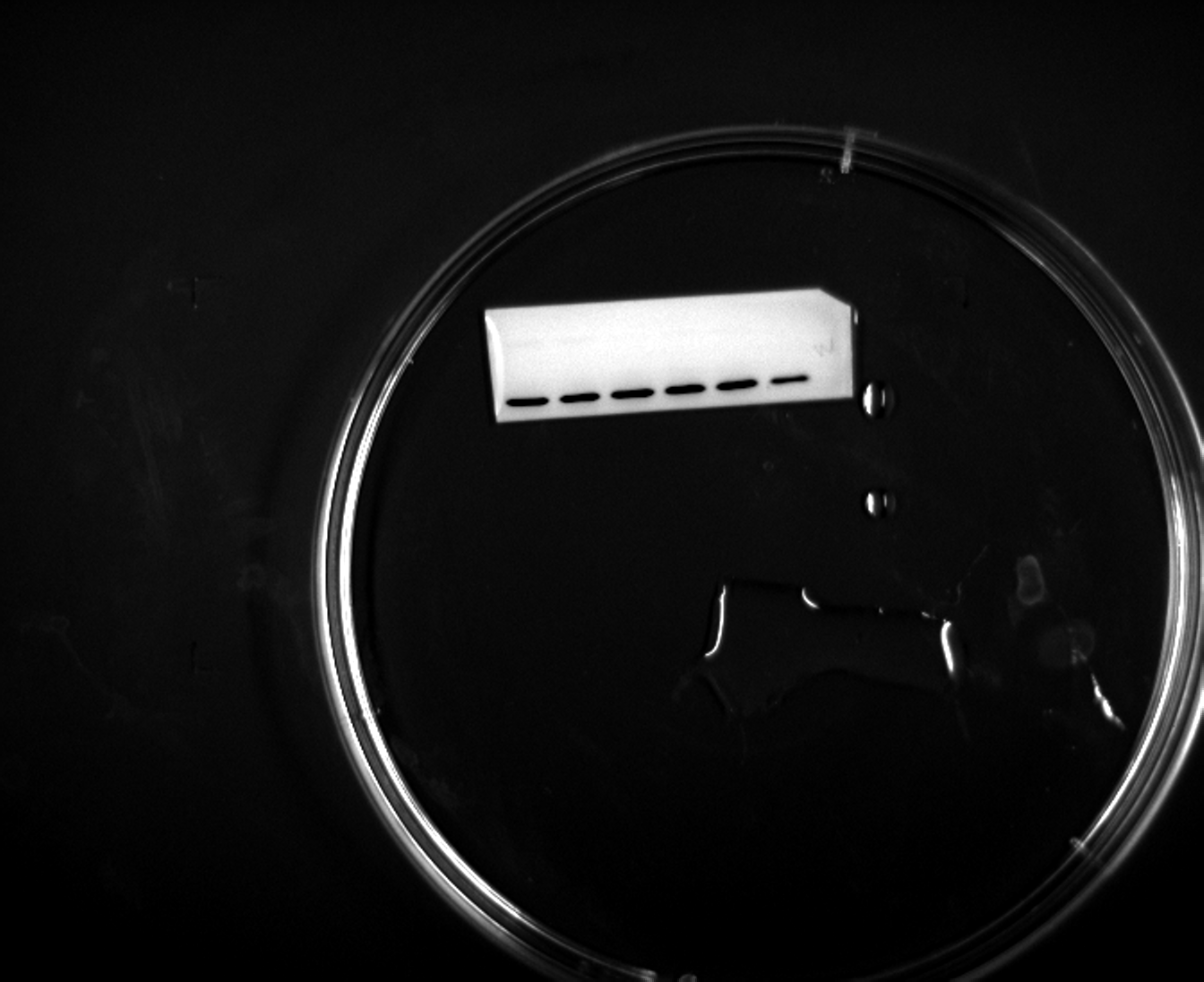

Supplement: Figure 2—source data 1. [file elife-82970-fig2-data1.zip › Figure_2-source_data_1/Figure_2-source_data_1_Figure_2C_WNT4.Tif]

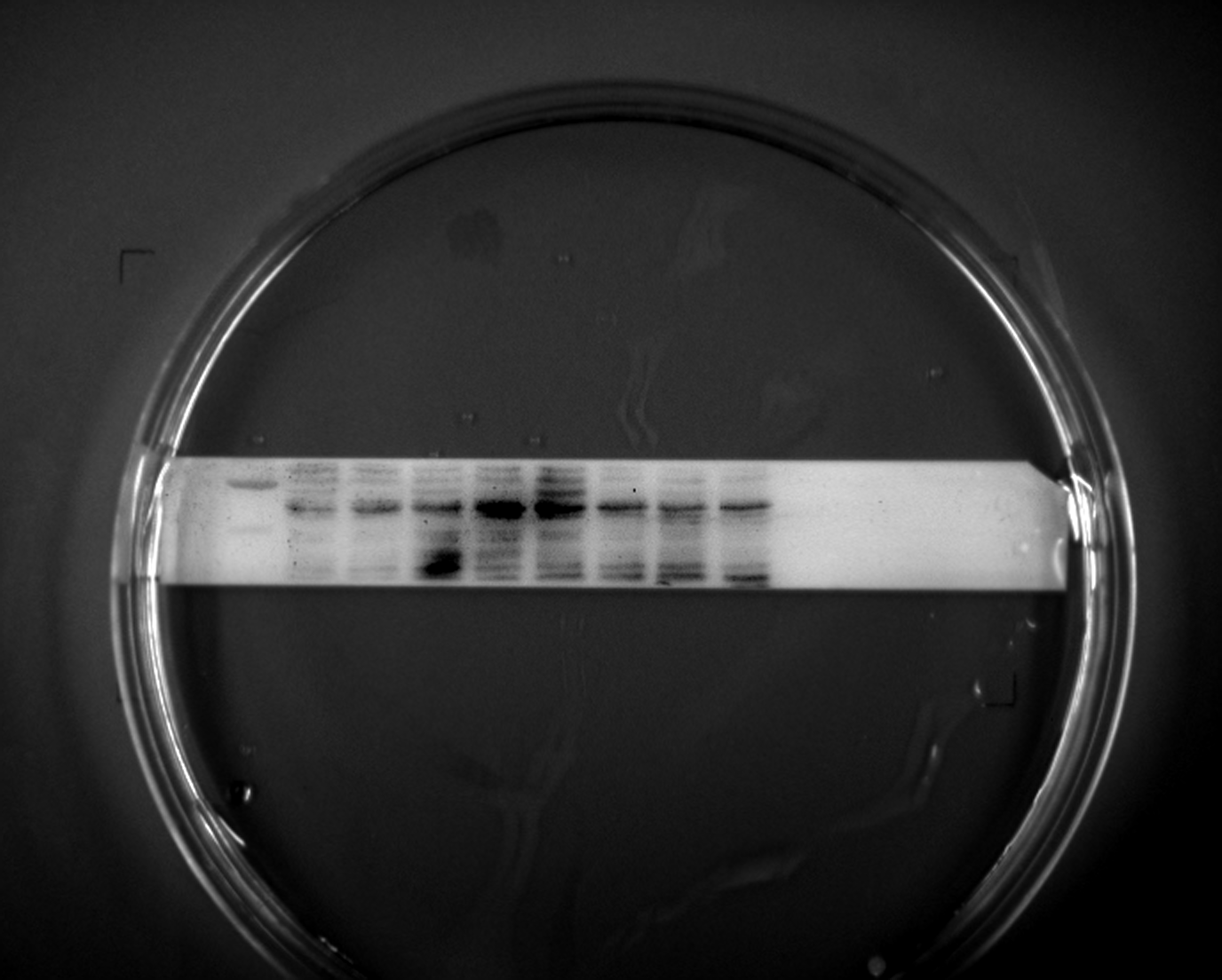

Supplement: Figure 2—source data 1. [file elife-82970-fig2-data1.zip › Figure_2-source_data_1/Figure_2-source_data_1_Figure_2D_ACTIVIN A.tif]

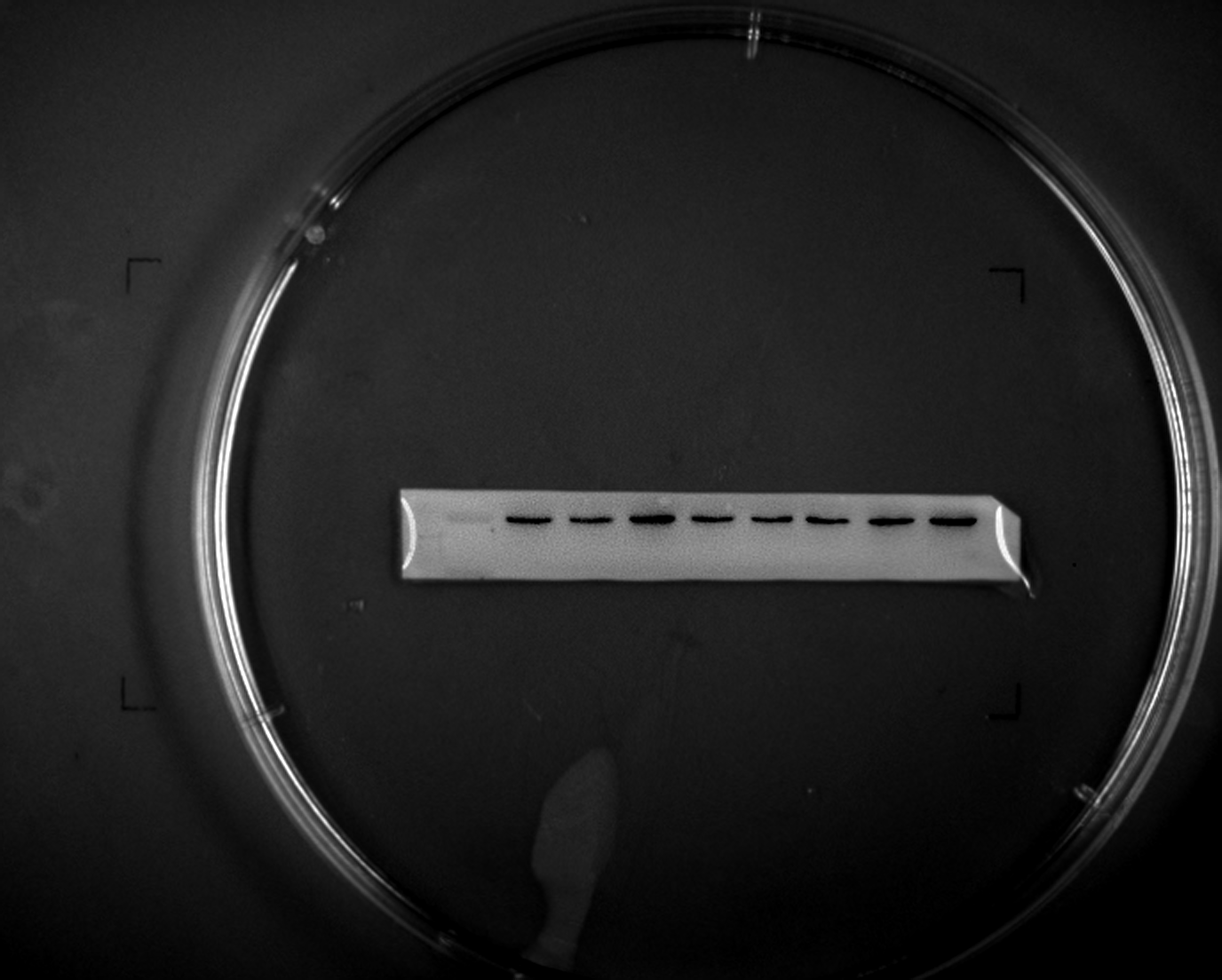

Supplement: Figure 2—source data 1. [file elife-82970-fig2-data1.zip › Figure_2-source_data_1/Figure_2-source_data_1_Figure_2D_TUBULIN.tif]

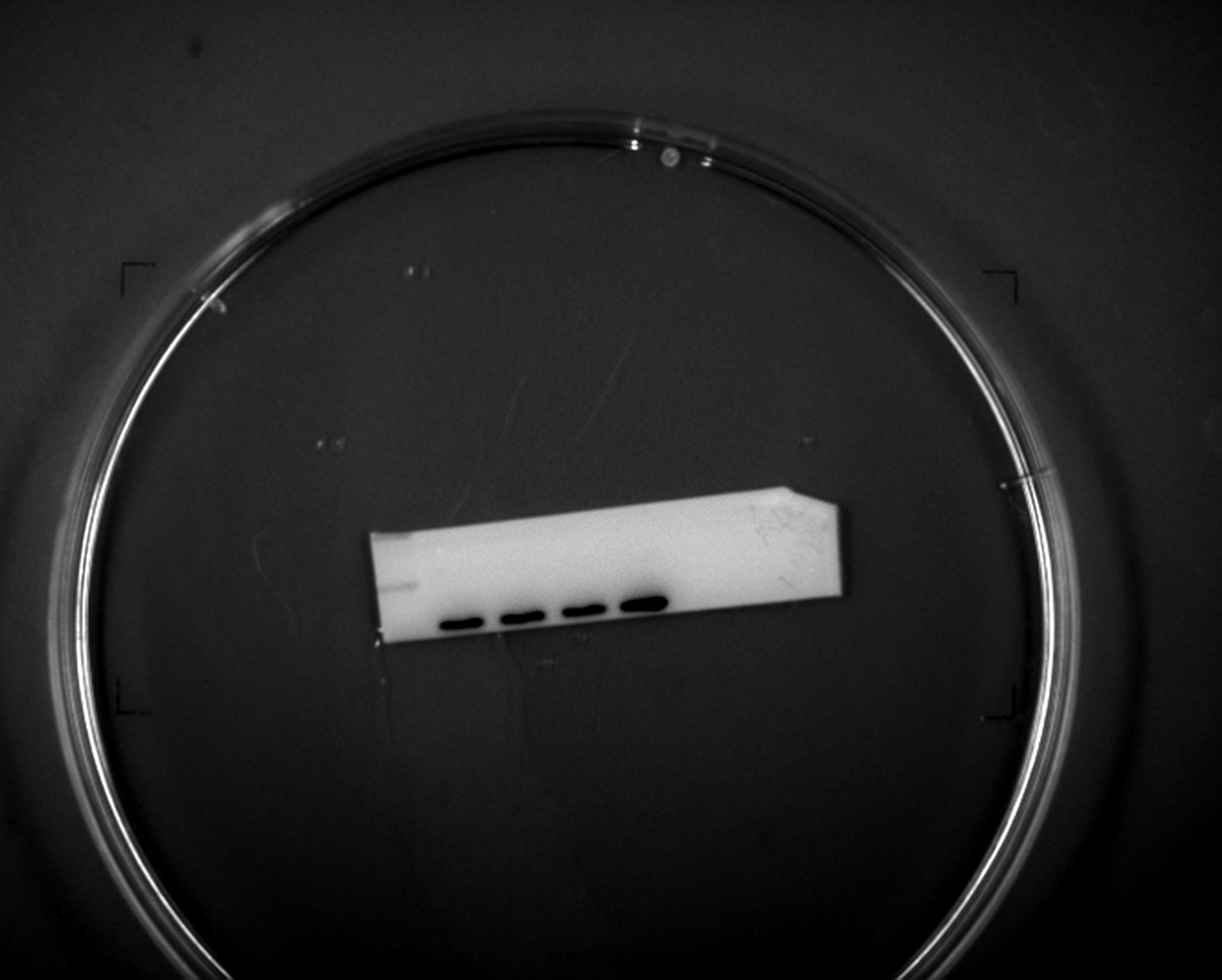

Supplement: Figure 2—source data 1. [file elife-82970-fig2-data1.zip › Figure_2-source_data_1/Figure_2-source_data_1_Figure_2E_BMP2.tif]

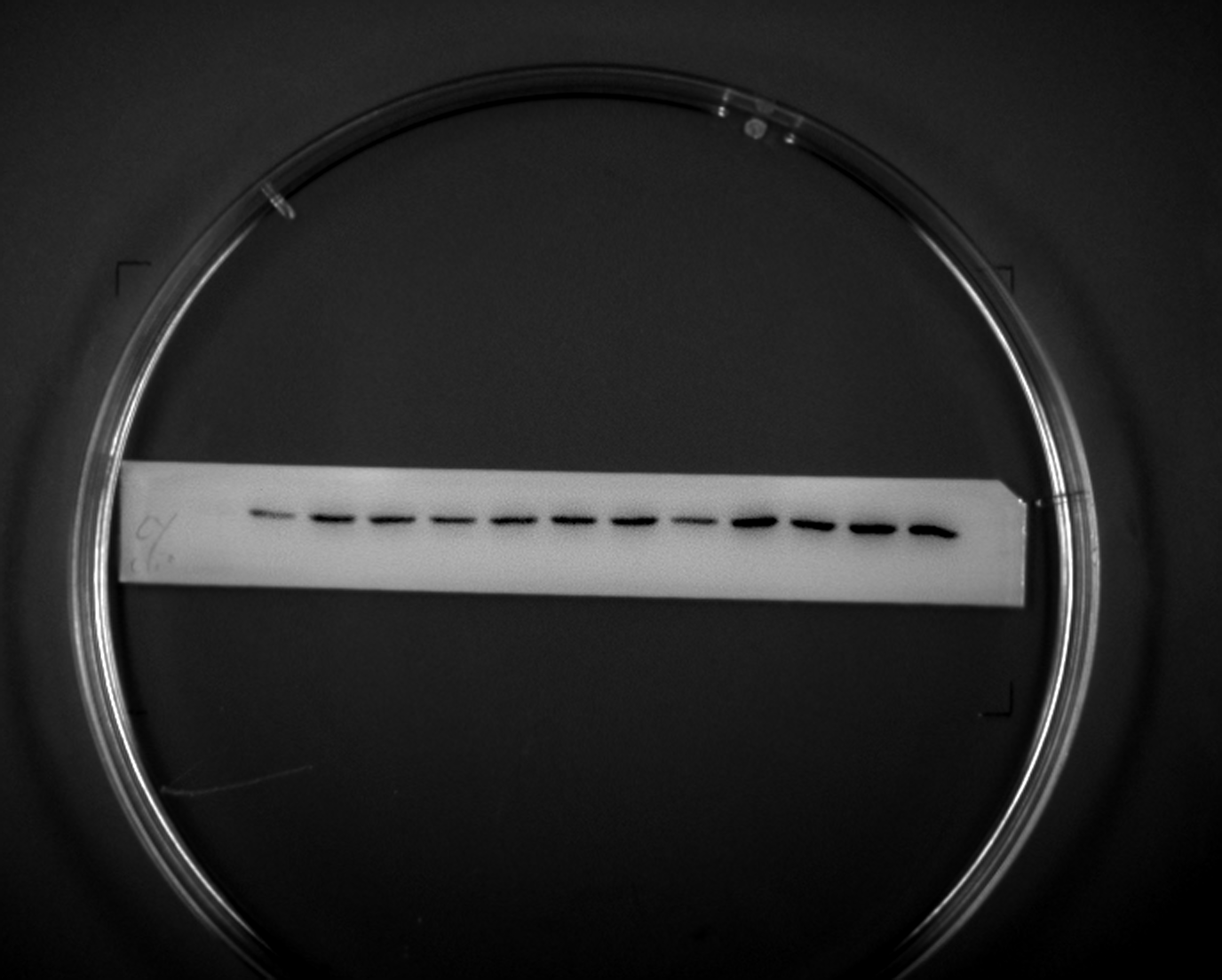

Supplement: Figure 2—source data 1. [file elife-82970-fig2-data1.zip › Figure_2-source_data_1/Figure_2-source_data_1_Figure_2E_CYCLIN D3.tif]

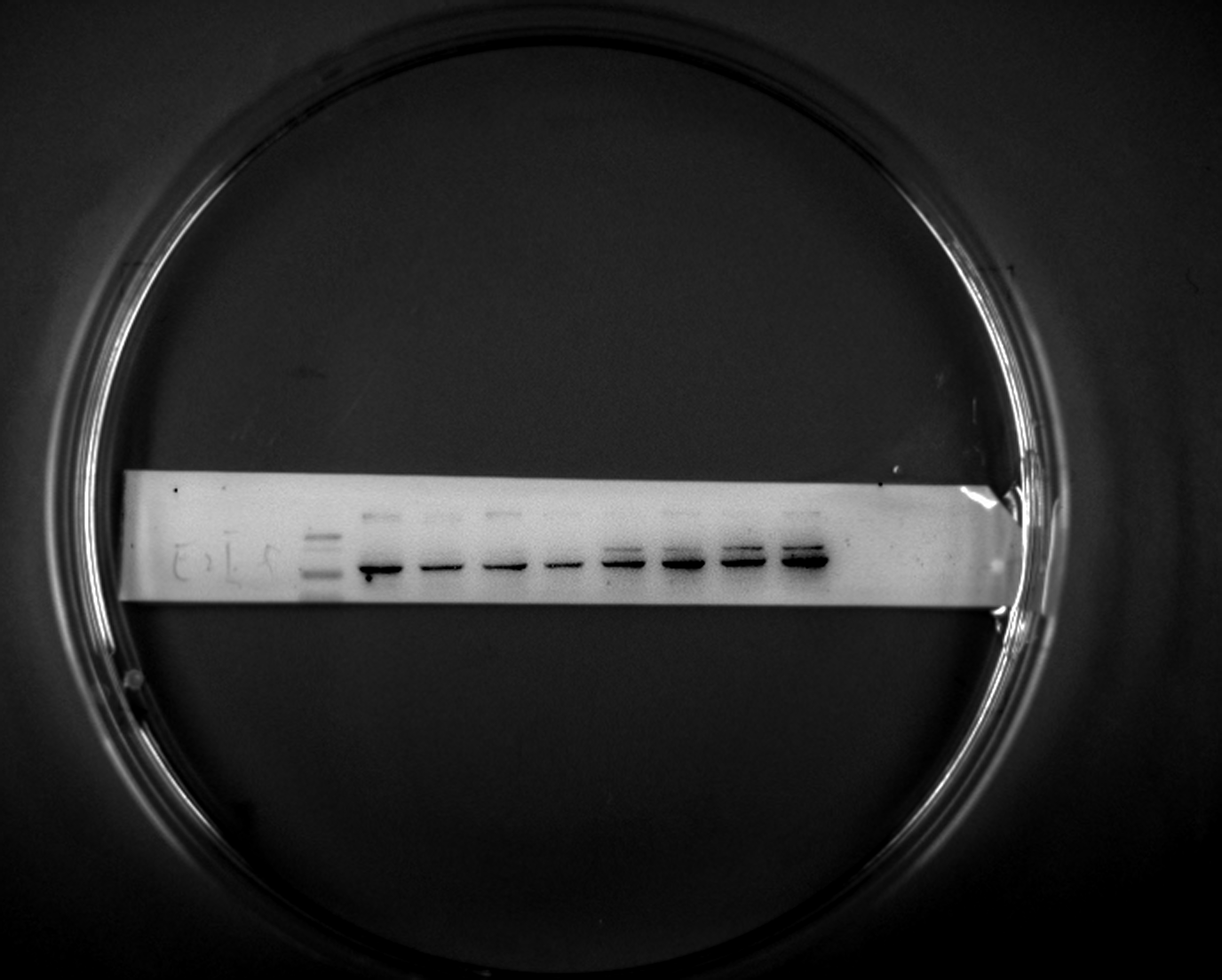

Supplement: Figure 2—source data 1. [file elife-82970-fig2-data1.zip › Figure_2-source_data_1/Figure_2-source_data_1_Figure_2E_E2F8.tif]

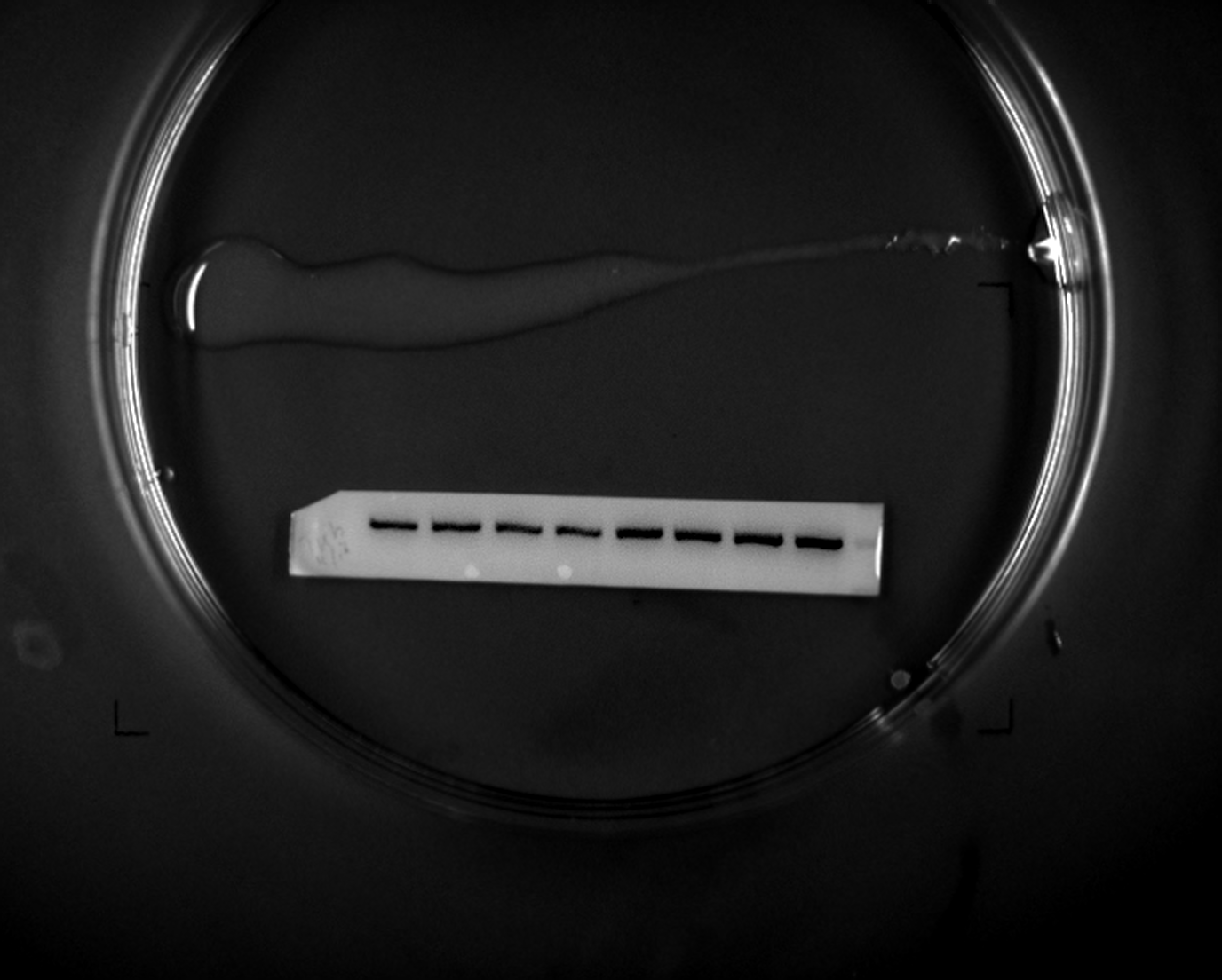

Supplement: Figure 2—source data 1. [file elife-82970-fig2-data1.zip › Figure_2-source_data_1/Figure_2-source_data_1_Figure_2E_TUBULIN.tif]

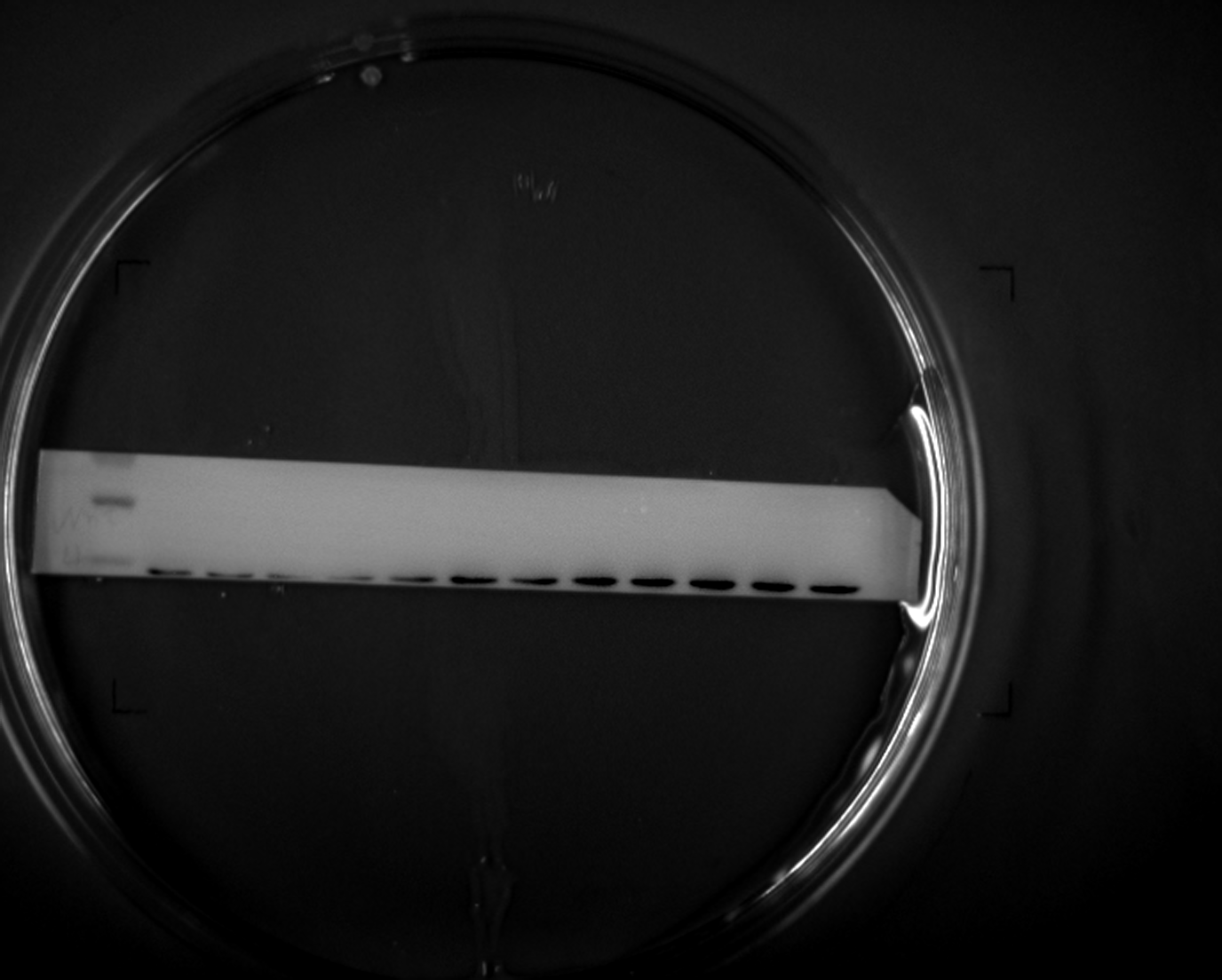

Supplement: Figure 2—source data 1. [file elife-82970-fig2-data1.zip › Figure_2-source_data_1/Figure_2-source_data_1_Figure_2E_WNT4.tif]

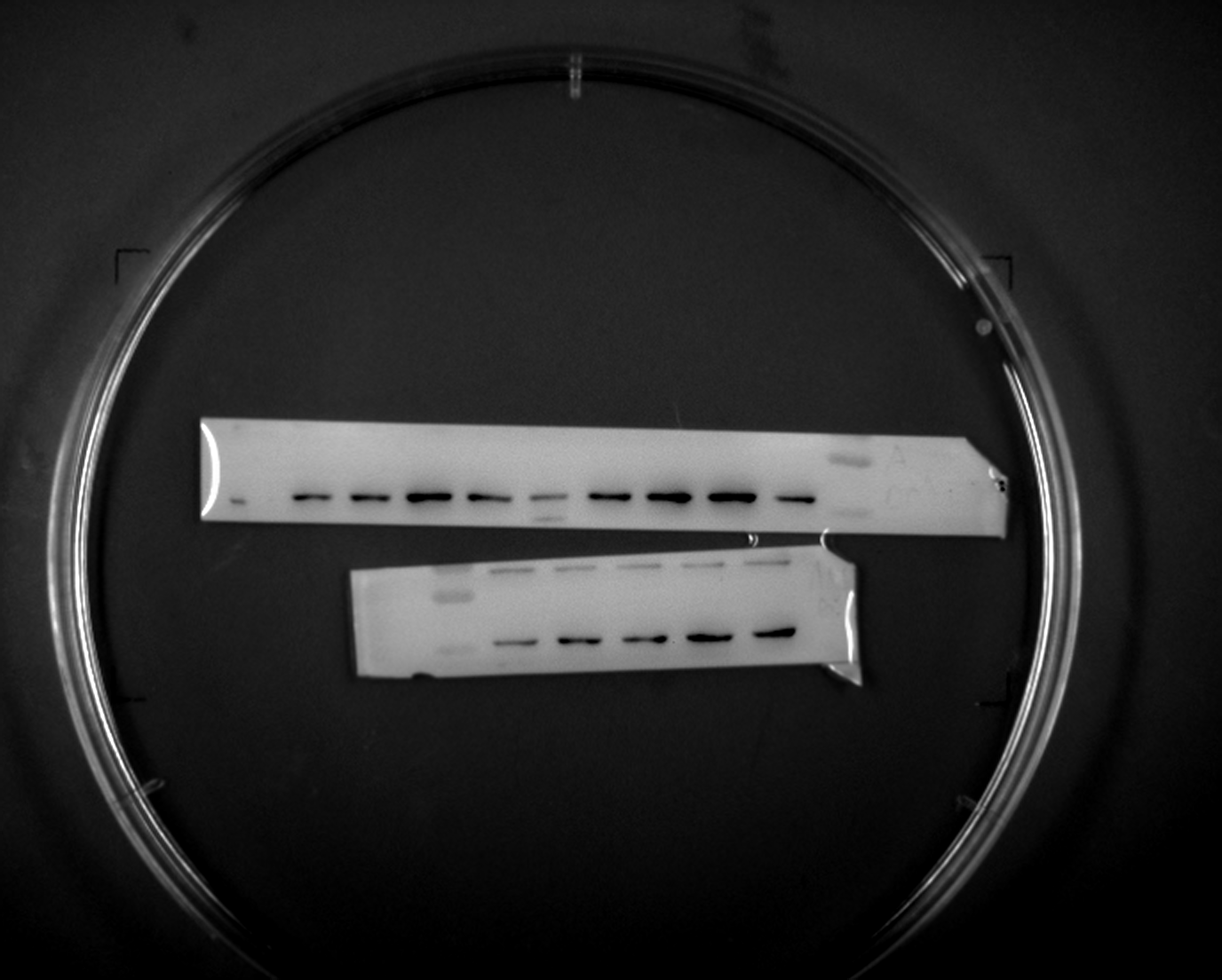

Supplement: Figure 2—source data 1. [file elife-82970-fig2-data1.zip › Figure_2-source_data_1/Figure_2-source_data_1_Figure_2F_BMP2.tif]

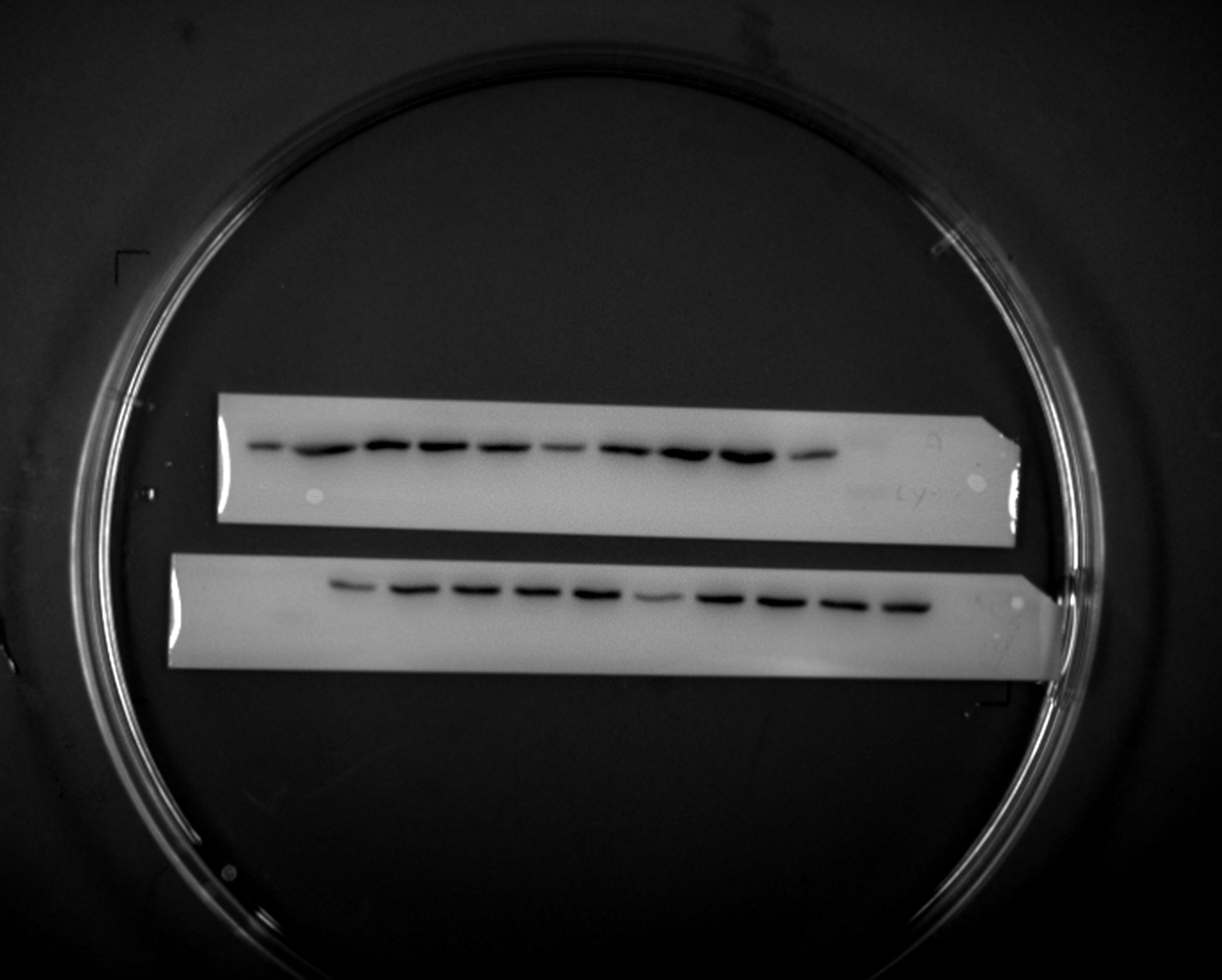

Supplement: Figure 2—source data 1. [file elife-82970-fig2-data1.zip › Figure_2-source_data_1/Figure_2-source_data_1_Figure_2F_CYCLIN D3.tif]

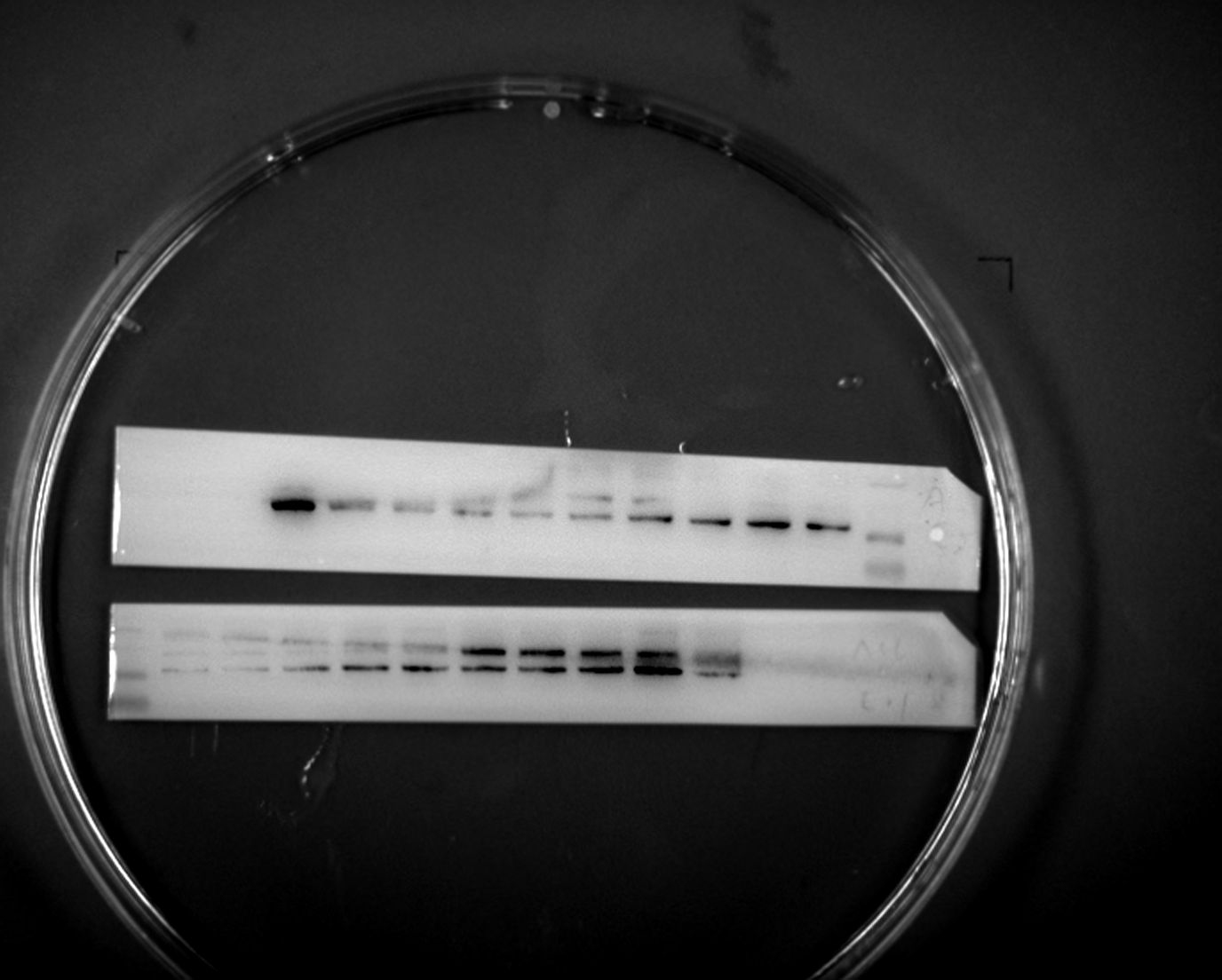

Supplement: Figure 2—source data 1. [file elife-82970-fig2-data1.zip › Figure_2-source_data_1/Figure_2-source_data_1_Figure_2F_E2F8.tif]

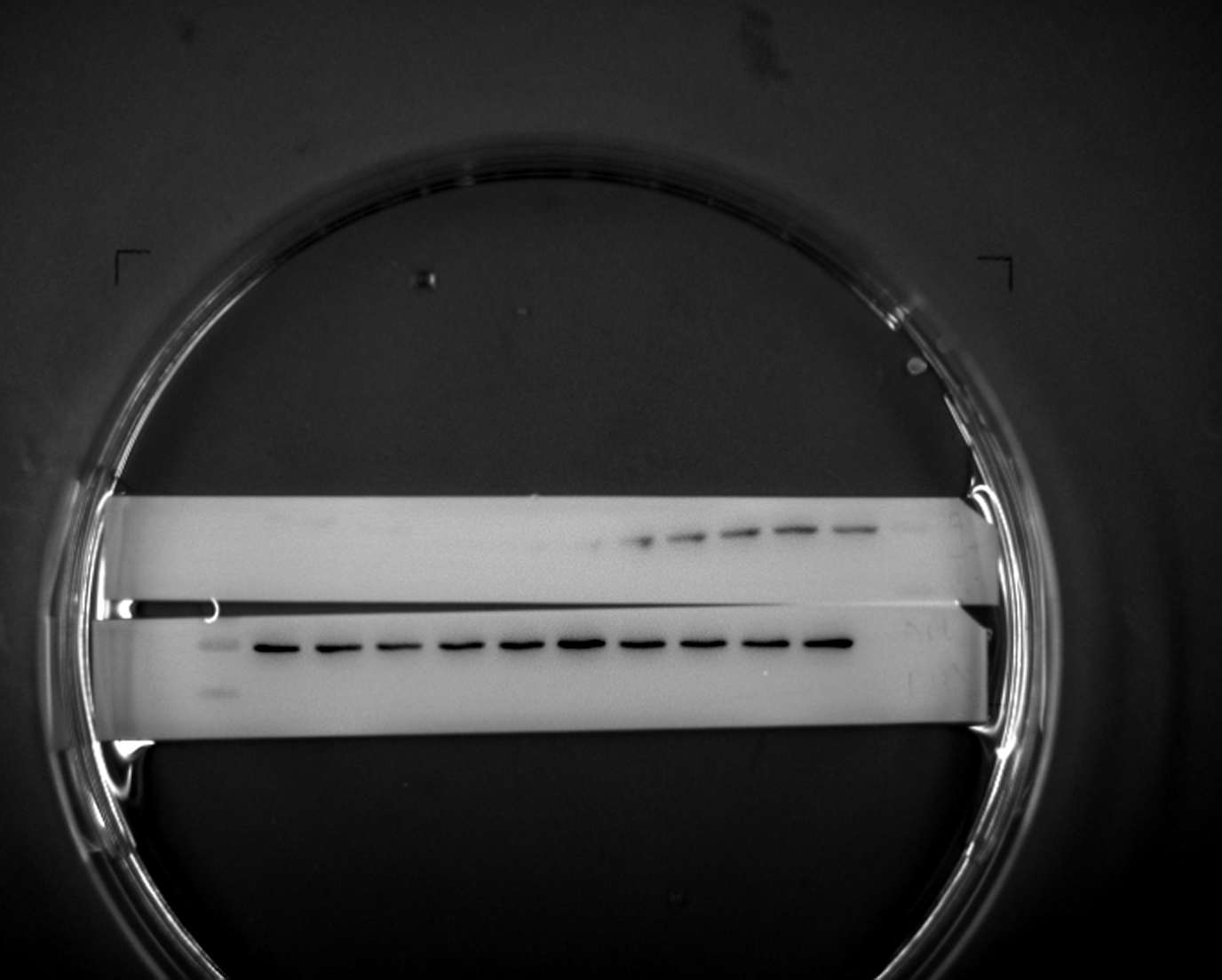

Supplement: Figure 2—source data 1. [file elife-82970-fig2-data1.zip › Figure_2-source_data_1/Figure_2-source_data_1_Figure_2F_TUBULIN.tif]

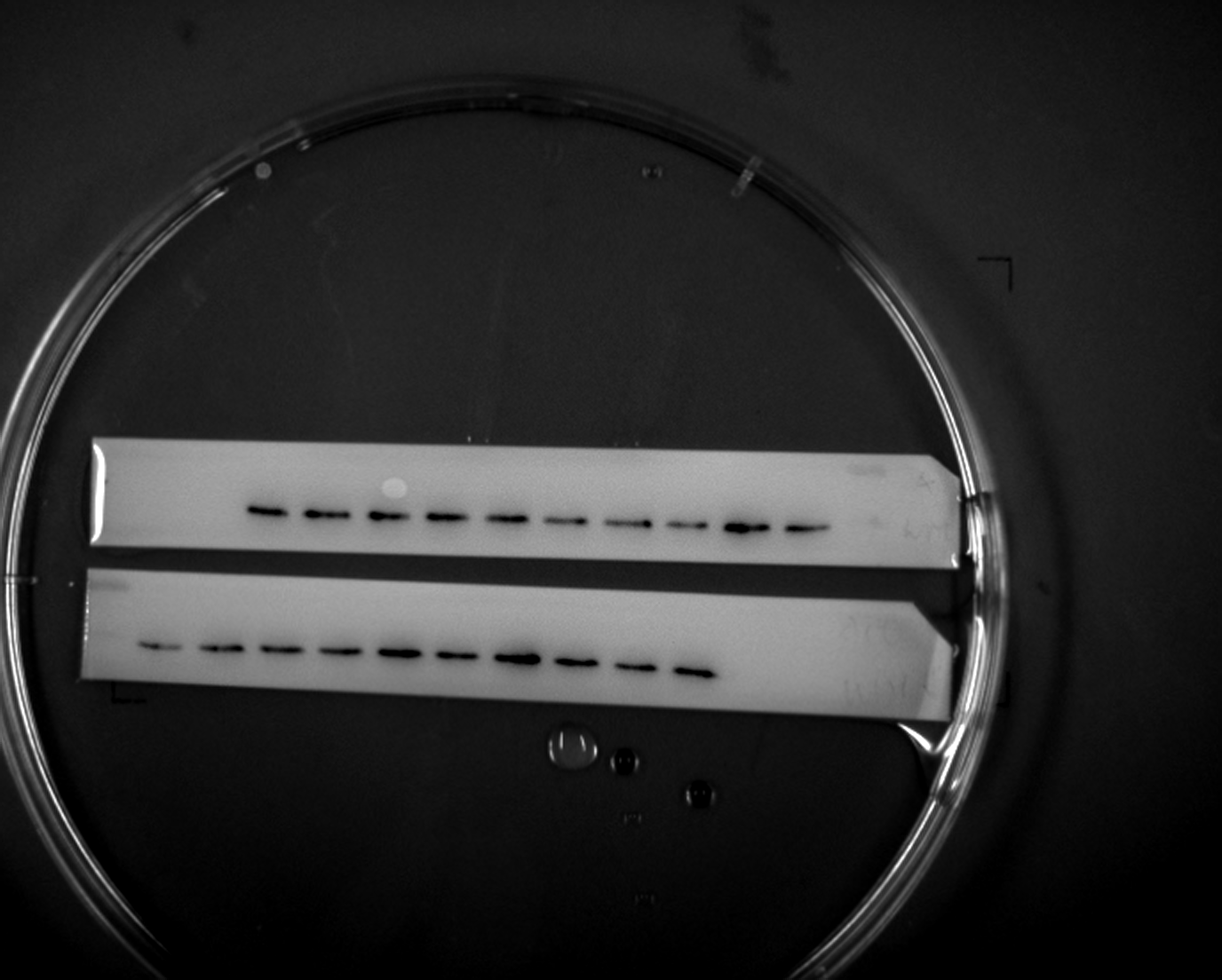

Supplement: Figure 2—source data 1. [file elife-82970-fig2-data1.zip › Figure_2-source_data_1/Figure_2-source_data_1_Figure_2F_WNT4.tif]

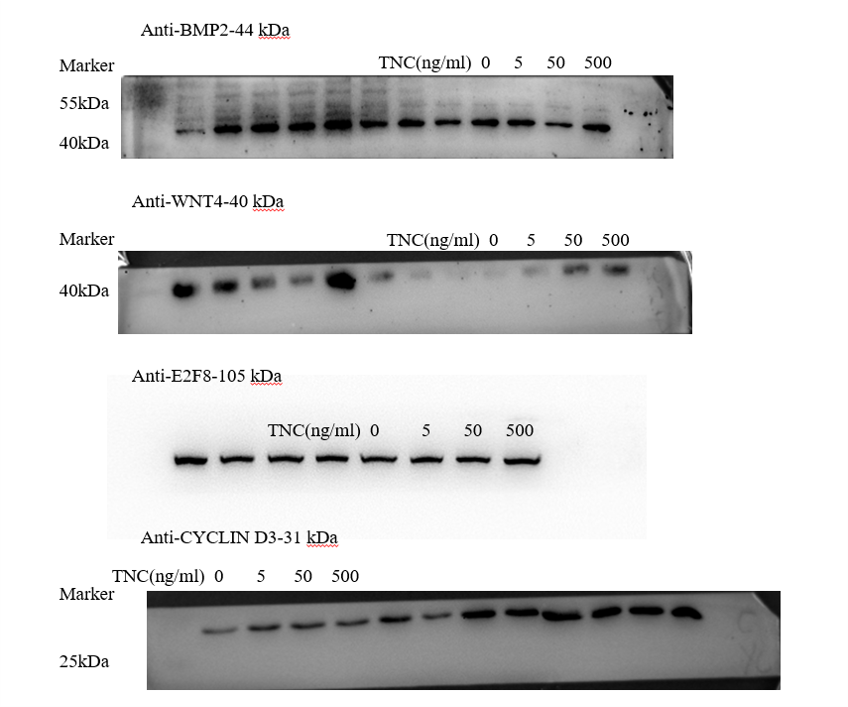

Supplement: Figure 2—source data 2. [file elife-82970-fig2-data2.zip › Figure_2-source_data_2/Figure_2-source_data_2-2A.png]

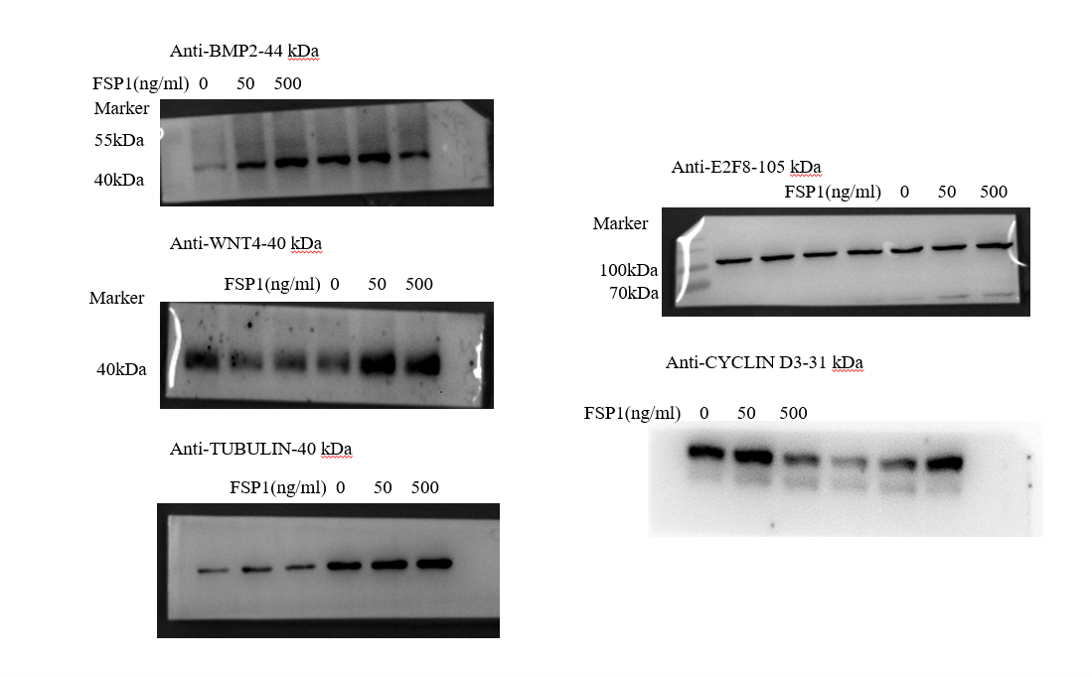

Supplement: Figure 2—source data 2. [file elife-82970-fig2-data2.zip › Figure_2-source_data_2/Figure_2-source_data_2-2B.png]

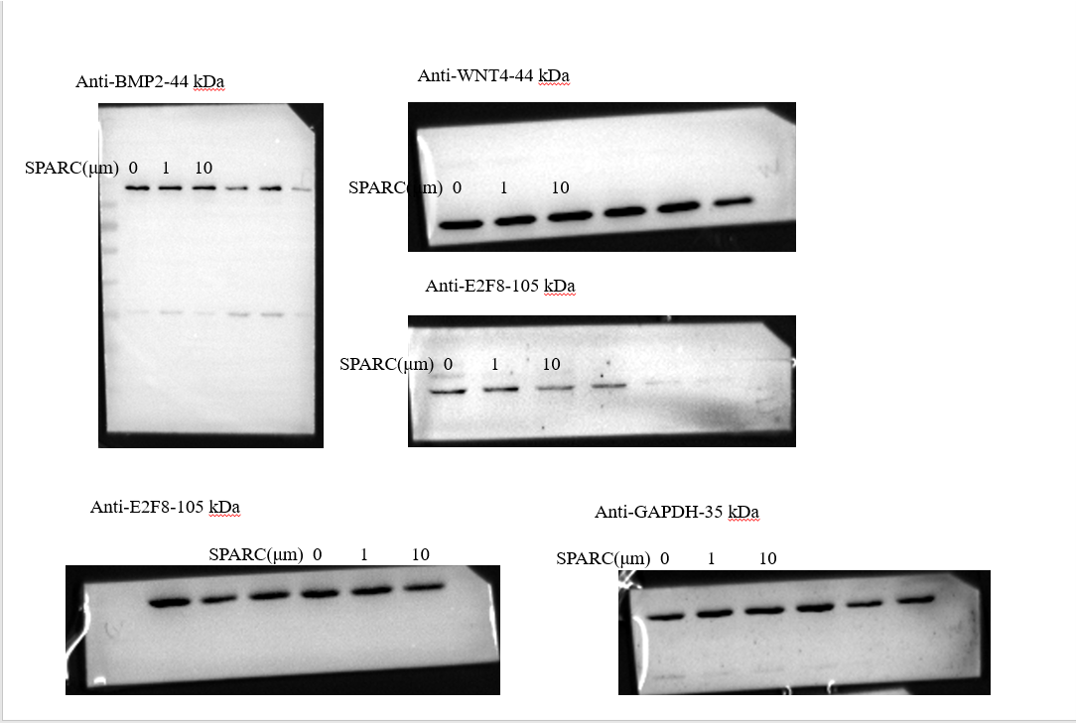

Supplement: Figure 2—source data 2. [file elife-82970-fig2-data2.zip › Figure_2-source_data_2/Figure_2-source_data_2-2C.png]

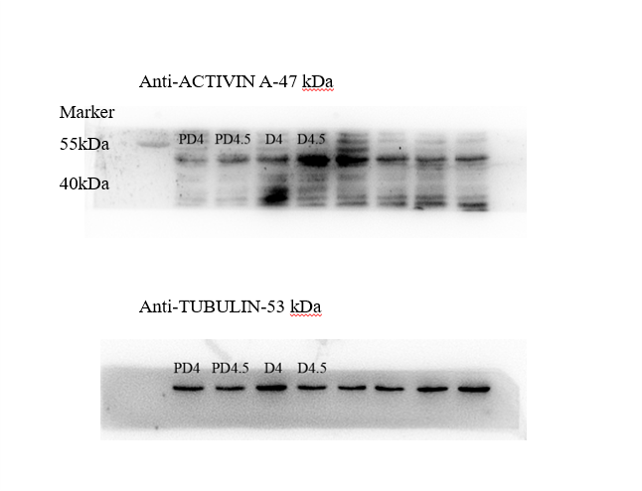

Supplement: Figure 2—source data 2. [file elife-82970-fig2-data2.zip › Figure_2-source_data_2/Figure_2-source_data_2-2D.png]

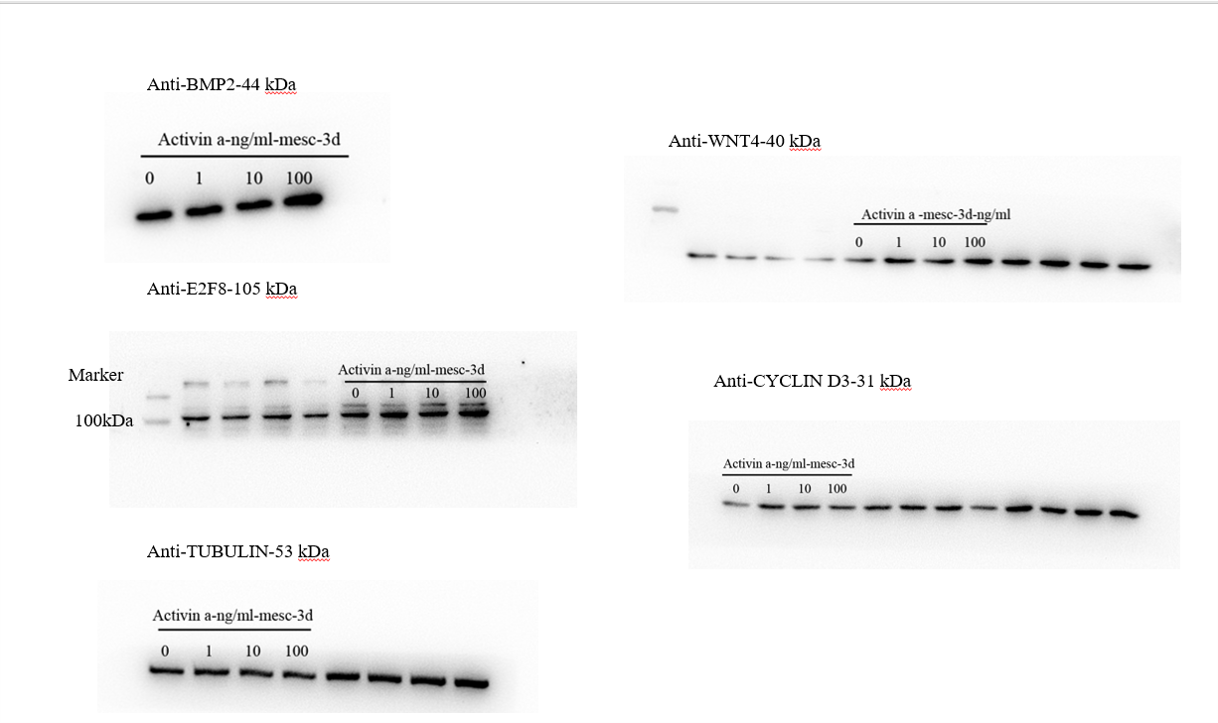

Supplement: Figure 2—source data 2. [file elife-82970-fig2-data2.zip › Figure_2-source_data_2/Figure_2-source_data_2-2E.png]

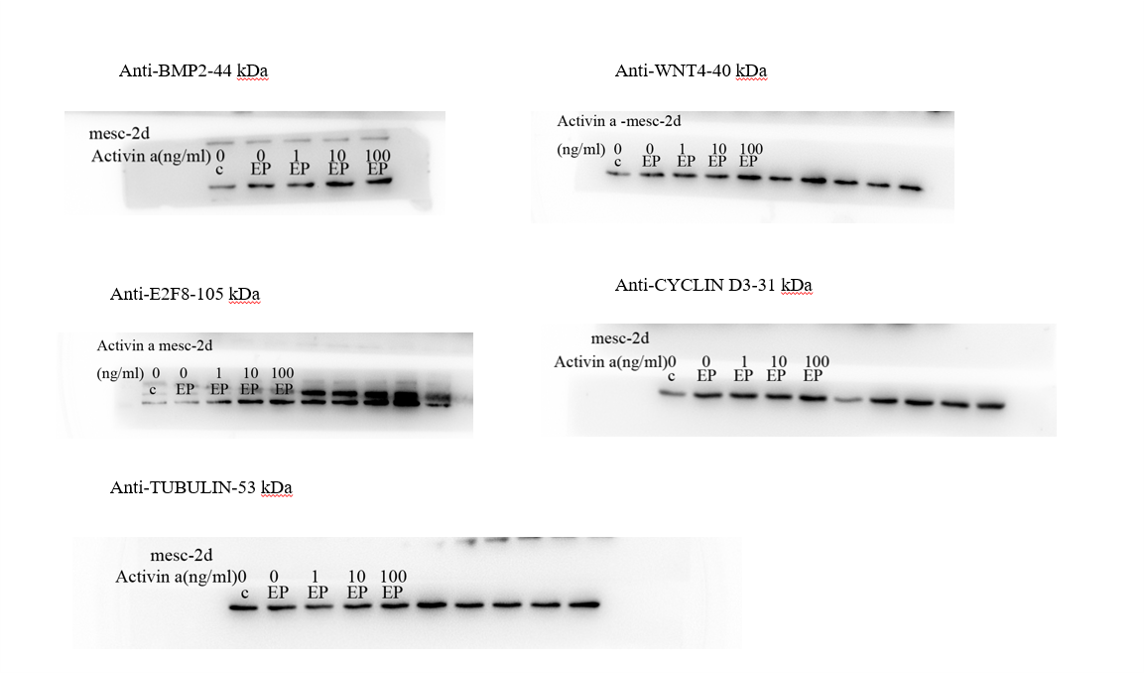

Supplement: Figure 2—source data 2. [file elife-82970-fig2-data2.zip › Figure_2-source_data_2/Figure_2-source_data_2-2F.png]

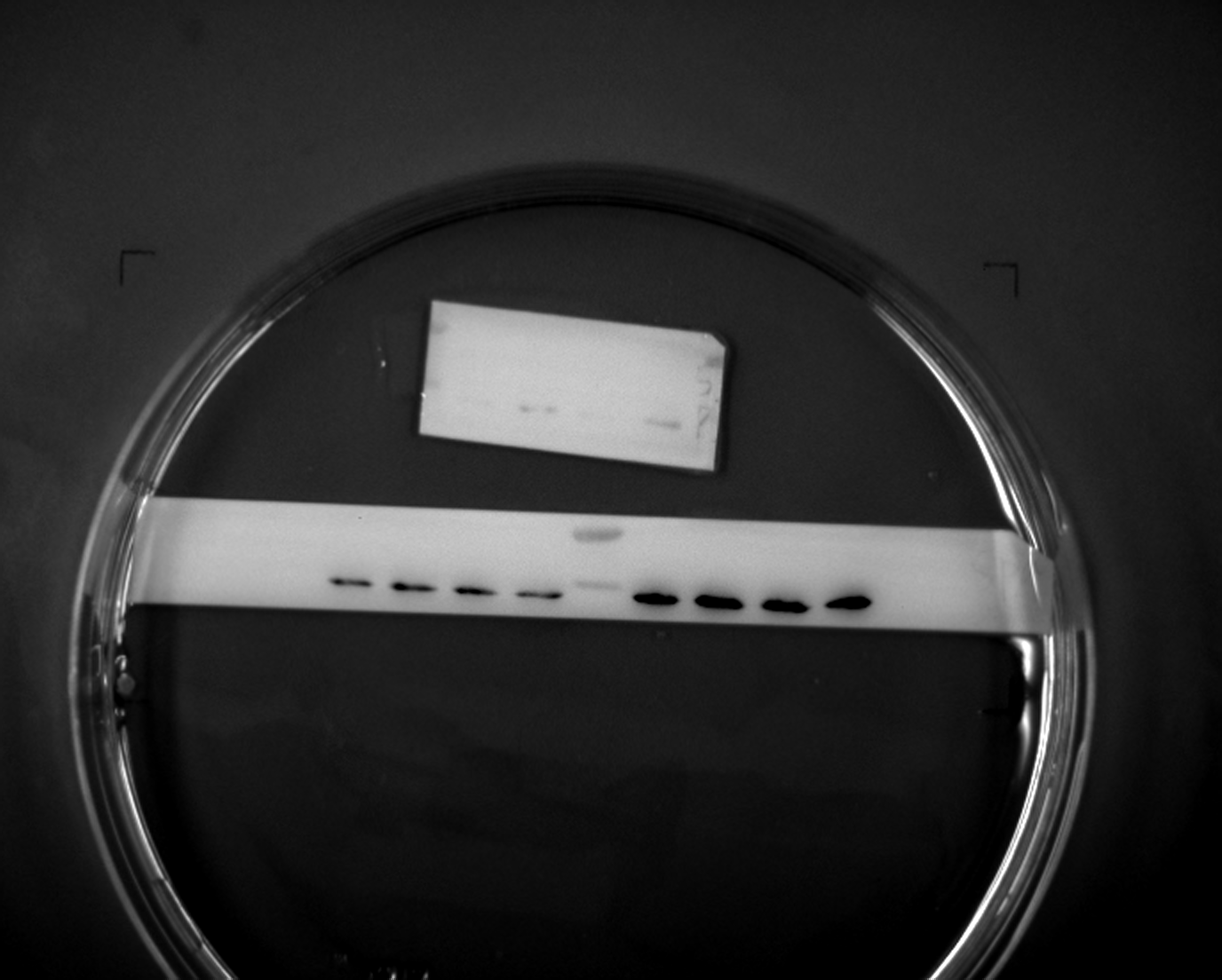

Supplement: Figure 3—source data 1. [file elife-82970-fig3-data1.zip › Figure_3-source_data_1/Figure_3-source_data_1_Figure_3A_SPARC.tif]

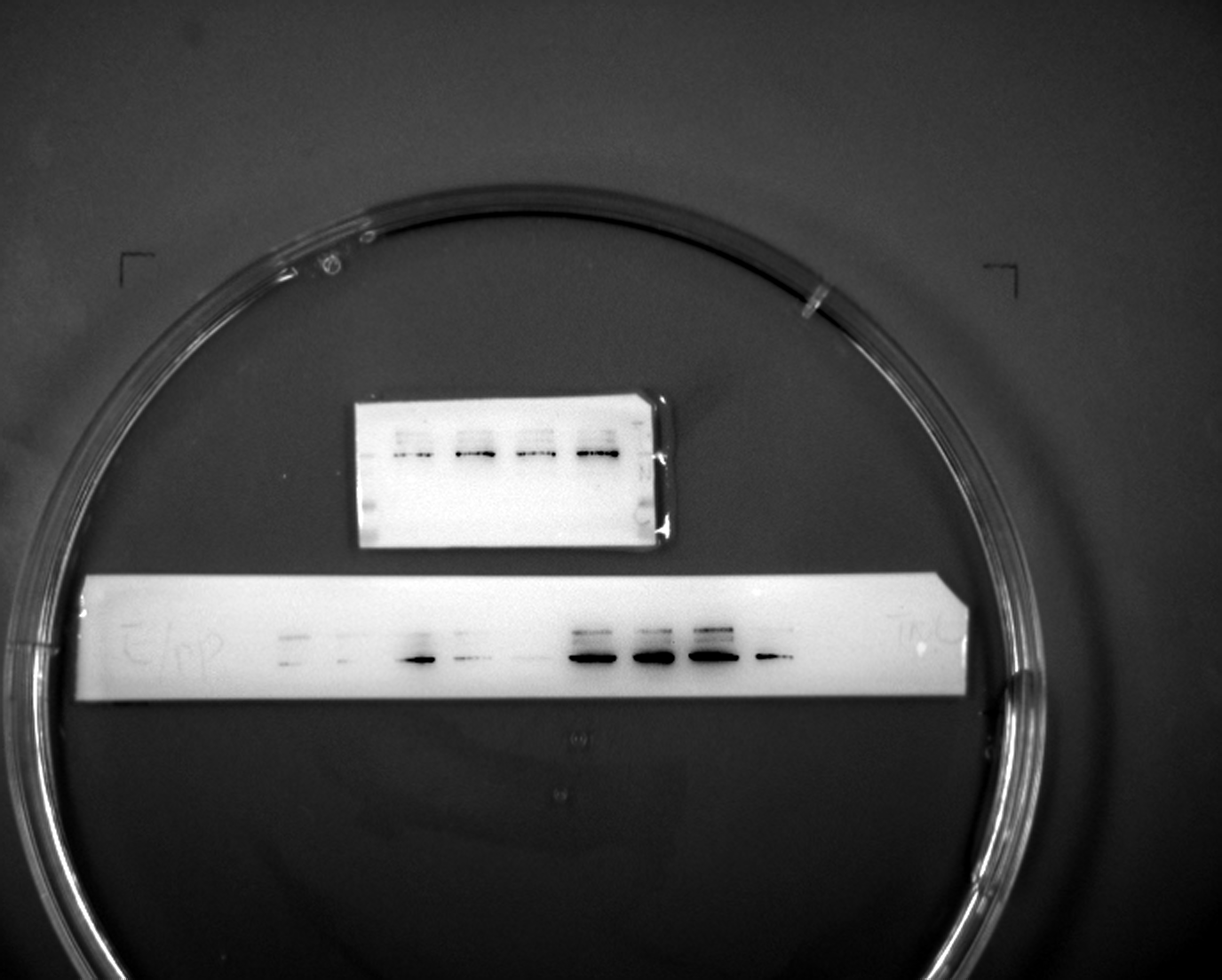

Supplement: Figure 3—source data 1. [file elife-82970-fig3-data1.zip › Figure_3-source_data_1/Figure_3-source_data_1_Figure_3A_TNC.tif]

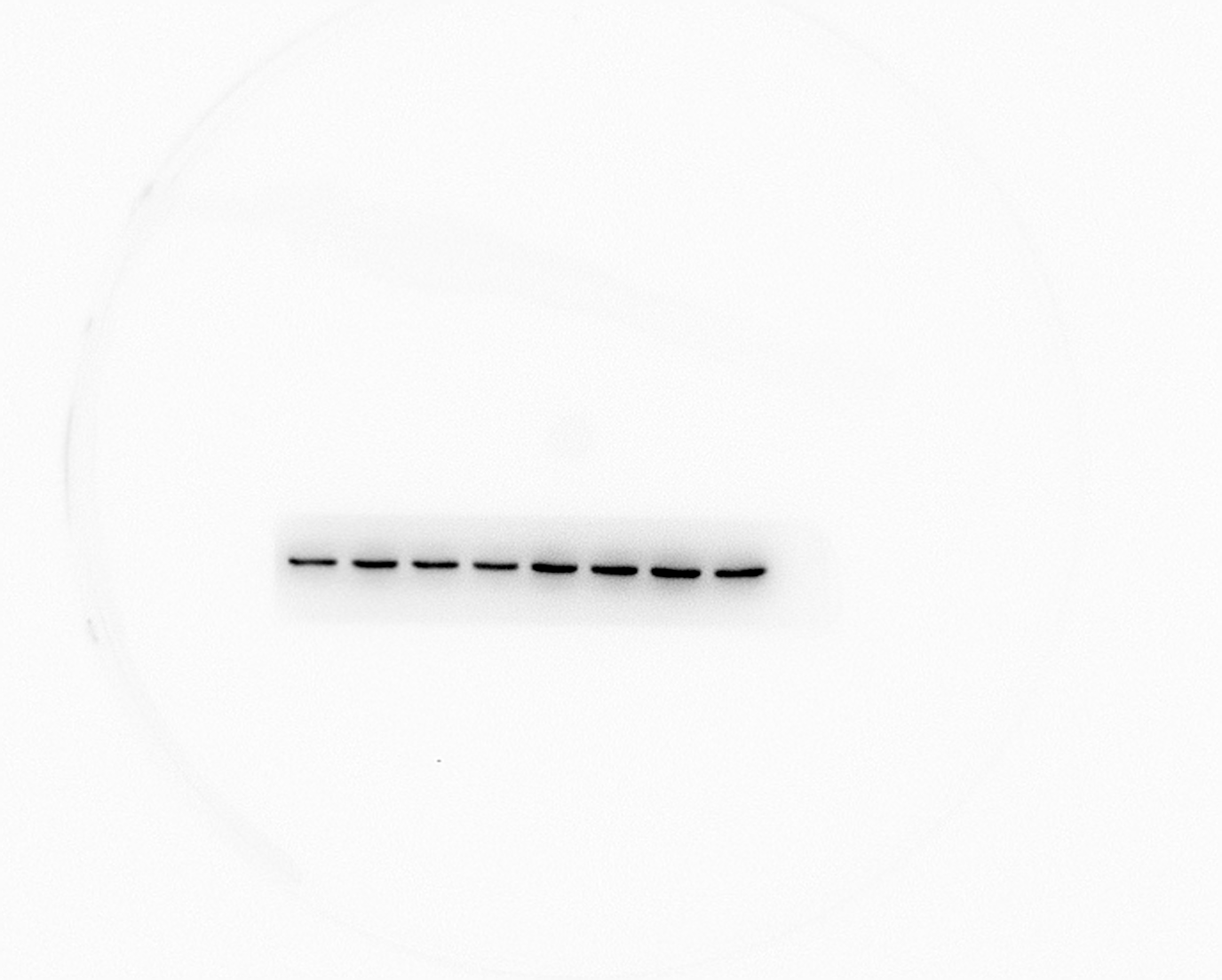

Supplement: Figure 3—source data 1. [file elife-82970-fig3-data1.zip › Figure_3-source_data_1/Figure_3-source_data_1_Figure_3A_TUBULIN.tif]

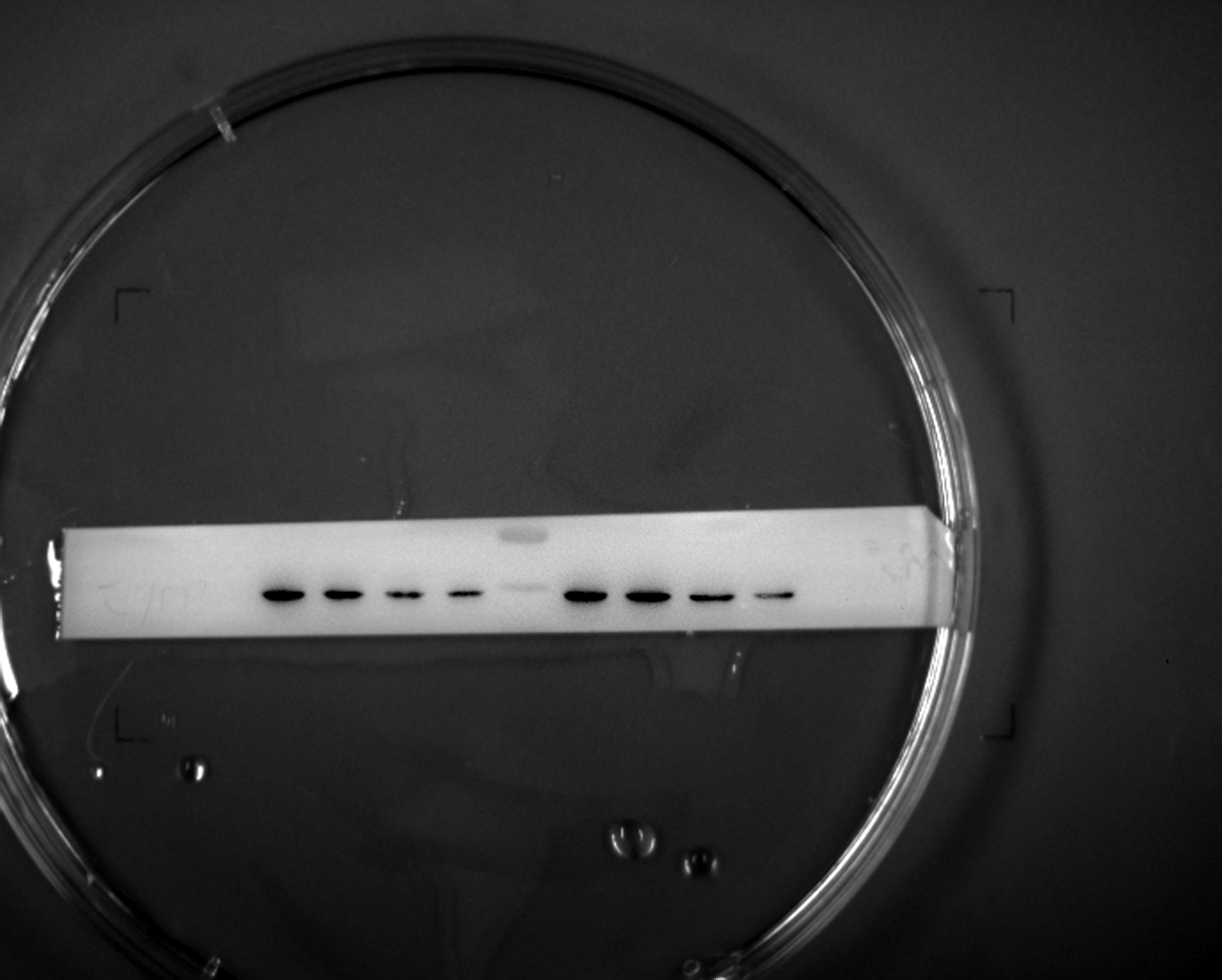

Supplement: Figure 3—source data 1. [file elife-82970-fig3-data1.zip › Figure_3-source_data_1/Figure_3-source_data_1_Figure_3A_a┴-SMA.tif]

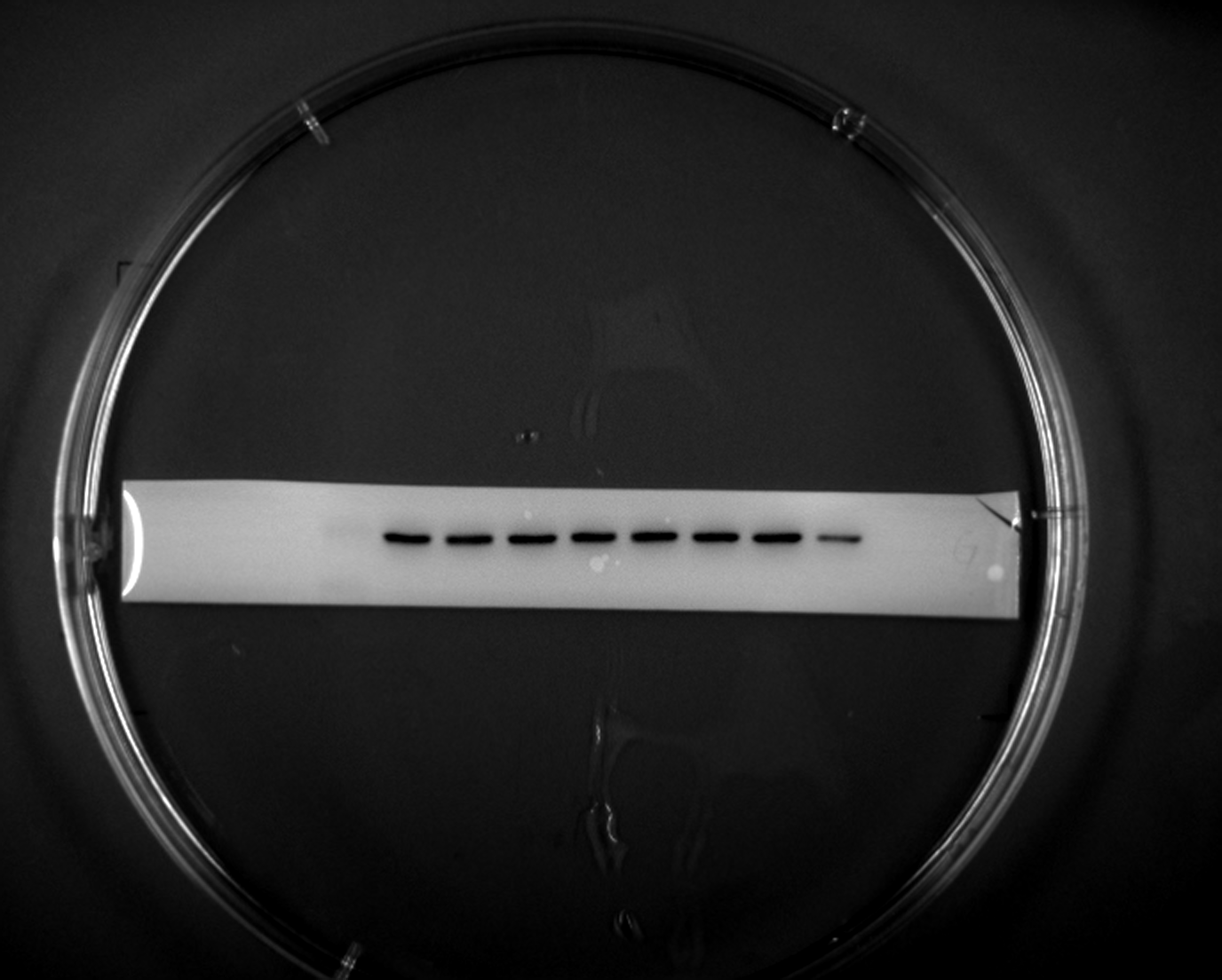

Supplement: Figure 3—source data 1. [file elife-82970-fig3-data1.zip › Figure_3-source_data_1/Figure_3-source_data_1_Figure_3B_GAPDH.tif]

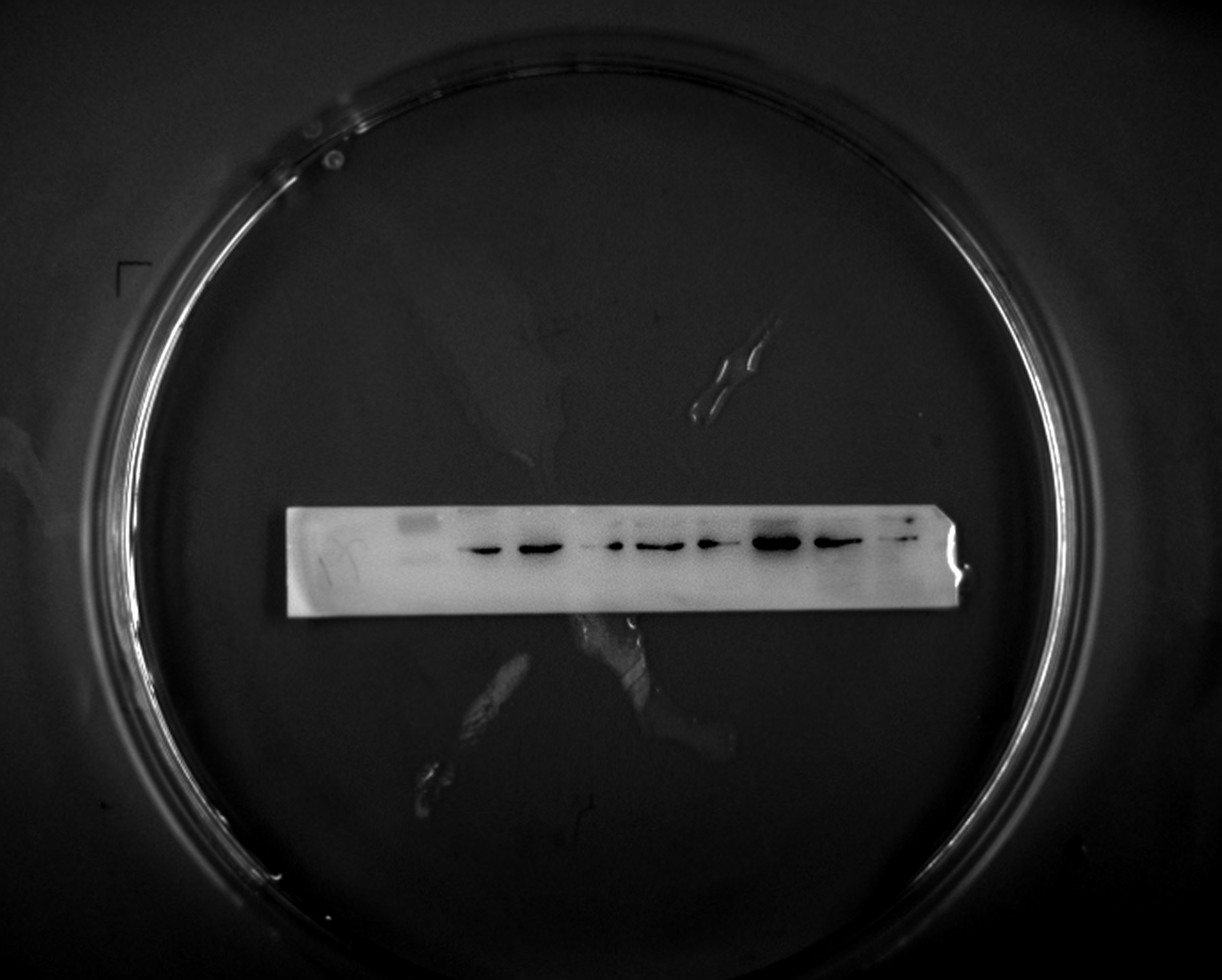

Supplement: Figure 3—source data 1. [file elife-82970-fig3-data1.zip › Figure_3-source_data_1/Figure_3-source_data_1_Figure_3B_PPARa─.tif]

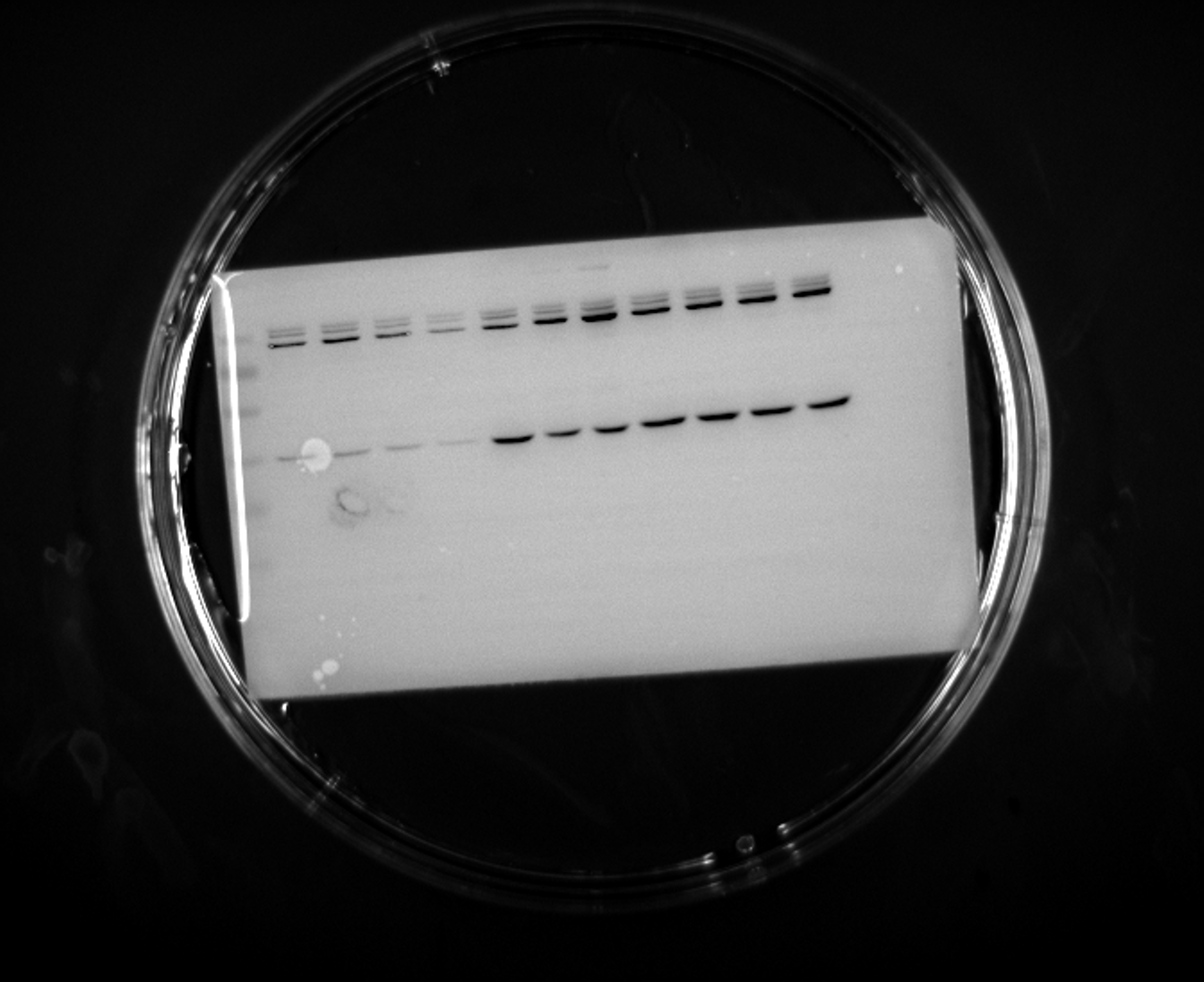

Supplement: Figure 3—source data 1. [file elife-82970-fig3-data1.zip › Figure_3-source_data_1/Figure_3-source_data_1_Figure_3B_PTGIR.Tif]

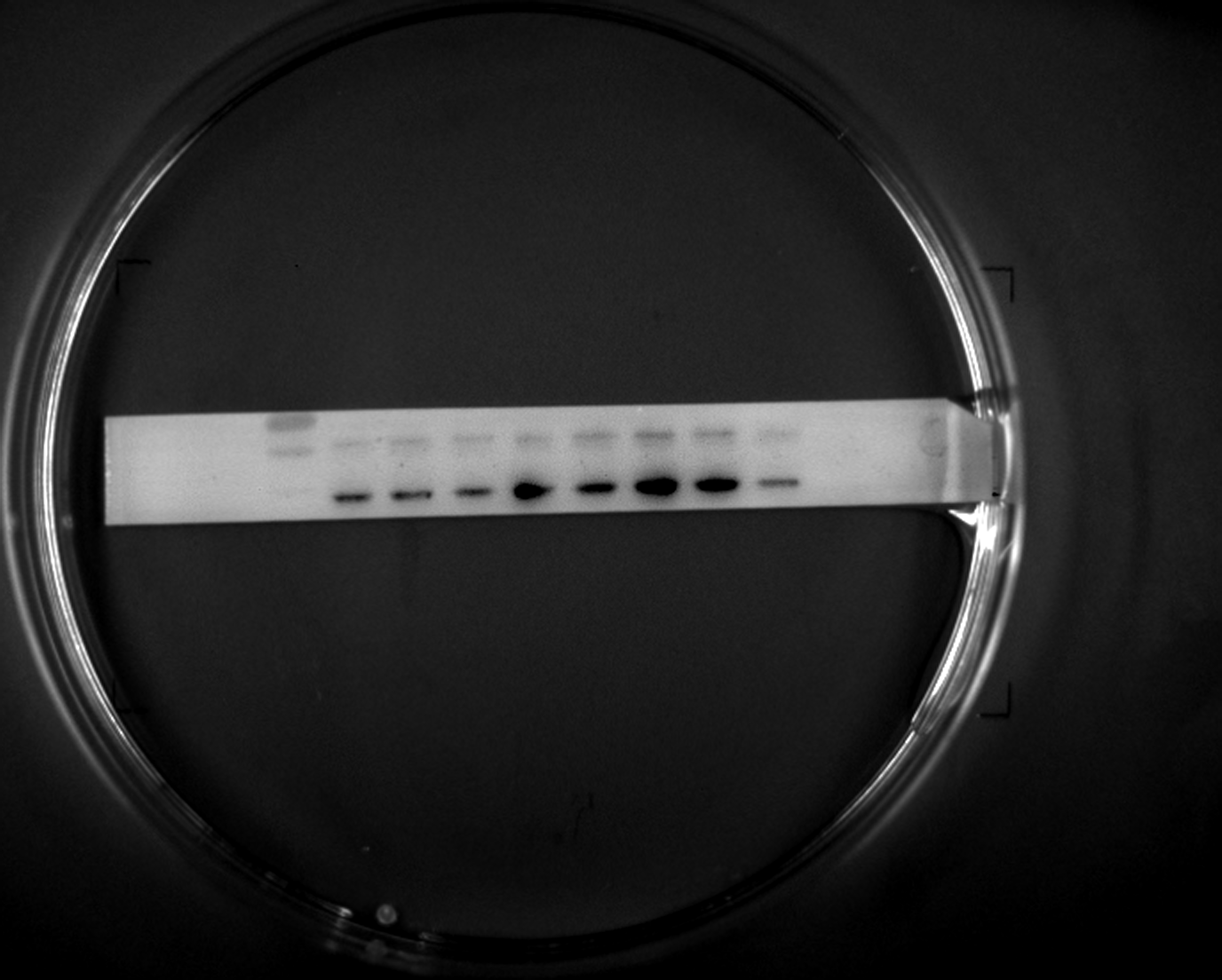

Supplement: Figure 3—source data 1. [file elife-82970-fig3-data1.zip › Figure_3-source_data_1/Figure_3-source_data_1_Figure_3B_SPARC.tif]

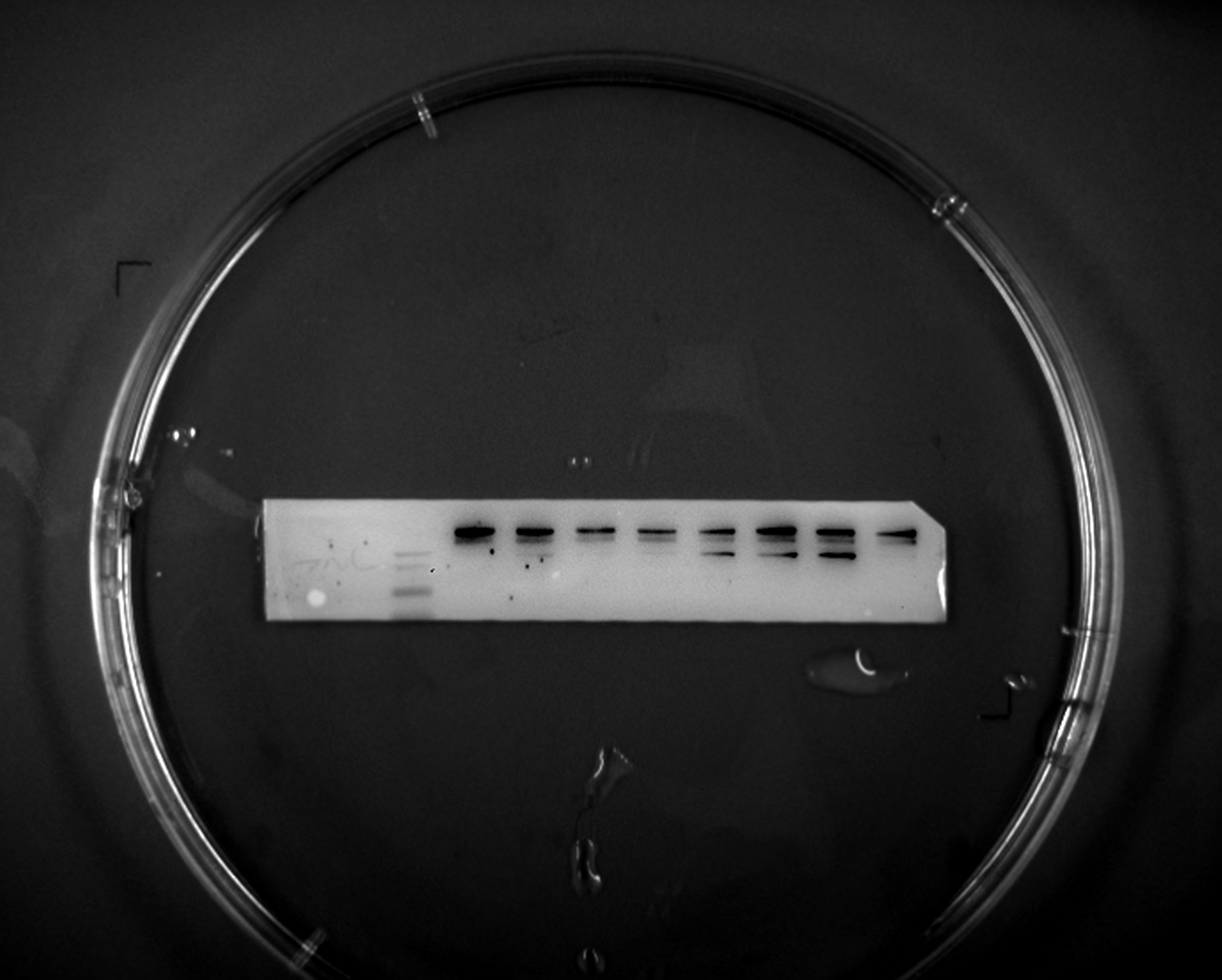

Supplement: Figure 3—source data 1. [file elife-82970-fig3-data1.zip › Figure_3-source_data_1/Figure_3-source_data_1_Figure_3B_TNC.tif]

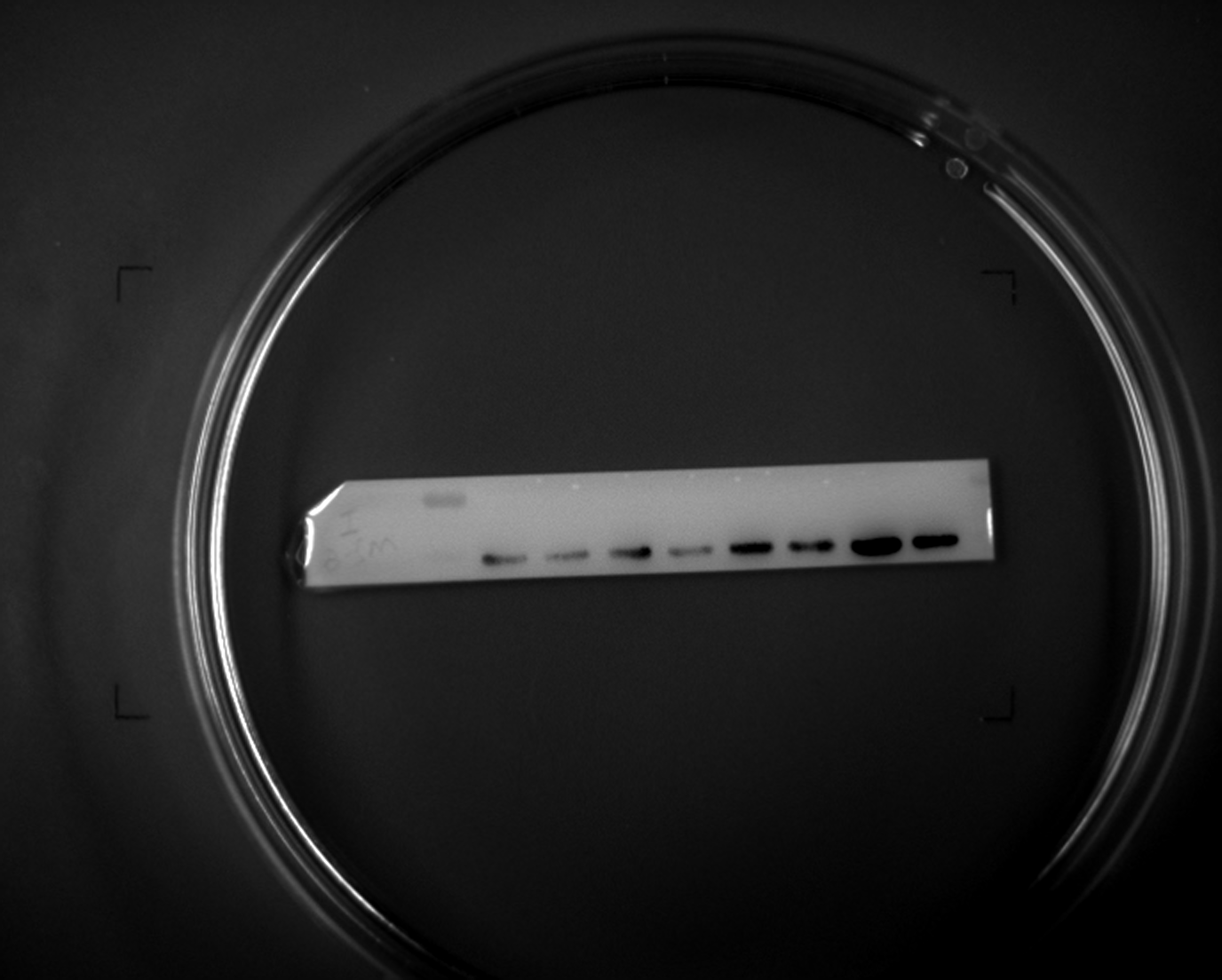

Supplement: Figure 3—source data 1. [file elife-82970-fig3-data1.zip › Figure_3-source_data_1/Figure_3-source_data_1_Figure_3B_a┴-SMA.tif]

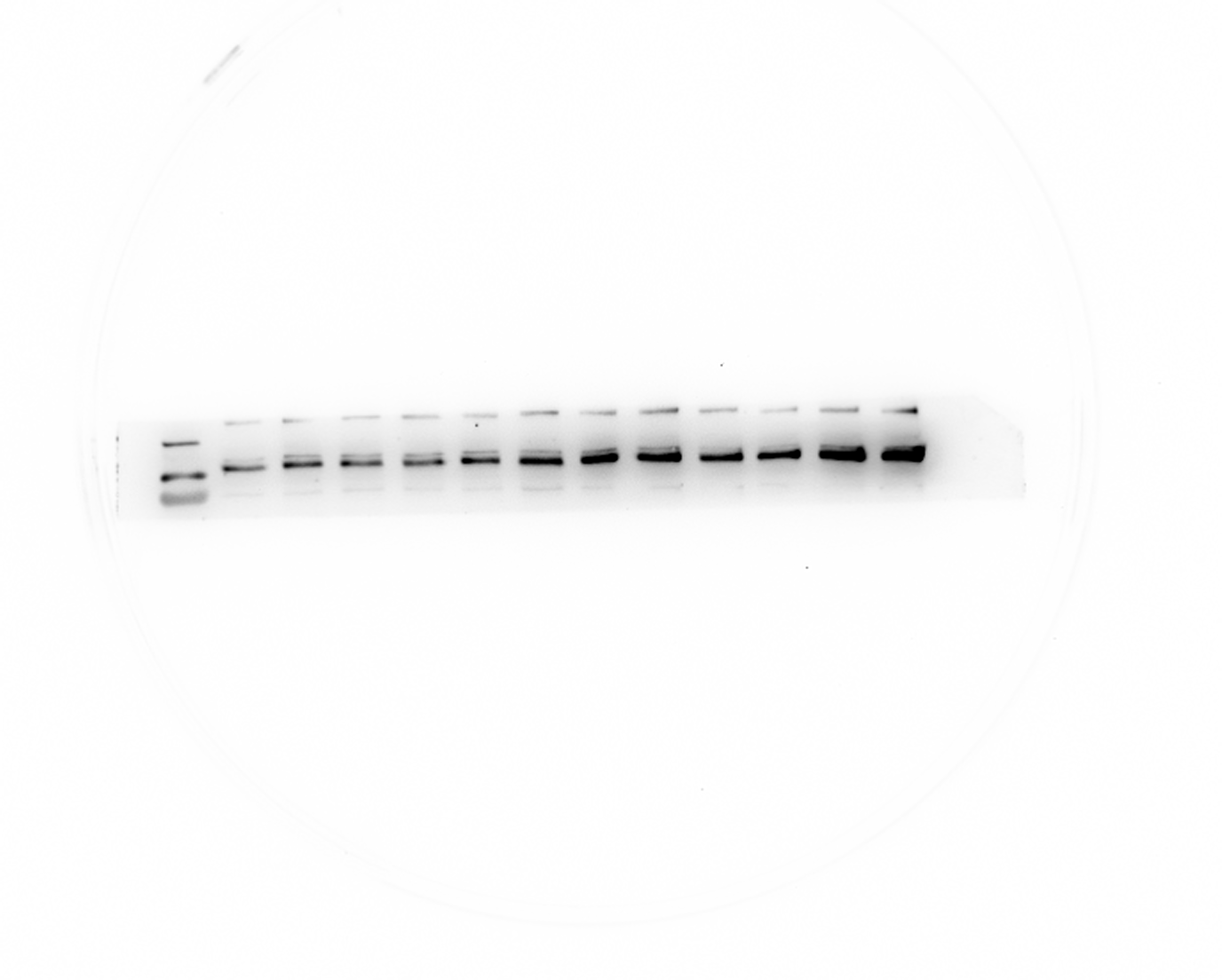

Supplement: Figure 3—source data 1. [file elife-82970-fig3-data1.zip › Figure_3-source_data_1/Figure_3-source_data_1_Figure_3C_SPARC.tif]

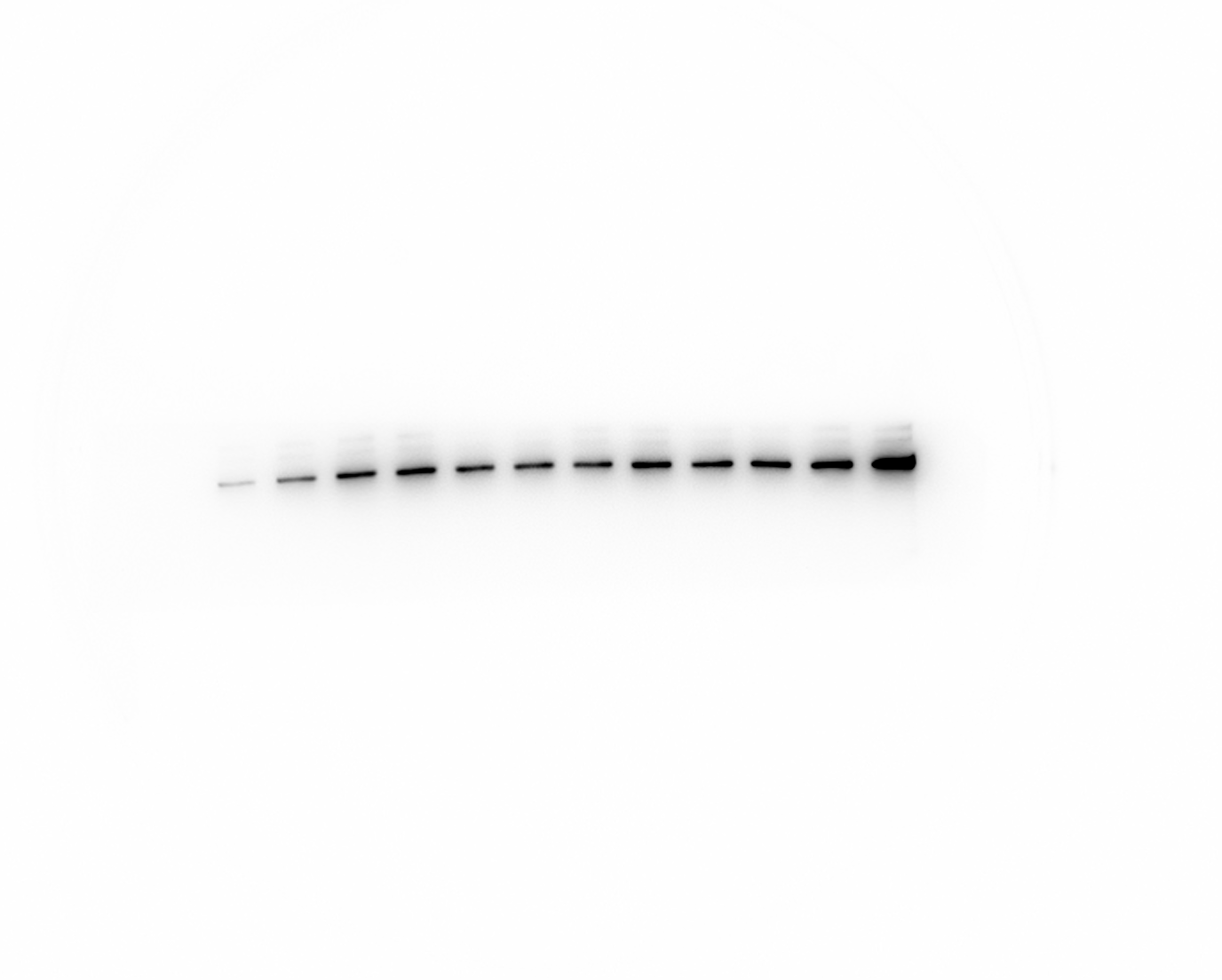

Supplement: Figure 3—source data 1. [file elife-82970-fig3-data1.zip › Figure_3-source_data_1/Figure_3-source_data_1_Figure_3C_TNC.tif]

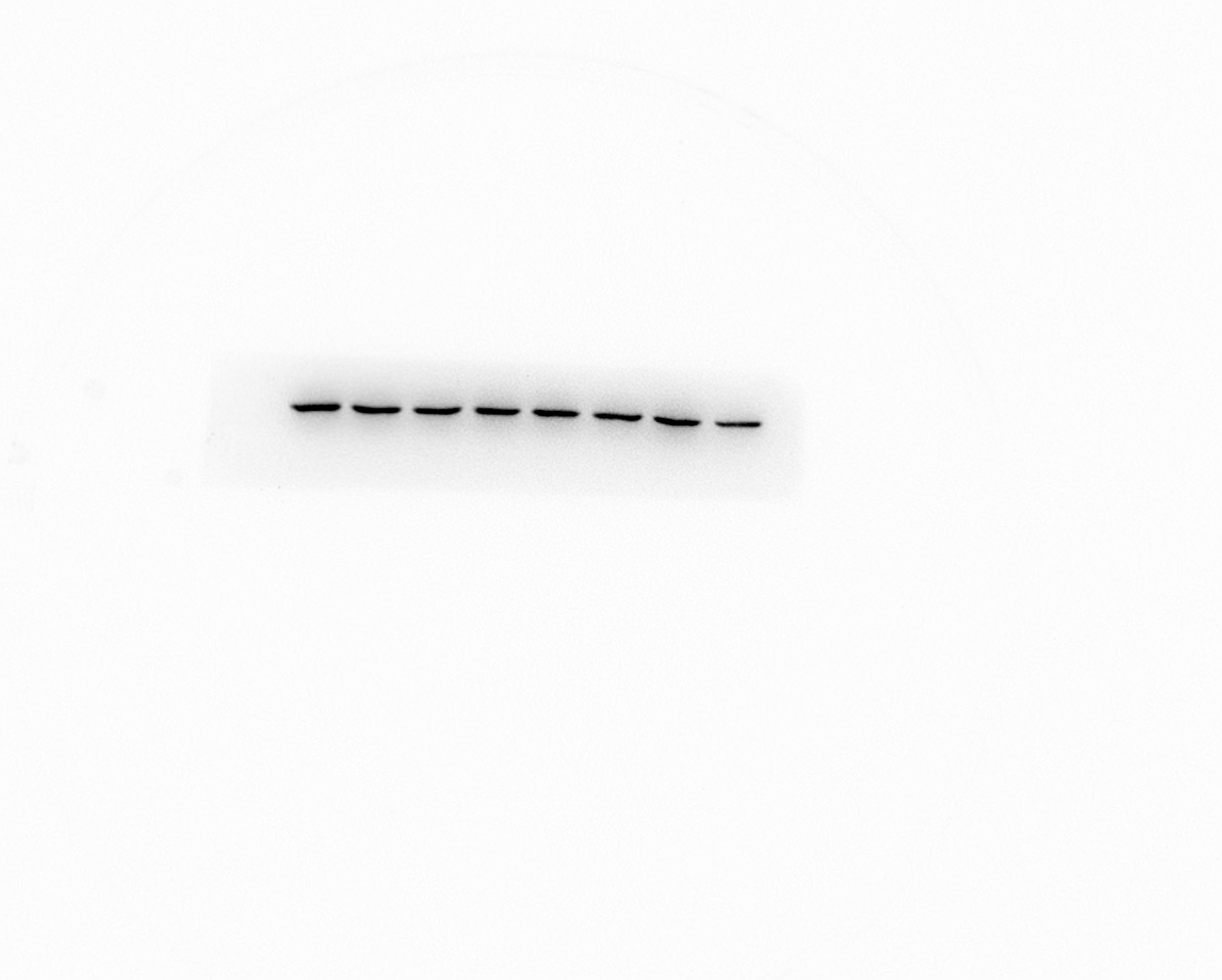

Supplement: Figure 3—source data 1. [file elife-82970-fig3-data1.zip › Figure_3-source_data_1/Figure_3-source_data_1_Figure_3C_TUBULIN.tif]

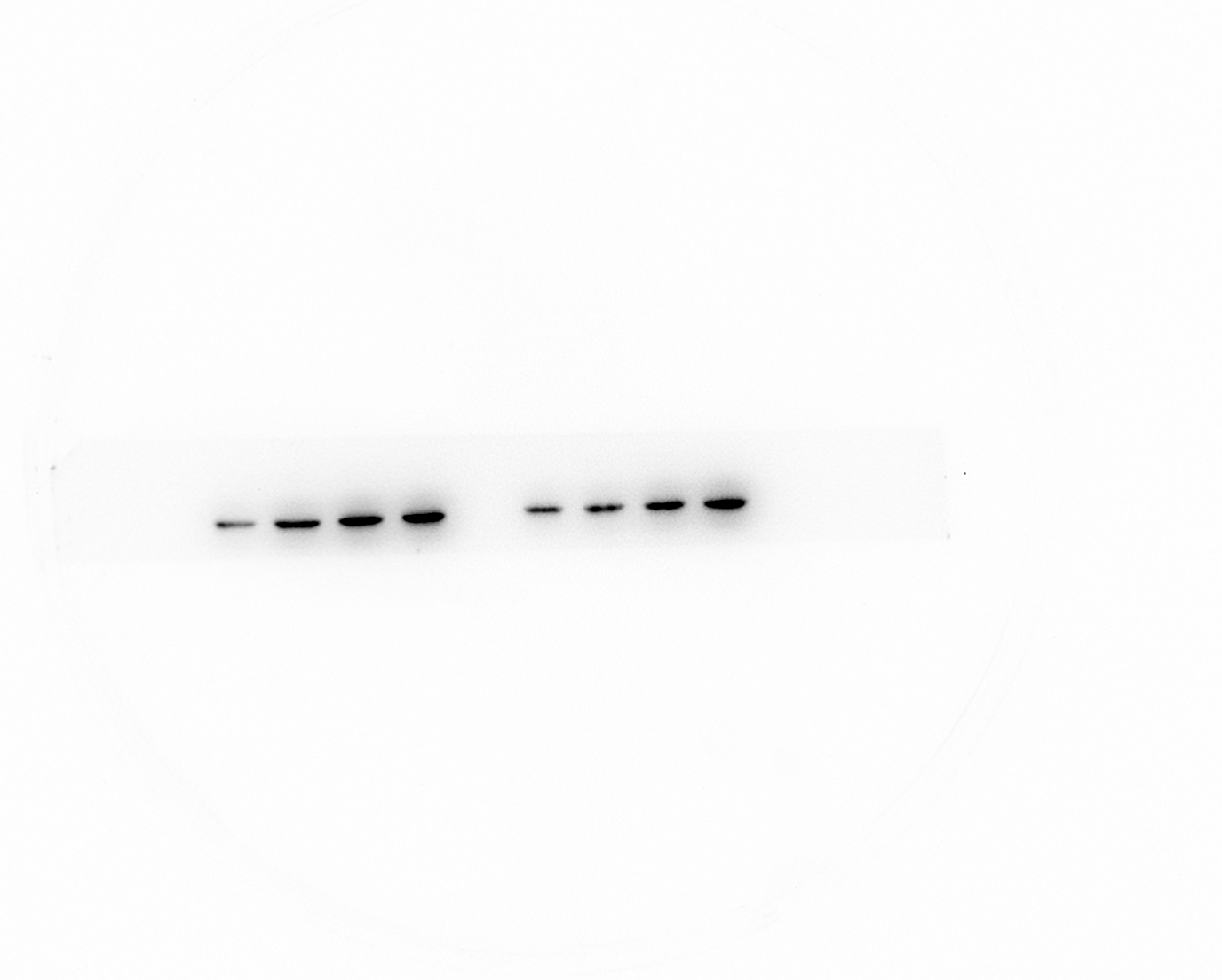

Supplement: Figure 3—source data 1. [file elife-82970-fig3-data1.zip › Figure_3-source_data_1/Figure_3-source_data_1_Figure_3C_a┴-SMA.tif]

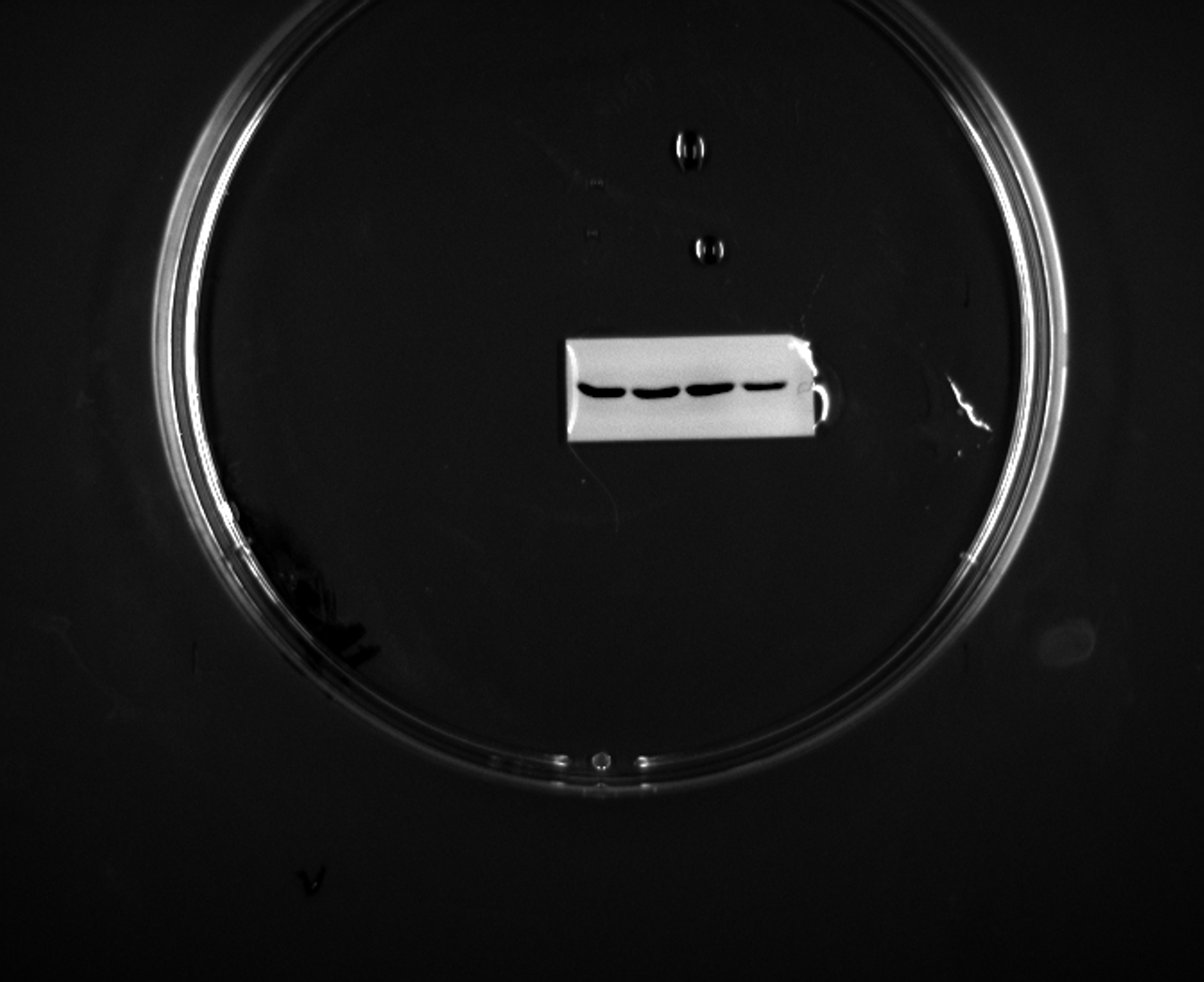

Supplement: Figure 3—source data 1. [file elife-82970-fig3-data1.zip › Figure_3-source_data_1/Figure_3-source_data_1_Figure_3D_GAPDH.Tif]

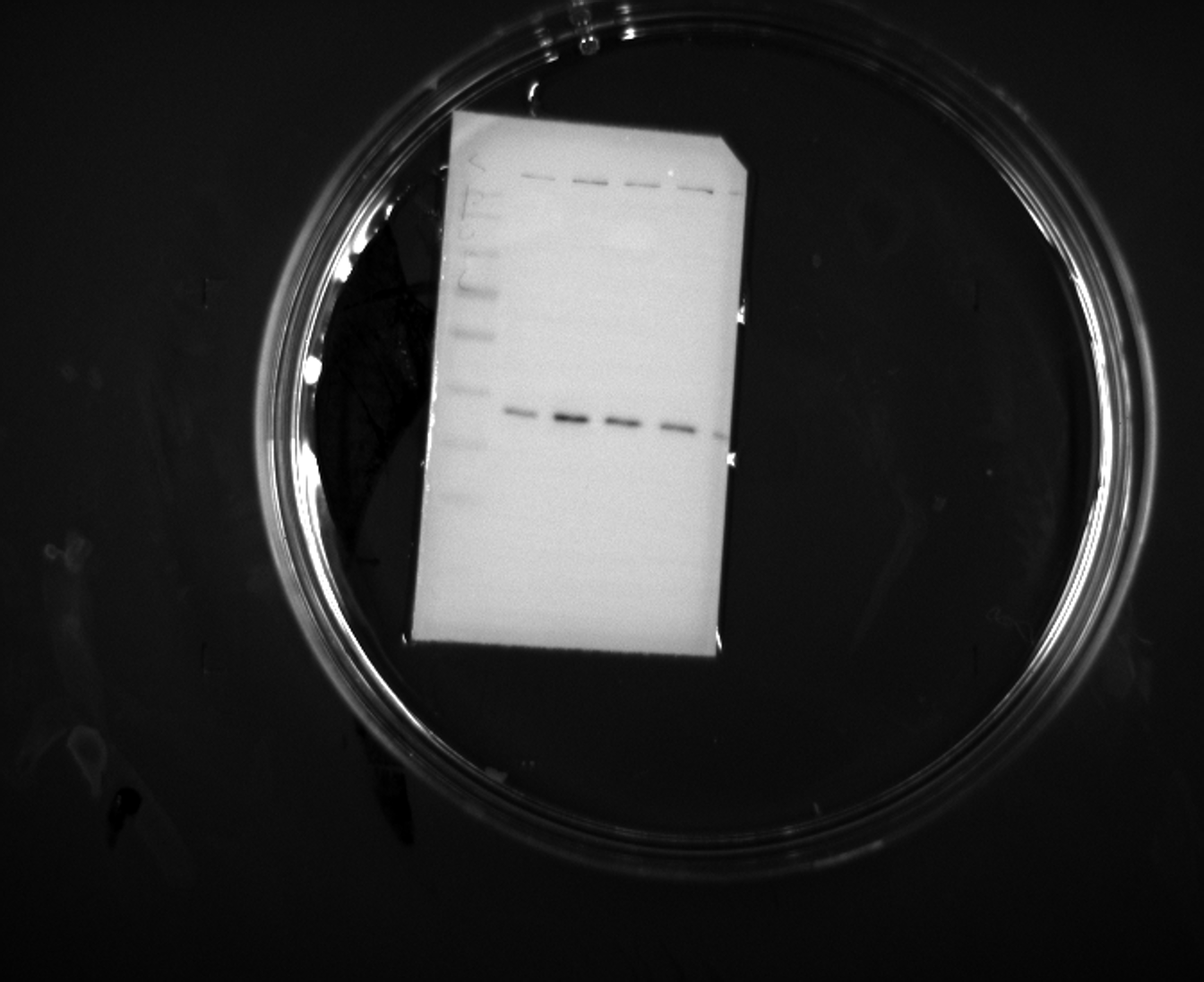

Supplement: Figure 3—source data 1. [file elife-82970-fig3-data1.zip › Figure_3-source_data_1/Figure_3-source_data_1_Figure_3D_SPARC.Tif]

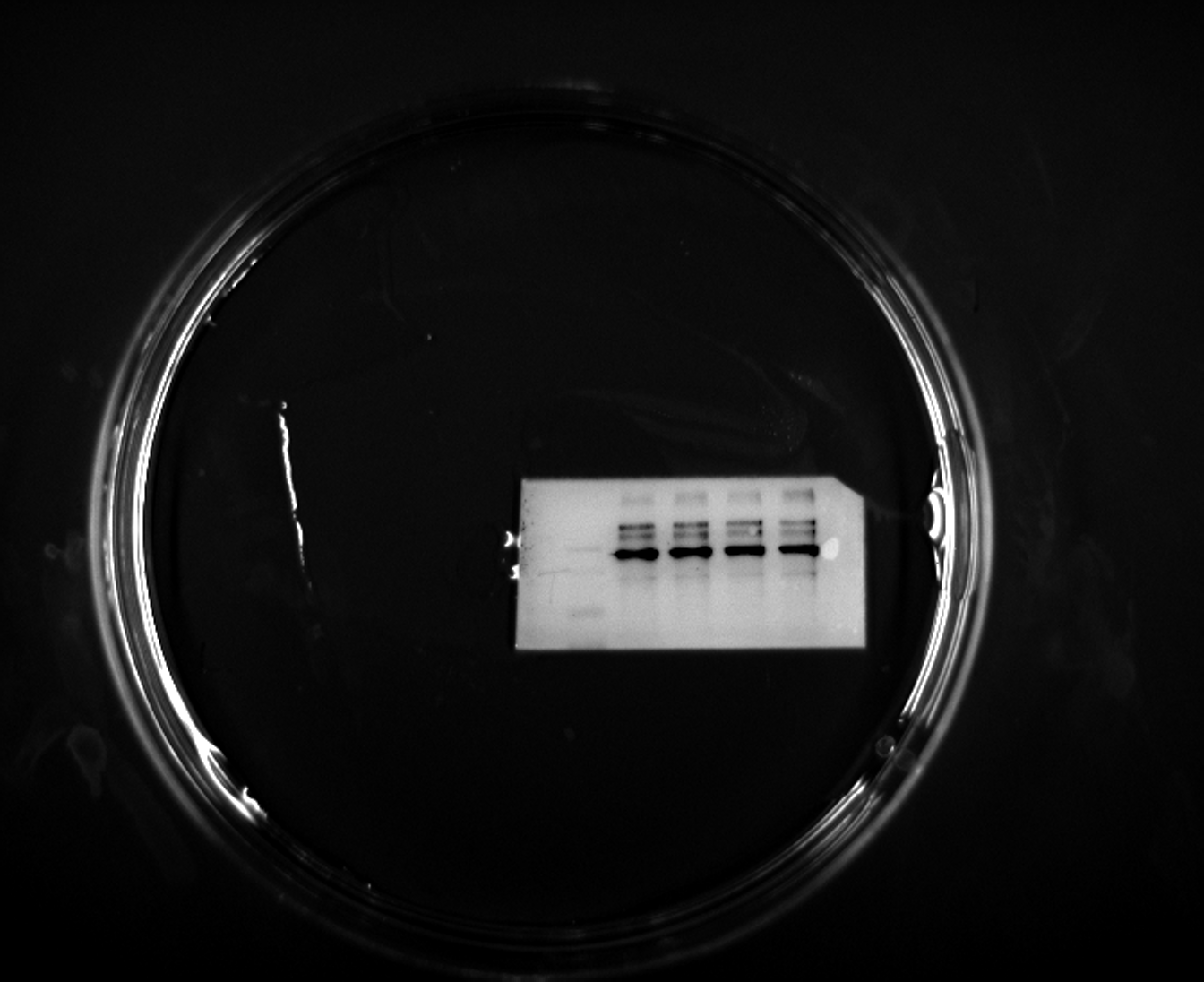

Supplement: Figure 3—source data 1. [file elife-82970-fig3-data1.zip › Figure_3-source_data_1/Figure_3-source_data_1_Figure_3D_TNC.Tif]

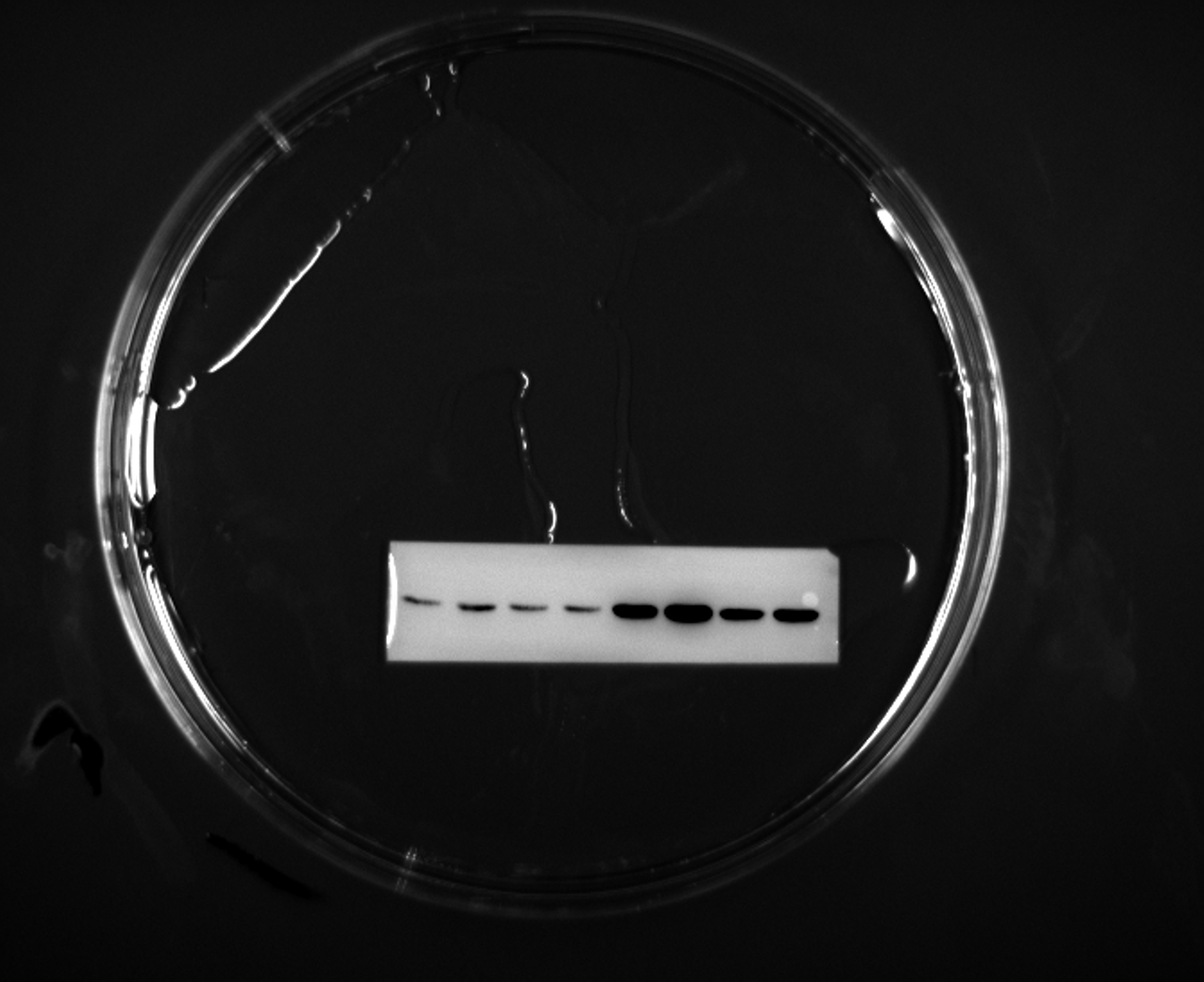

Supplement: Figure 3—source data 1. [file elife-82970-fig3-data1.zip › Figure_3-source_data_1/Figure_3-source_data_1_Figure_3D_a┴-SMA.Tif]

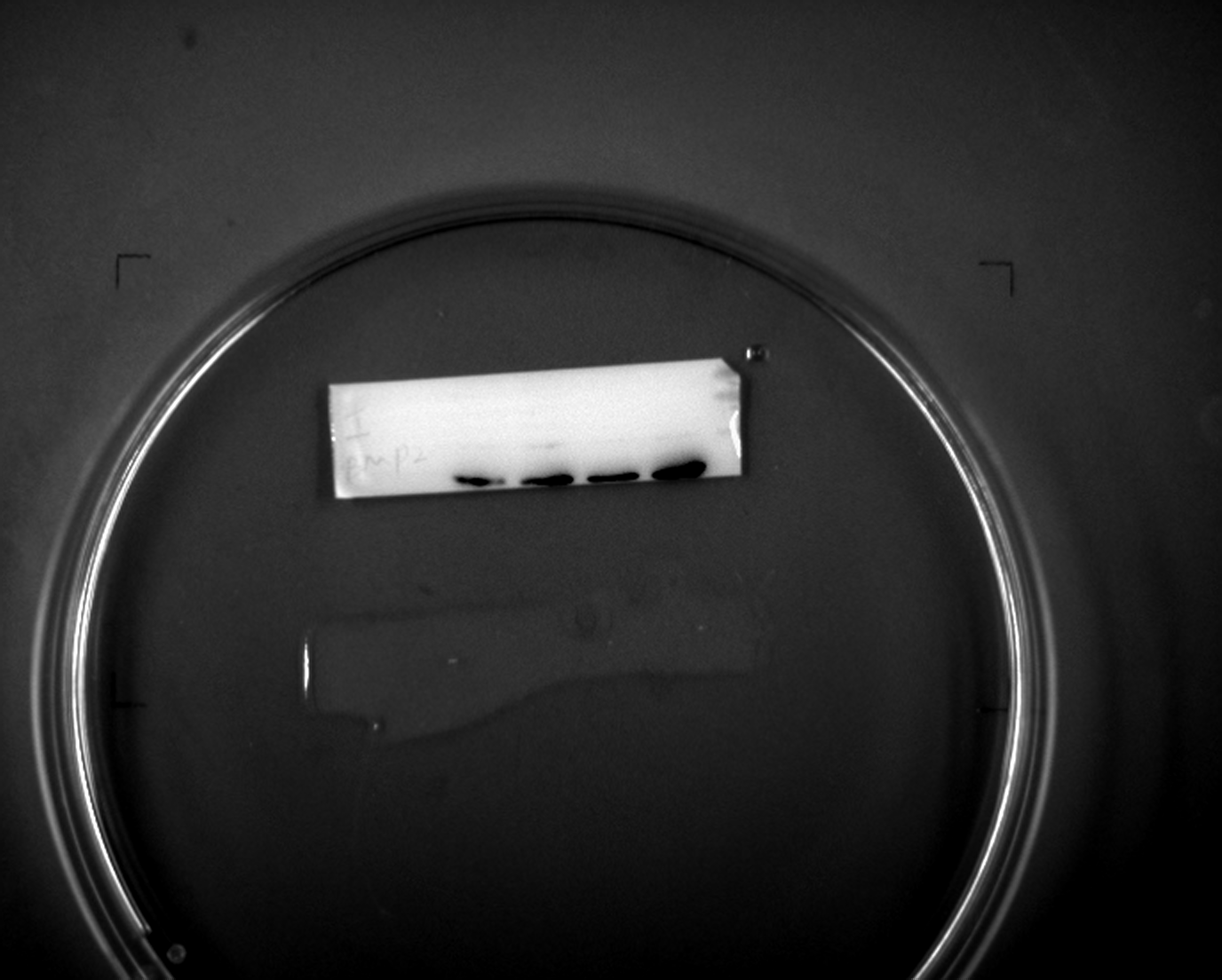

Supplement: Figure 3—source data 1. [file elife-82970-fig3-data1.zip › Figure_3-source_data_1/Figure_3-source_data_1_Figure_3E_BMP2.tif]

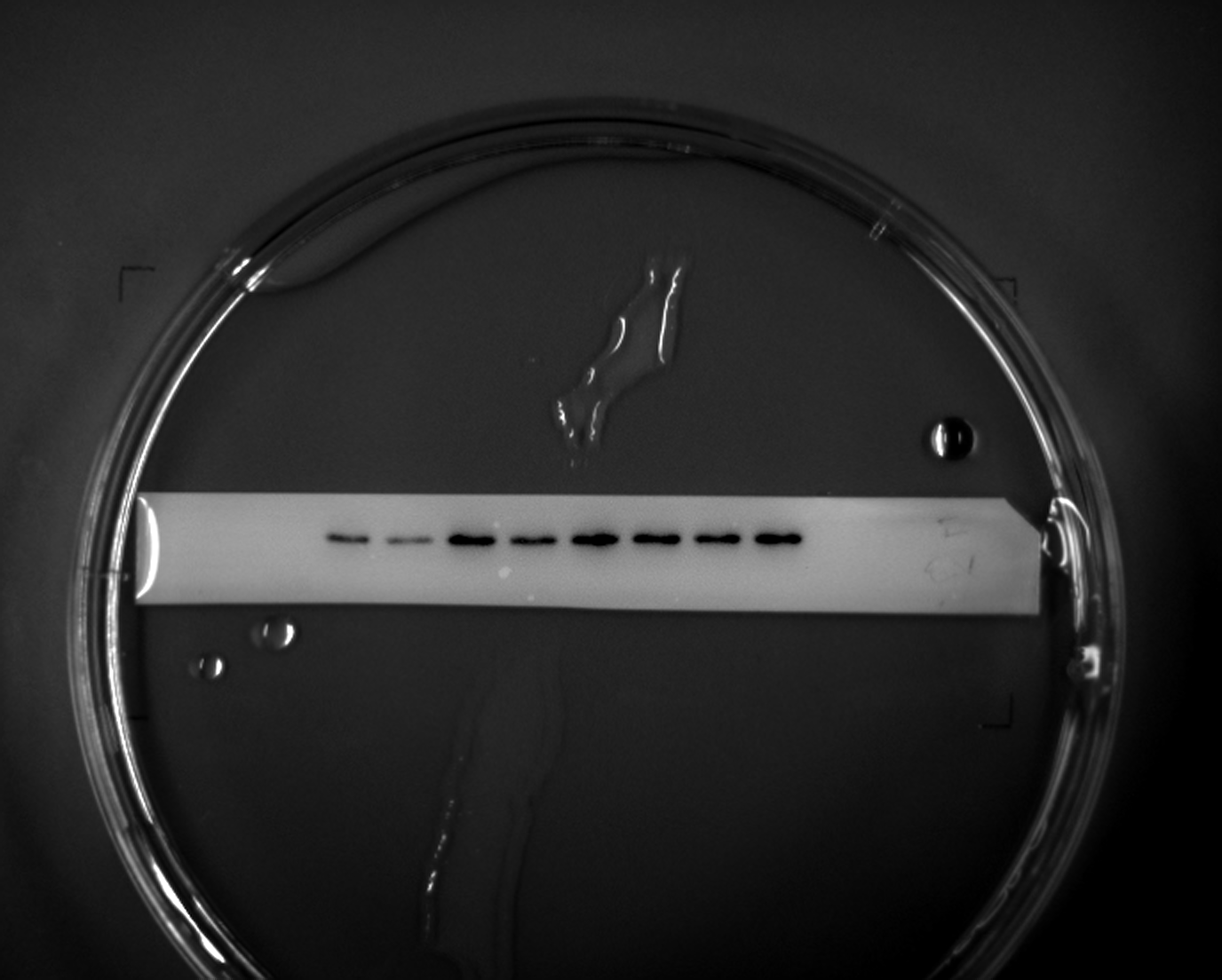

Supplement: Figure 3—source data 1. [file elife-82970-fig3-data1.zip › Figure_3-source_data_1/Figure_3-source_data_1_Figure_3E_CYCLIN D3.tif]

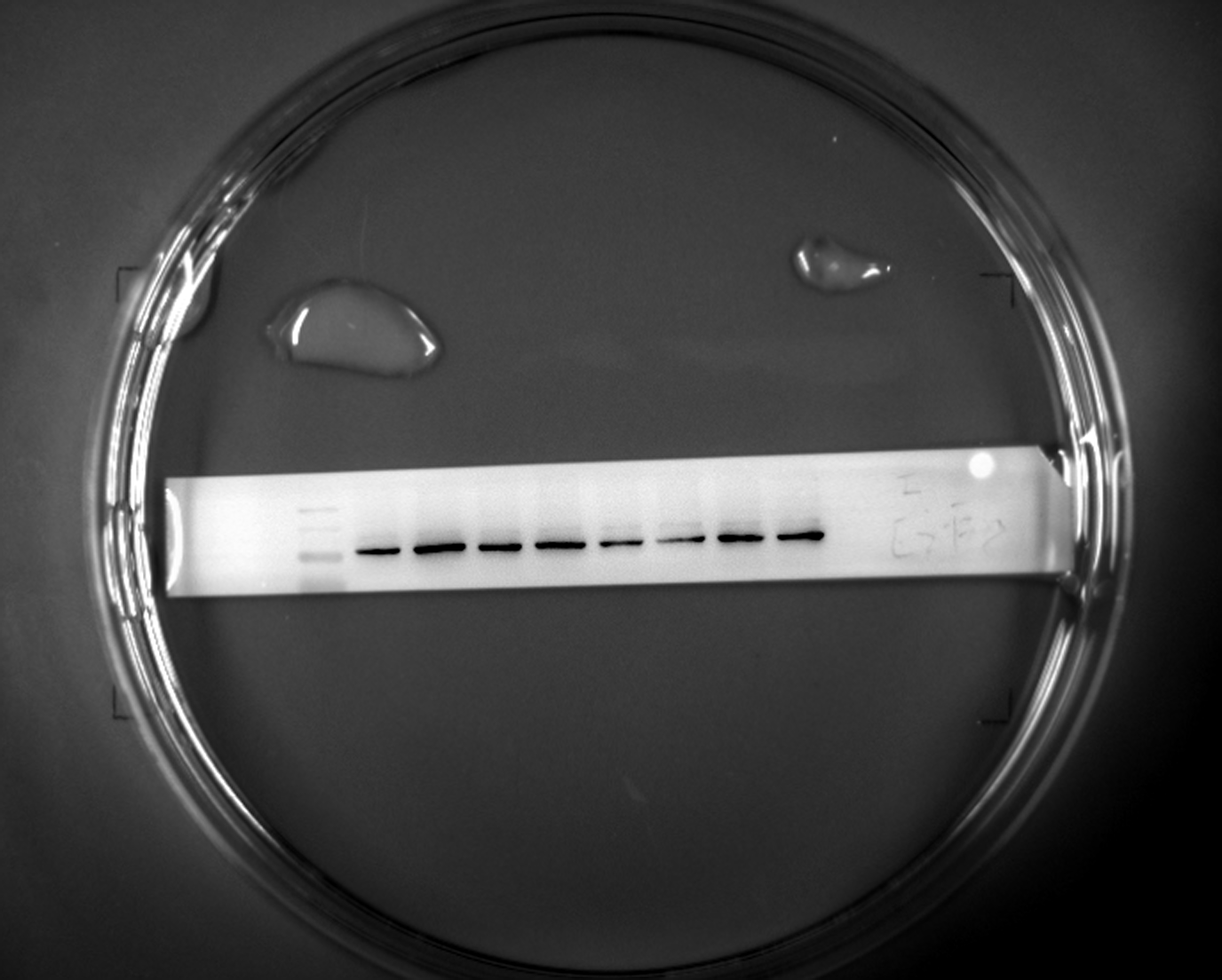

Supplement: Figure 3—source data 1. [file elife-82970-fig3-data1.zip › Figure_3-source_data_1/Figure_3-source_data_1_Figure_3E_E2F8.tif]

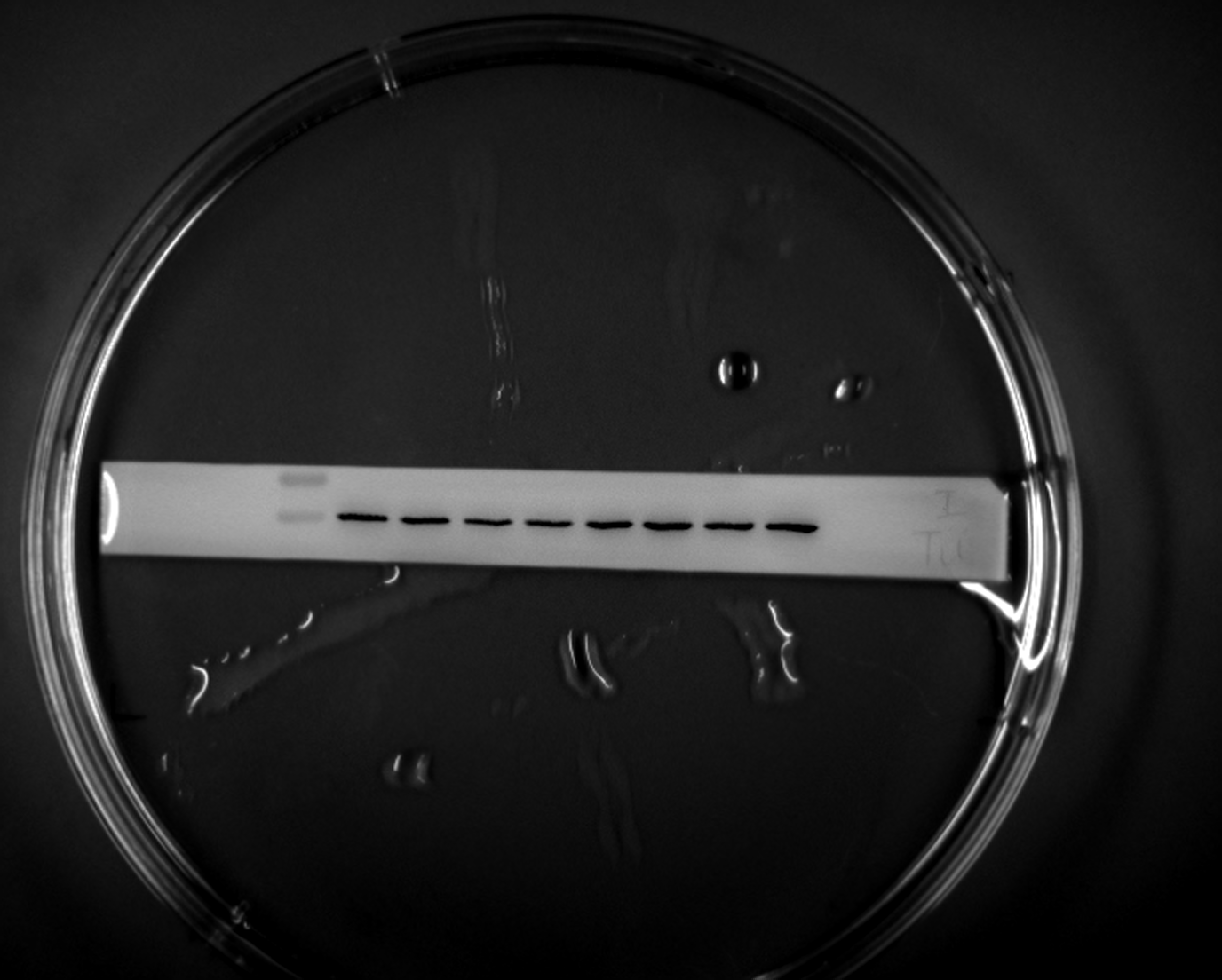

Supplement: Figure 3—source data 1. [file elife-82970-fig3-data1.zip › Figure_3-source_data_1/Figure_3-source_data_1_Figure_3E_TUBULIN.tif]

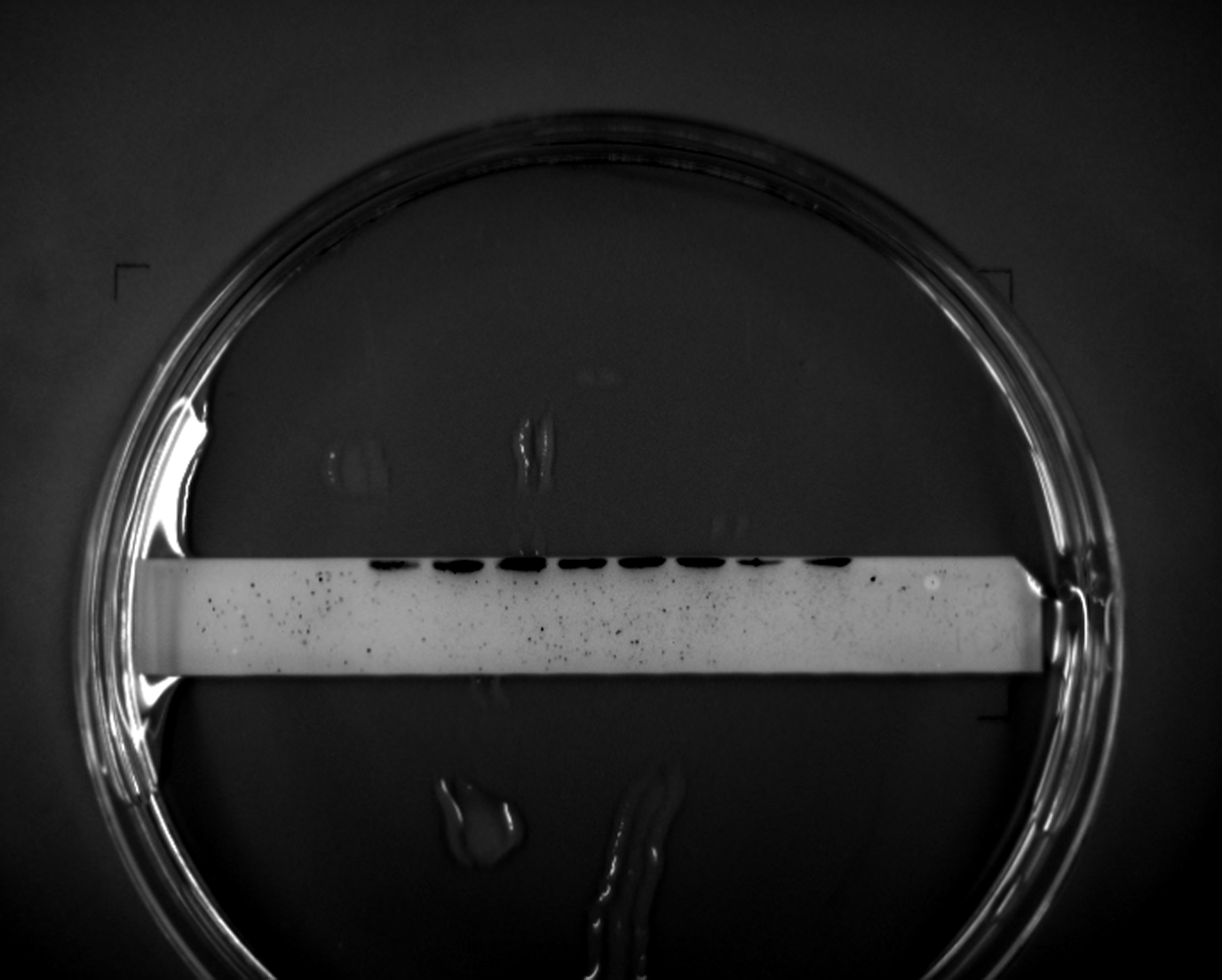

Supplement: Figure 3—source data 1. [file elife-82970-fig3-data1.zip › Figure_3-source_data_1/Figure_3-source_data_1_Figure_3E_WNT4.tif]

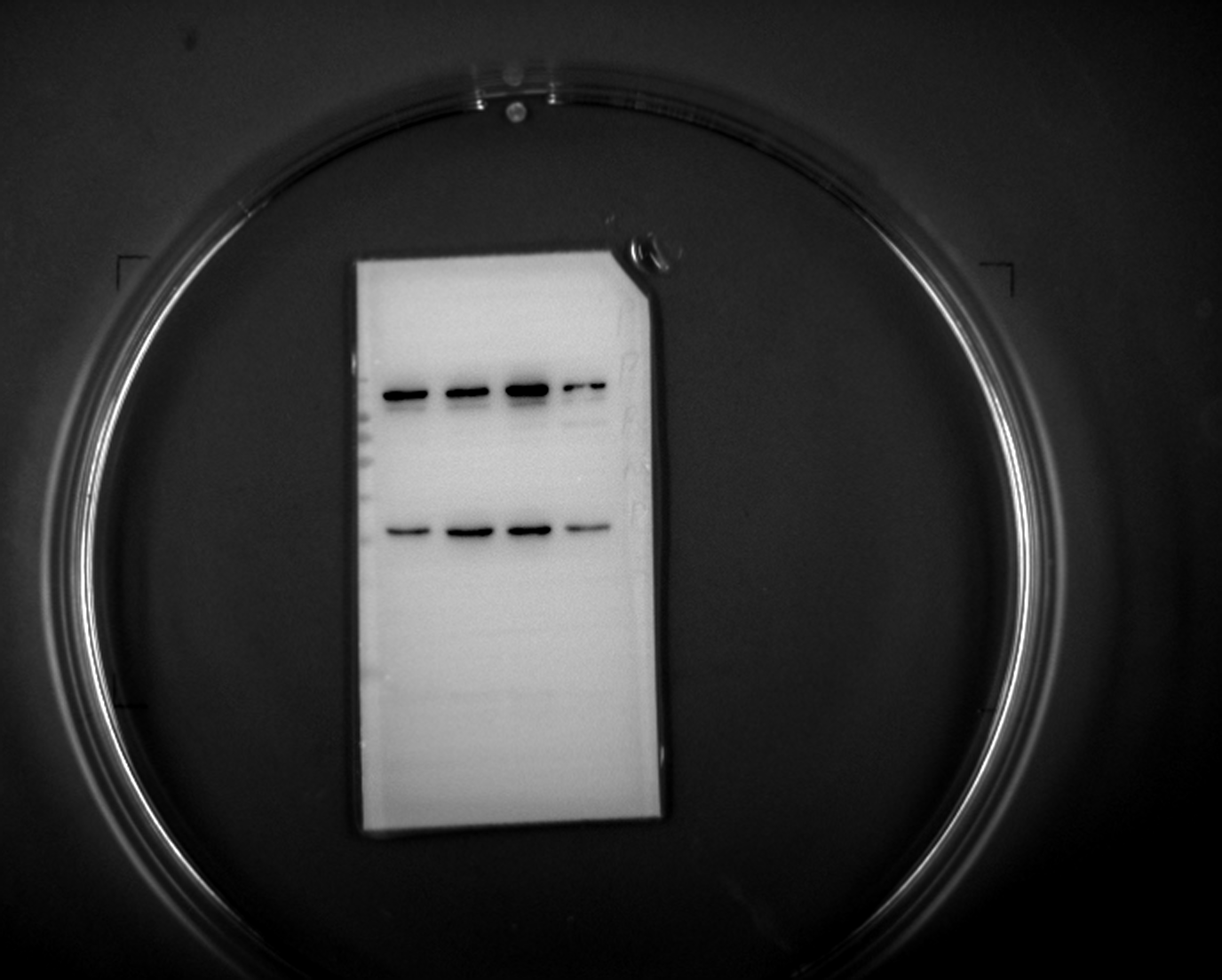

Supplement: Figure 3—source data 1. [file elife-82970-fig3-data1.zip › Figure_3-source_data_1/Figure_3-source_data_1_Figure_3F_BMP2.tif]

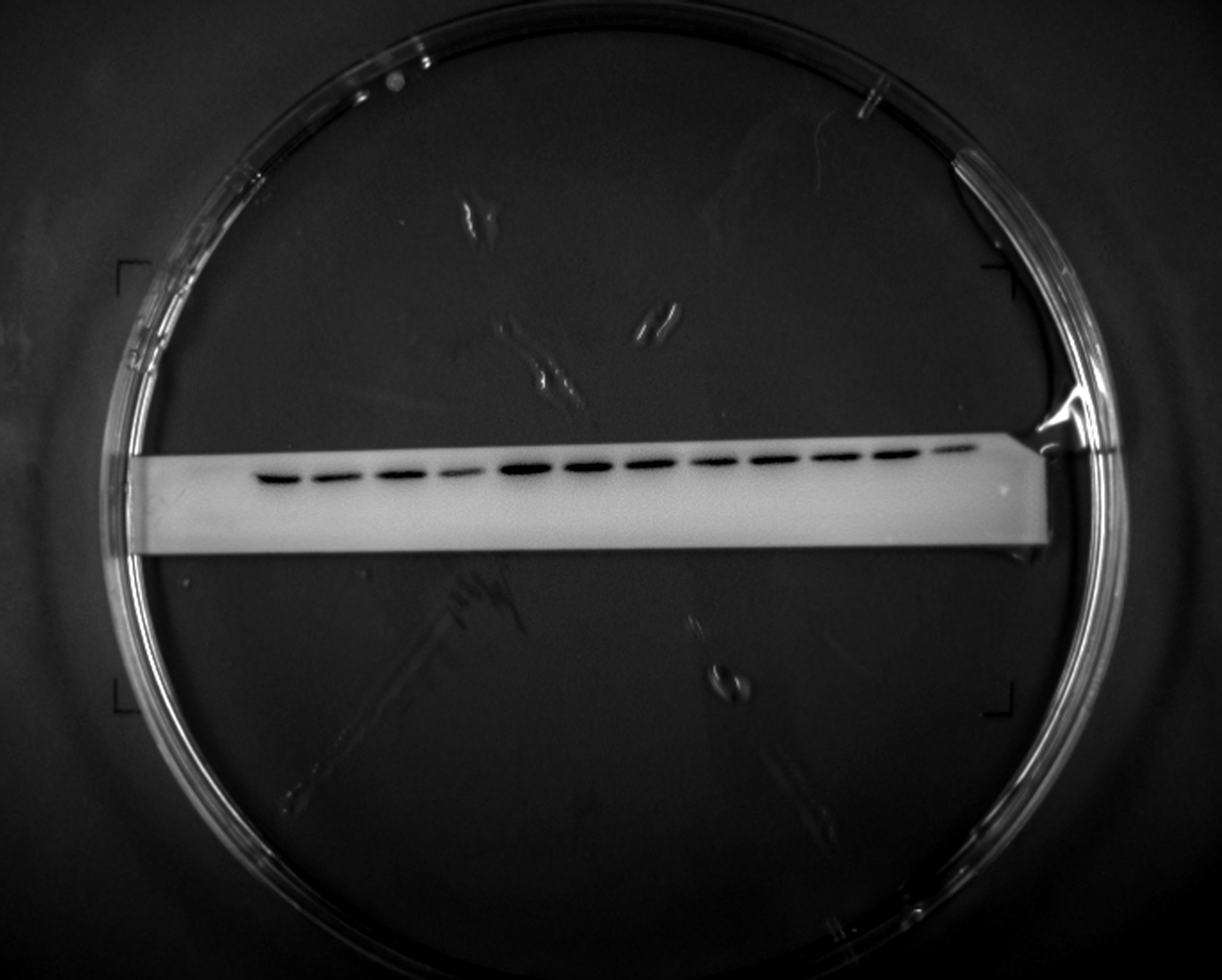

Supplement: Figure 3—source data 1. [file elife-82970-fig3-data1.zip › Figure_3-source_data_1/Figure_3-source_data_1_Figure_3F_CYCLIN D3.tif]

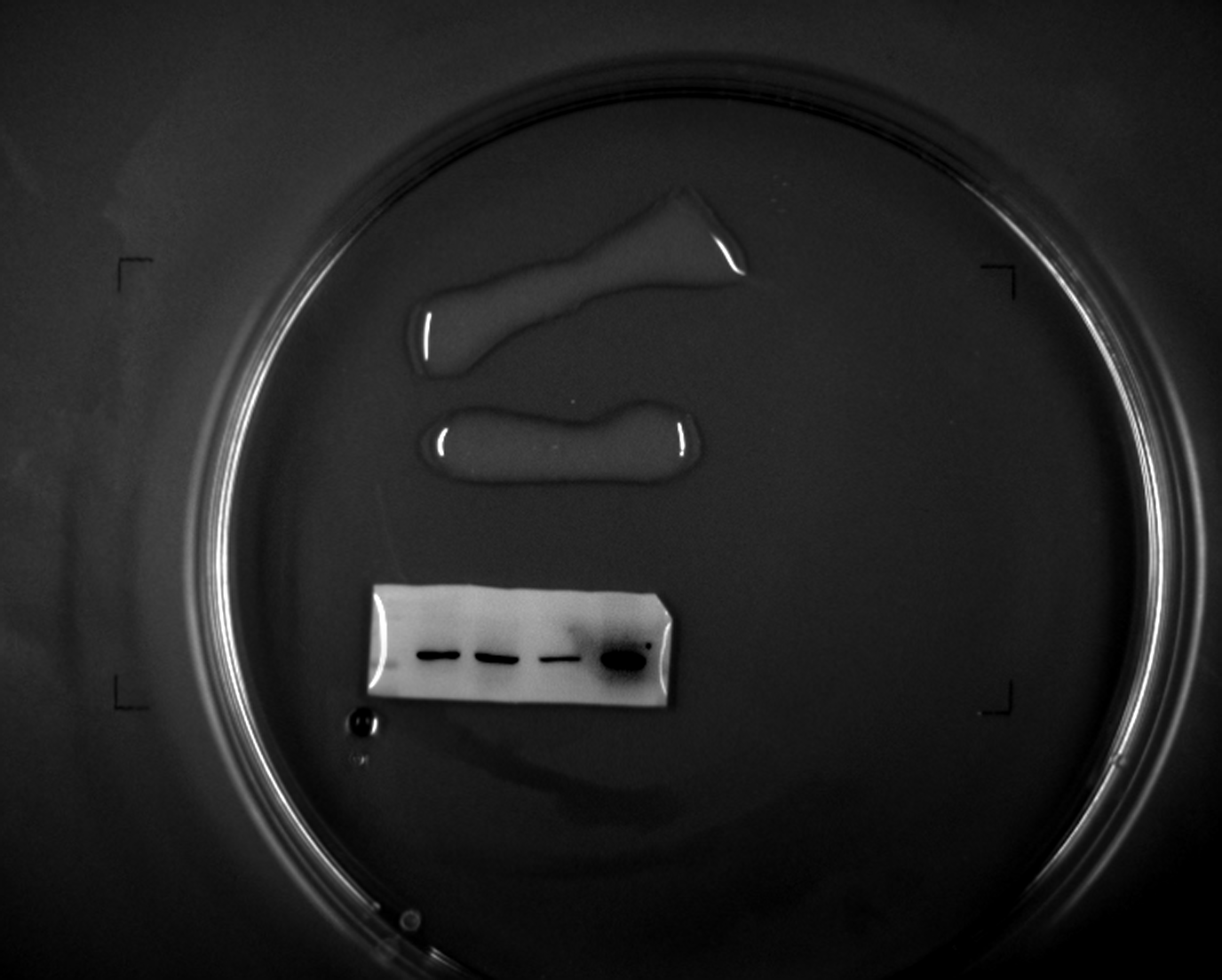

Supplement: Figure 3—source data 1. [file elife-82970-fig3-data1.zip › Figure_3-source_data_1/Figure_3-source_data_1_Figure_3F_E2F8.tif]

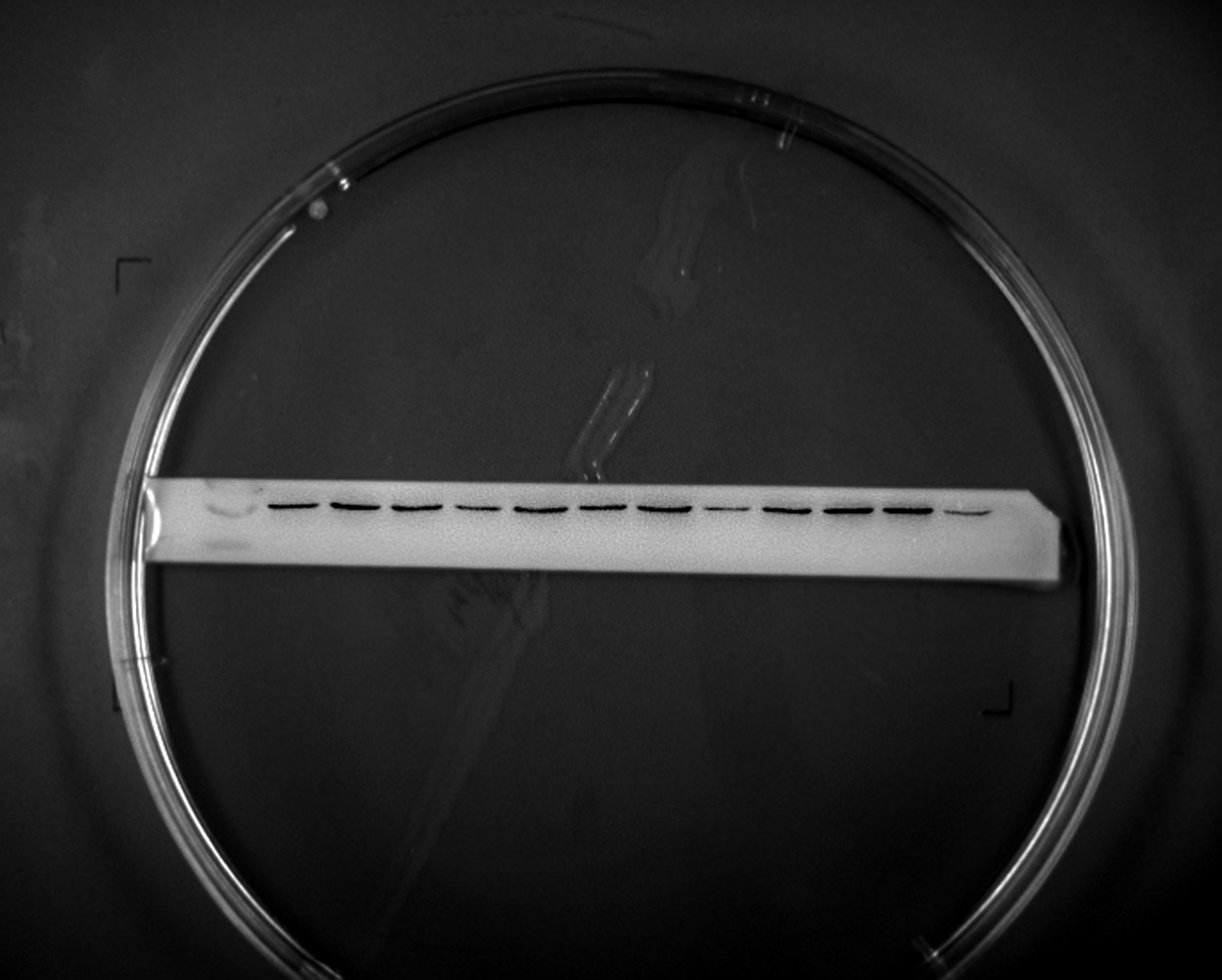

Supplement: Figure 3—source data 1. [file elife-82970-fig3-data1.zip › Figure_3-source_data_1/Figure_3-source_data_1_Figure_3F_TUBULIN.tif]

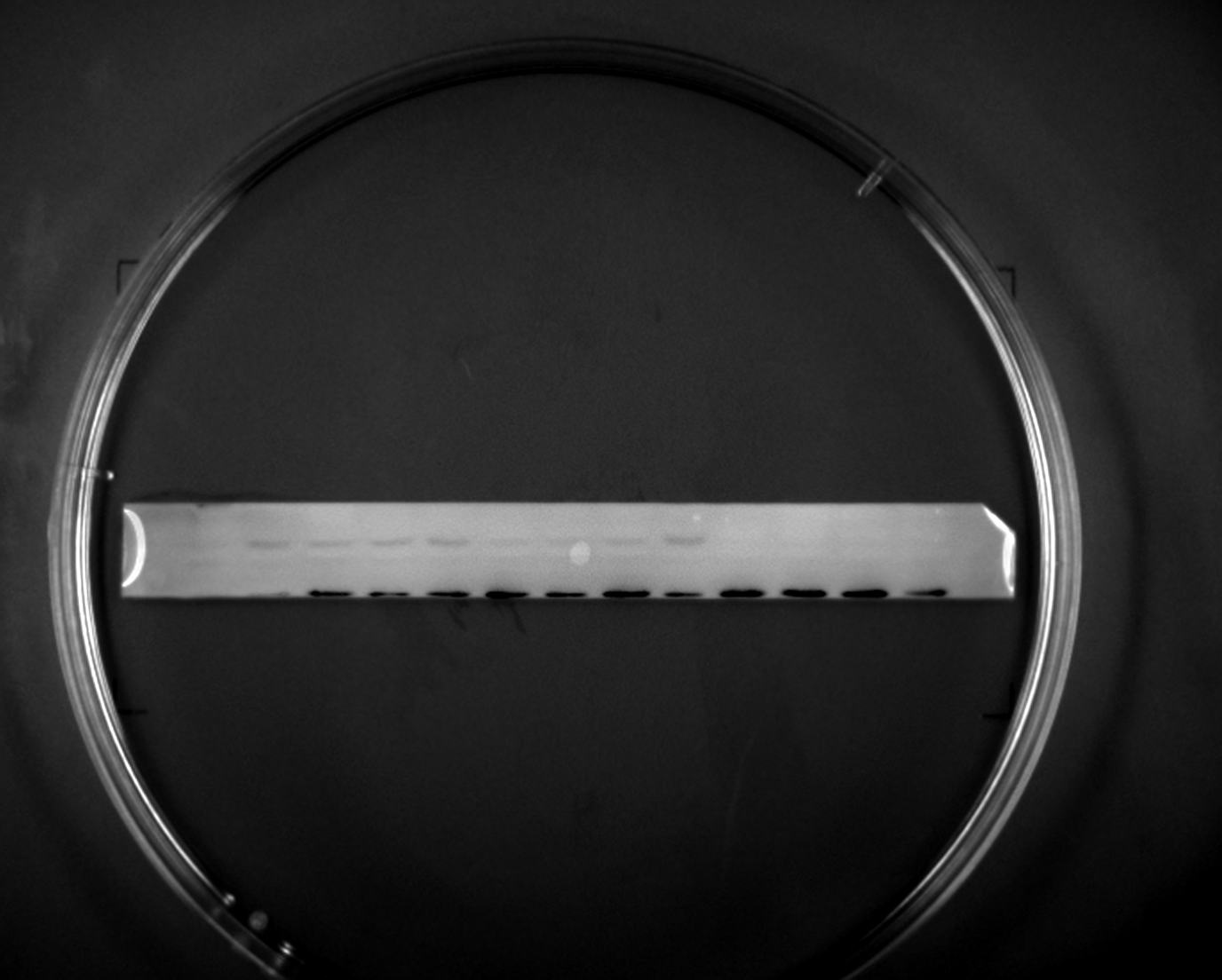

Supplement: Figure 3—source data 1. [file elife-82970-fig3-data1.zip › Figure_3-source_data_1/Figure_3-source_data_1_Figure_3F_WNT4.tif]

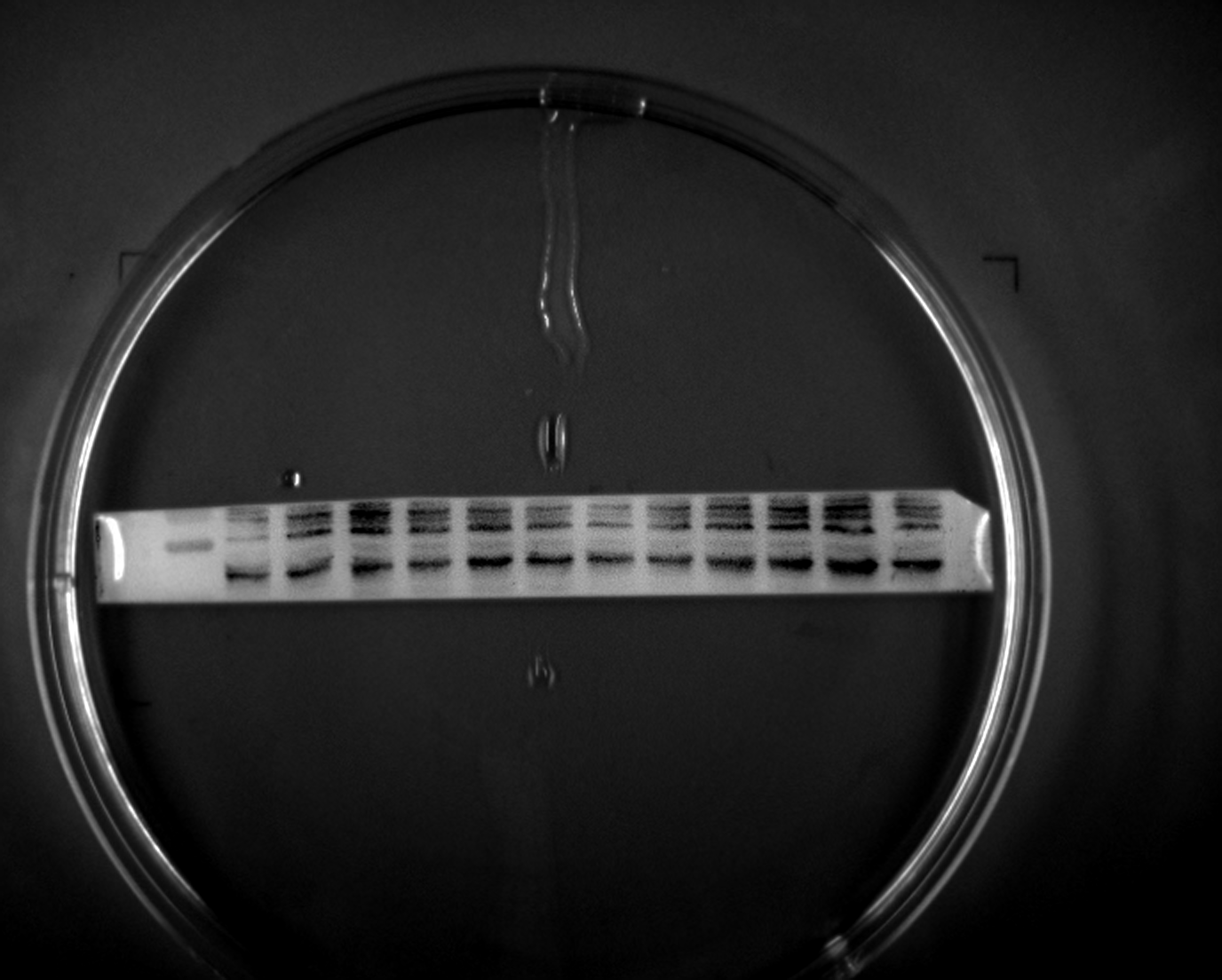

Supplement: Figure 3—source data 1. [file elife-82970-fig3-data1.zip › Figure_3-source_data_1/Figure_3-source_data_1_Figure_3G_ACTIVIN A.tif]

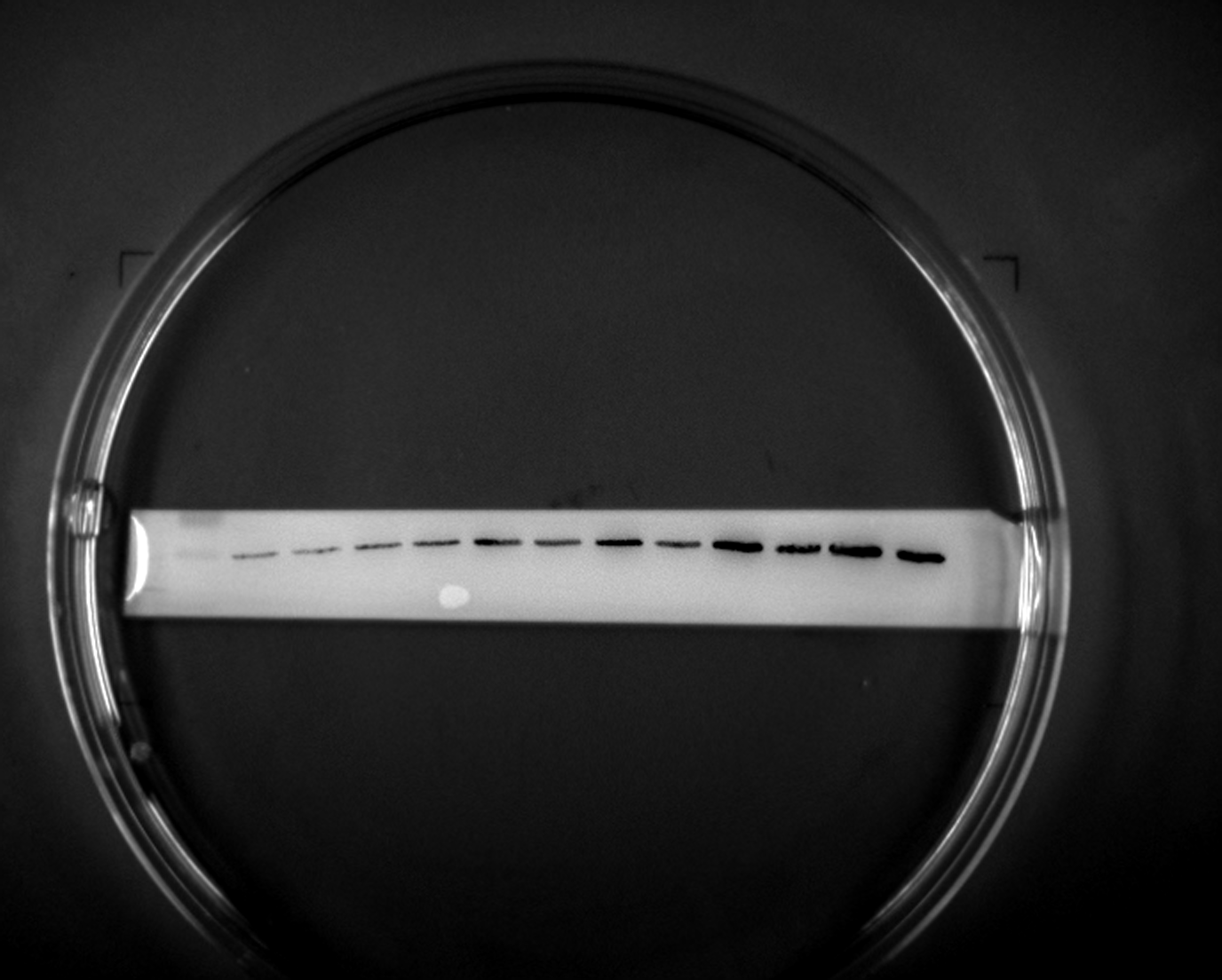

Supplement: Figure 3—source data 1. [file elife-82970-fig3-data1.zip › Figure_3-source_data_1/Figure_3-source_data_1_Figure_3G_TUBULIN.tif]

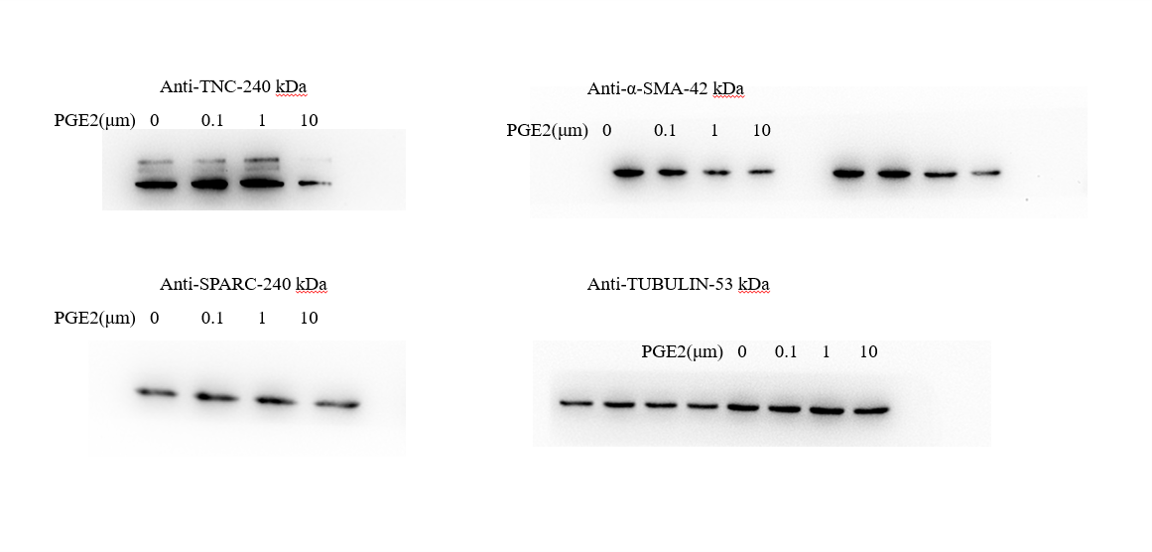

Supplement: Figure 3—source data 2. [file elife-82970-fig3-data2.zip › Figure_3-source_data_2/Figure_3-source_data_2-3A.png]

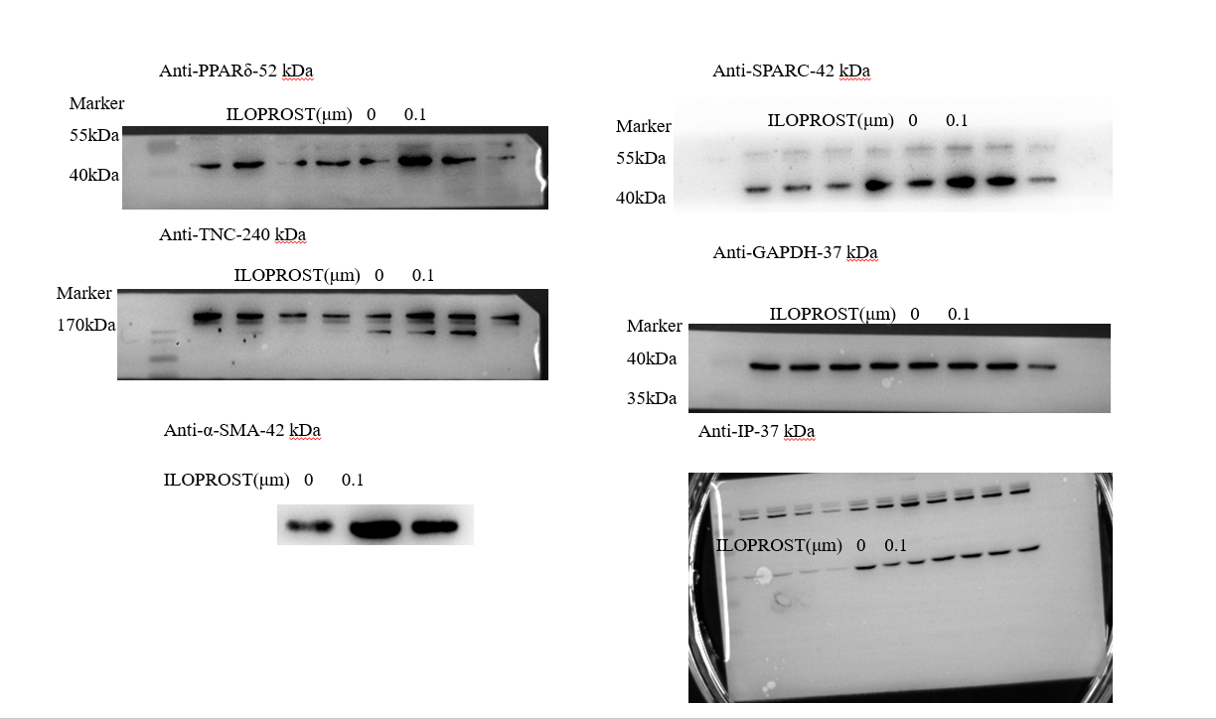

Supplement: Figure 3—source data 2. [file elife-82970-fig3-data2.zip › Figure_3-source_data_2/Figure_3-source_data_2-3B.png]

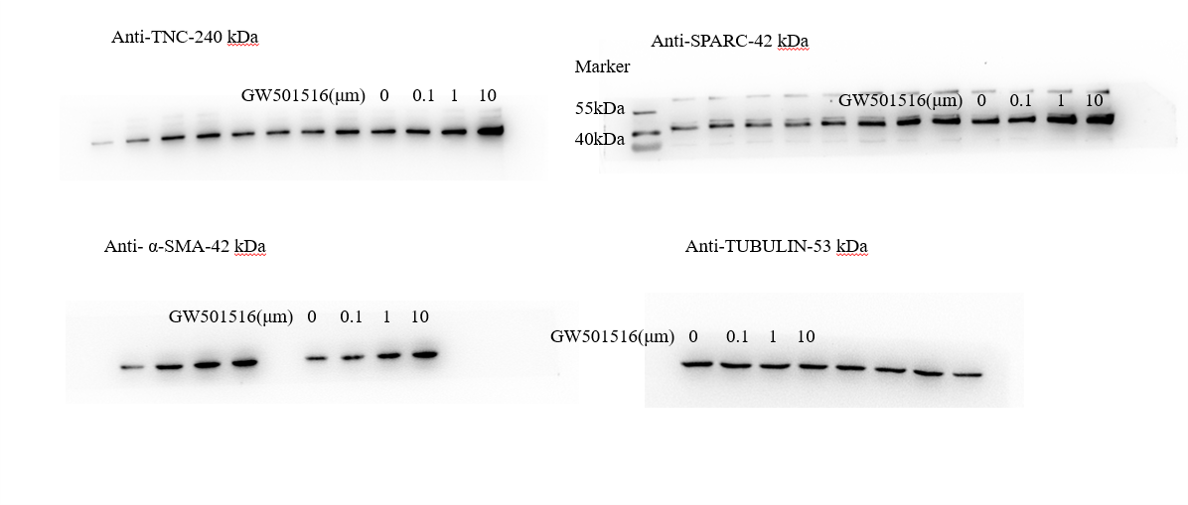

Supplement: Figure 3—source data 2. [file elife-82970-fig3-data2.zip › Figure_3-source_data_2/Figure_3-source_data_2-3C.png]

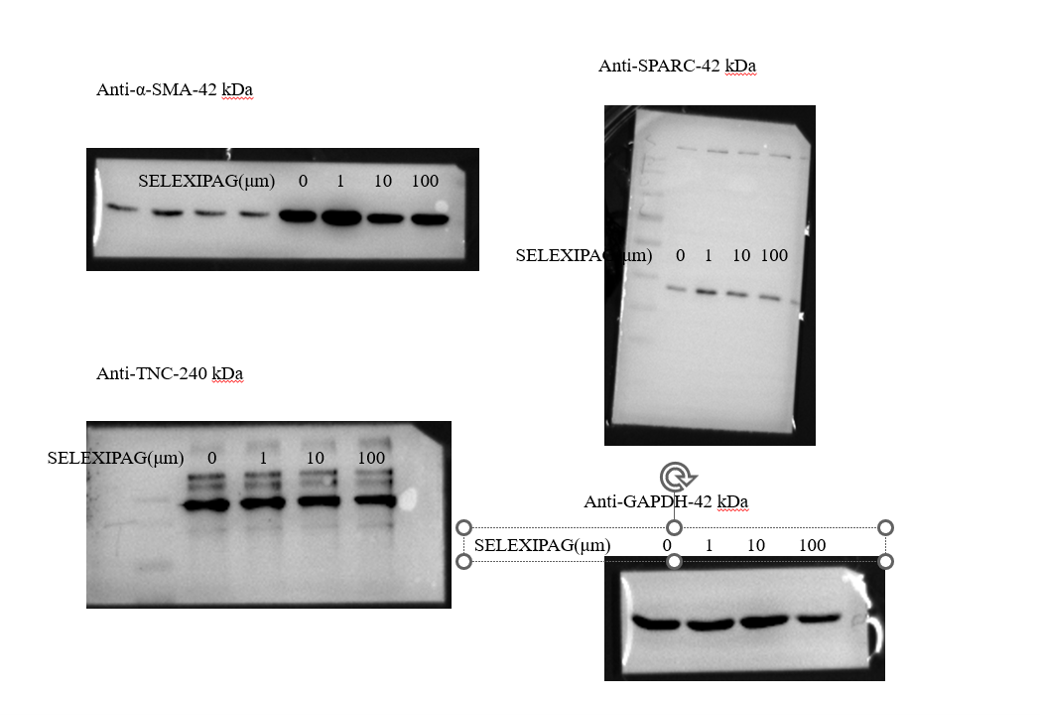

Supplement: Figure 3—source data 2. [file elife-82970-fig3-data2.zip › Figure_3-source_data_2/Figure_3-source_data_2-3D.png]

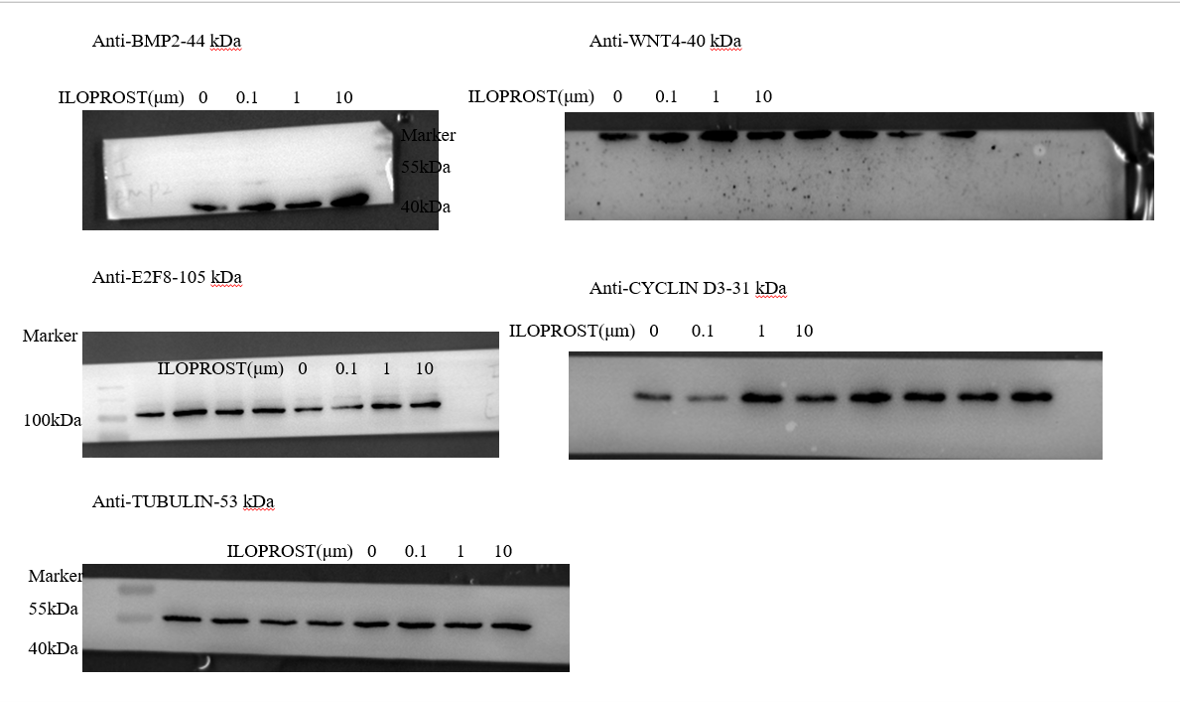

Supplement: Figure 3—source data 2. [file elife-82970-fig3-data2.zip › Figure_3-source_data_2/Figure_3-source_data_2-3E.png]

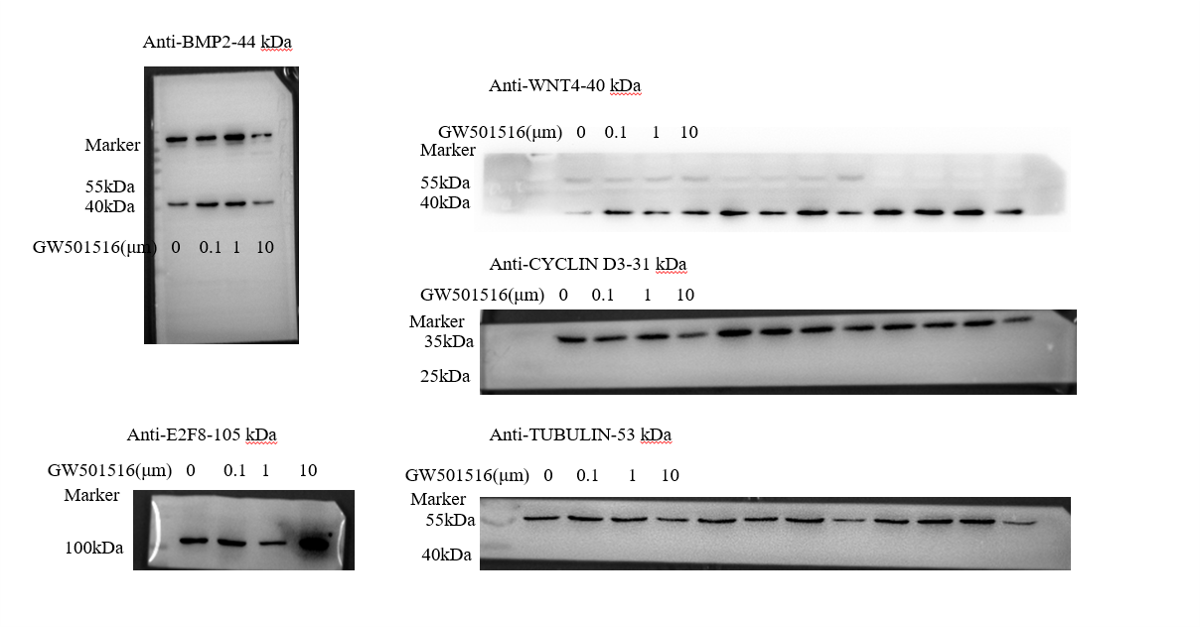

Supplement: Figure 3—source data 2. [file elife-82970-fig3-data2.zip › Figure_3-source_data_2/Figure_3-source_data_2-3F.png]

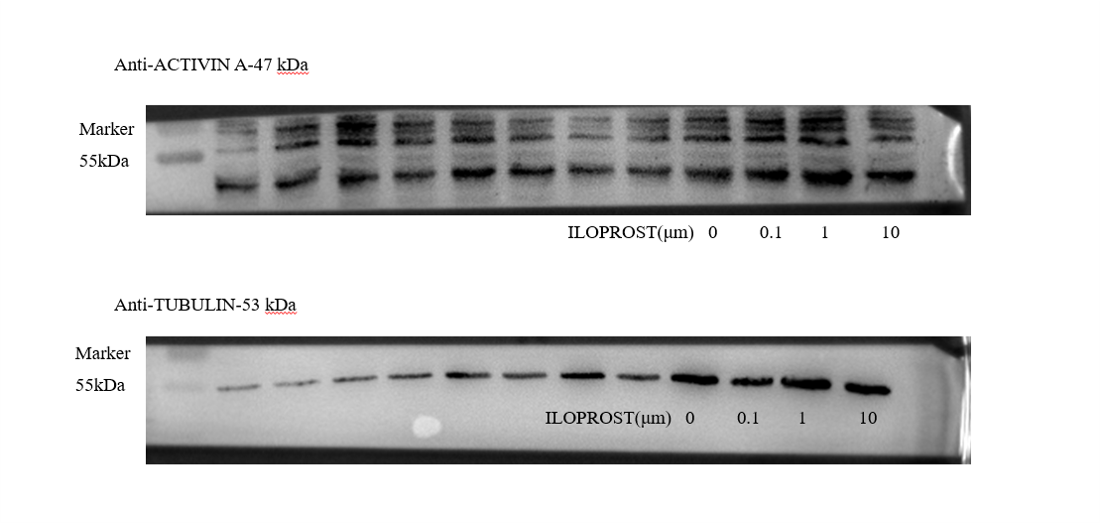

Supplement: Figure 3—source data 2. [file elife-82970-fig3-data2.zip › Figure_3-source_data_2/Figure_3-source_data_2-3G.png]

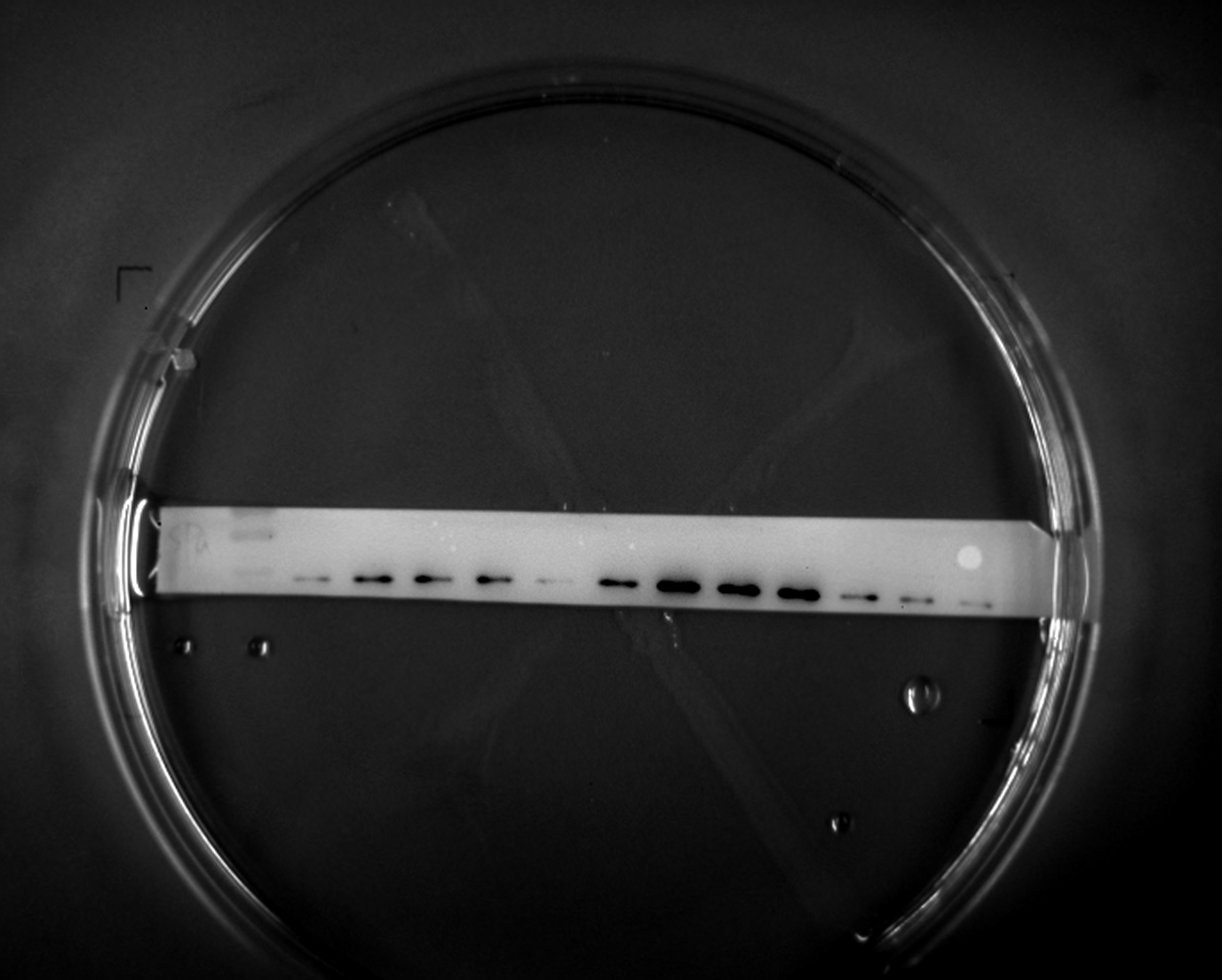

Supplement: Figure 4—source data 1. [file elife-82970-fig4-data1.zip › Figure_4-source_data_1/Figure_4-source_data_1_Figure_4A_SPARC.tif]

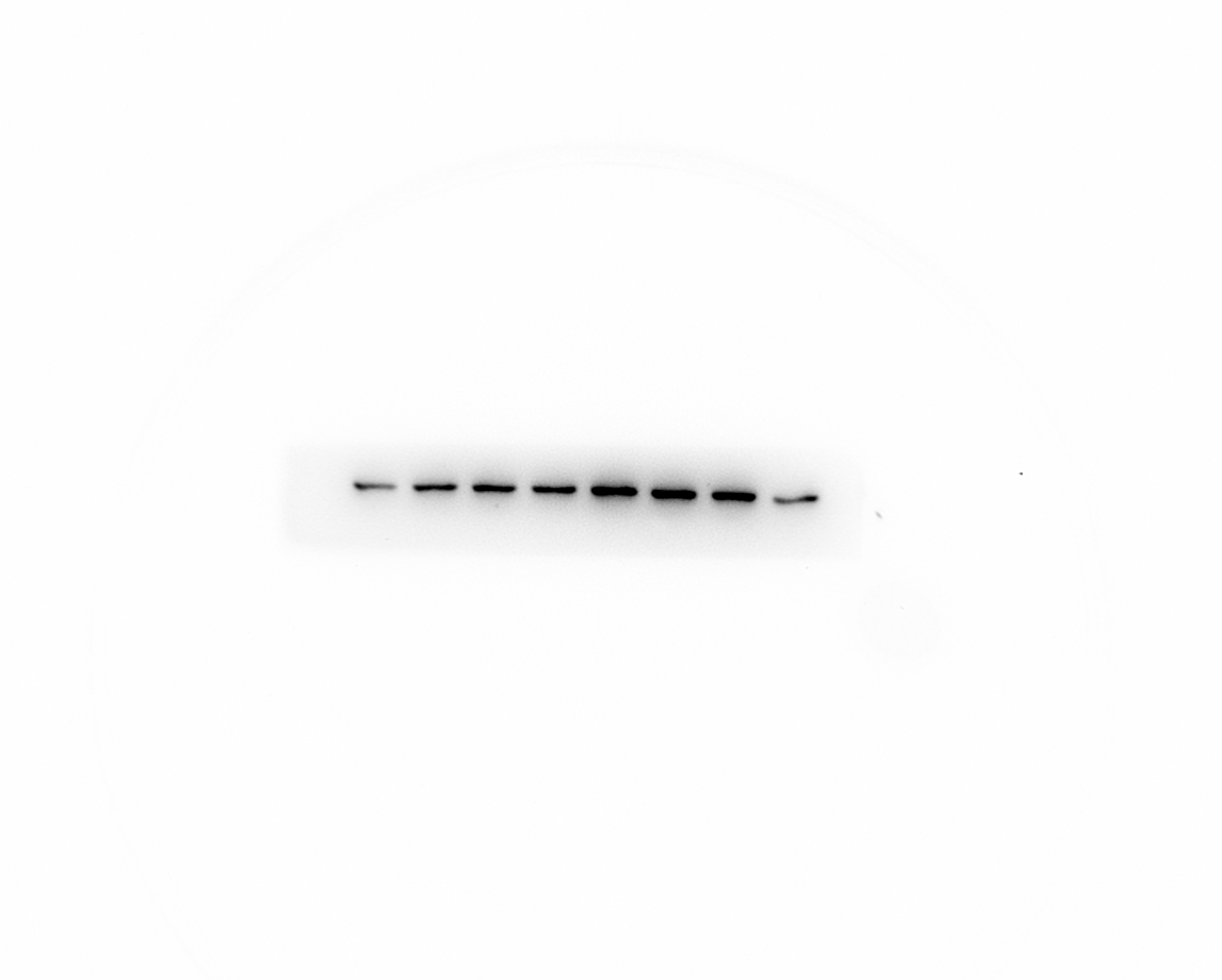

Supplement: Figure 4—source data 1. [file elife-82970-fig4-data1.zip › Figure_4-source_data_1/Figure_4-source_data_1_Figure_4A_TNC.tif]

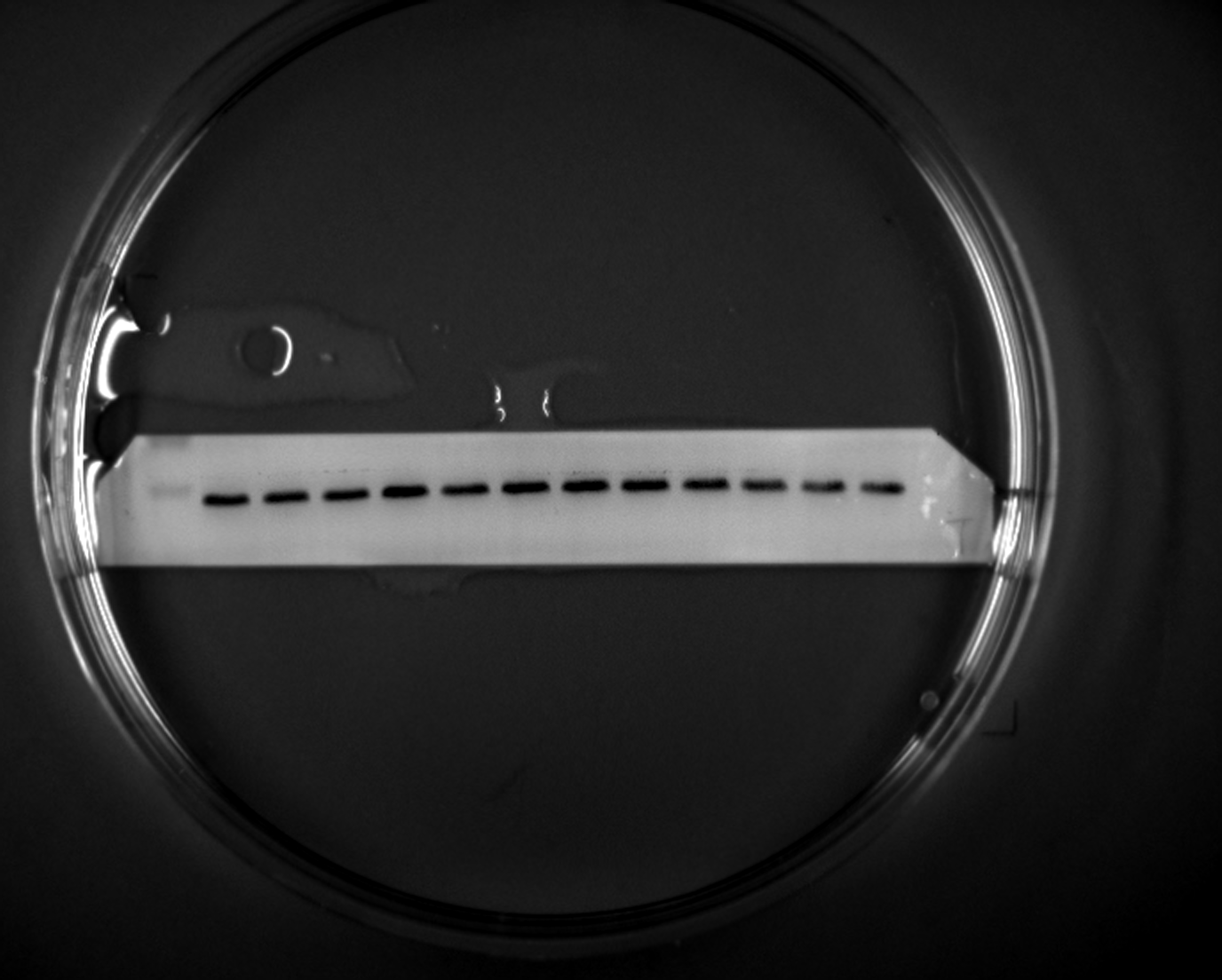

Supplement: Figure 4—source data 1. [file elife-82970-fig4-data1.zip › Figure_4-source_data_1/Figure_4-source_data_1_Figure_4A_TUBULIN.tif]

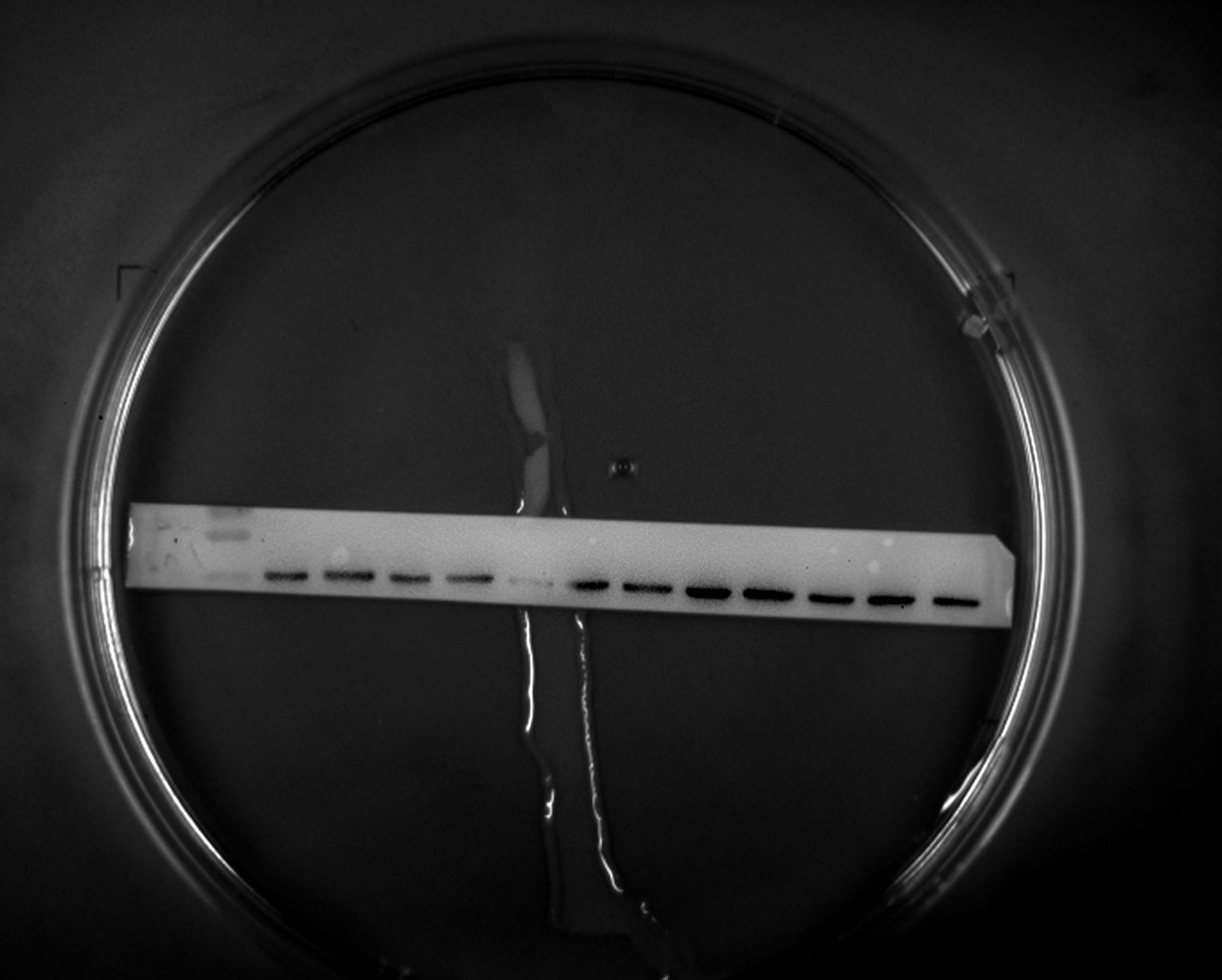

Supplement: Figure 4—source data 1. [file elife-82970-fig4-data1.zip › Figure_4-source_data_1/Figure_4-source_data_1_Figure_4A_a┴-SMA.tif]

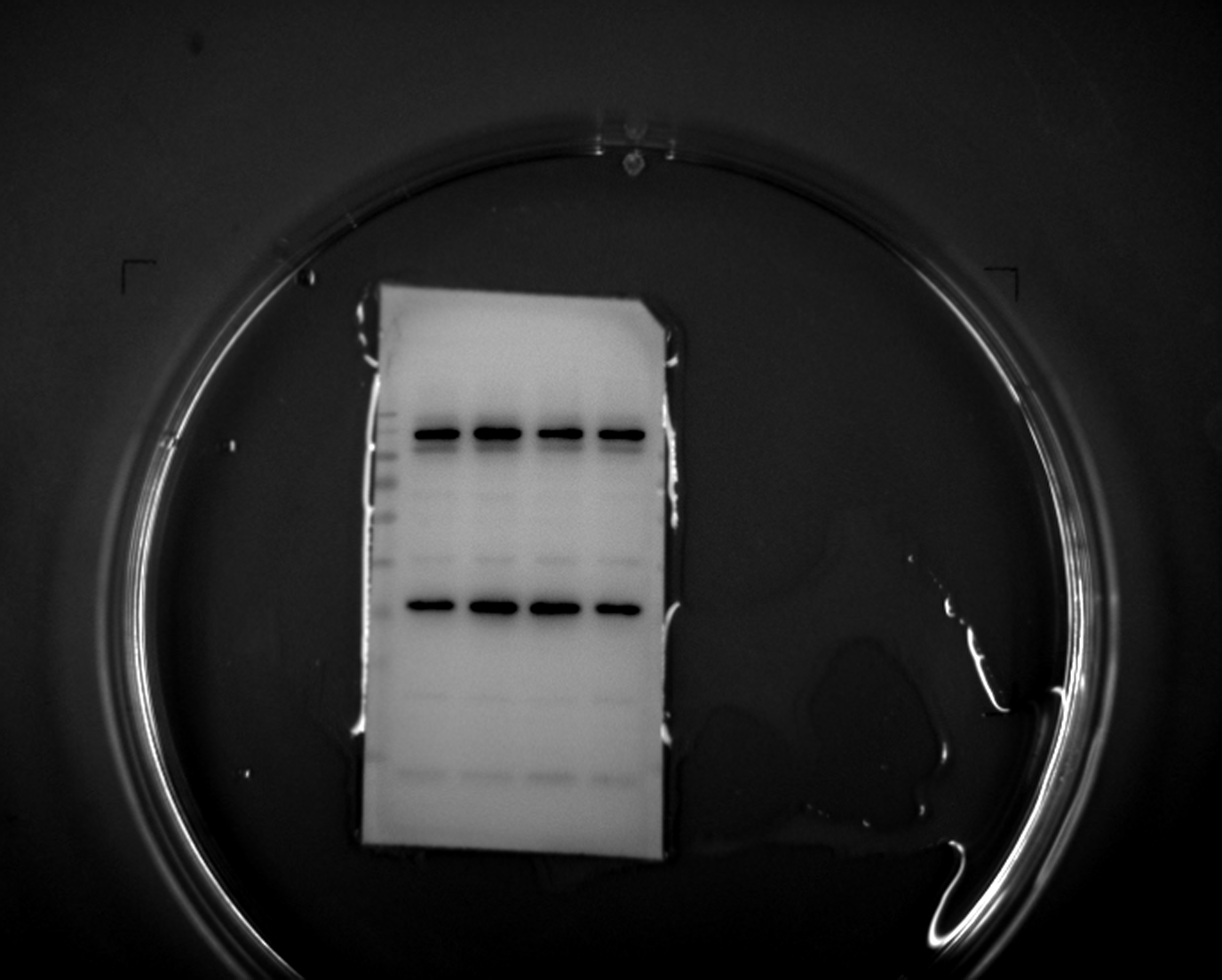

Supplement: Figure 4—source data 1. [file elife-82970-fig4-data1.zip › Figure_4-source_data_1/Figure_4-source_data_1_Figure_4B_BMP2.tif]

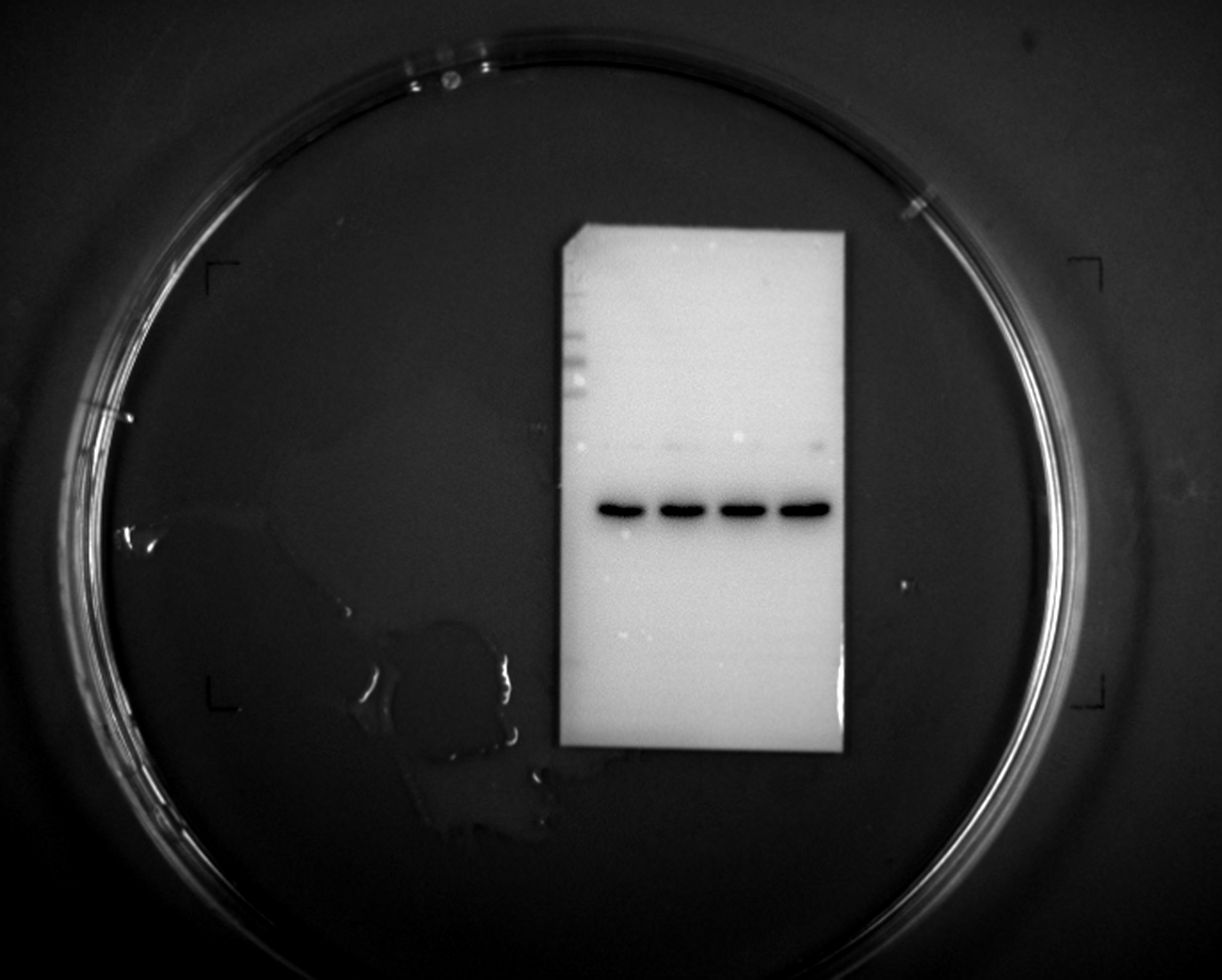

Supplement: Figure 4—source data 1. [file elife-82970-fig4-data1.zip › Figure_4-source_data_1/Figure_4-source_data_1_Figure_4B_CYCLIN D3.tif]

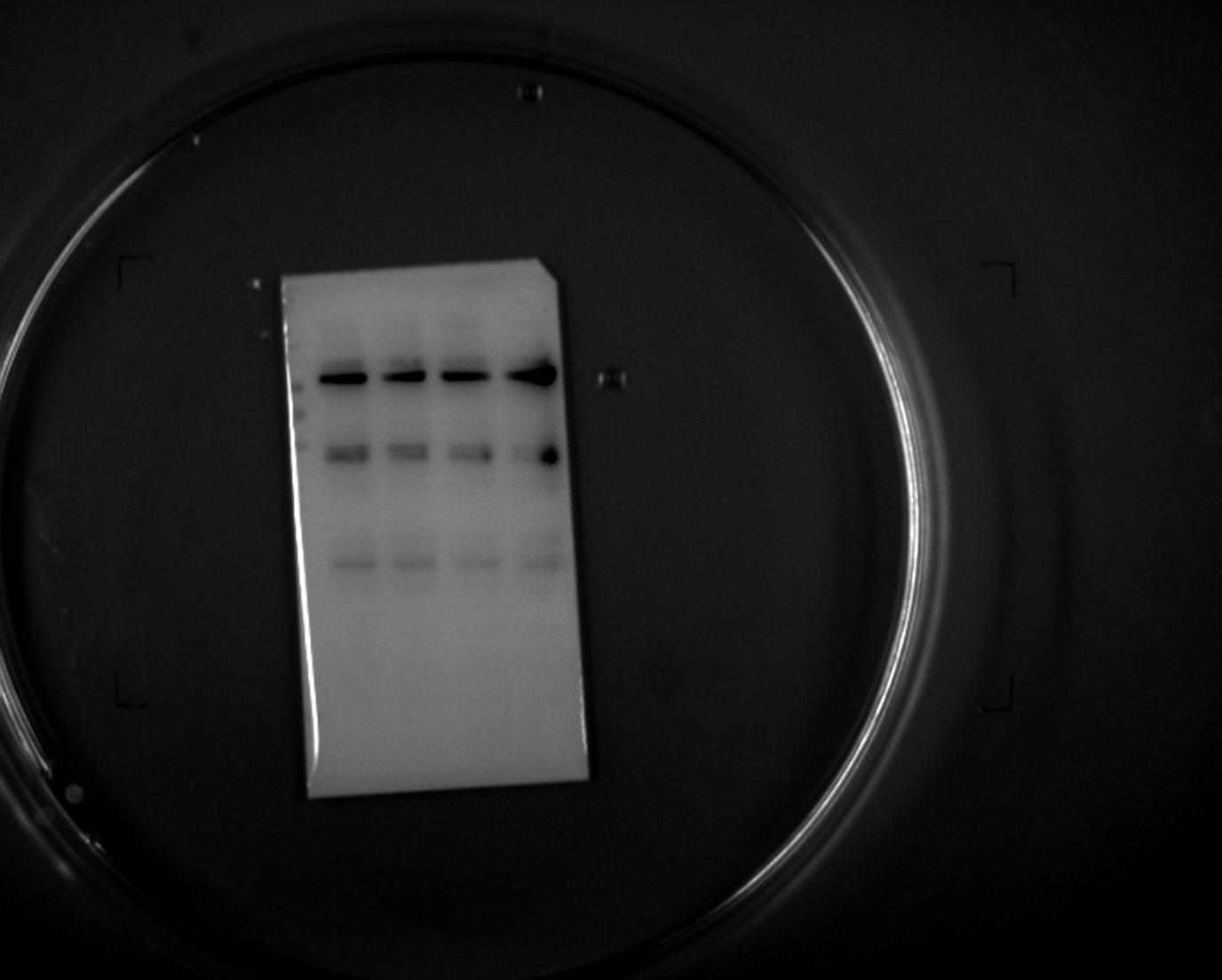

Supplement: Figure 4—source data 1. [file elife-82970-fig4-data1.zip › Figure_4-source_data_1/Figure_4-source_data_1_Figure_4B_E2F8.tif]

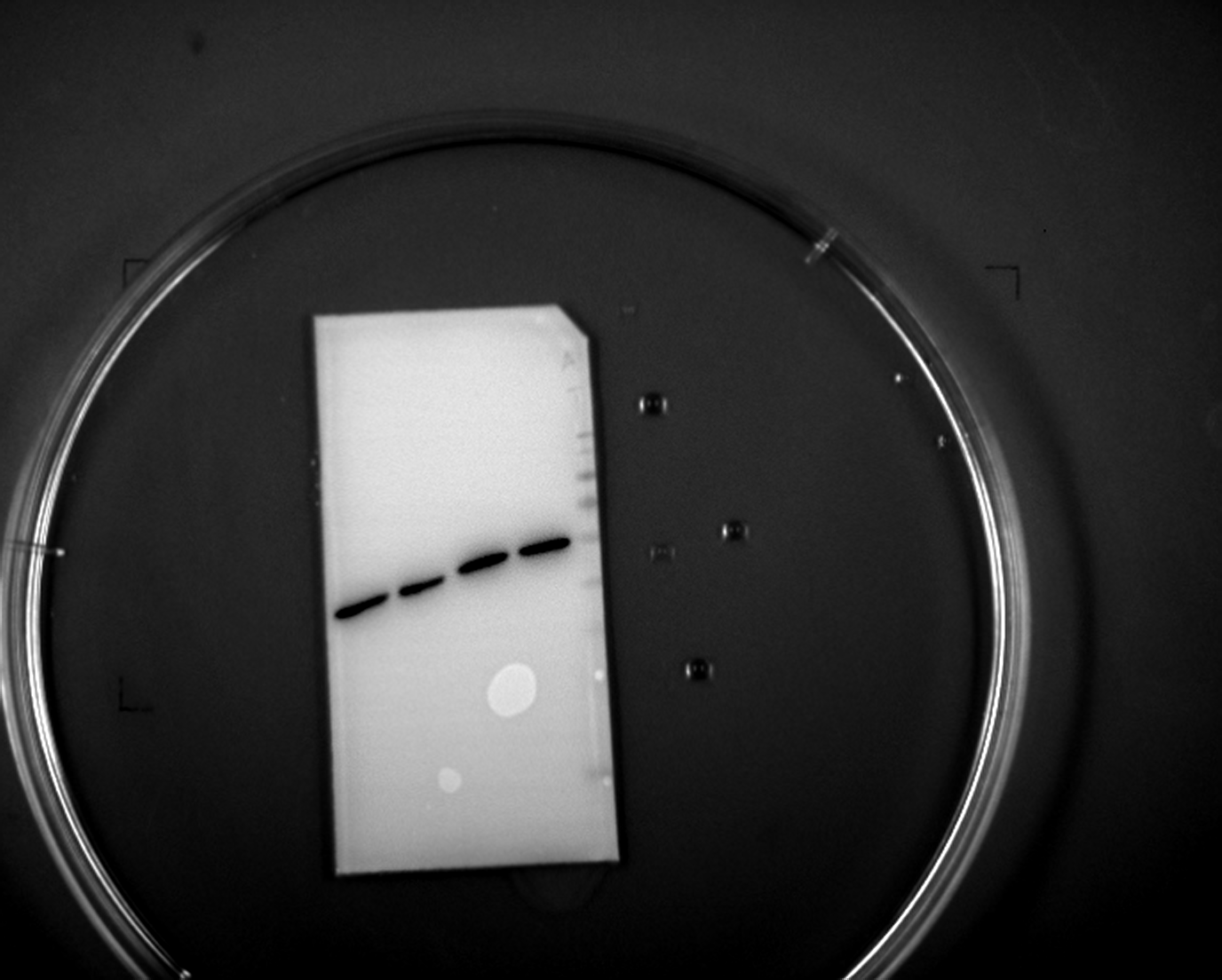

Supplement: Figure 4—source data 1. [file elife-82970-fig4-data1.zip › Figure_4-source_data_1/Figure_4-source_data_1_Figure_4B_TUBULIN.tif]

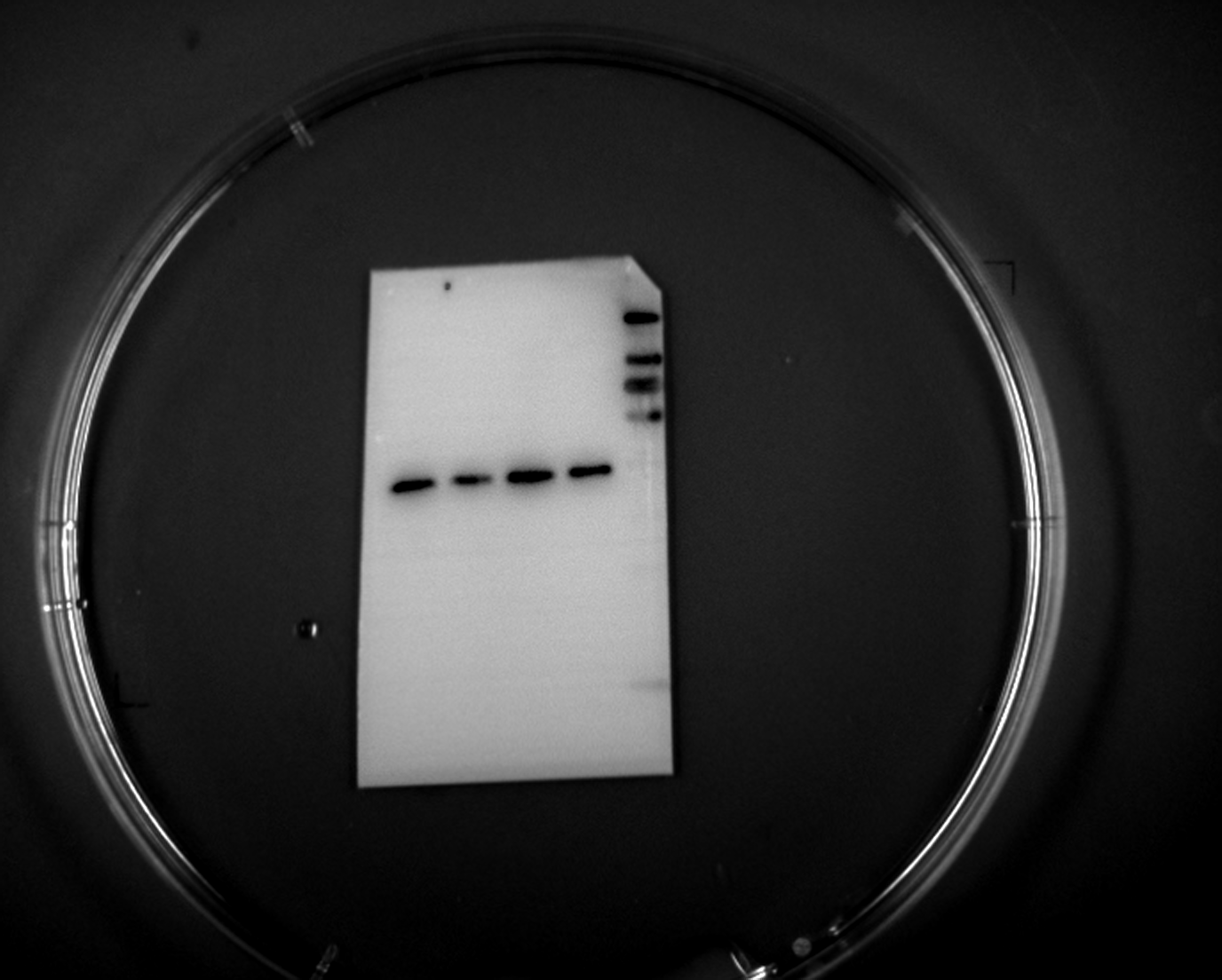

Supplement: Figure 4—source data 1. [file elife-82970-fig4-data1.zip › Figure_4-source_data_1/Figure_4-source_data_1_Figure_4B_WNT4.tif]

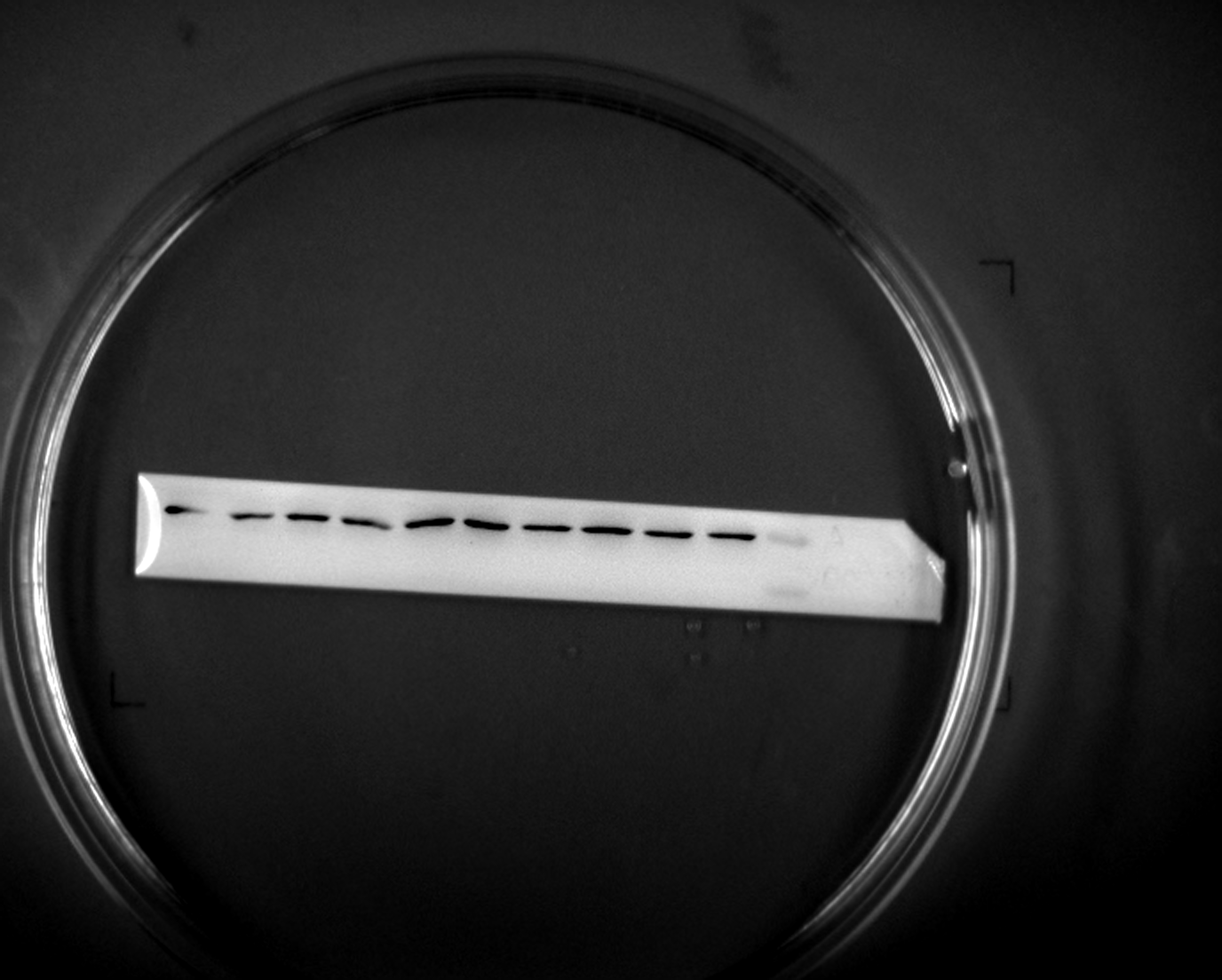

Supplement: Figure 4—source data 1. [file elife-82970-fig4-data1.zip › Figure_4-source_data_1/Figure_4-source_data_1_Figure_4C_TUBULIN.tif]

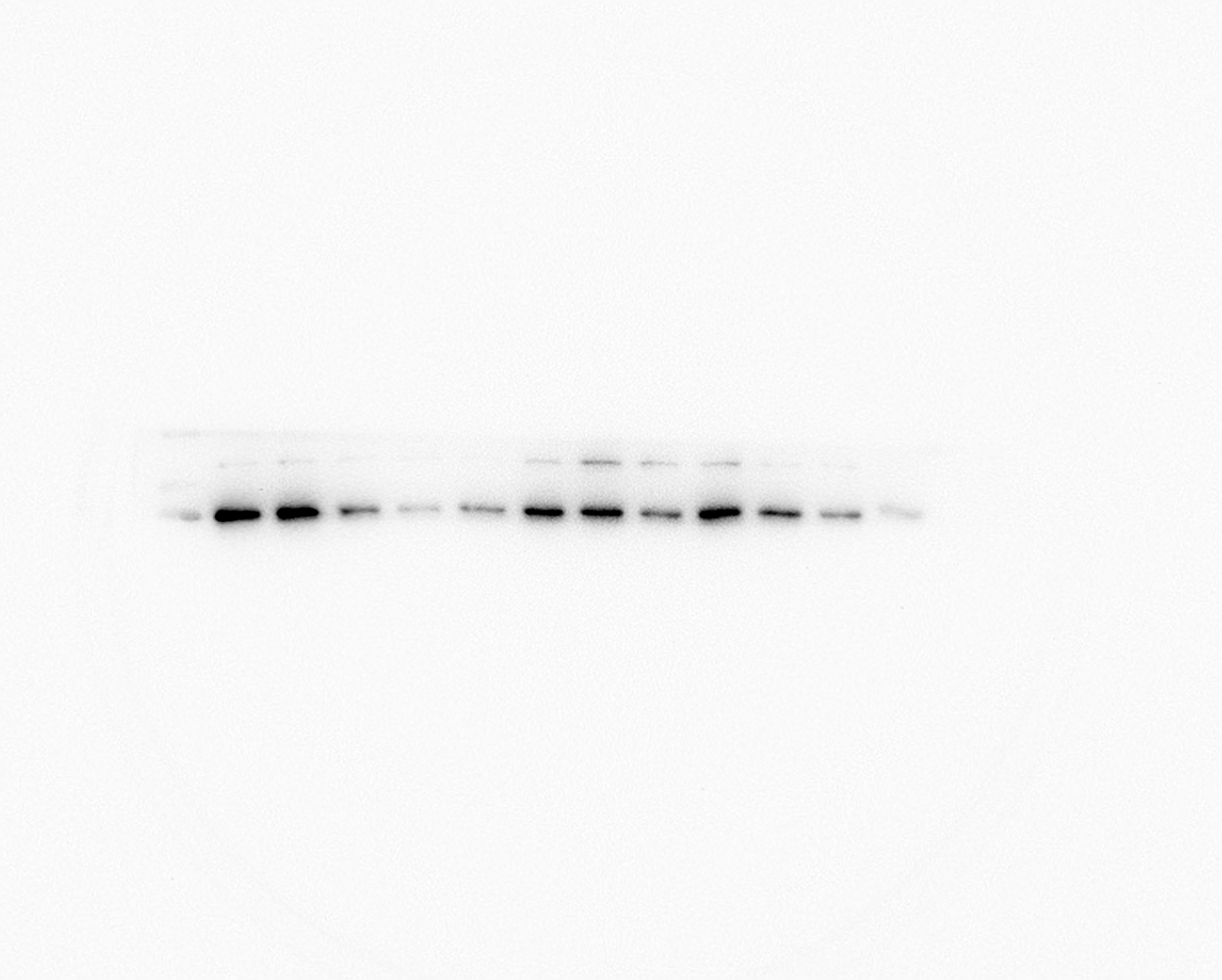

Supplement: Figure 4—source data 1. [file elife-82970-fig4-data1.zip › Figure_4-source_data_1/Figure_4-source_data_1_Figure_4E_COX2.tif]

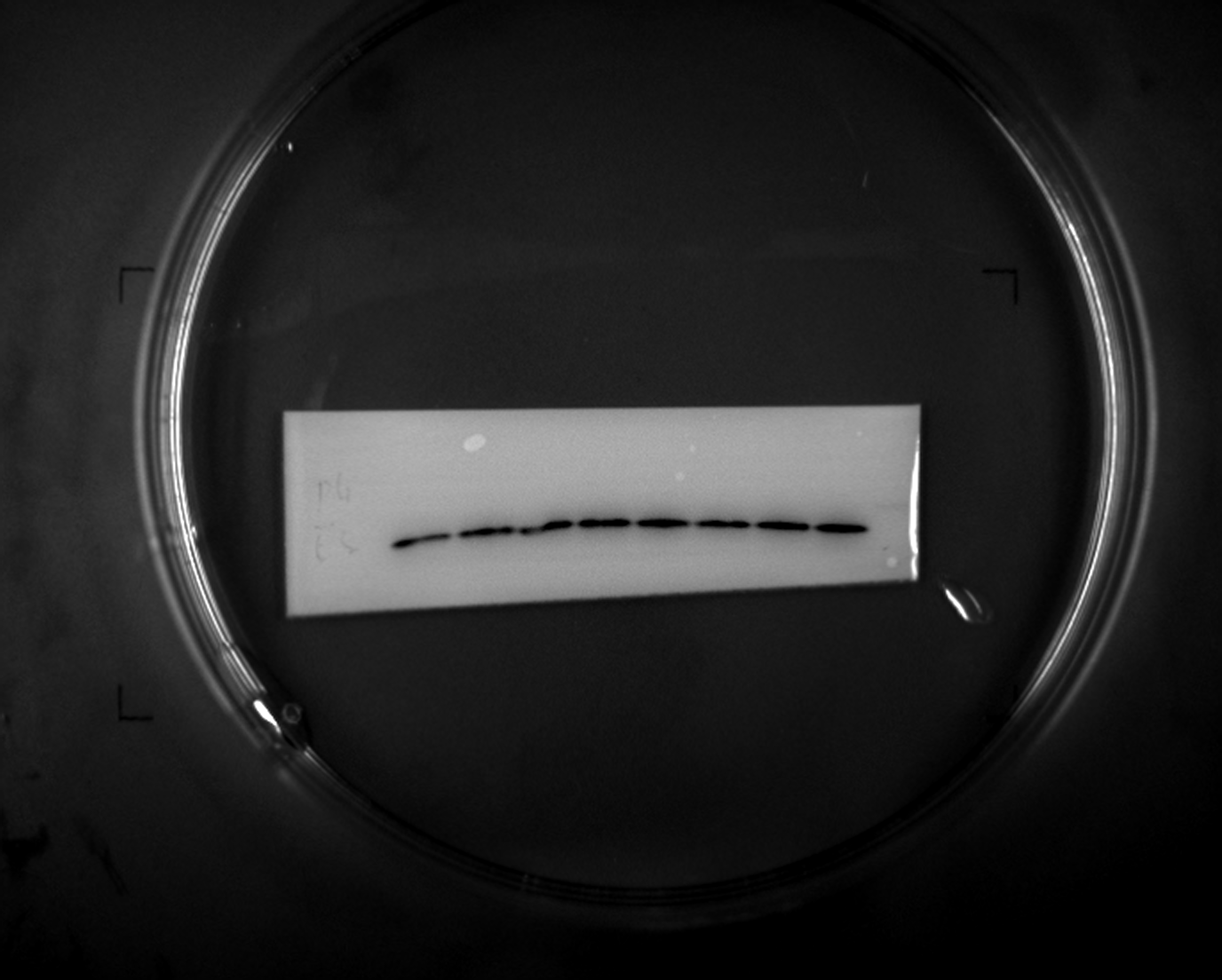

Supplement: Figure 4—source data 1. [file elife-82970-fig4-data1.zip › Figure_4-source_data_1/Figure_4-source_data_1_Figure_4E_PGES.tif]

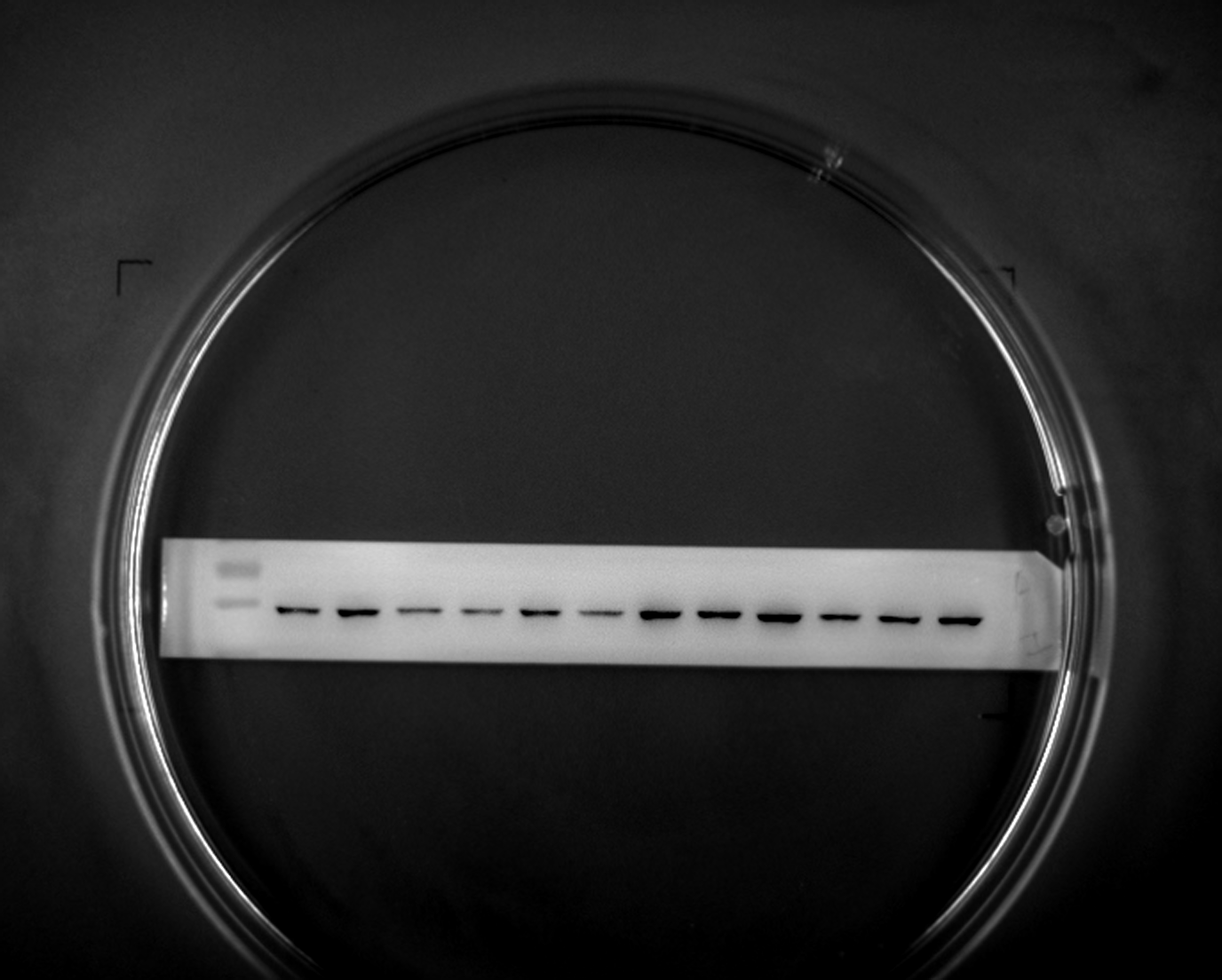

Supplement: Figure 4—source data 1. [file elife-82970-fig4-data1.zip › Figure_4-source_data_1/Figure_4-source_data_1_Figure_4E_PGIS.tif]

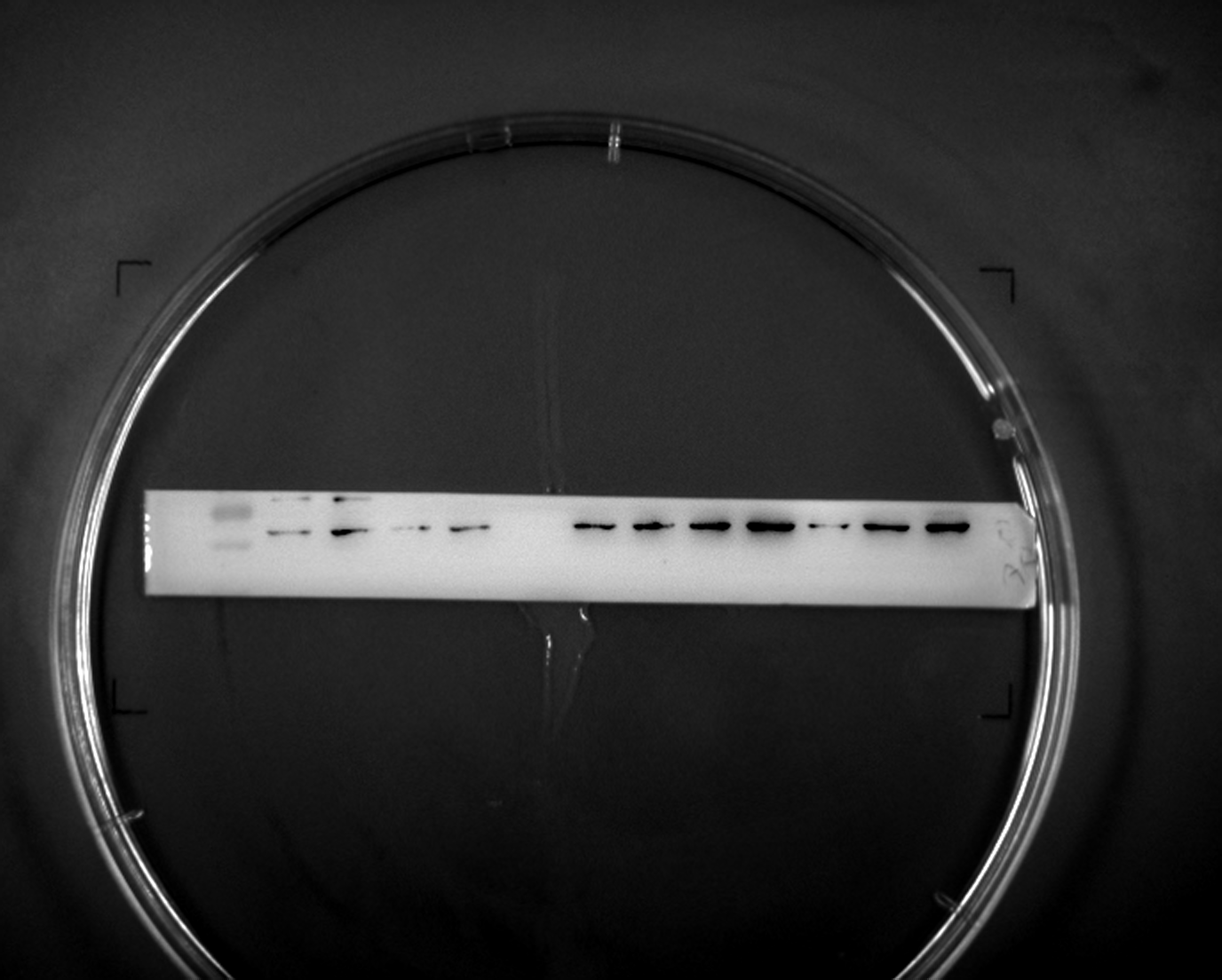

Supplement: Figure 4—source data 1. [file elife-82970-fig4-data1.zip › Figure_4-source_data_1/Figure_4-source_data_1_Figure_4E_PPARa─.tif]

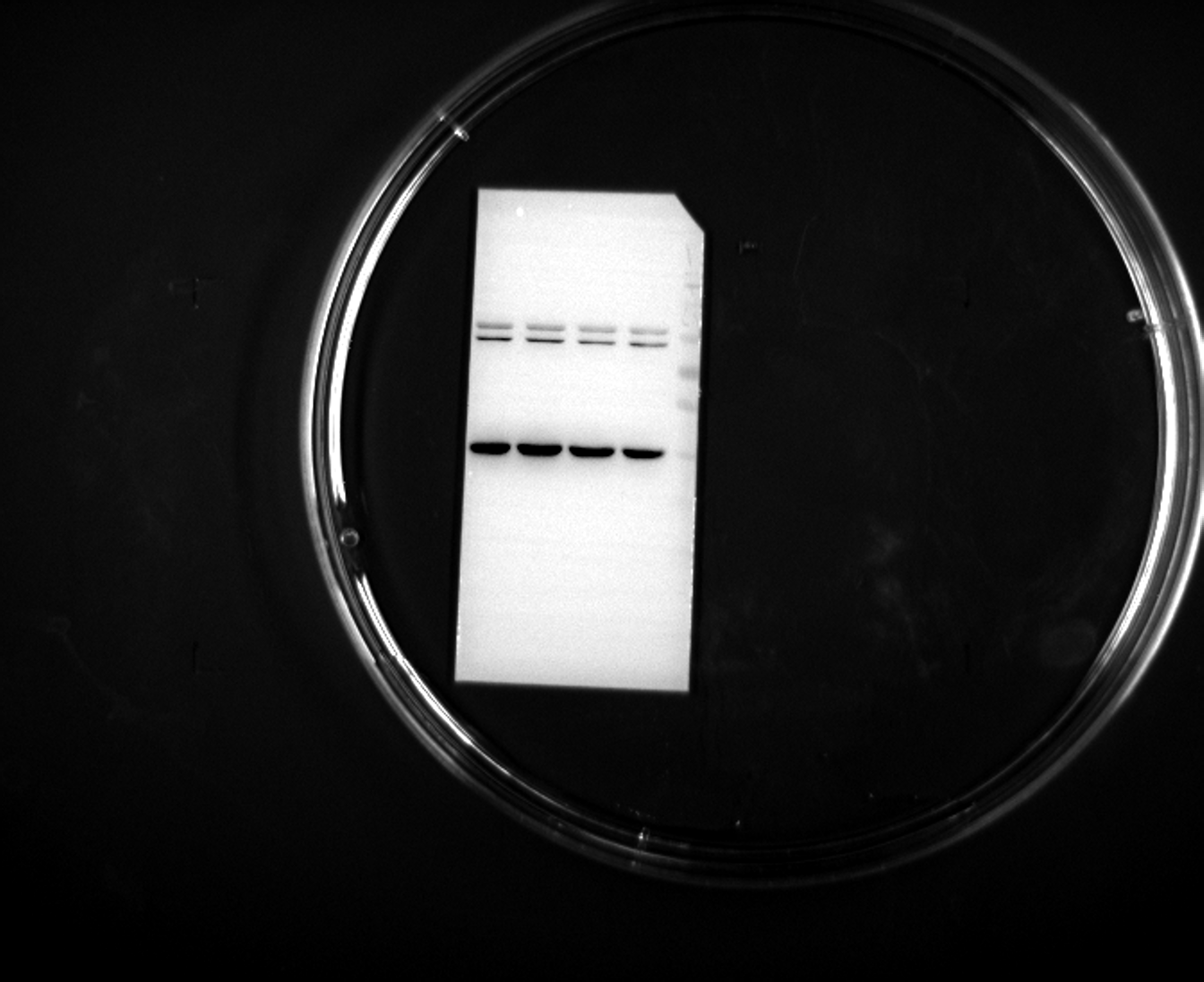

Supplement: Figure 4—source data 1. [file elife-82970-fig4-data1.zip › Figure_4-source_data_1/Figure_4-source_data_1_Figure_4E_PTGIR.Tif]

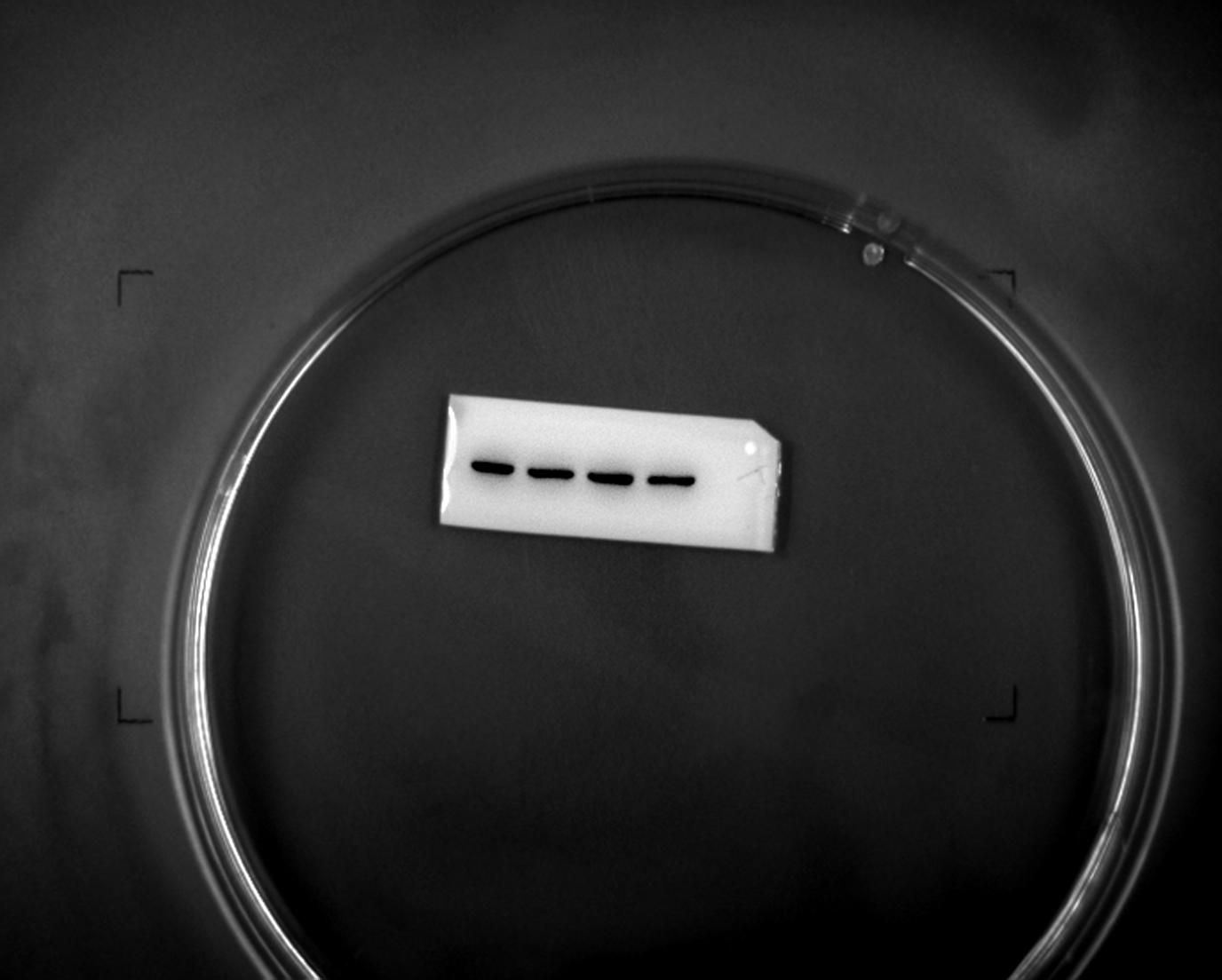

Supplement: Figure 4—source data 1. [file elife-82970-fig4-data1.zip › Figure_4-source_data_1/Figure_4-source_data_1_Figure_4E_TUBULIN.tif]

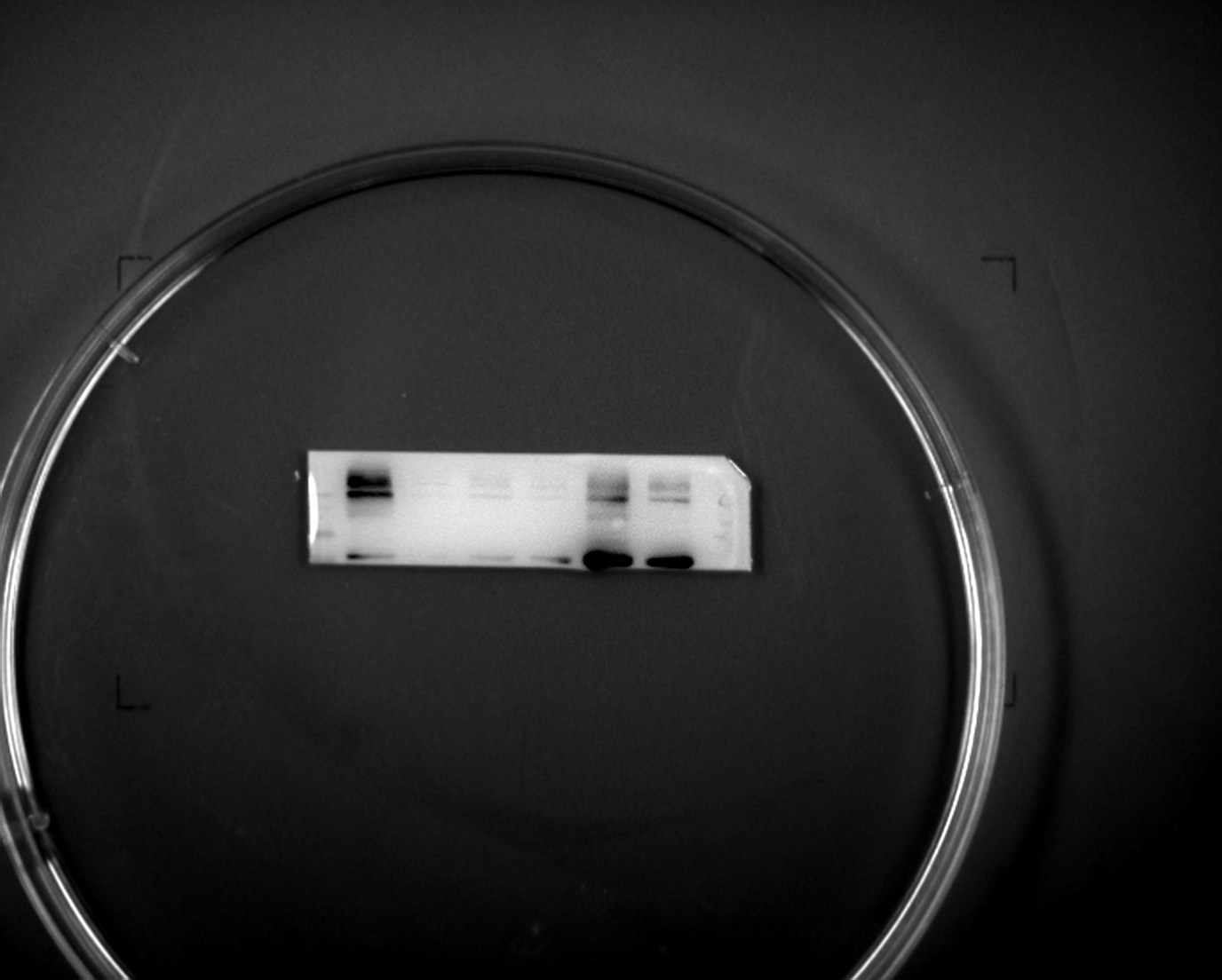

Supplement: Figure 4—source data 1. [file elife-82970-fig4-data1.zip › Figure_4-source_data_1/Figure_4-source_data_1_Figure_4F_COX2.tif]

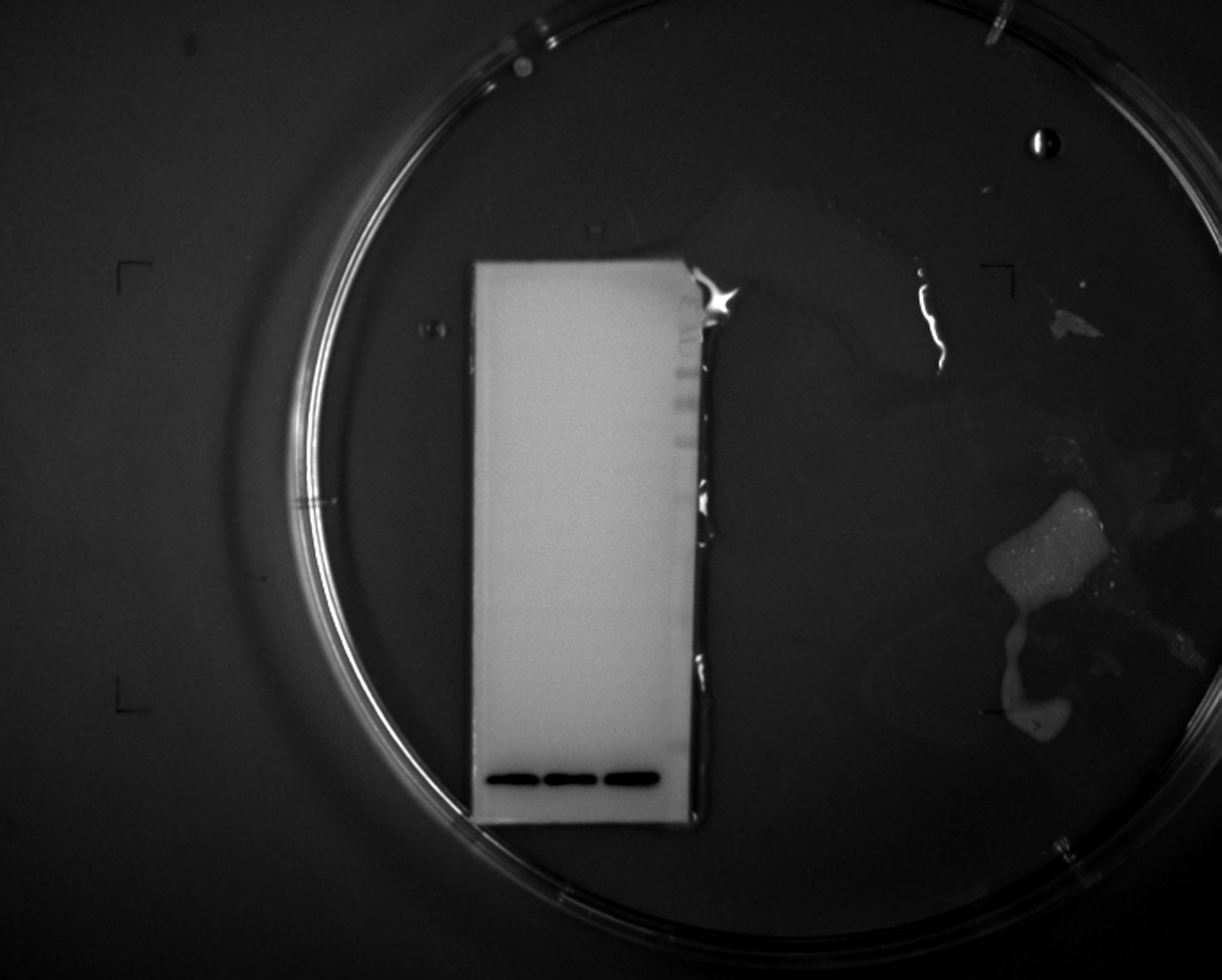

Supplement: Figure 4—source data 1. [file elife-82970-fig4-data1.zip › Figure_4-source_data_1/Figure_4-source_data_1_Figure_4F_PGES.tif]

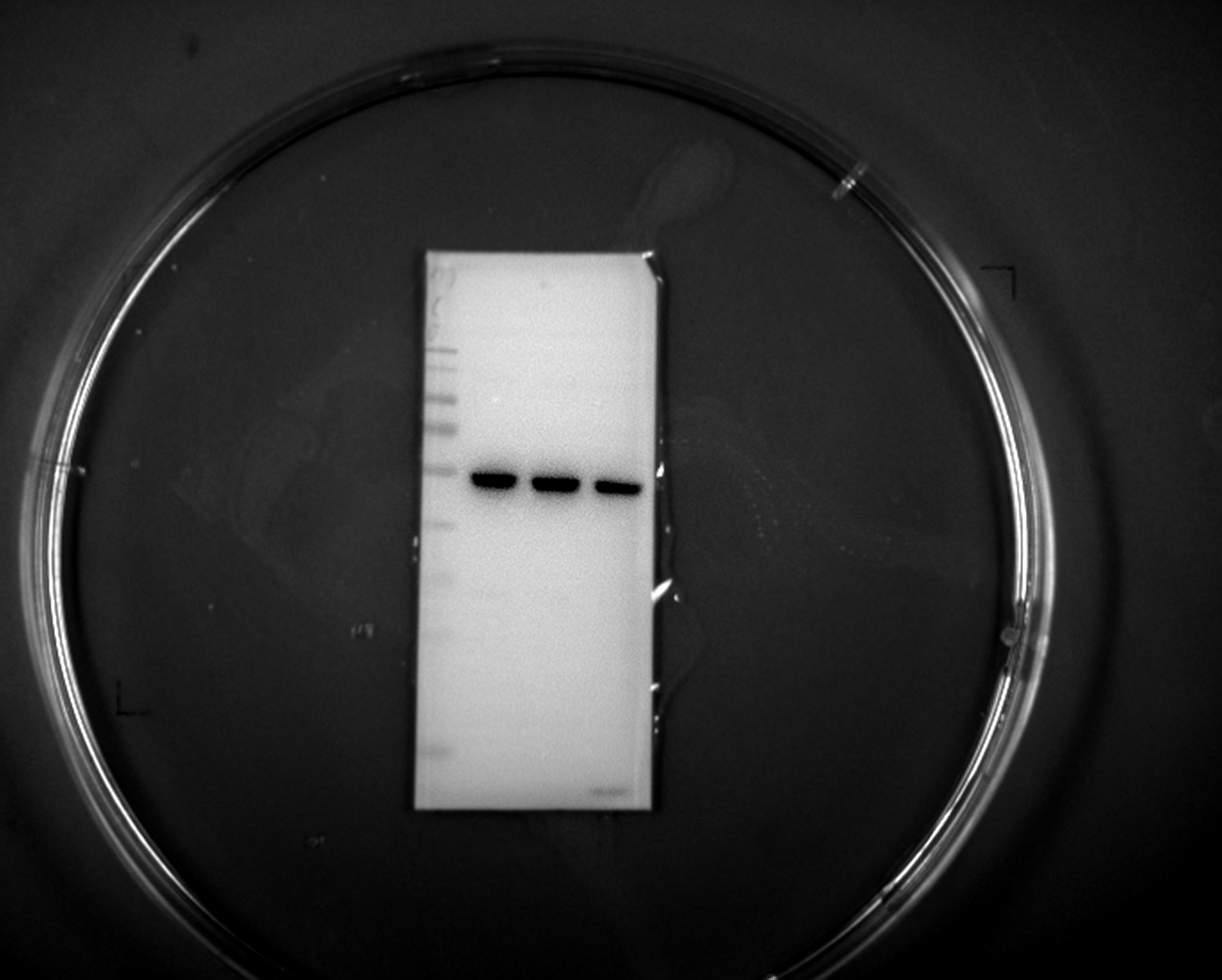

Supplement: Figure 4—source data 1. [file elife-82970-fig4-data1.zip › Figure_4-source_data_1/Figure_4-source_data_1_Figure_4F_PGIS.tif]

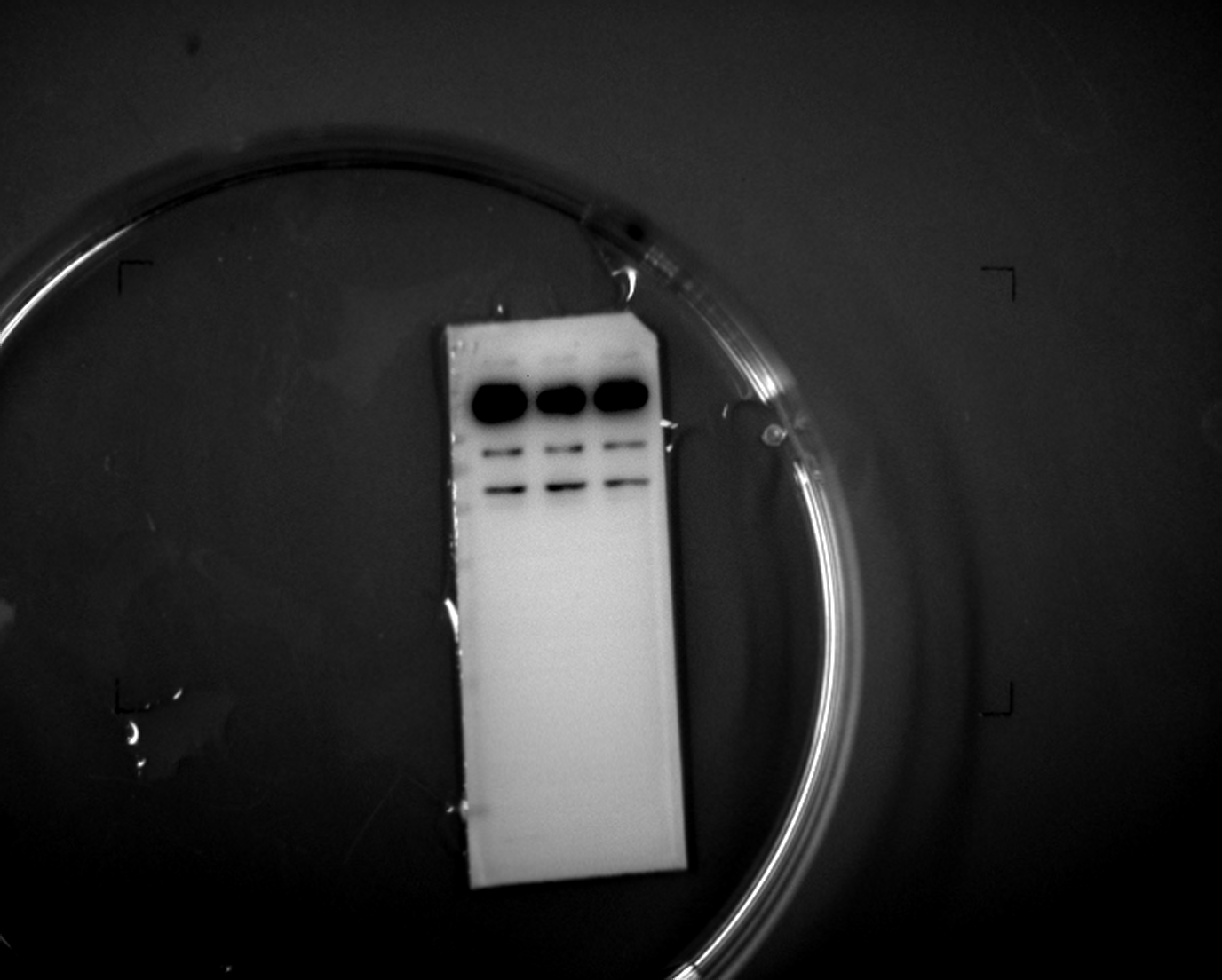

Supplement: Figure 4—source data 1. [file elife-82970-fig4-data1.zip › Figure_4-source_data_1/Figure_4-source_data_1_Figure_4F_PPARa─.tif]

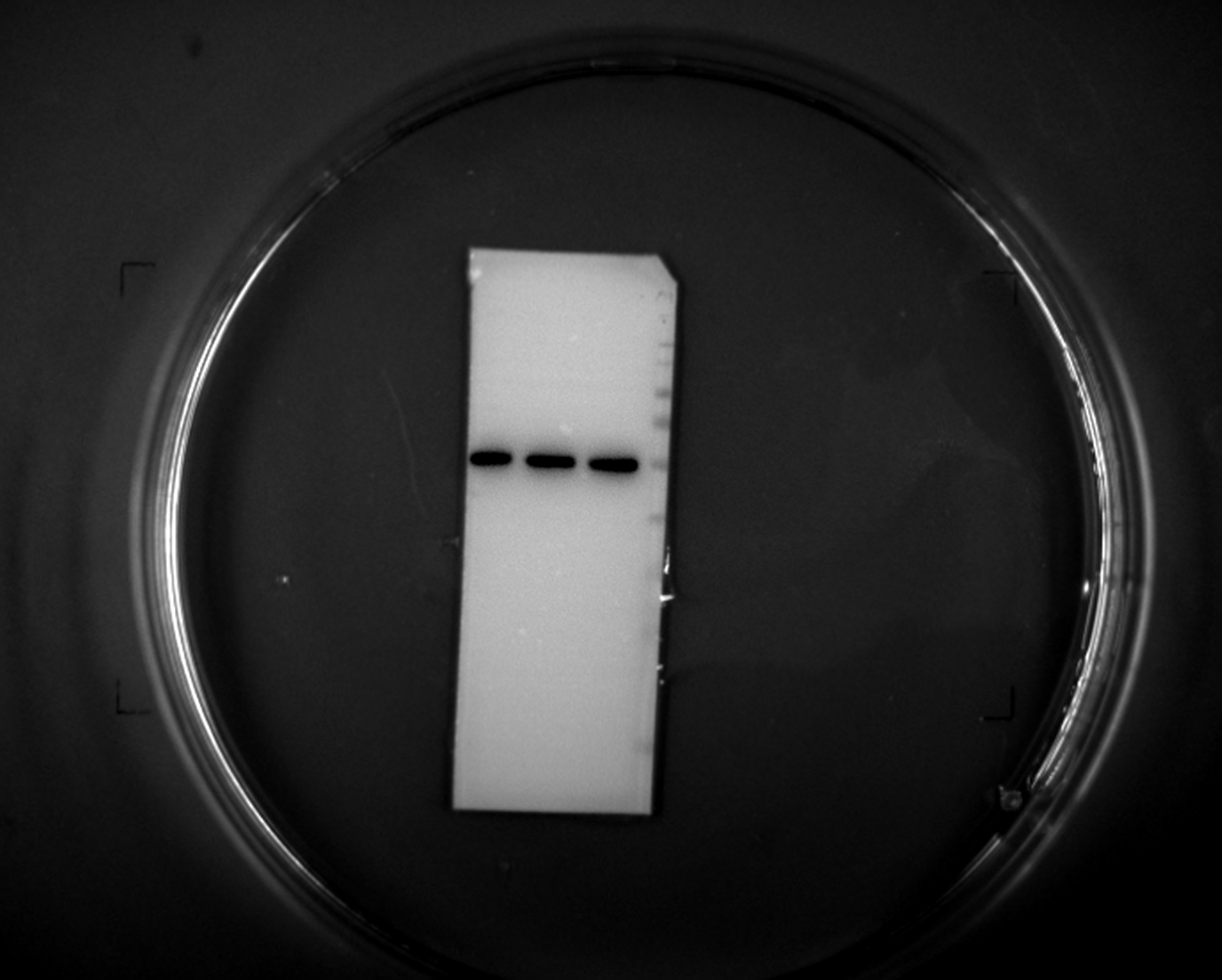

Supplement: Figure 4—source data 1. [file elife-82970-fig4-data1.zip › Figure_4-source_data_1/Figure_4-source_data_1_Figure_4F_TUBULIN.tif]

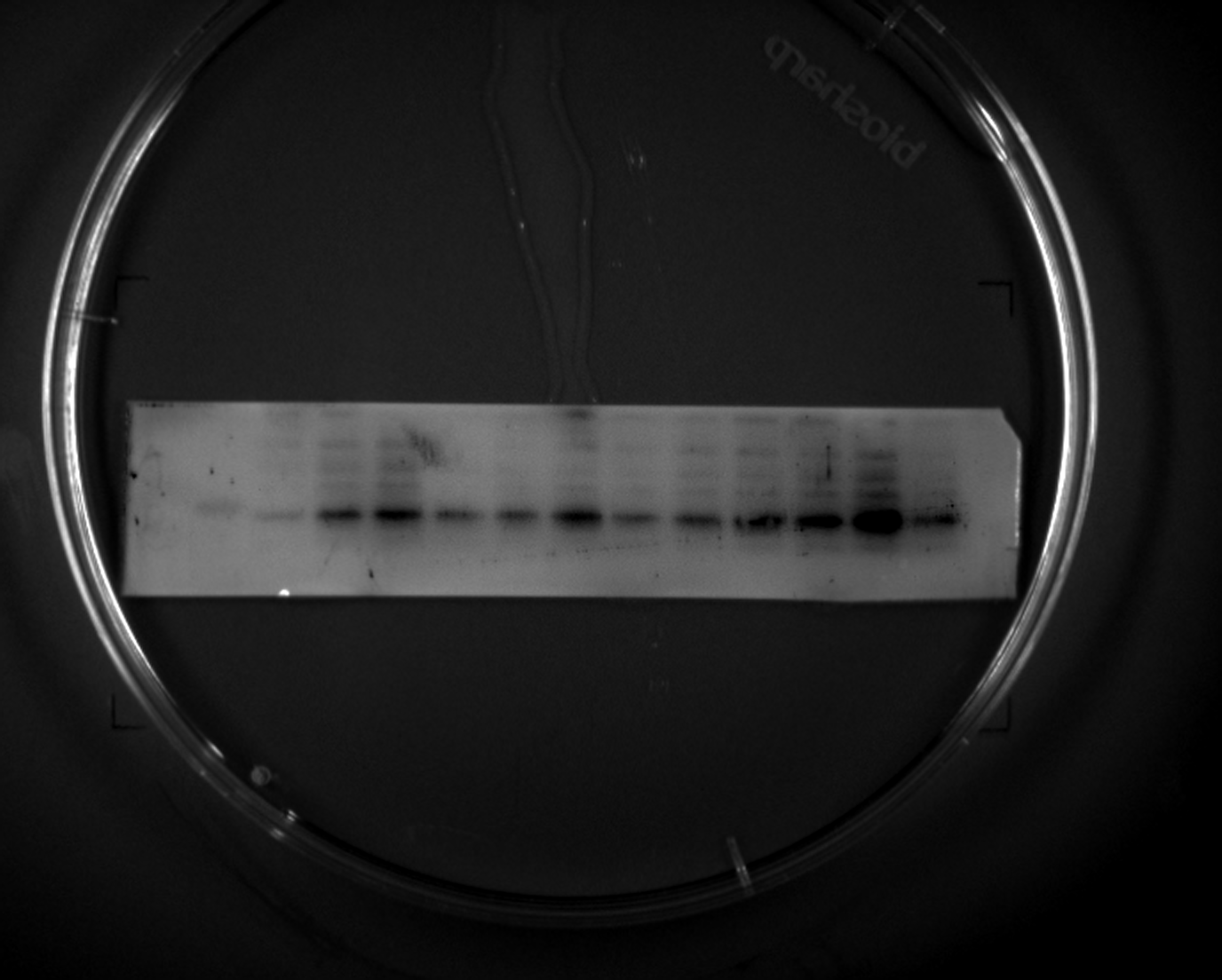

Supplement: Figure 4—source data 1. [file elife-82970-fig4-data1.zip › Figure_4-source_data_1/Figure_4-source_data_1_Figure_4J_ACTIVIN A.tif]

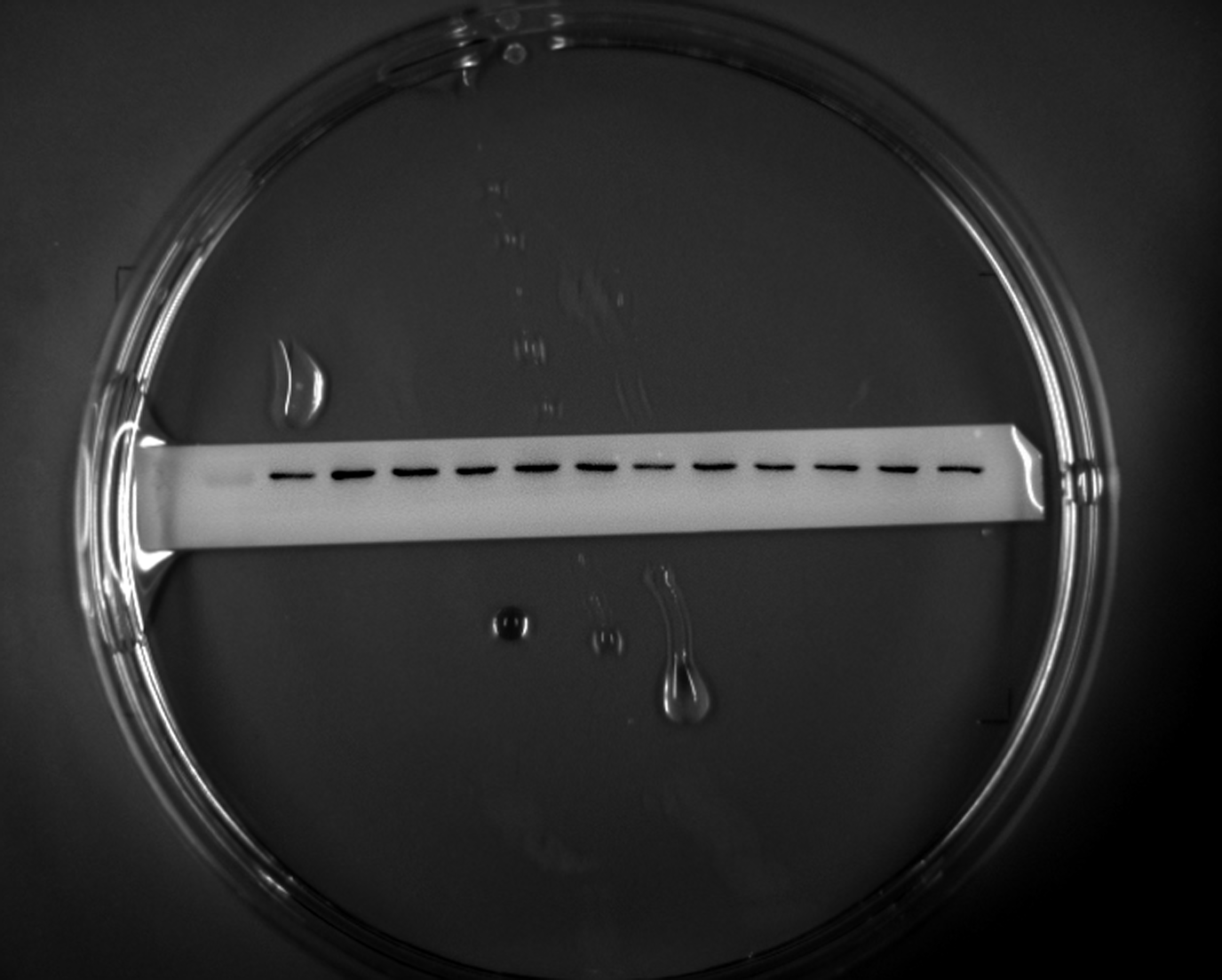

Supplement: Figure 4—source data 1. [file elife-82970-fig4-data1.zip › Figure_4-source_data_1/Figure_4-source_data_1_Figure_4J_TUBULIN.tif]

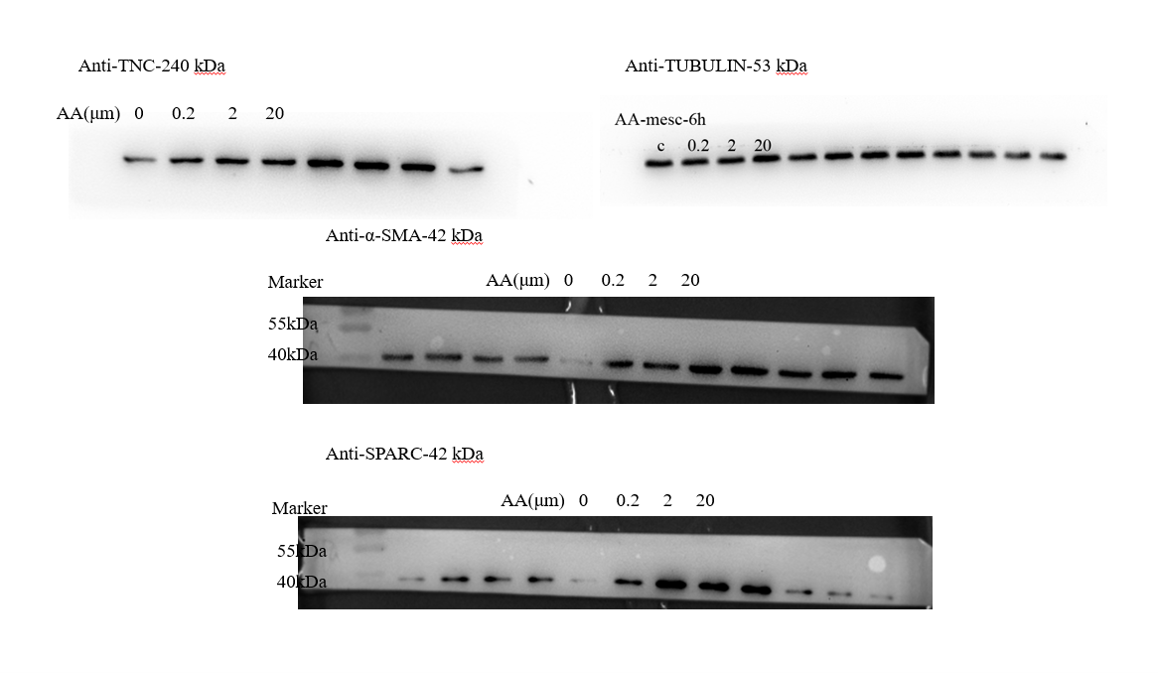

Supplement: Figure 4—source data 2. [file elife-82970-fig4-data2.zip › Figure_4-source_data_2/Figure_4-source_data_2-4A.png]

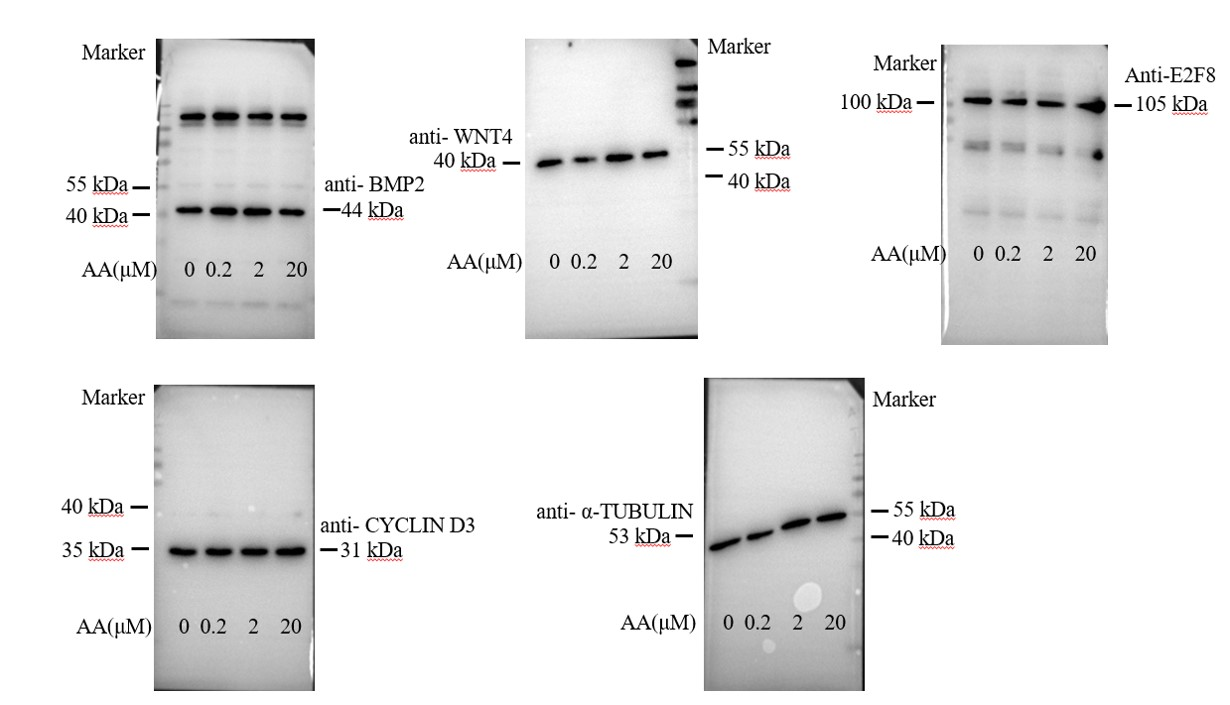

Supplement: Figure 4—source data 2. [file elife-82970-fig4-data2.zip › Figure_4-source_data_2/Figure_4-source_data_2-4B.png]

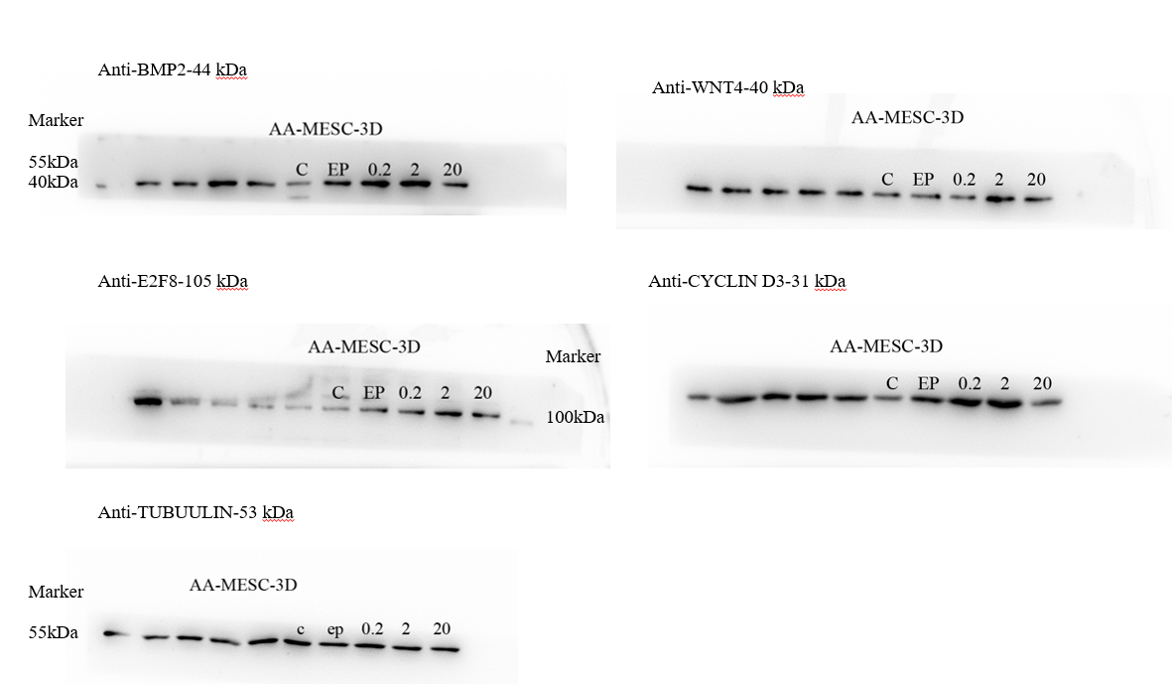

Supplement: Figure 4—source data 2. [file elife-82970-fig4-data2.zip › Figure_4-source_data_2/Figure_4-source_data_2-4C.png]
