# Supplementary figures and images for: Embryo-derive TNF promotes decidualization via fibroblast activation (part 2 of 2)
Source: eLife. 2023 Jul 17;12:e82970. doi: 10.7554/eLife.82970 (PMC10374279; doi:10.7554/eLife.82970)

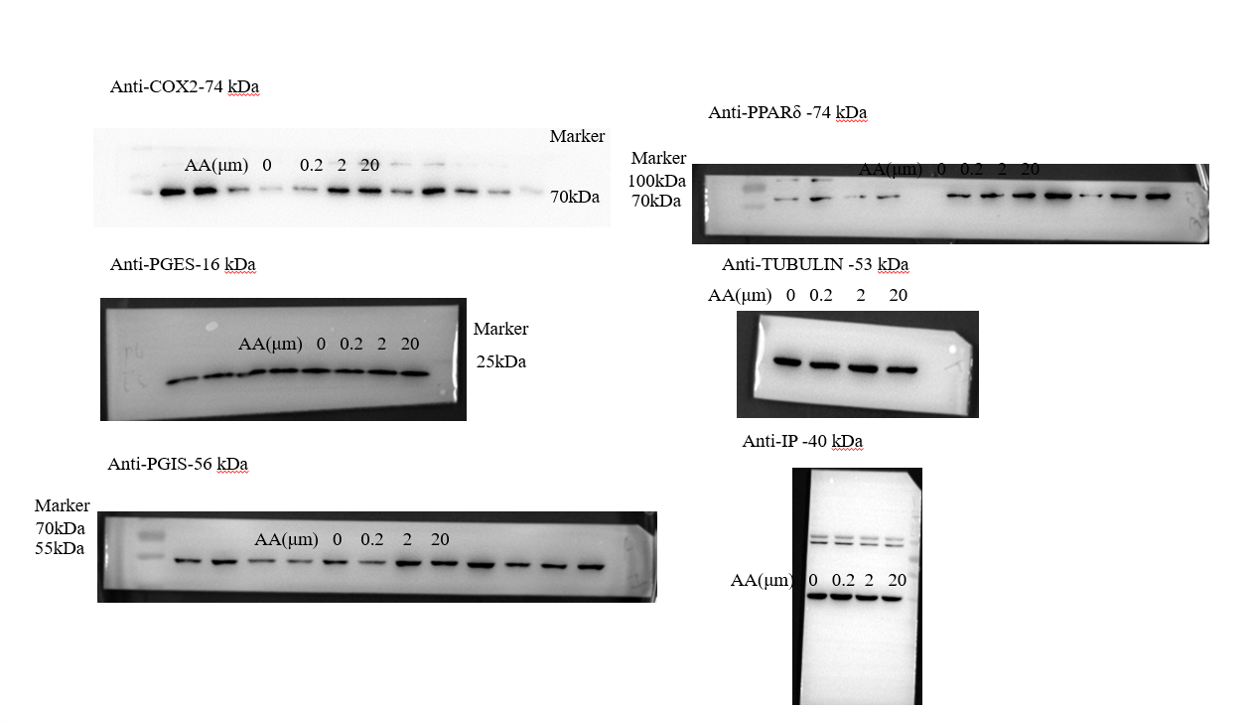

Supplement: Figure 4—source data 2. [file elife-82970-fig4-data2.zip › Figure_4-source_data_2/Figure_4-source_data_2-4E.png]

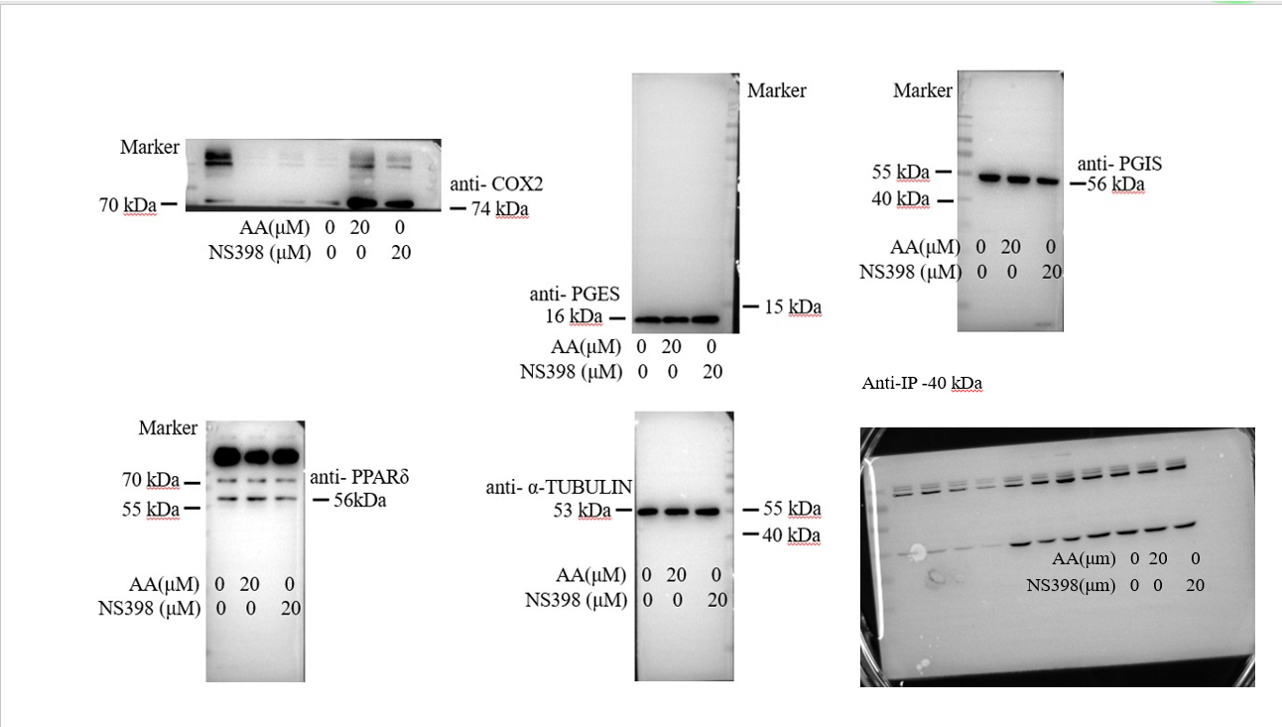

Supplement: Figure 4—source data 2. [file elife-82970-fig4-data2.zip › Figure_4-source_data_2/Figure_4-source_data_2-4F.png]

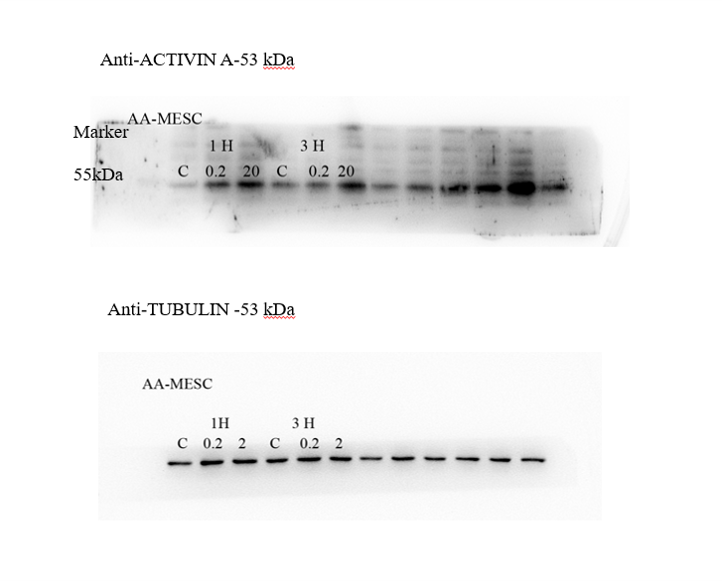

Supplement: Figure 4—source data 2. [file elife-82970-fig4-data2.zip › Figure_4-source_data_2/Figure_4-source_data_2-4J.png]

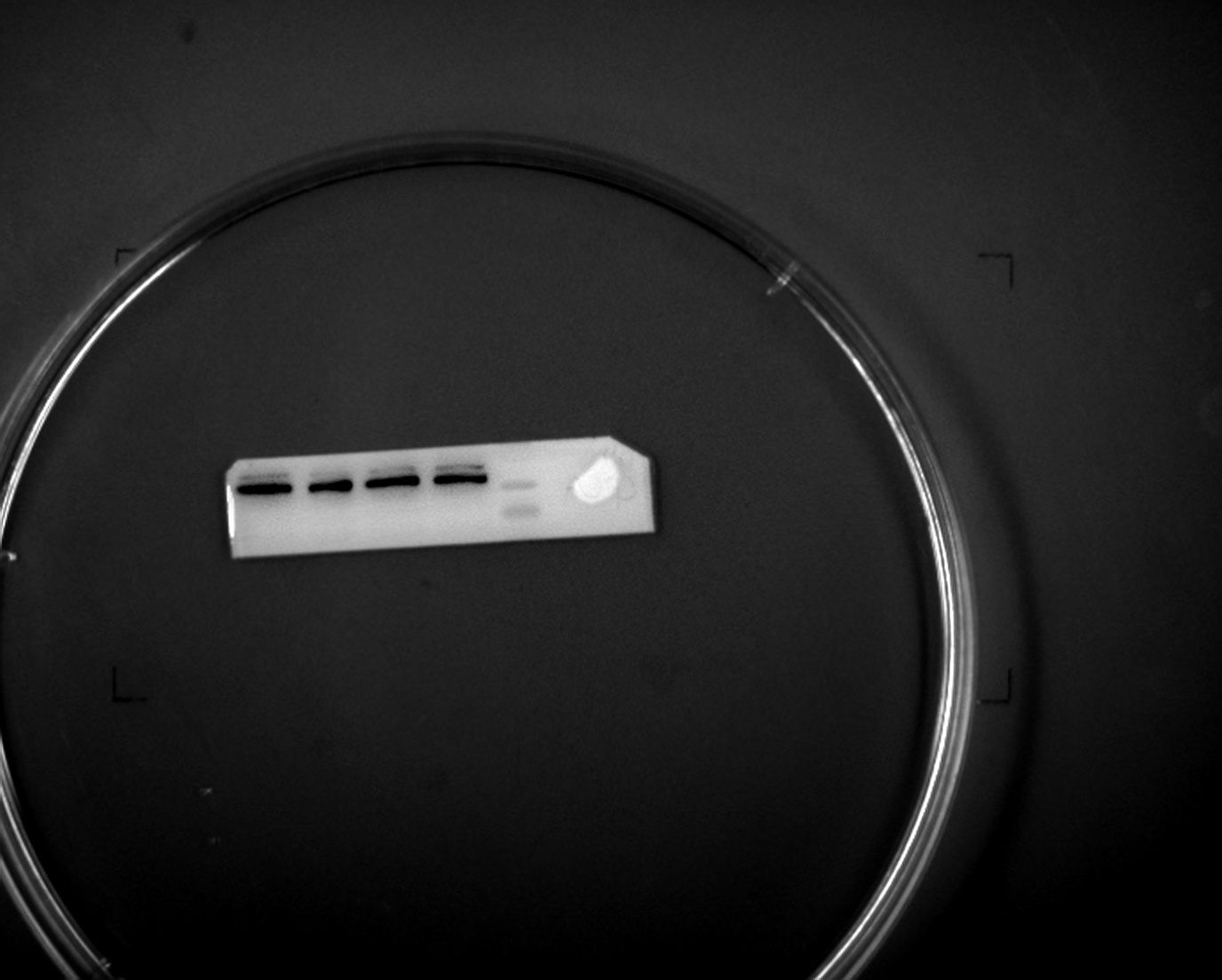

Supplement: Figure 5—source data 1. [file elife-82970-fig5-data1.zip › Figure_5-source_data_1/Figure_5-source_data_1_Figure_5E_CPLA2a┴.tif]

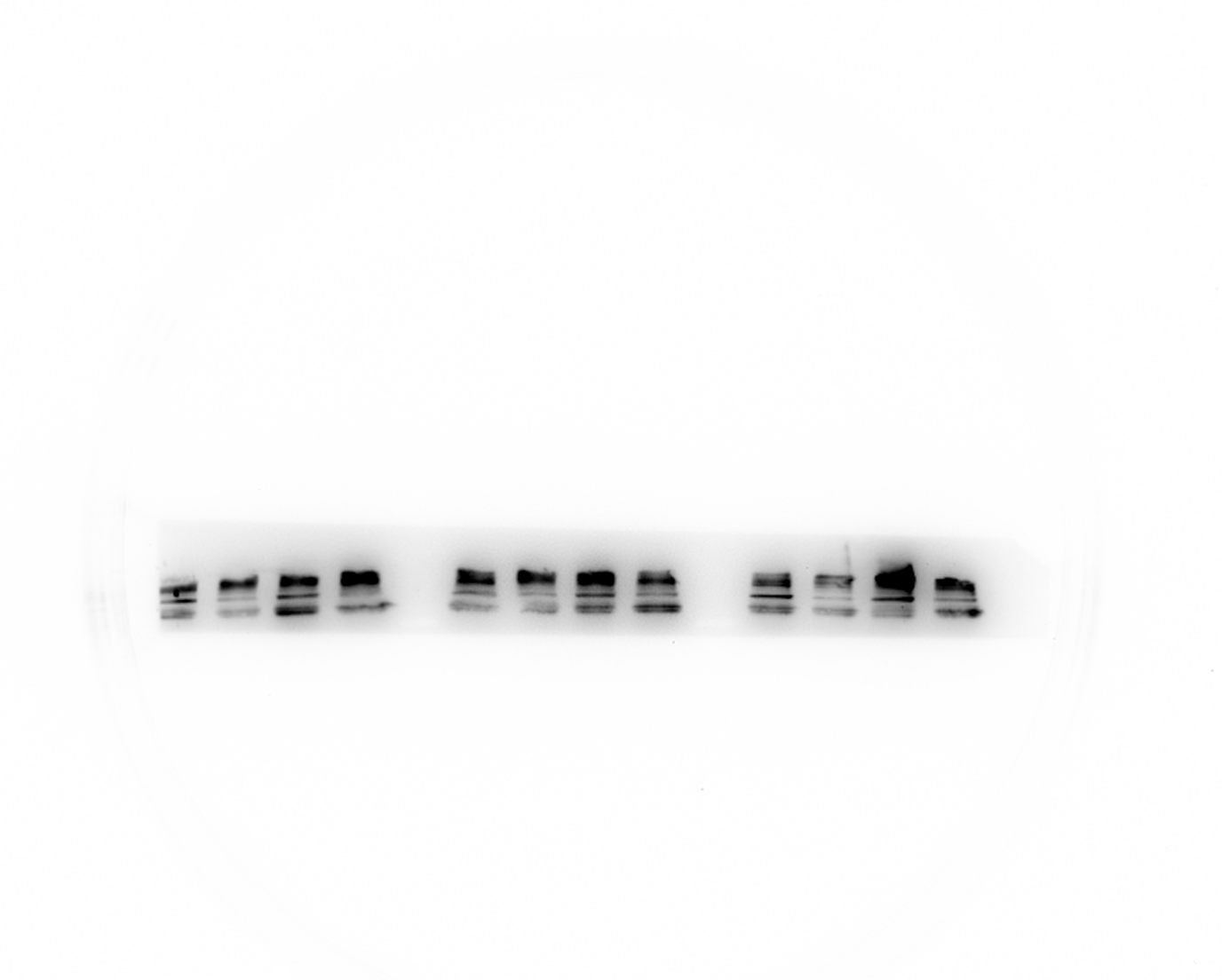

Supplement: Figure 5—source data 1. [file elife-82970-fig5-data1.zip › Figure_5-source_data_1/Figure_5-source_data_1_Figure_5E_P-CPLA2a┴.tif]

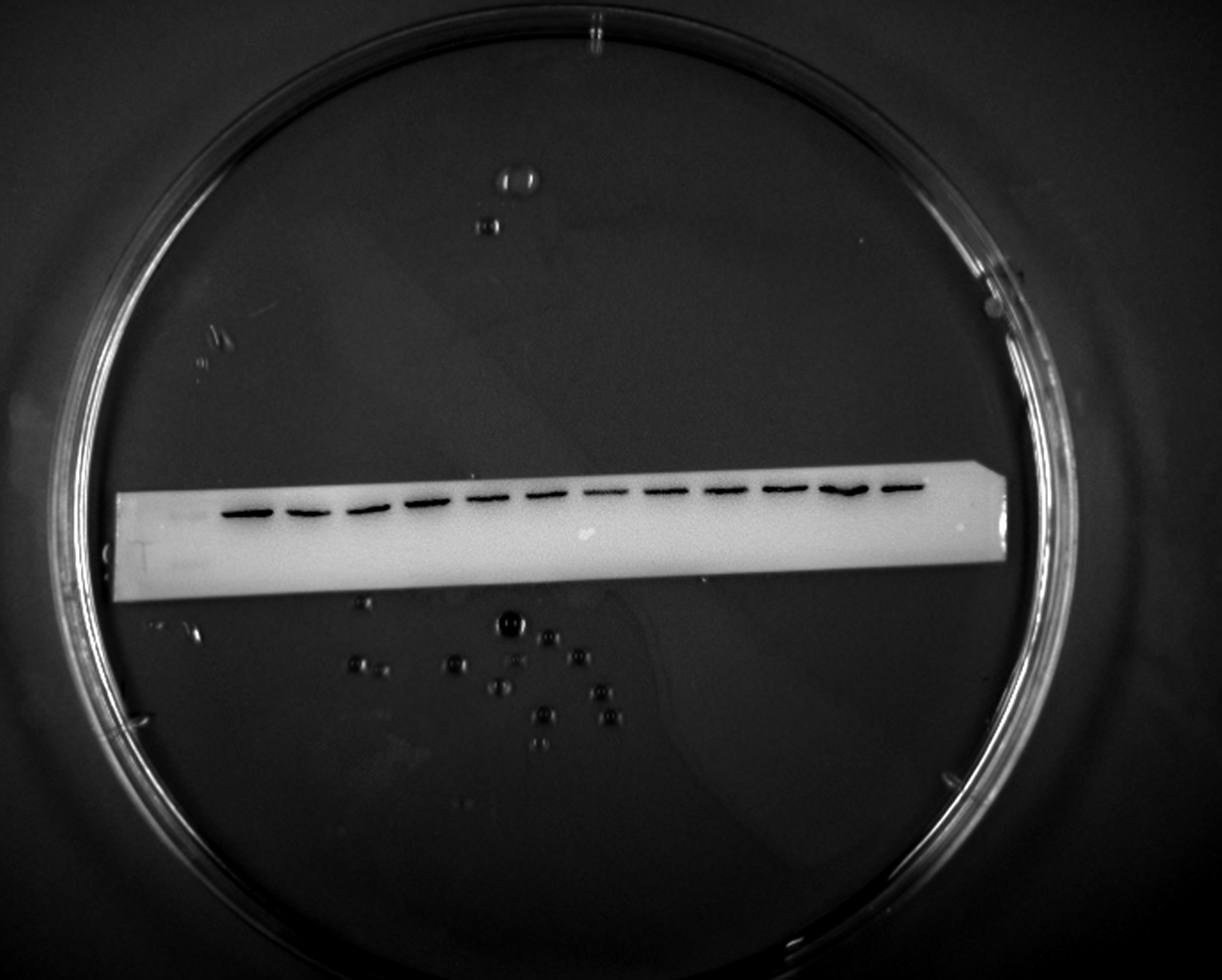

Supplement: Figure 5—source data 1. [file elife-82970-fig5-data1.zip › Figure_5-source_data_1/Figure_5-source_data_1_Figure_5E_TUBULIN.tif]

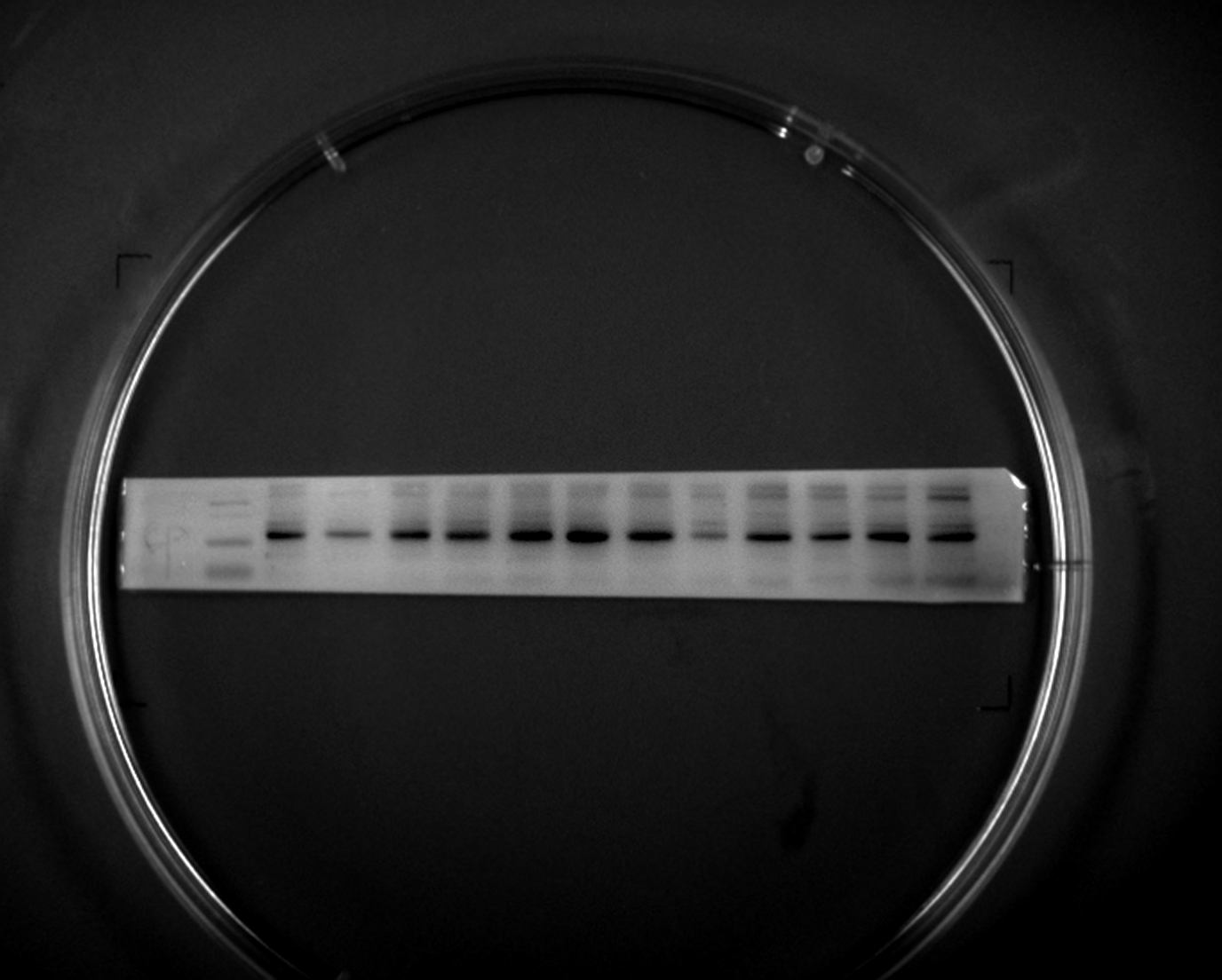

Supplement: Figure 5—source data 1. [file elife-82970-fig5-data1.zip › Figure_5-source_data_1/Figure_5-source_data_1_Figure_5F_CPLA2a┴.tif]

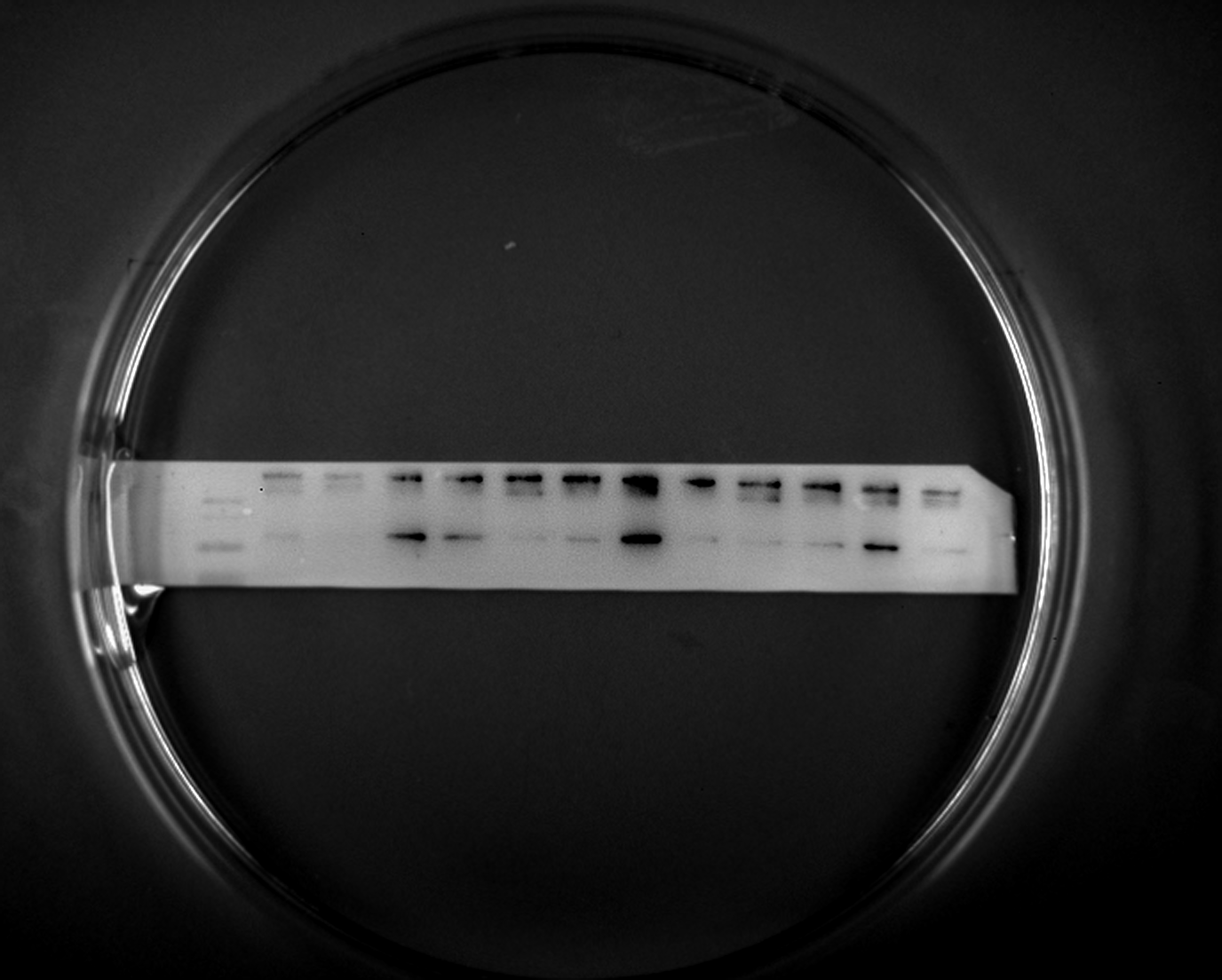

Supplement: Figure 5—source data 1. [file elife-82970-fig5-data1.zip › Figure_5-source_data_1/Figure_5-source_data_1_Figure_5F_P-CPLA2a┴.tif]

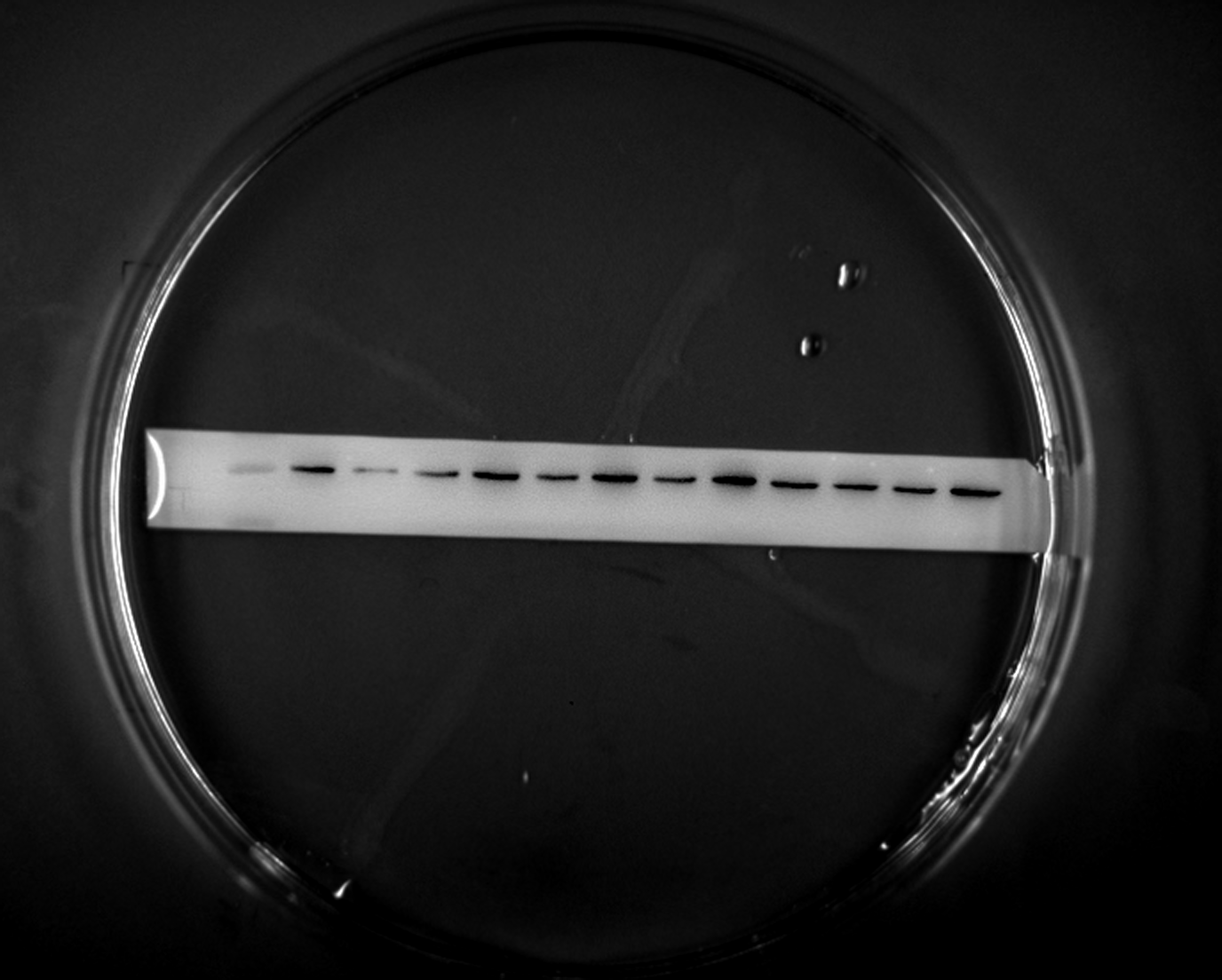

Supplement: Figure 5—source data 1. [file elife-82970-fig5-data1.zip › Figure_5-source_data_1/Figure_5-source_data_1_Figure_5F_TUBULIN.tif]

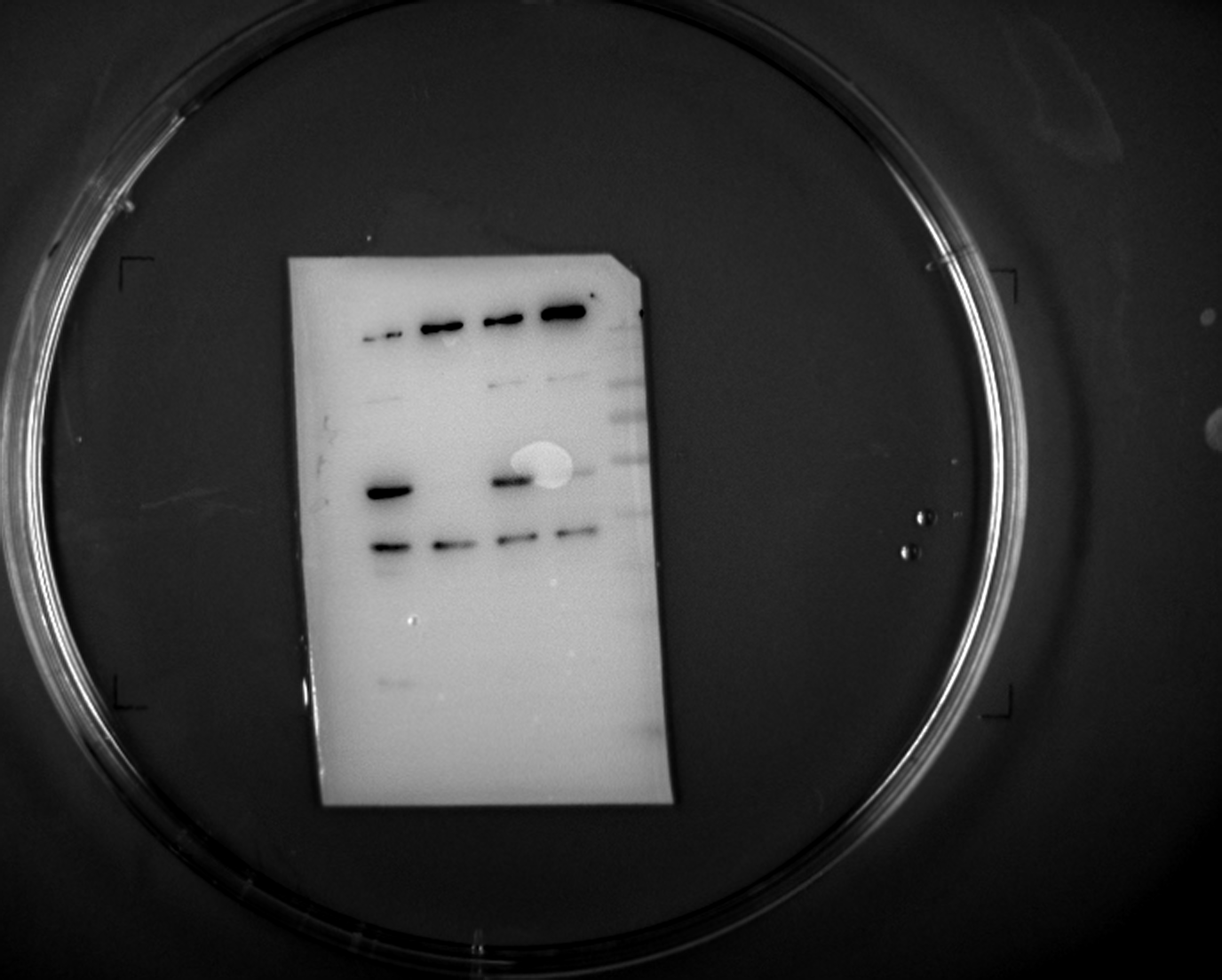

Supplement: Figure 5—source data 1. [file elife-82970-fig5-data1.zip › Figure_5-source_data_1/Figure_5-source_data_1_Figure_5H_SPARC.tif]

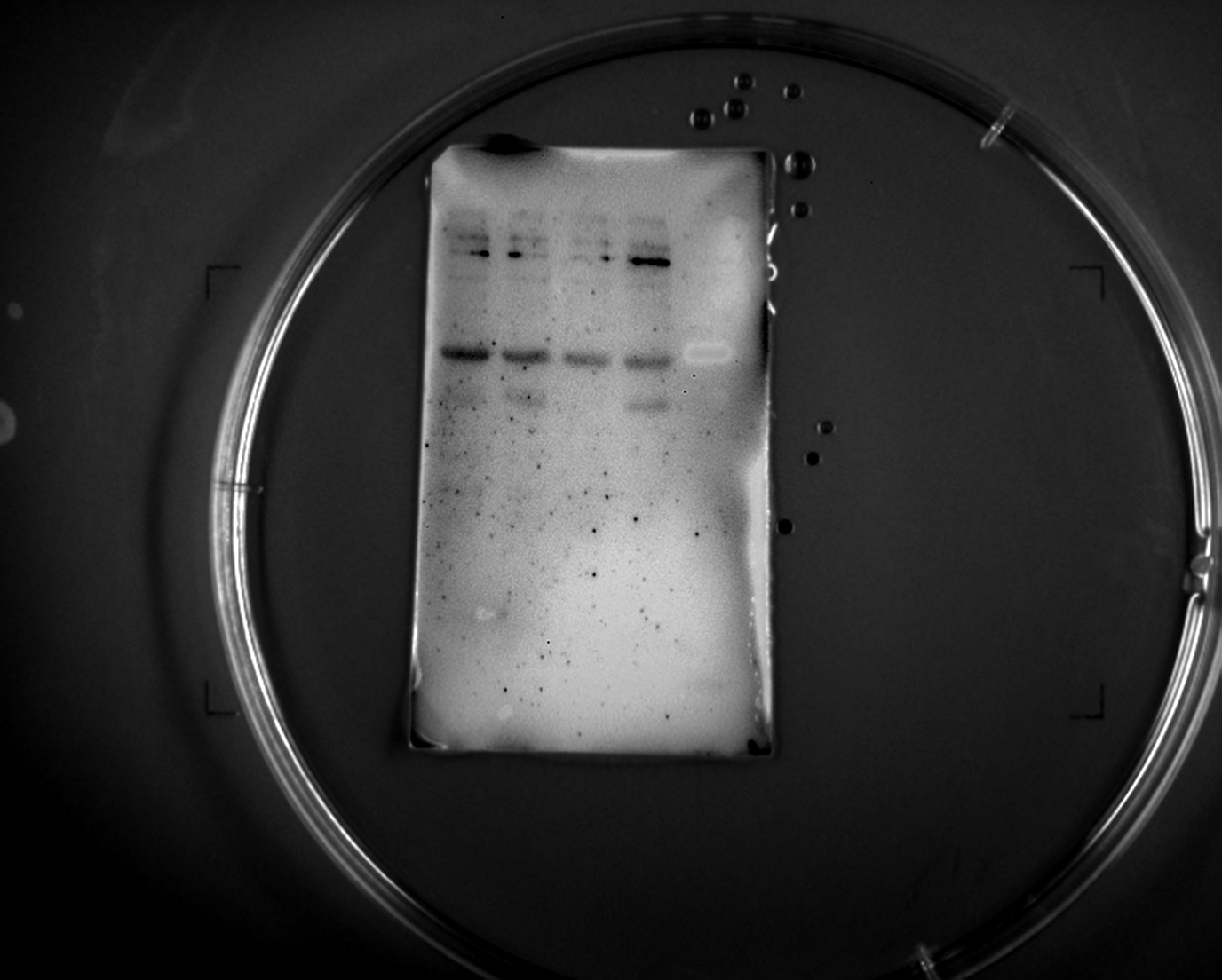

Supplement: Figure 5—source data 1. [file elife-82970-fig5-data1.zip › Figure_5-source_data_1/Figure_5-source_data_1_Figure_5H_TNC.tif]

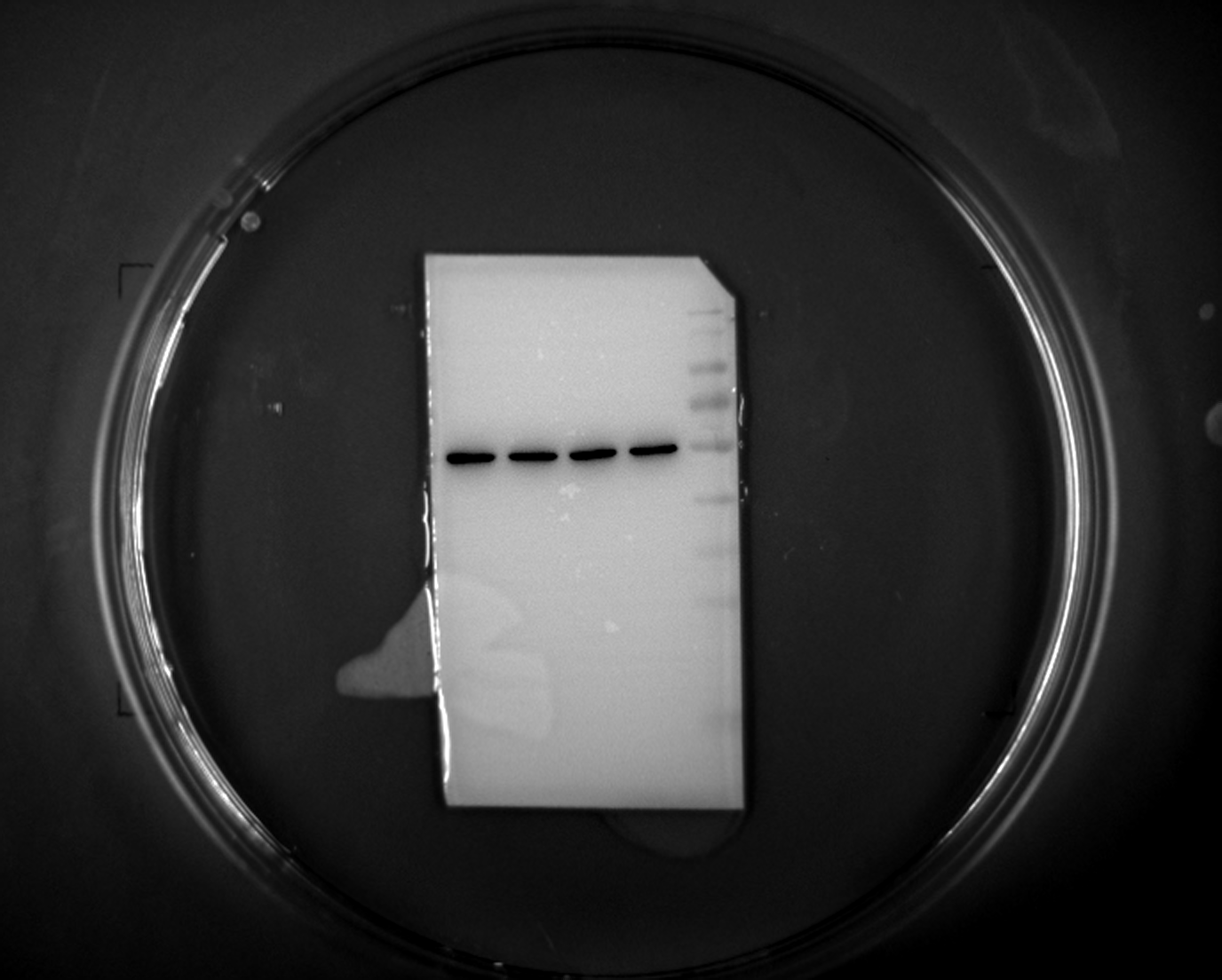

Supplement: Figure 5—source data 1. [file elife-82970-fig5-data1.zip › Figure_5-source_data_1/Figure_5-source_data_1_Figure_5H_TUBULIN.tif]

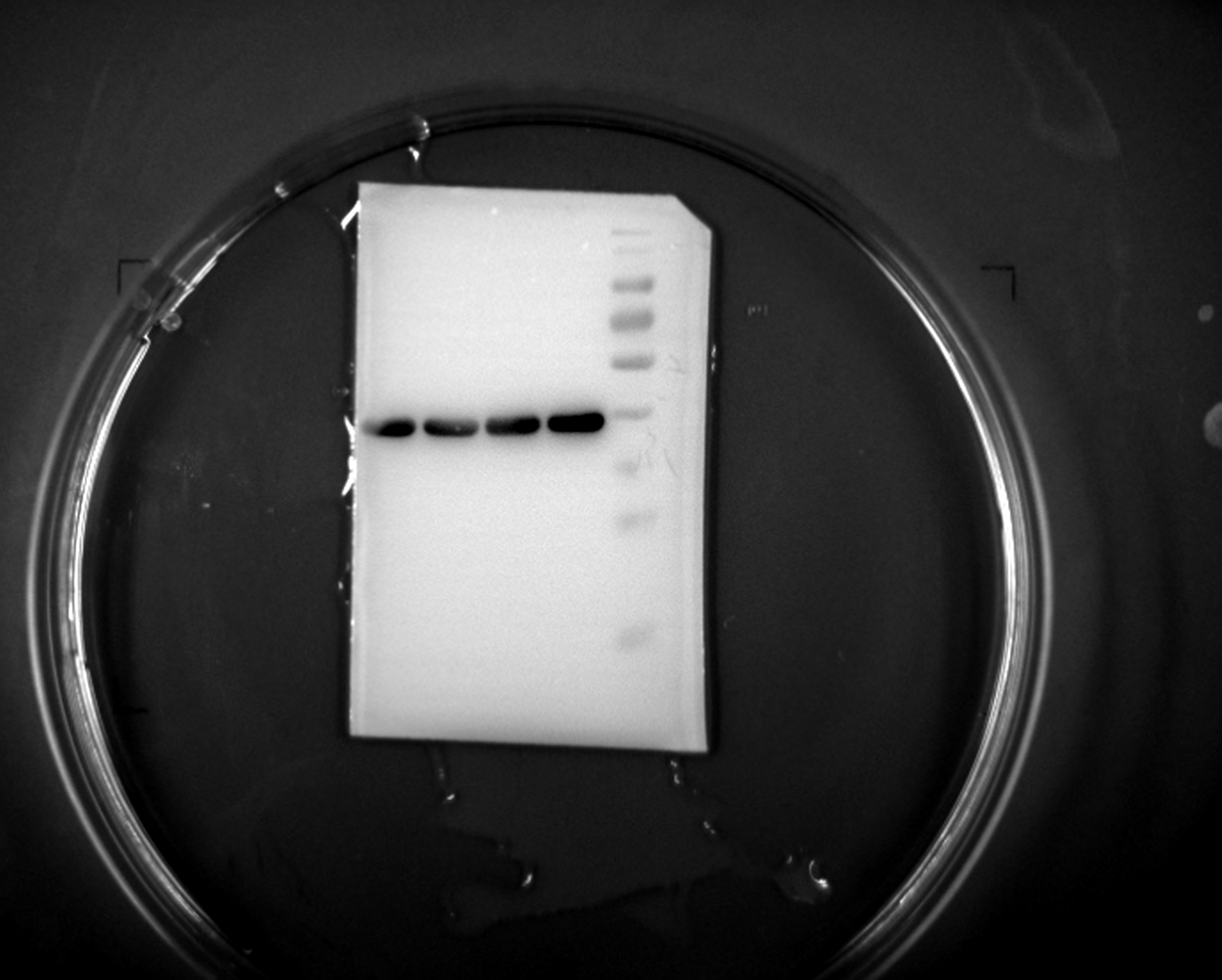

Supplement: Figure 5—source data 1. [file elife-82970-fig5-data1.zip › Figure_5-source_data_1/Figure_5-source_data_1_Figure_5H_a┴-SMA.tif]

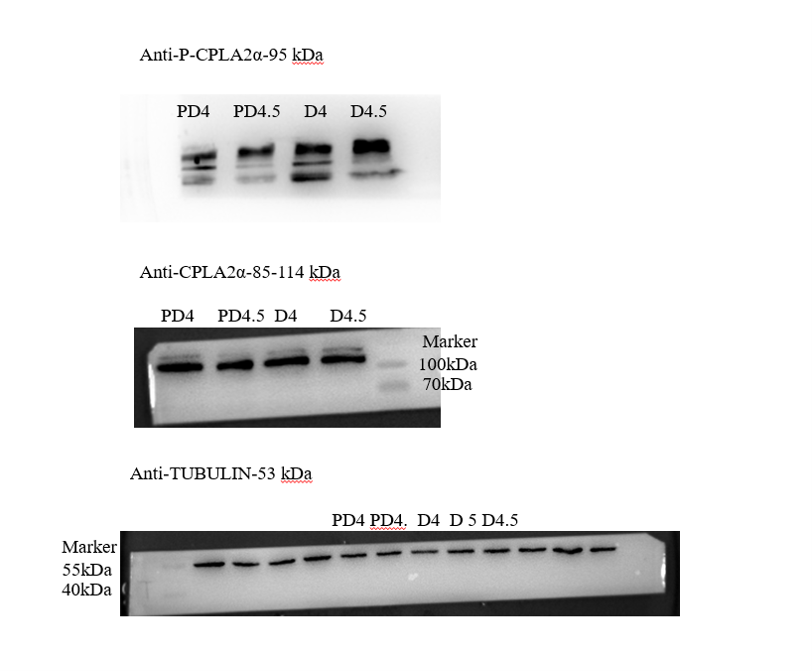

Supplement: Figure 5—source data 2. [file elife-82970-fig5-data2.zip › Figure_5-source_data_2/Figure_5-source_data_2-5E.png]

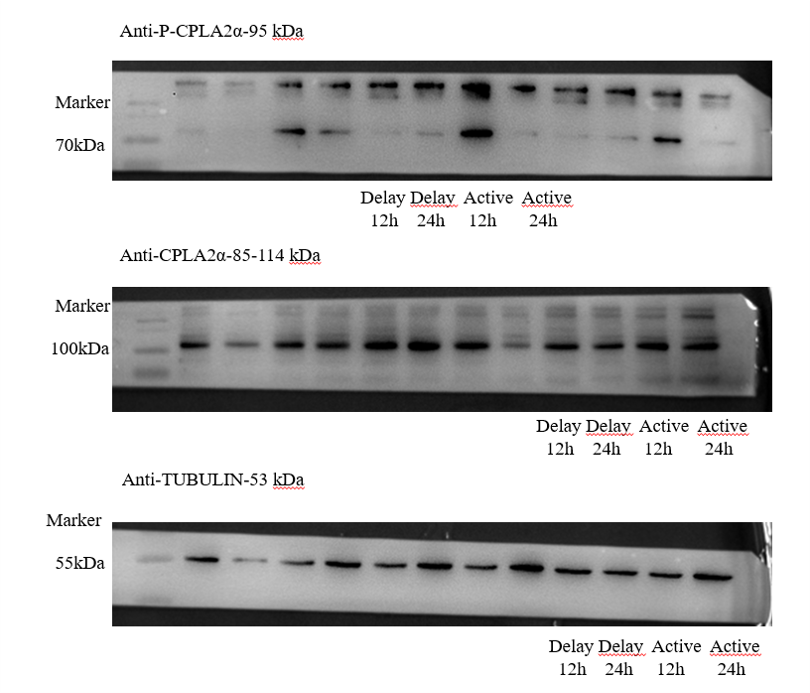

Supplement: Figure 5—source data 2. [file elife-82970-fig5-data2.zip › Figure_5-source_data_2/Figure_5-source_data_2-5F.png]

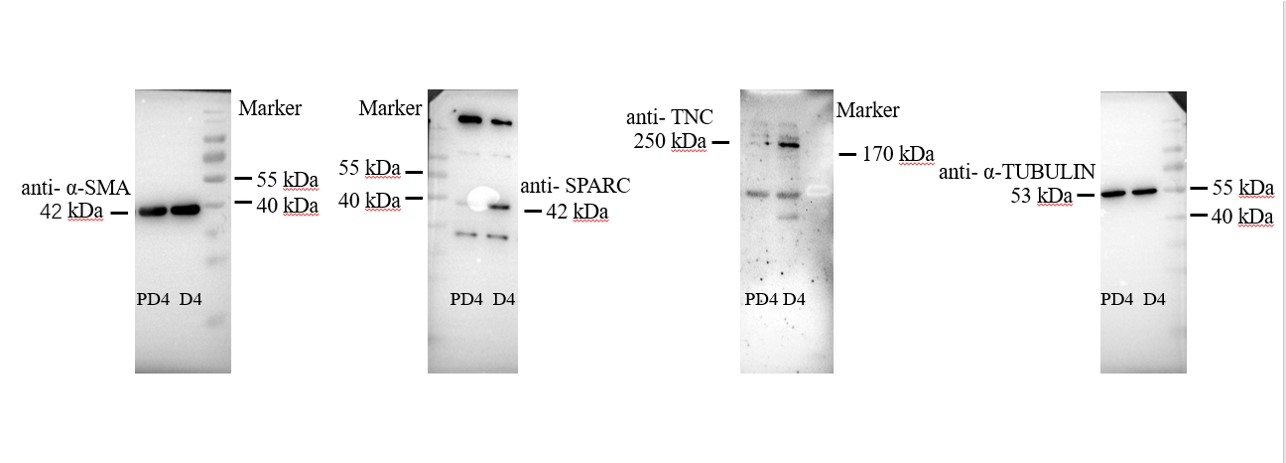

Supplement: Figure 5—source data 2. [file elife-82970-fig5-data2.zip › Figure_5-source_data_2/Figure_5-source_data_2-5H.png]

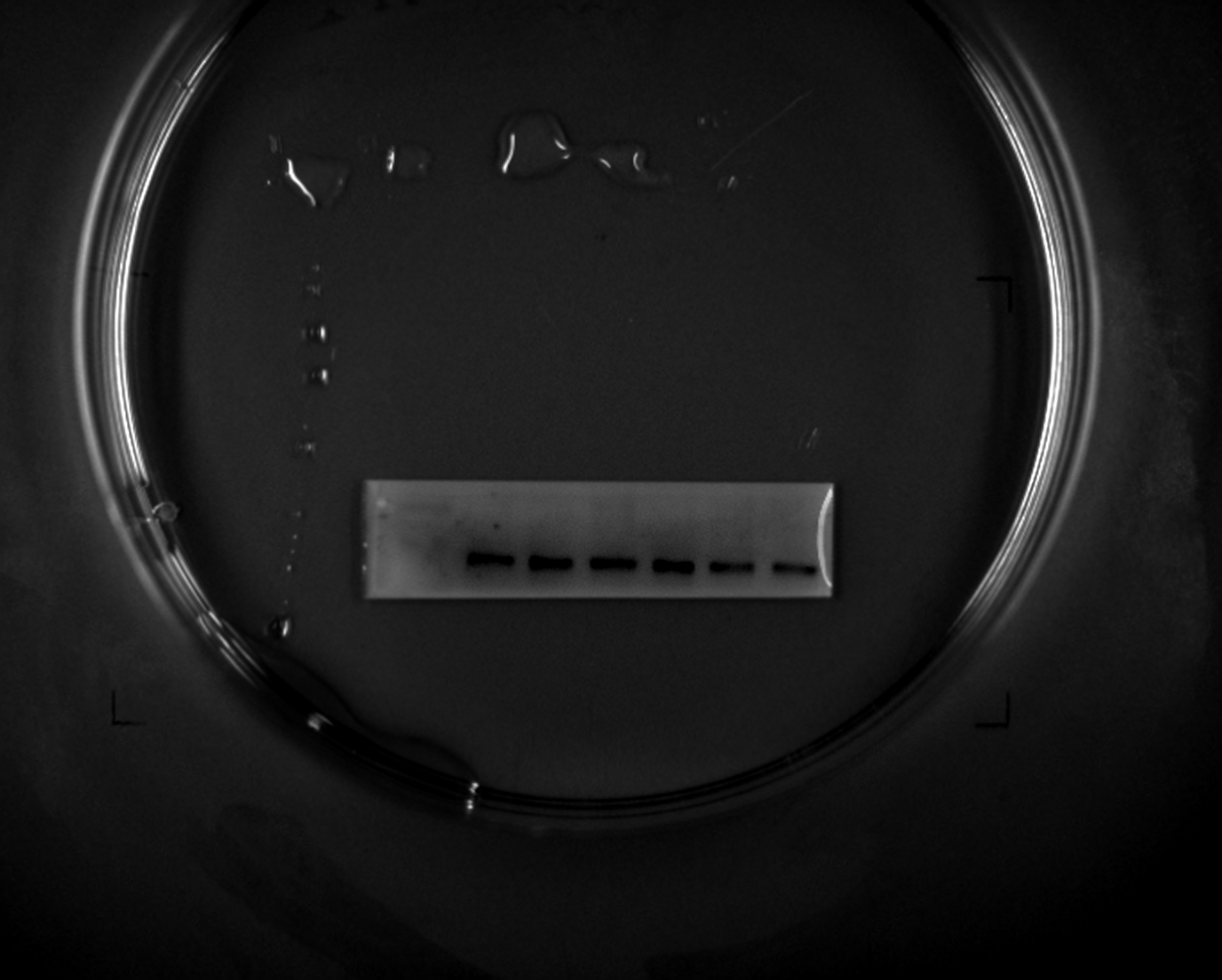

Supplement: Figure 6—source data 1. [file elife-82970-fig6-data1.zip › Figure_6-source_data_1/Figure_6-source_data_1_Figure_6E_CPLA2a┴.tif]

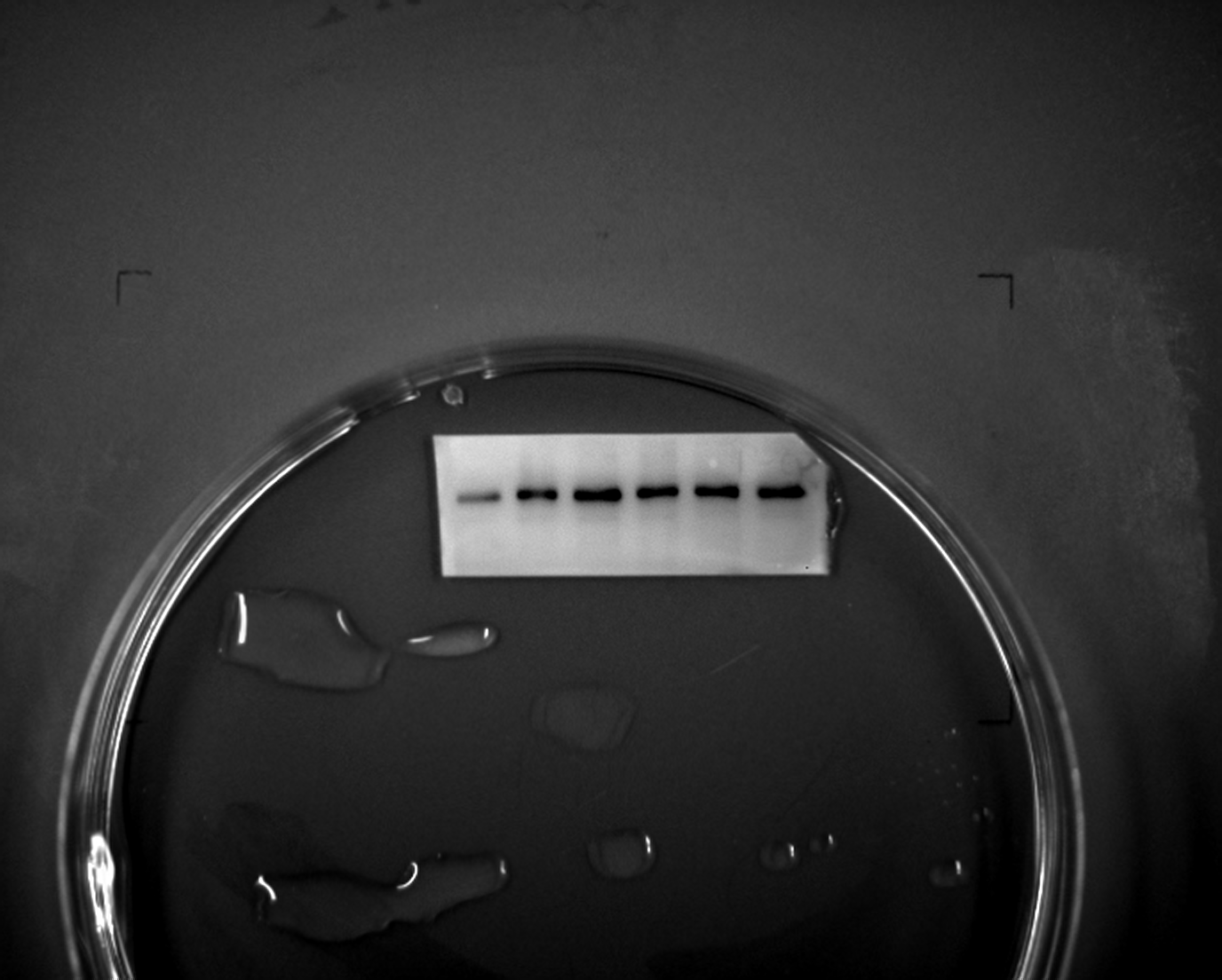

Supplement: Figure 6—source data 1. [file elife-82970-fig6-data1.zip › Figure_6-source_data_1/Figure_6-source_data_1_Figure_6E_P-CPLA2a┴.tif]

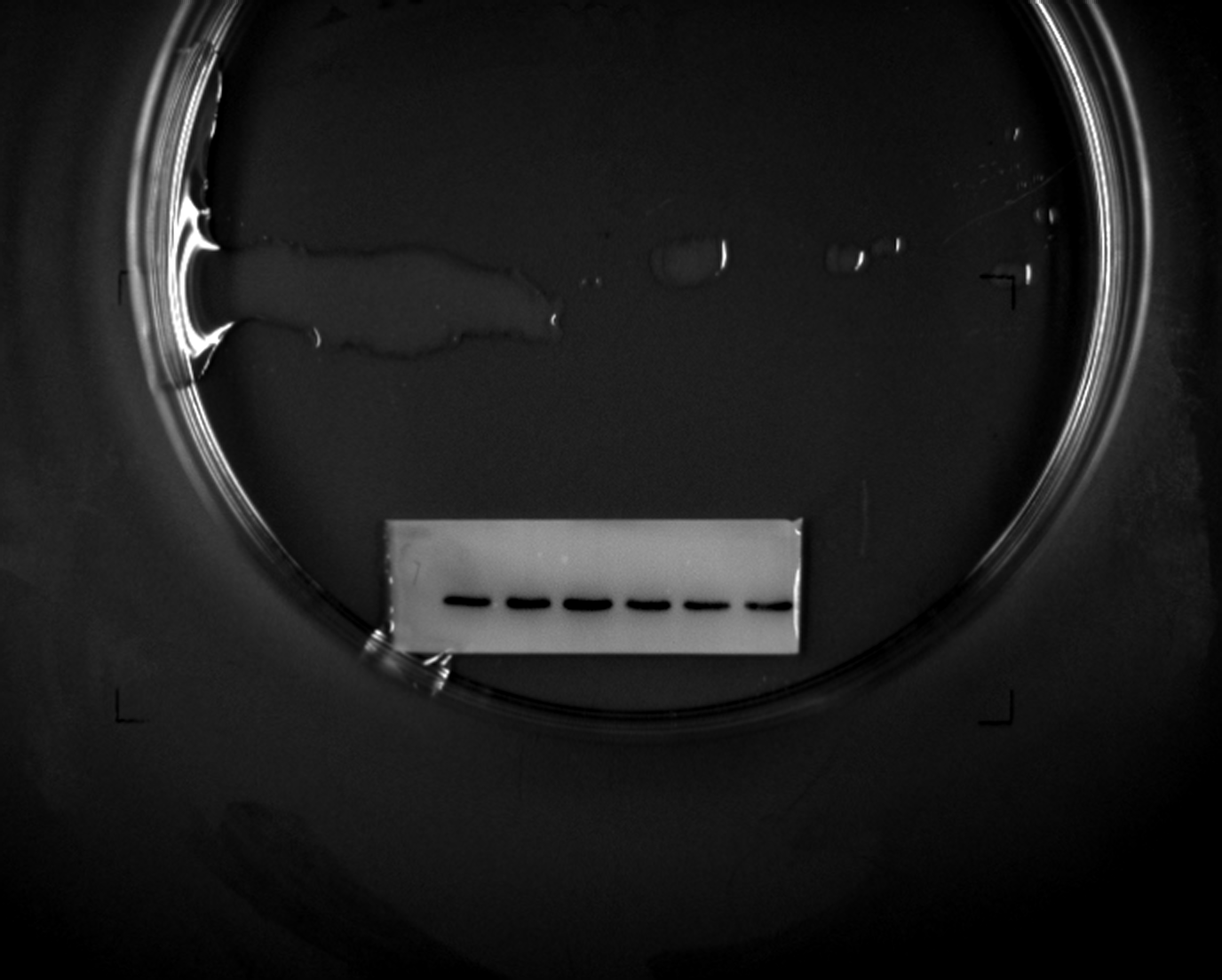

Supplement: Figure 6—source data 1. [file elife-82970-fig6-data1.zip › Figure_6-source_data_1/Figure_6-source_data_1_Figure_6E_TUBULIN.tif]

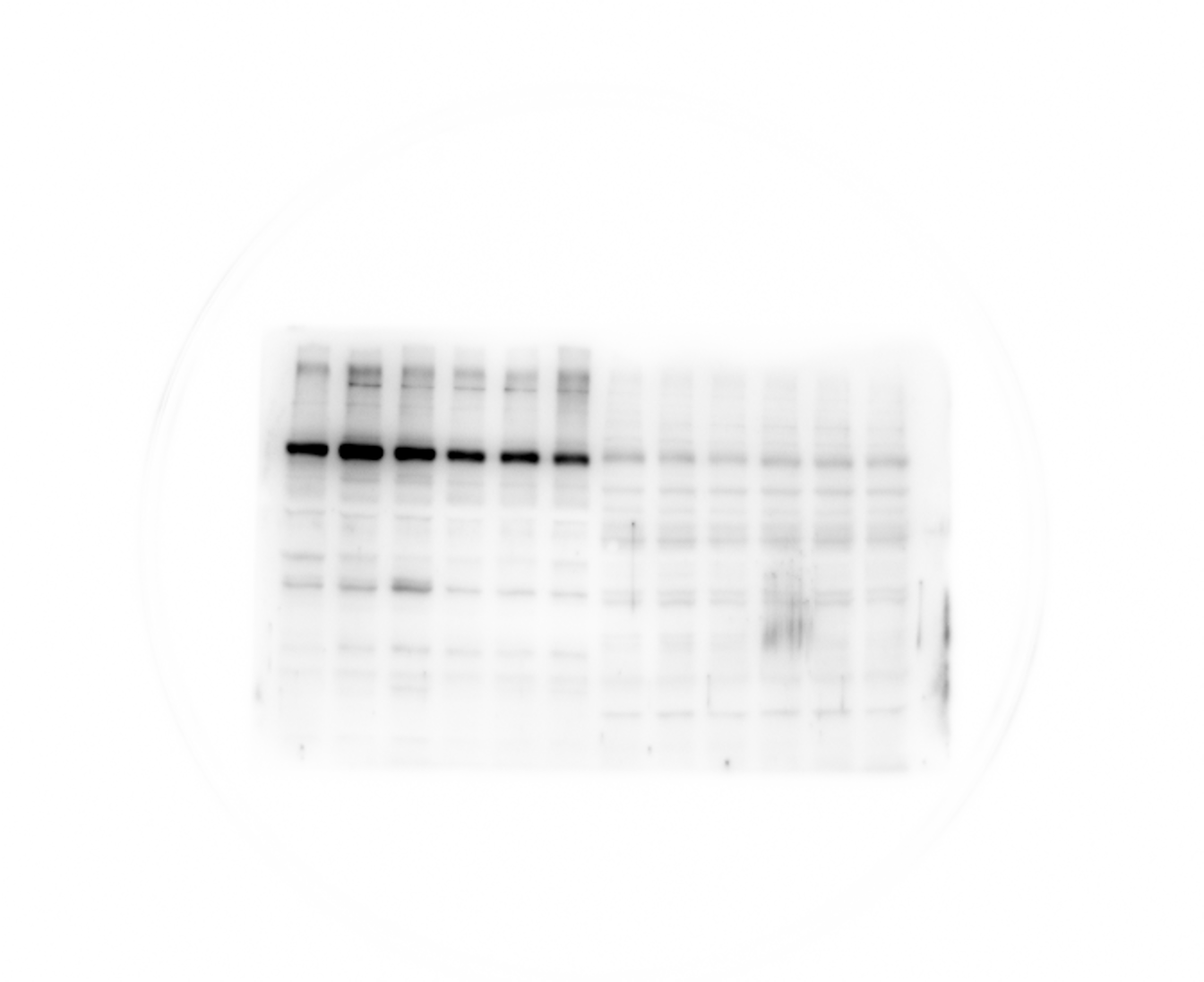

Supplement: Figure 6—source data 1. [file elife-82970-fig6-data1.zip › Figure_6-source_data_1/Figure_6-source_data_1_Figure_6F_CPLA2a┴.Tif]

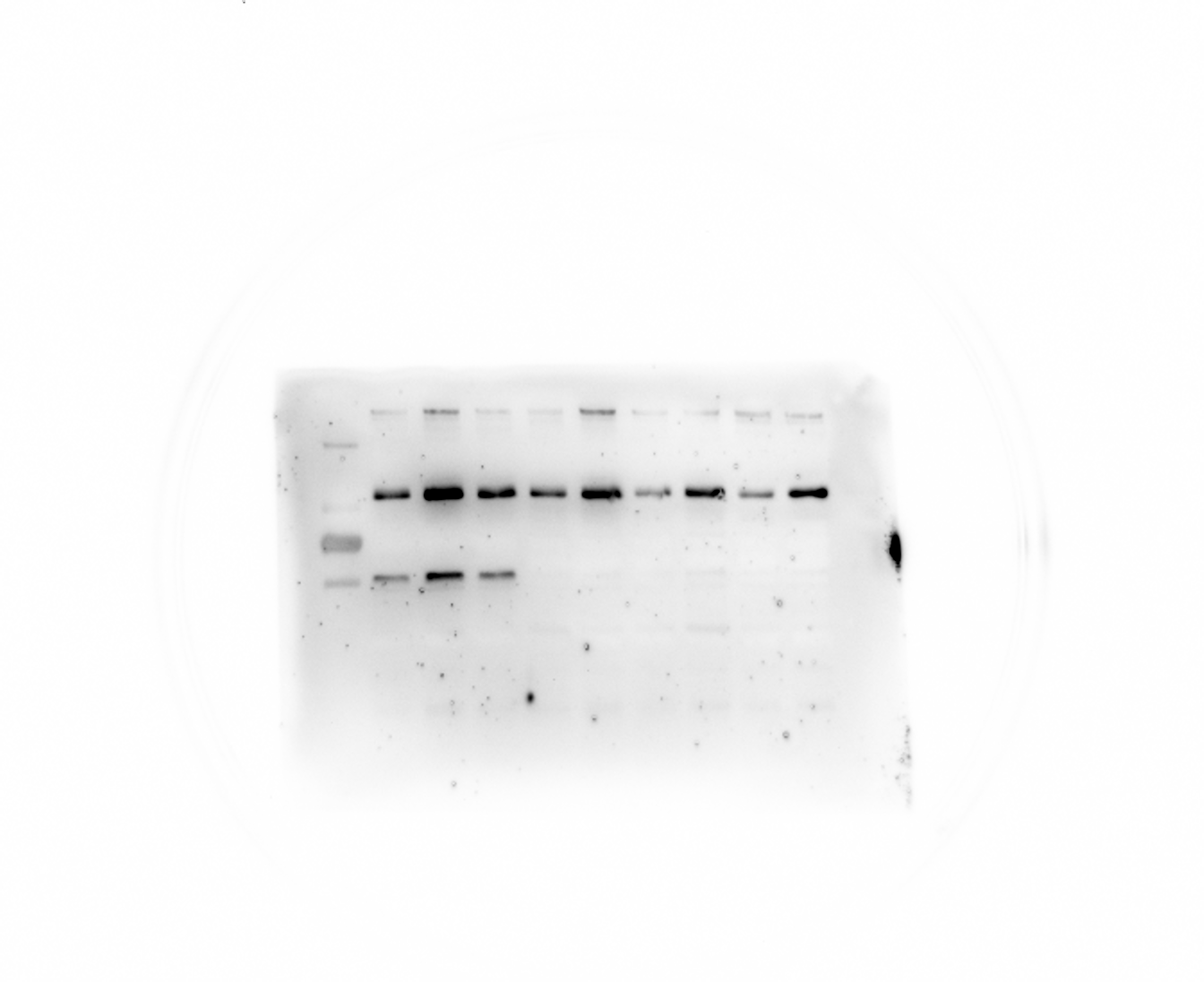

Supplement: Figure 6—source data 1. [file elife-82970-fig6-data1.zip › Figure_6-source_data_1/Figure_6-source_data_1_Figure_6F_P-CPLA2a┴.Tif]

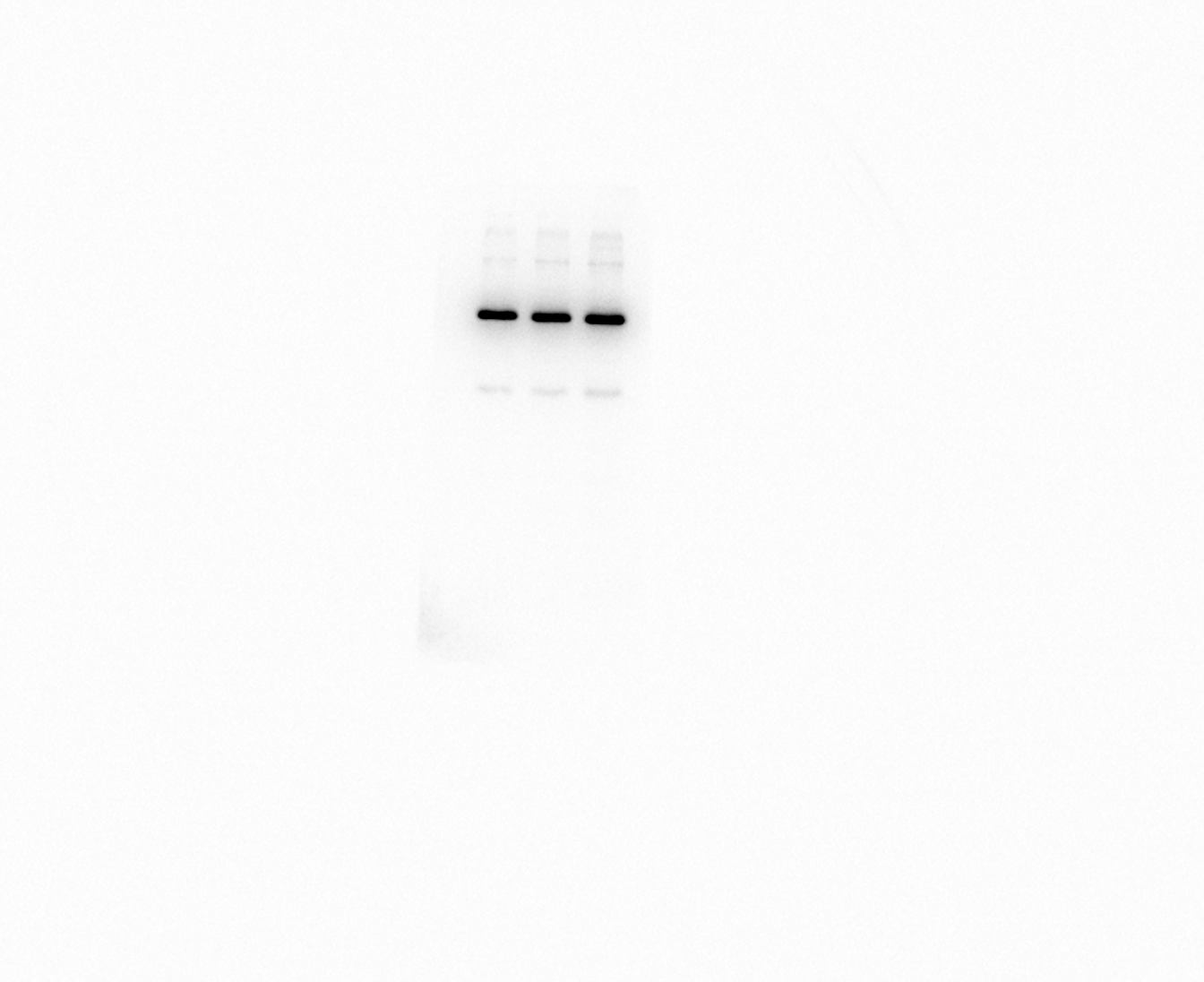

Supplement: Figure 6—source data 1. [file elife-82970-fig6-data1.zip › Figure_6-source_data_1/Figure_6-source_data_1_Figure_6F_TUBULIN.Tif]

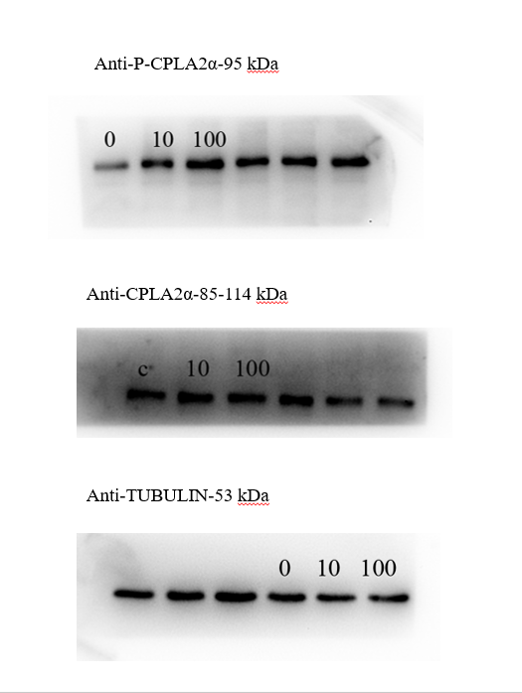

Supplement: Figure 6—source data 2. [file elife-82970-fig6-data2.zip › Figure_6-source_data_2/Figure_6-source_data_2-6E.png]

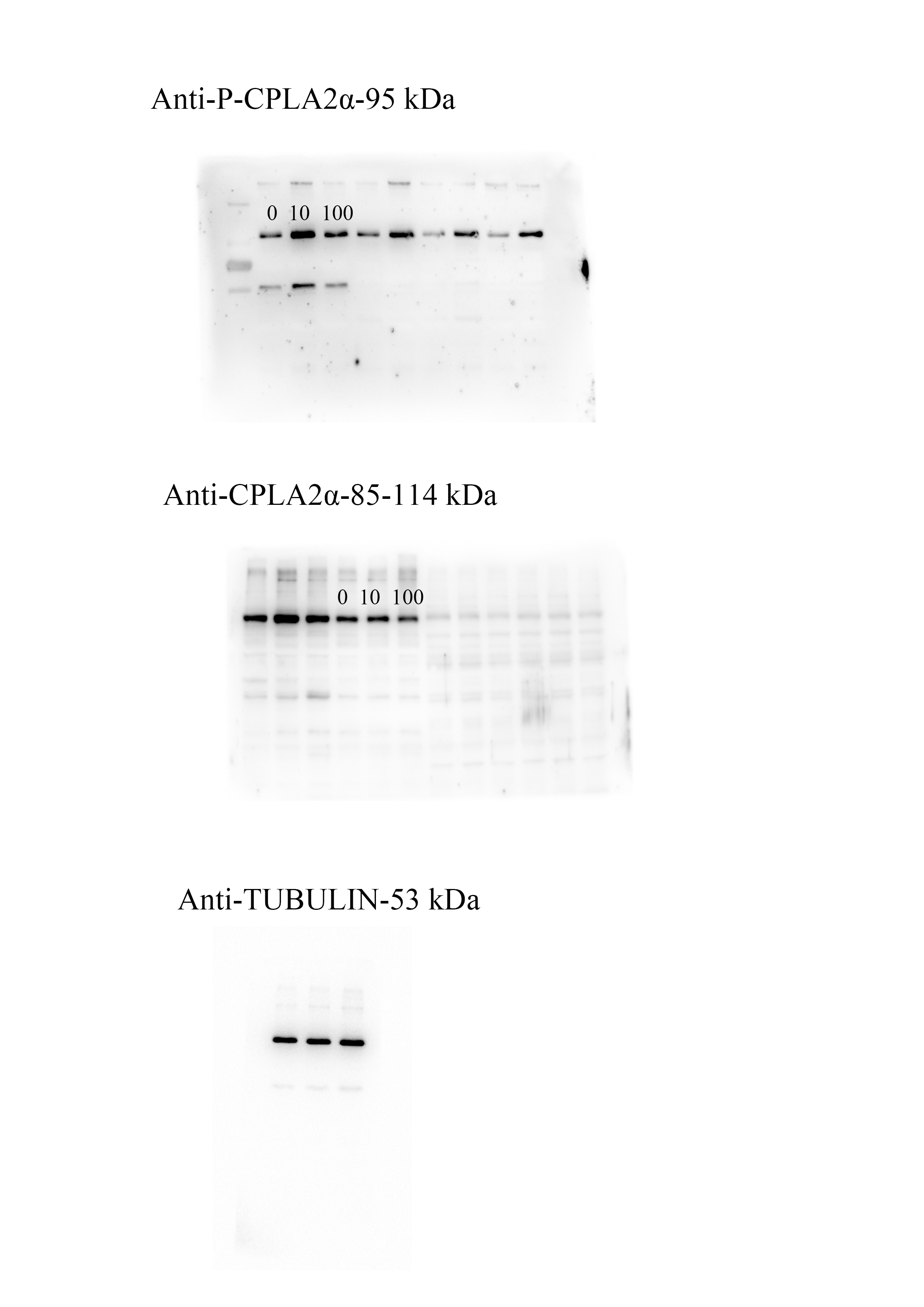

Supplement: Figure 6—source data 2. [file elife-82970-fig6-data2.zip › Figure_6-source_data_2/Figure_6-source_data_2-6F.jpg]

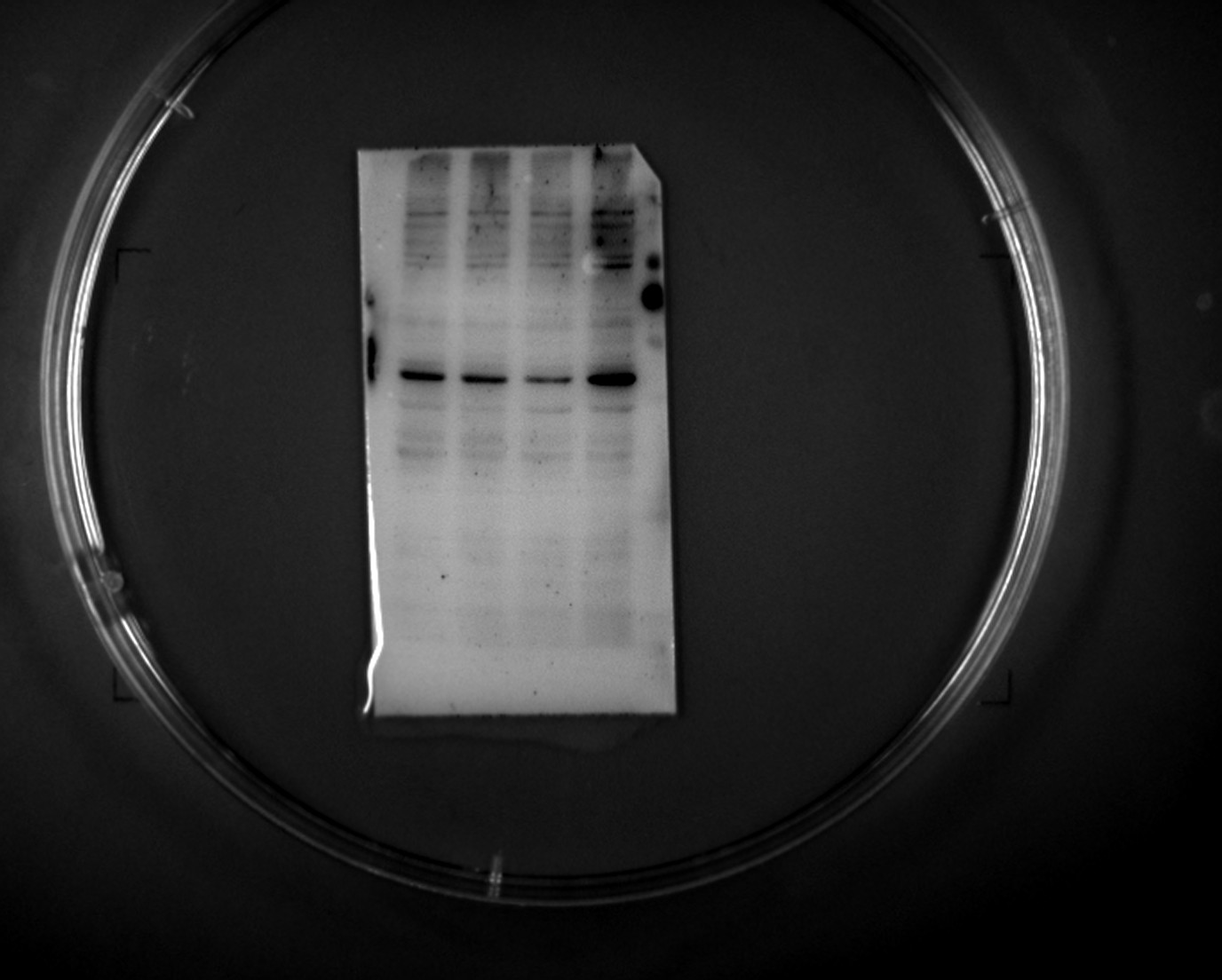

Supplement: Figure 7—source data 1. [file elife-82970-fig7-data1.zip › Figure_7-source_data_1/Figure_7-source_data_1_Figure_7A_ACTIVIN A.jpg]

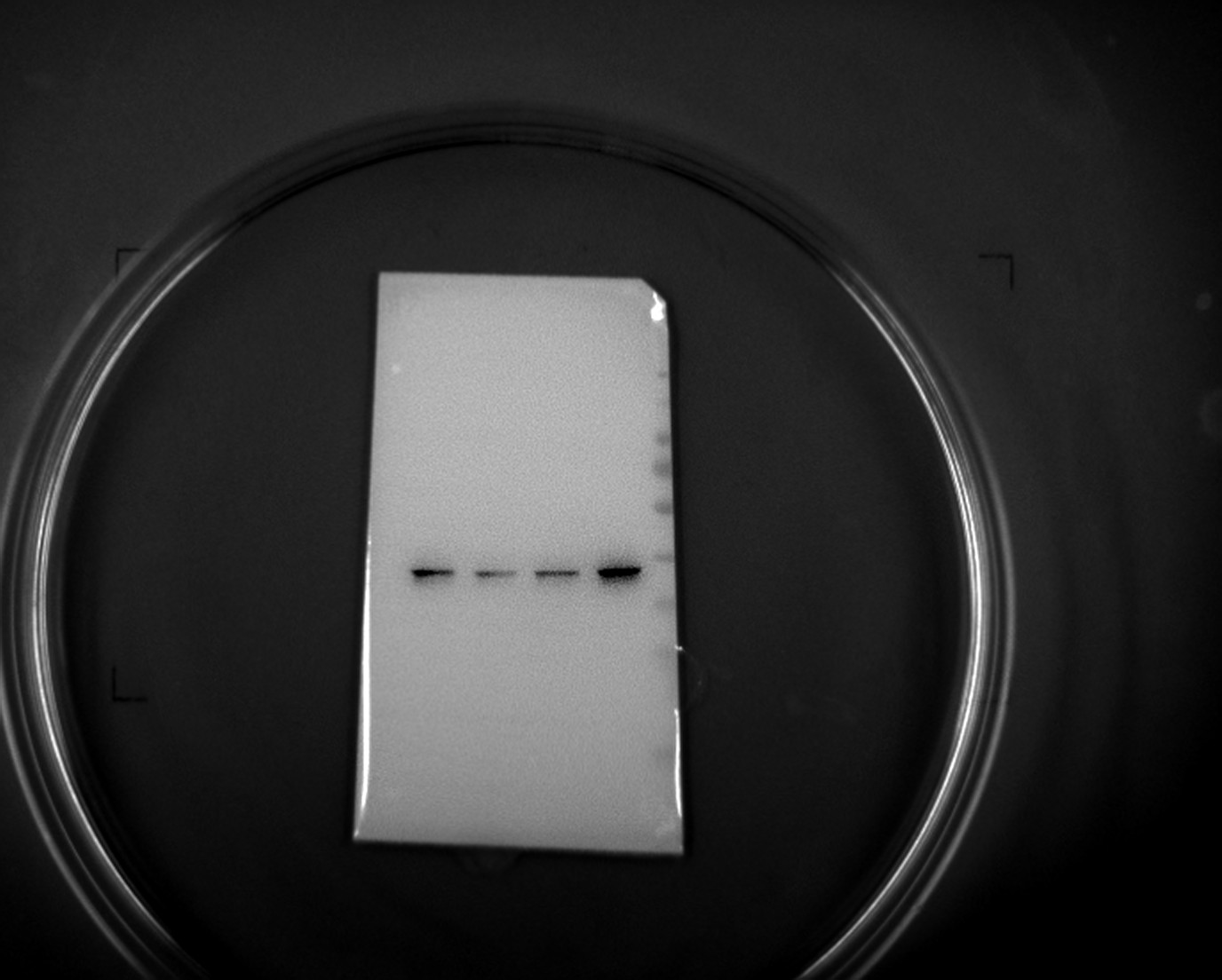

Supplement: Figure 7—source data 1. [file elife-82970-fig7-data1.zip › Figure_7-source_data_1/Figure_7-source_data_1_Figure_7A_SPARC.jpg]

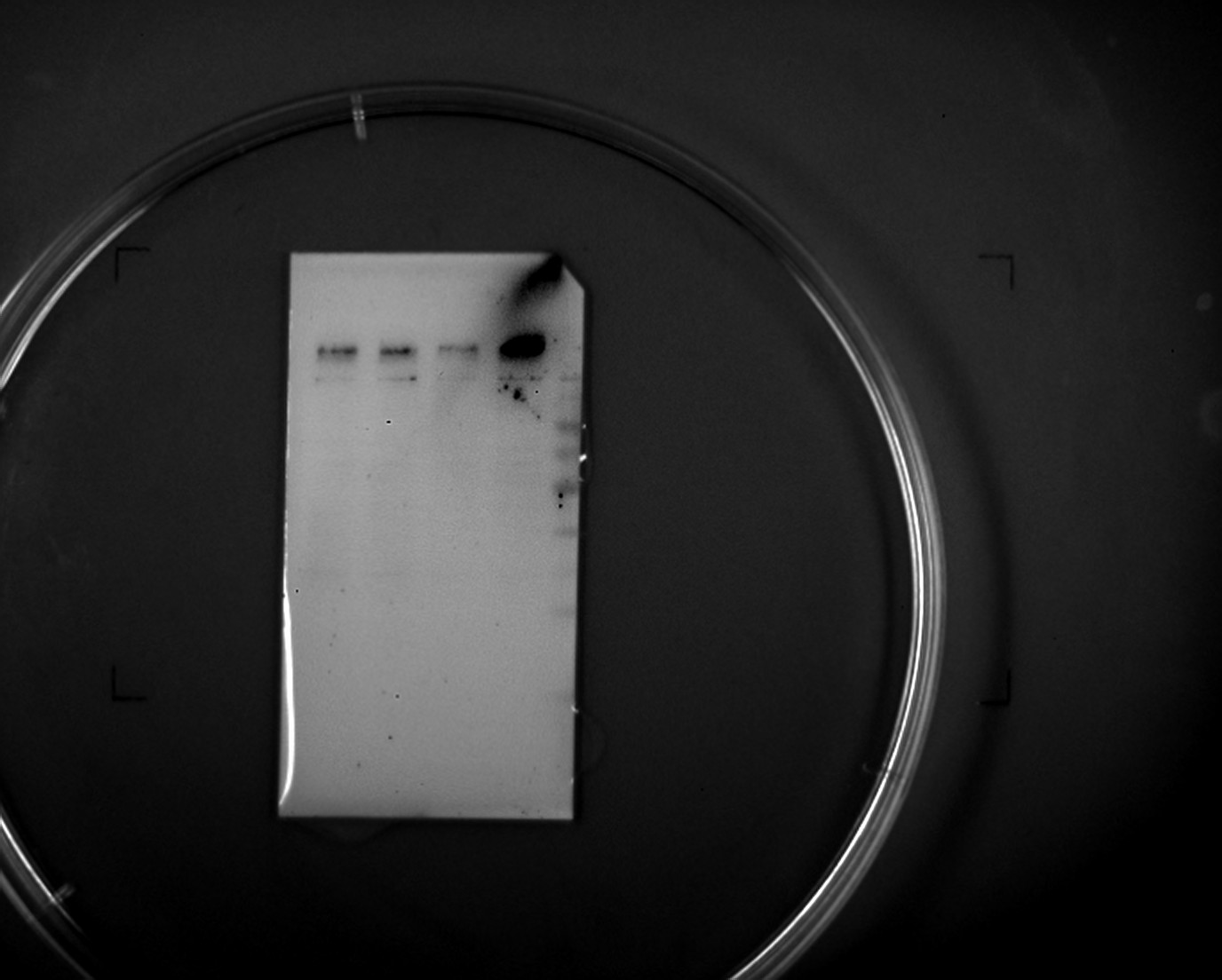

Supplement: Figure 7—source data 1. [file elife-82970-fig7-data1.zip › Figure_7-source_data_1/Figure_7-source_data_1_Figure_7A_TNC.jpg]

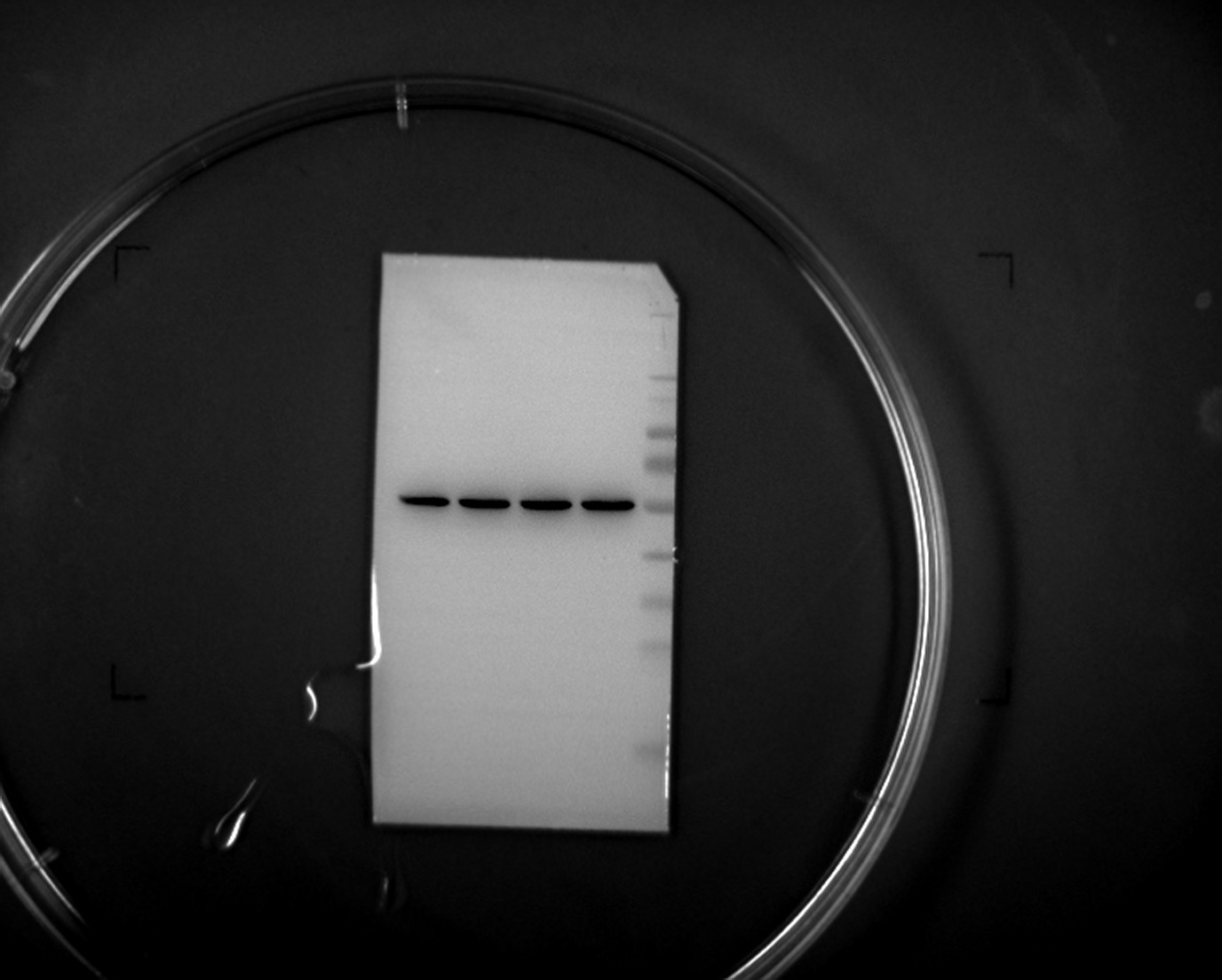

Supplement: Figure 7—source data 1. [file elife-82970-fig7-data1.zip › Figure_7-source_data_1/Figure_7-source_data_1_Figure_7A_TUBULIN.jpg]

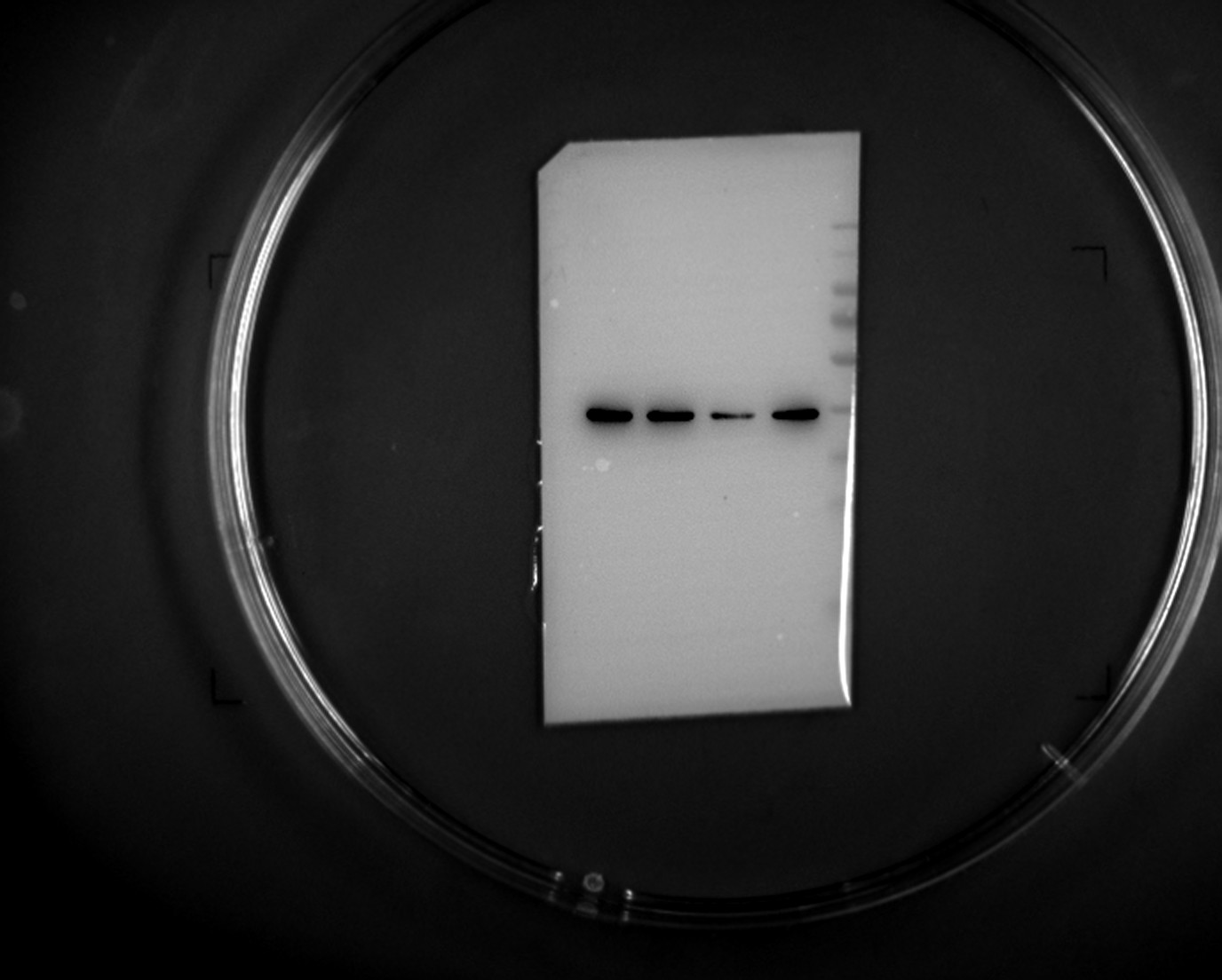

Supplement: Figure 7—source data 1. [file elife-82970-fig7-data1.zip › Figure_7-source_data_1/Figure_7-source_data_1_Figure_7A_a┴-SMA.jpg]

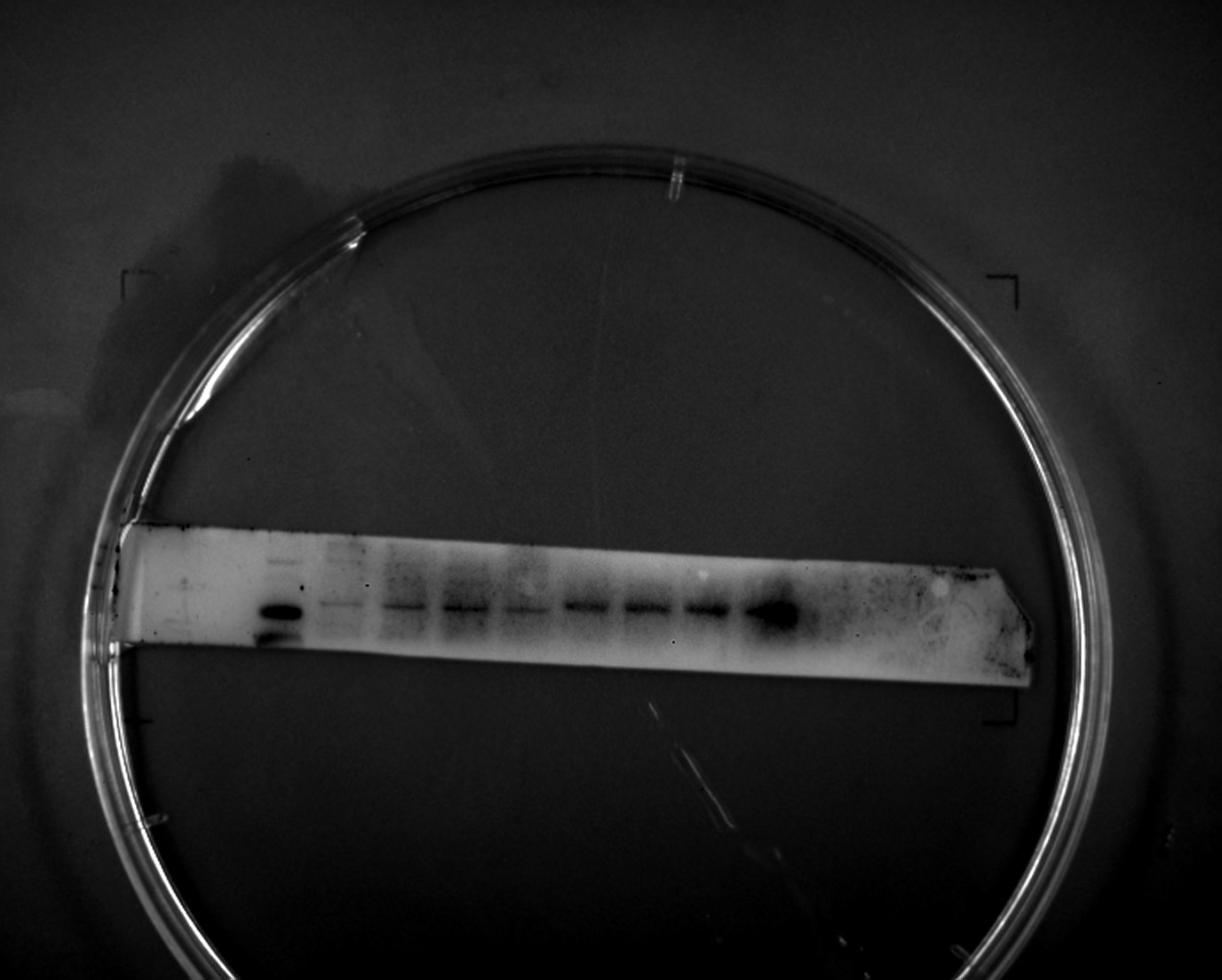

Supplement: Figure 7—source data 1. [file elife-82970-fig7-data1.zip › Figure_7-source_data_1/Figure_7-source_data_1_Figure_7B_CPLA2a┴.jpg]

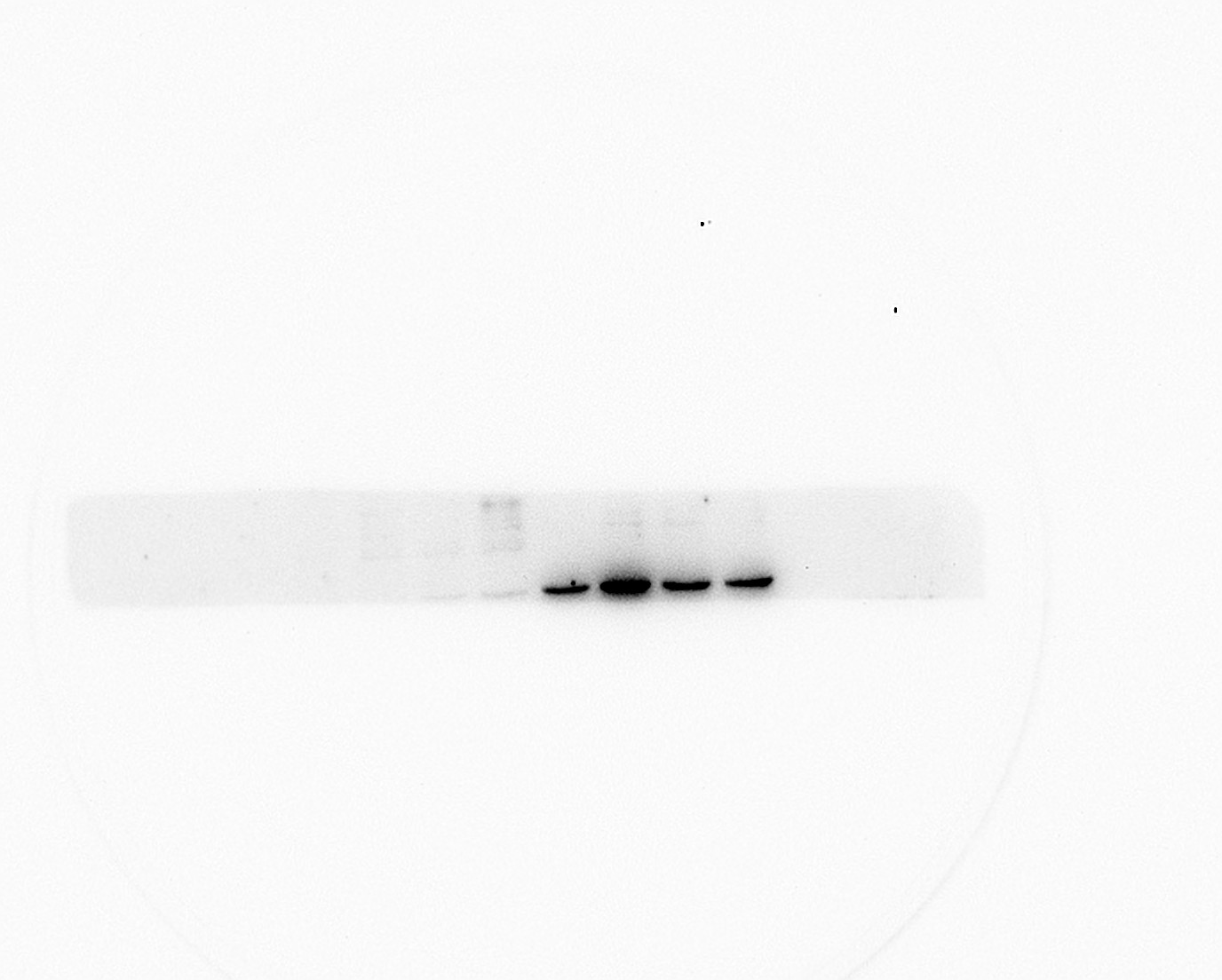

Supplement: Figure 7—source data 1. [file elife-82970-fig7-data1.zip › Figure_7-source_data_1/Figure_7-source_data_1_Figure_7B_P-CPLA2a┴.jpg]

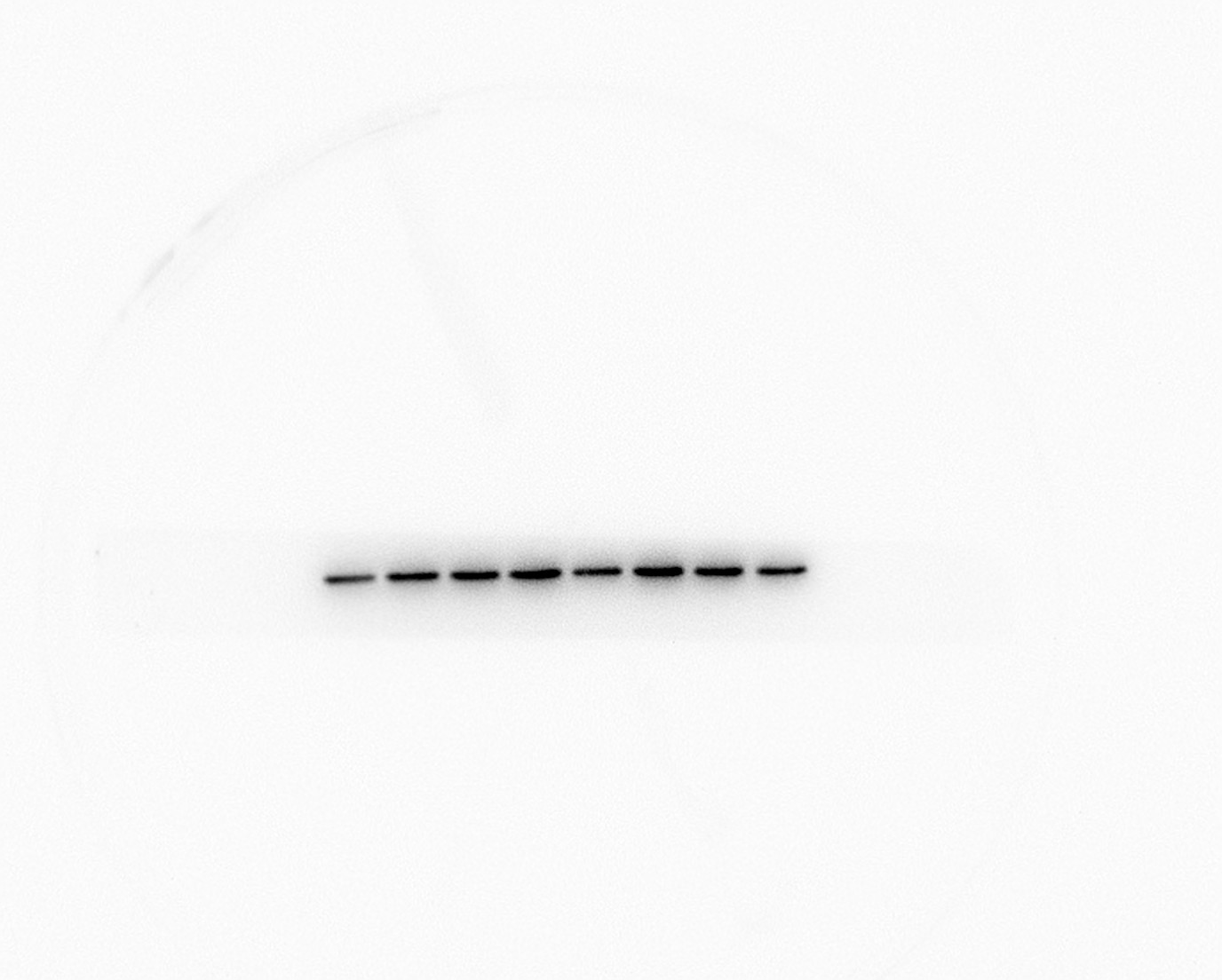

Supplement: Figure 7—source data 1. [file elife-82970-fig7-data1.zip › Figure_7-source_data_1/Figure_7-source_data_1_Figure_7B_TUBULIN.jpg]

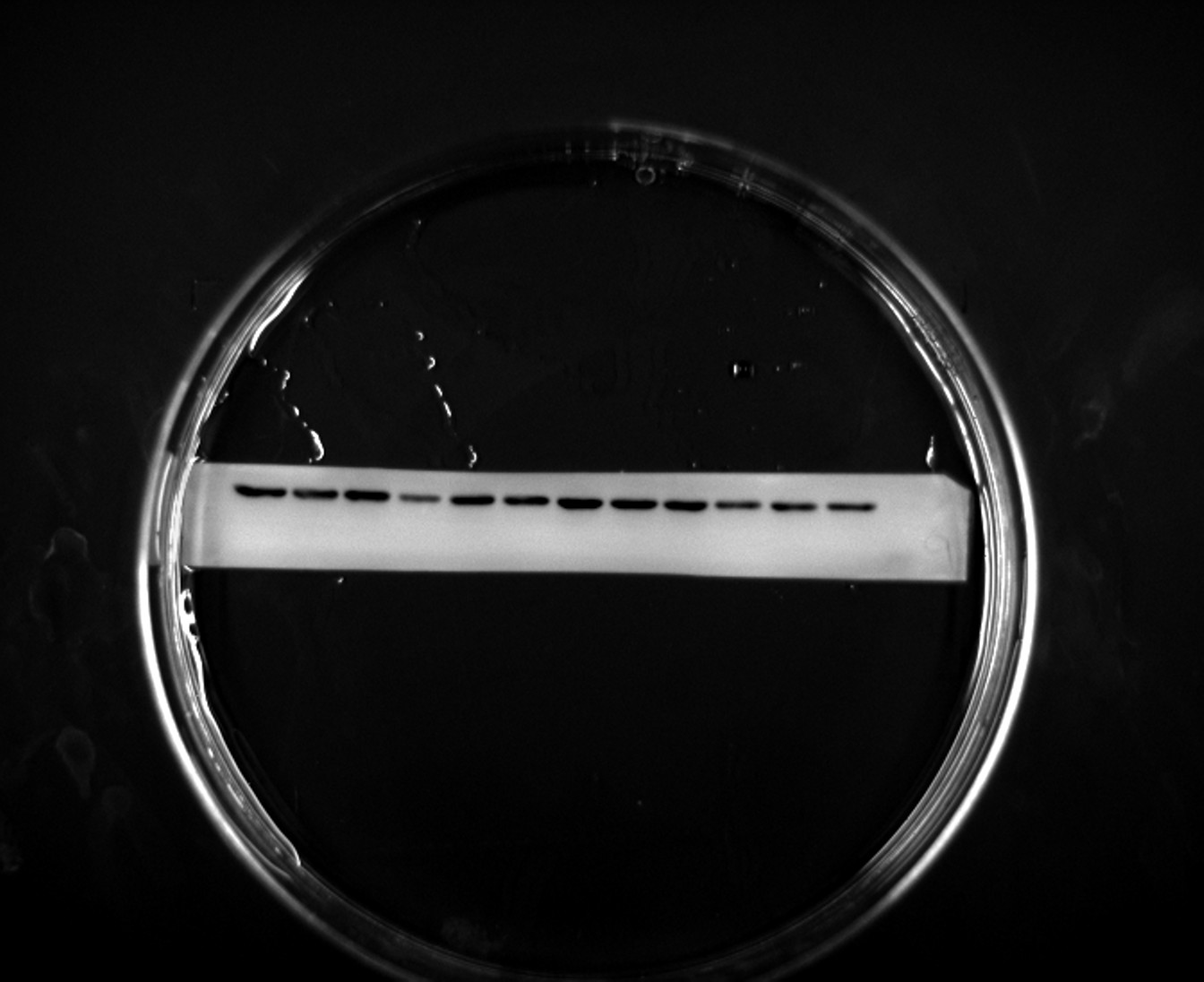

Supplement: Figure 7—source data 1. [file elife-82970-fig7-data1.zip › Figure_7-source_data_1/Figure_7-source_data_1_Figure_7C_a-SMA.jpg]

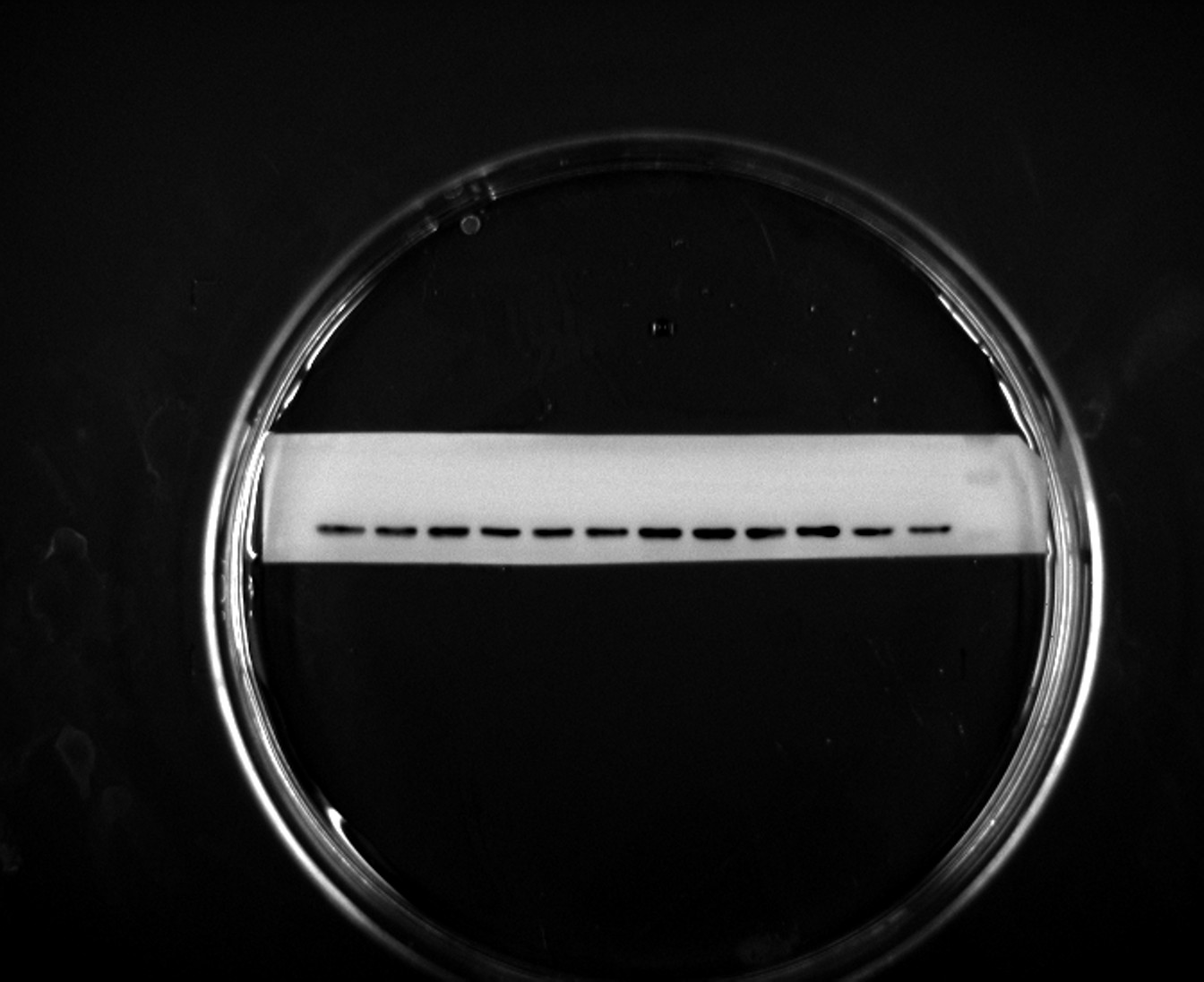

Supplement: Figure 7—source data 1. [file elife-82970-fig7-data1.zip › Figure_7-source_data_1/Figure_7-source_data_1_Figure_7C_GAPDH.jpg]

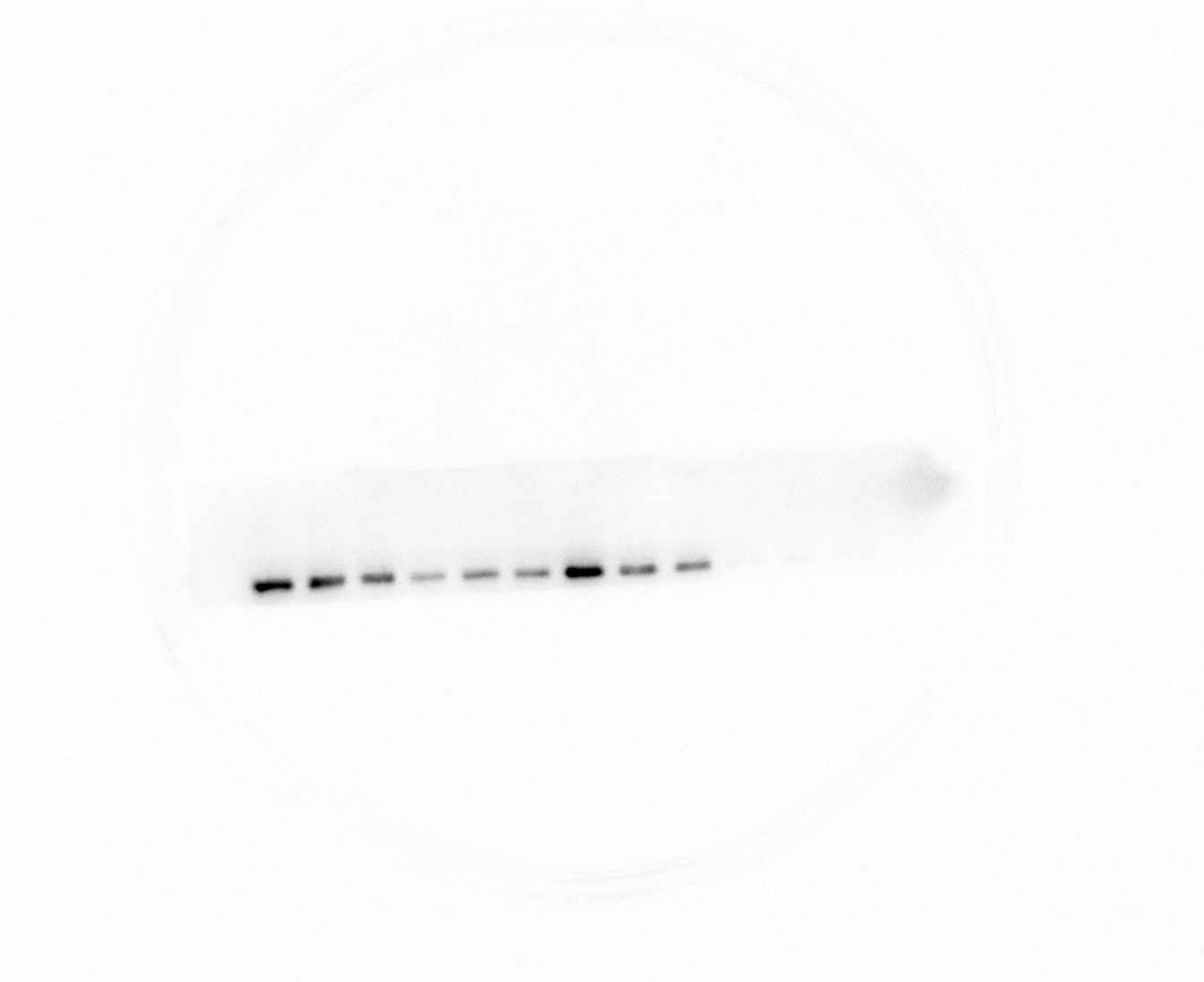

Supplement: Figure 7—source data 1. [file elife-82970-fig7-data1.zip › Figure_7-source_data_1/Figure_7-source_data_1_Figure_7C_SPARC.jpg]

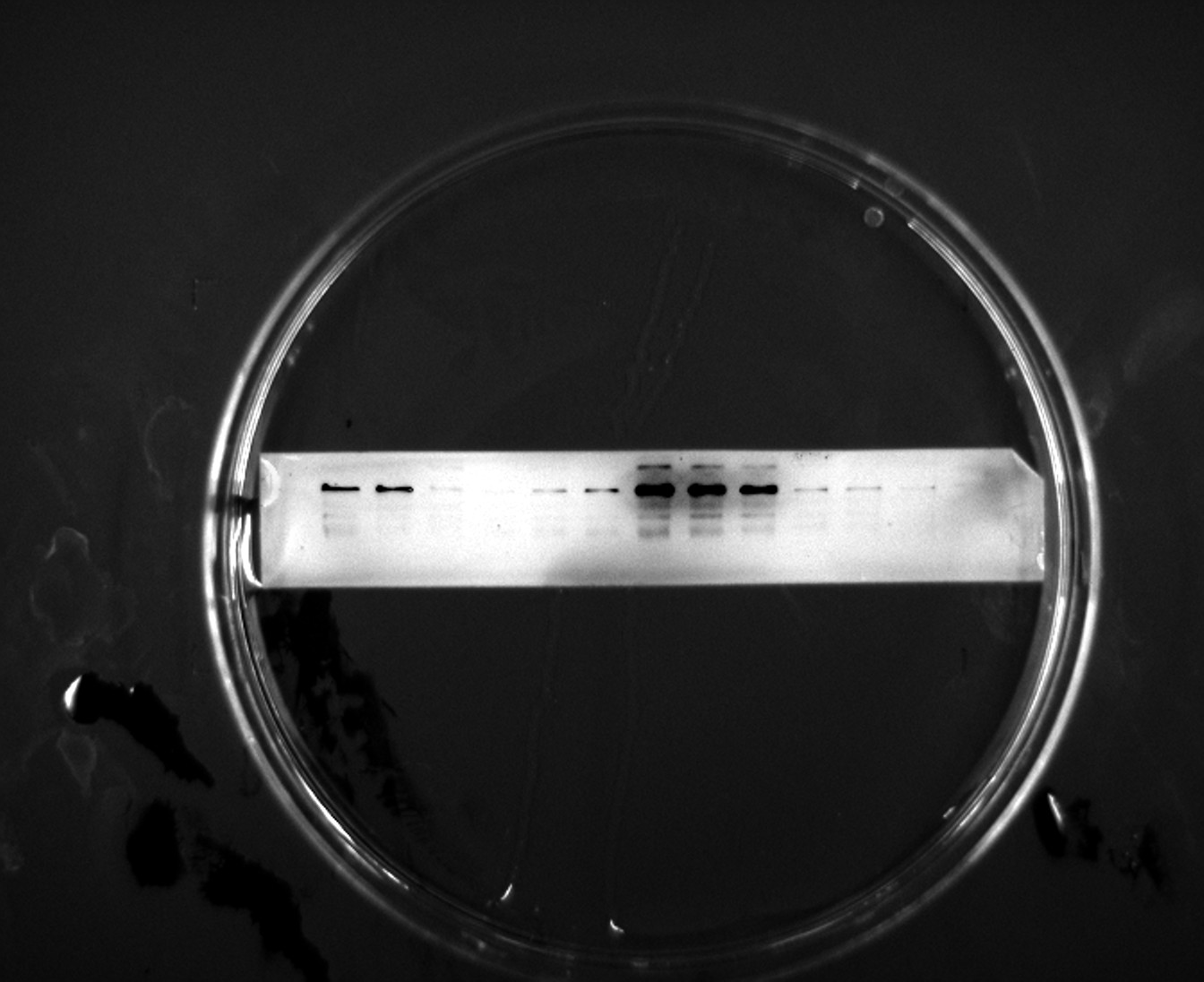

Supplement: Figure 7—source data 1. [file elife-82970-fig7-data1.zip › Figure_7-source_data_1/Figure_7-source_data_1_Figure_7C_TNC.jpg]

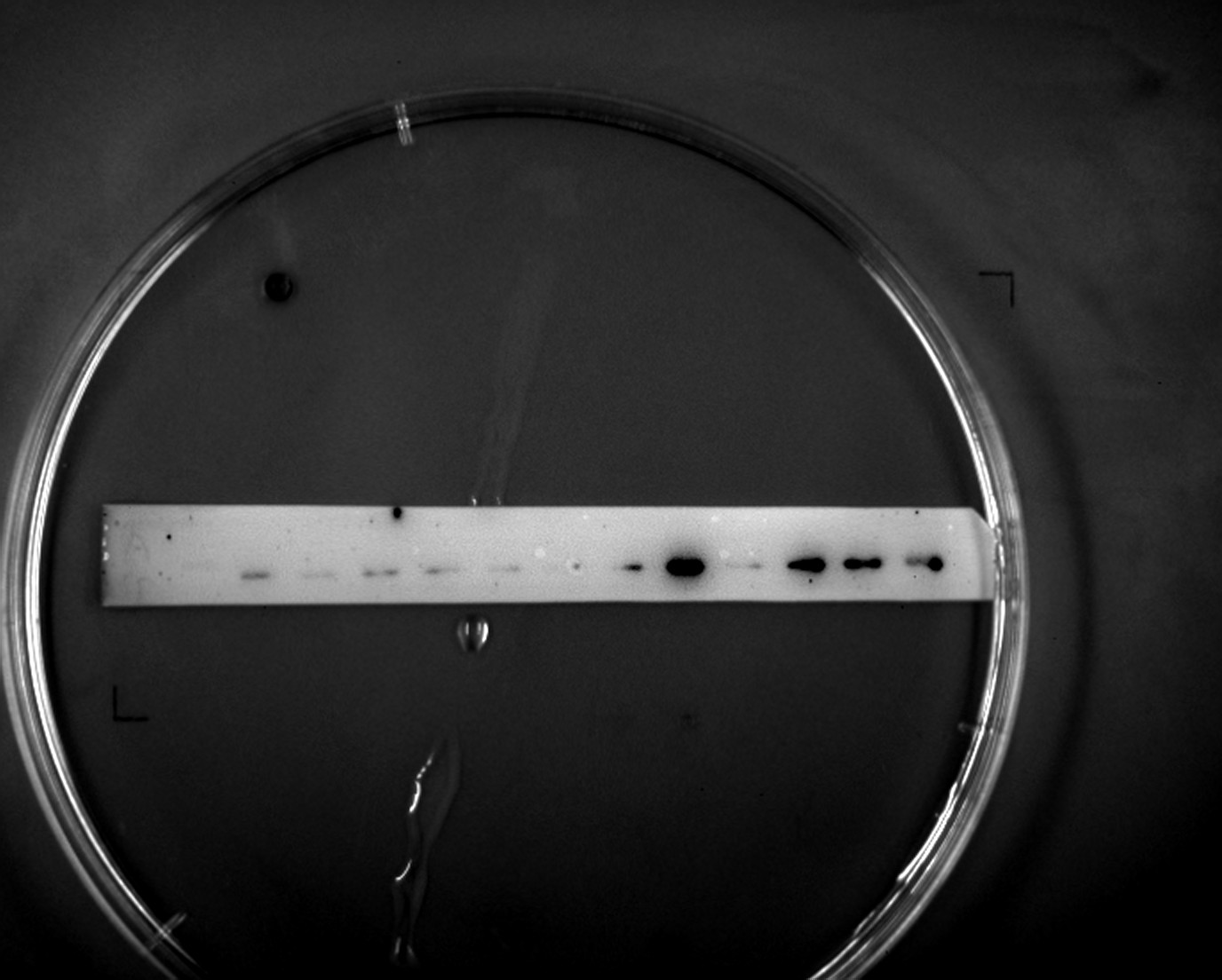

Supplement: Figure 7—source data 1. [file elife-82970-fig7-data1.zip › Figure_7-source_data_1/Figure_7-source_data_1_Figure_7D_SPARC.jpg]

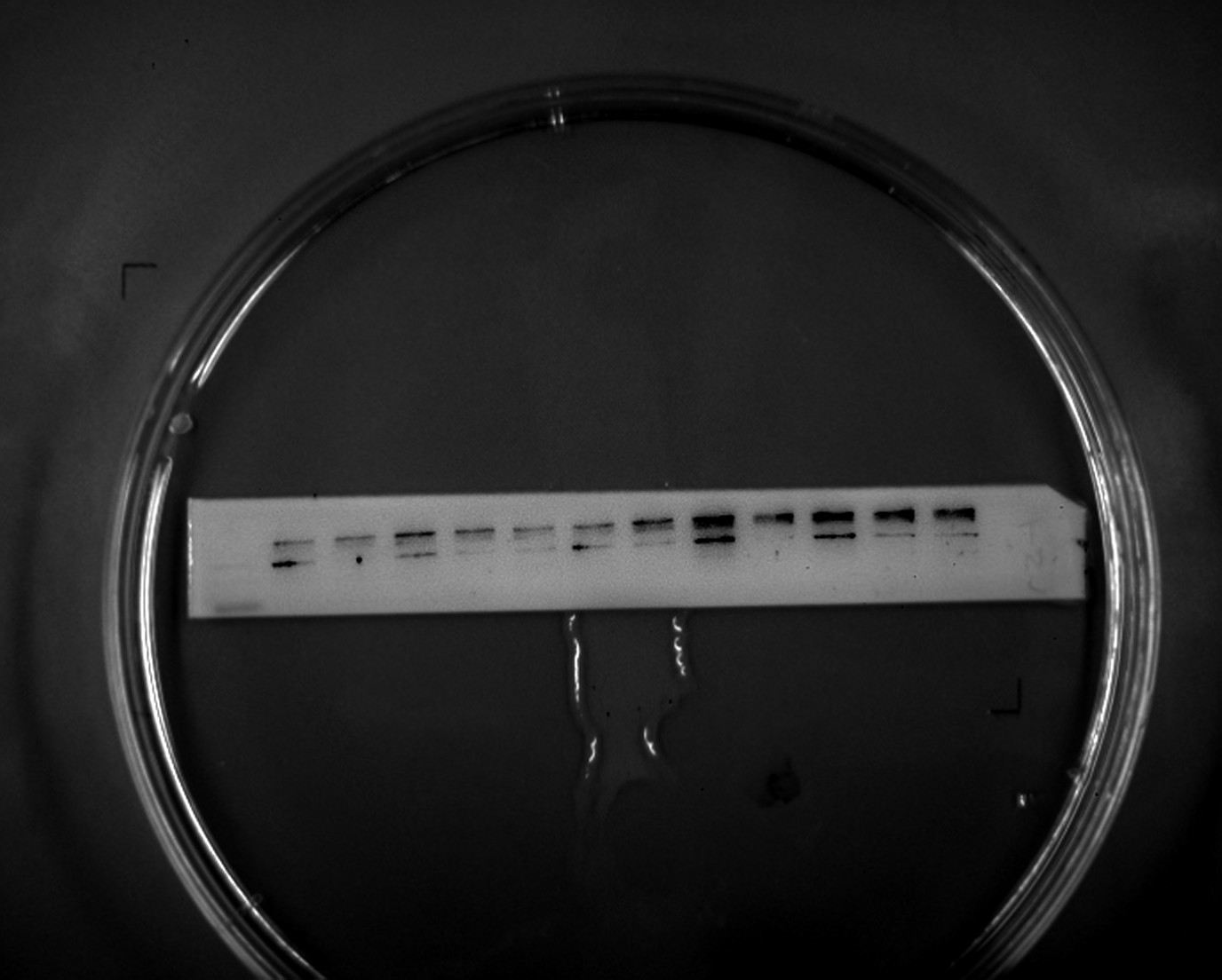

Supplement: Figure 7—source data 1. [file elife-82970-fig7-data1.zip › Figure_7-source_data_1/Figure_7-source_data_1_Figure_7D_TNC.jpg]

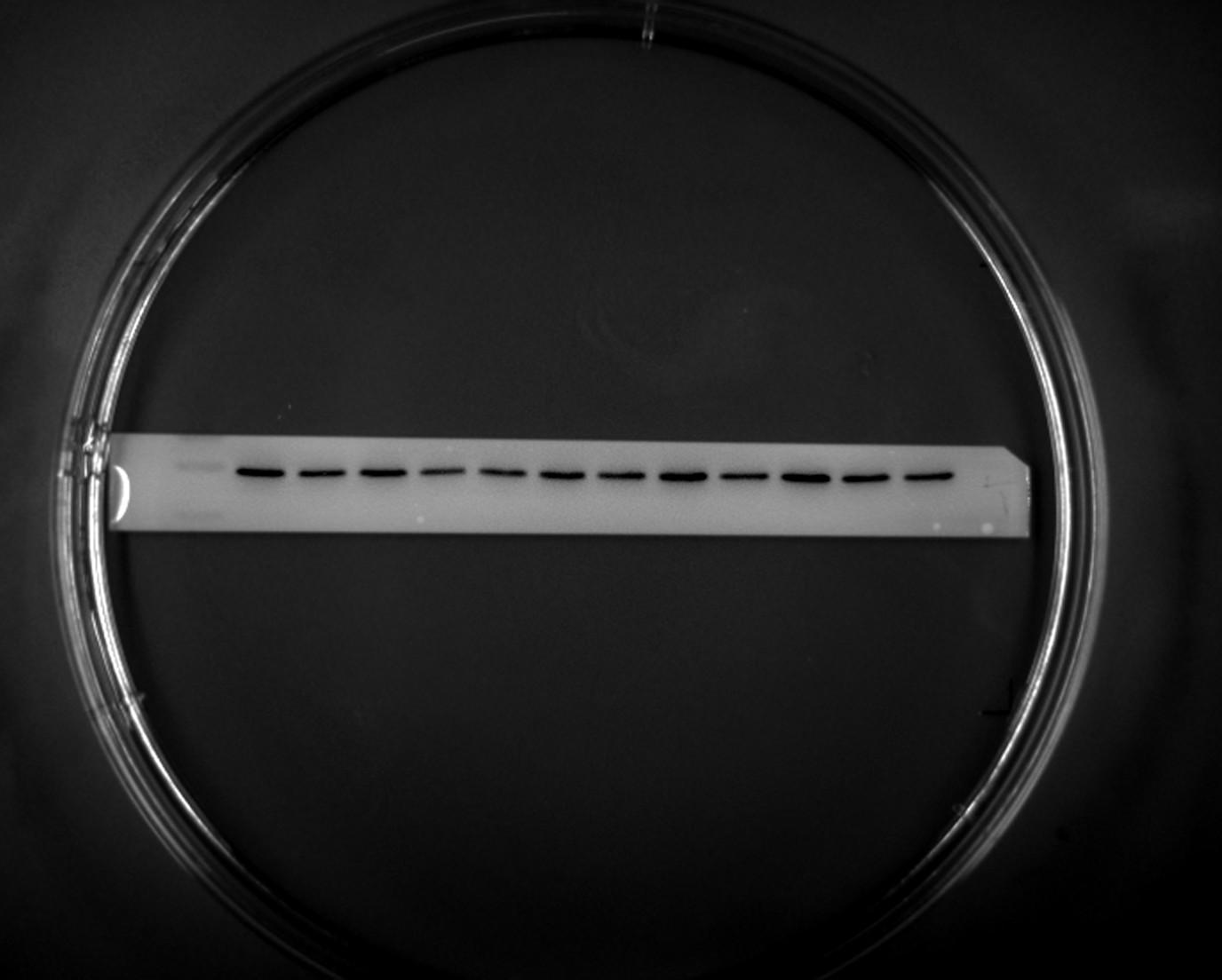

Supplement: Figure 7—source data 1. [file elife-82970-fig7-data1.zip › Figure_7-source_data_1/Figure_7-source_data_1_Figure_7D_TUBULIN.jpg]

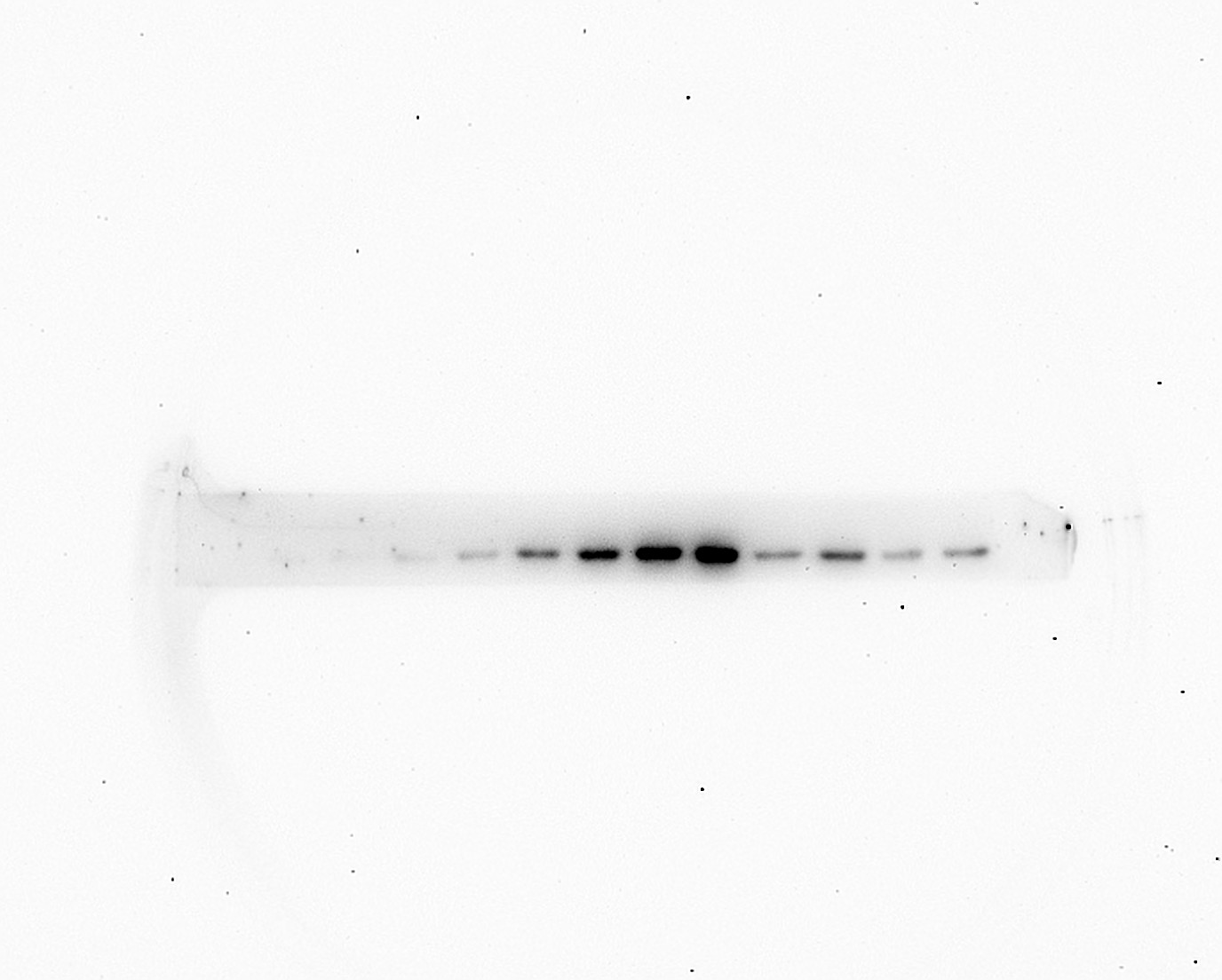

Supplement: Figure 7—source data 1. [file elife-82970-fig7-data1.zip › Figure_7-source_data_1/Figure_7-source_data_1_Figure_7D_a┴-SMA.jpg]

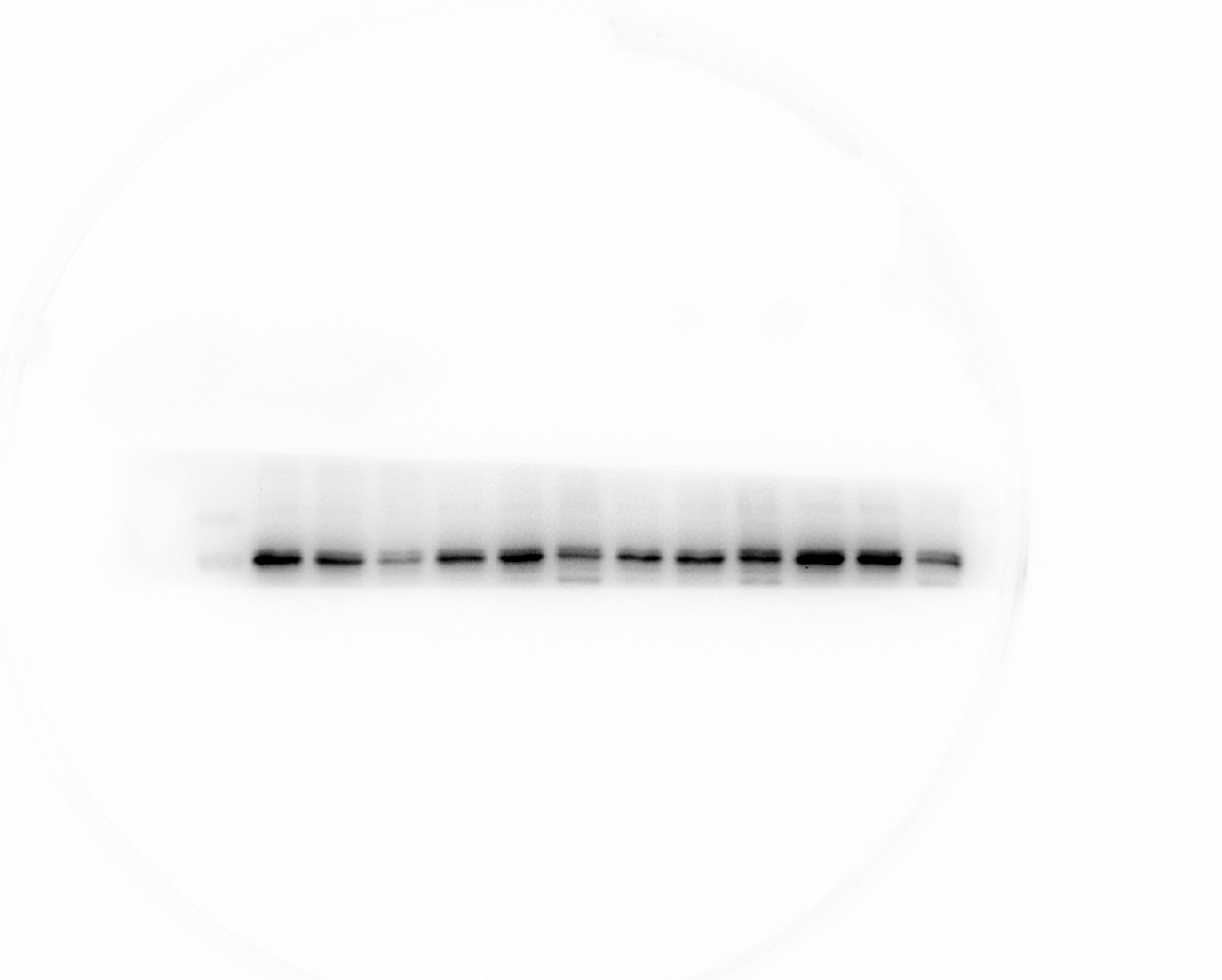

Supplement: Figure 7—source data 1. [file elife-82970-fig7-data1.zip › Figure_7-source_data_1/Figure_7-source_data_1_Figure_7E_COX2.jpg]

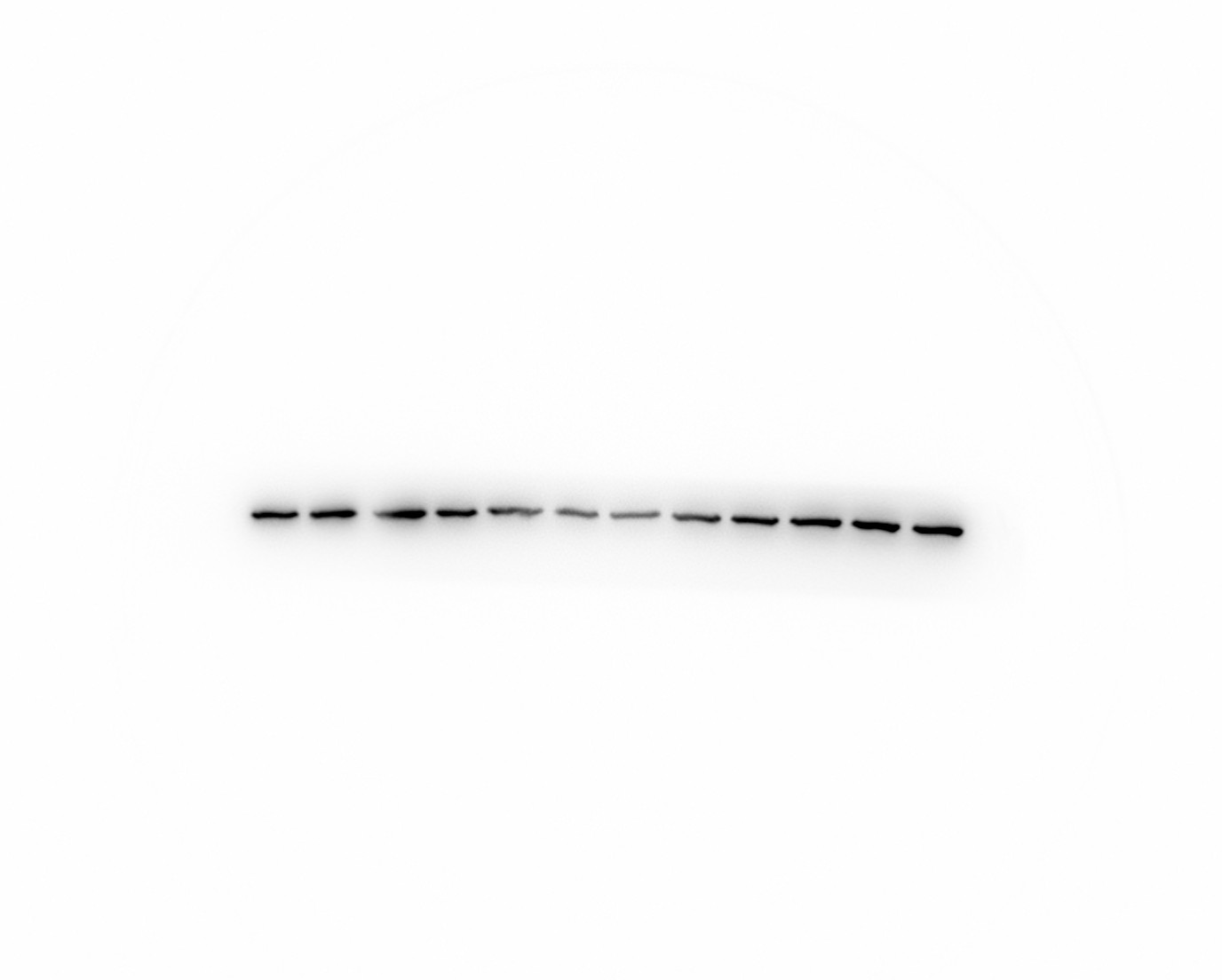

Supplement: Figure 7—source data 1. [file elife-82970-fig7-data1.zip › Figure_7-source_data_1/Figure_7-source_data_1_Figure_7E_IP.jpg]

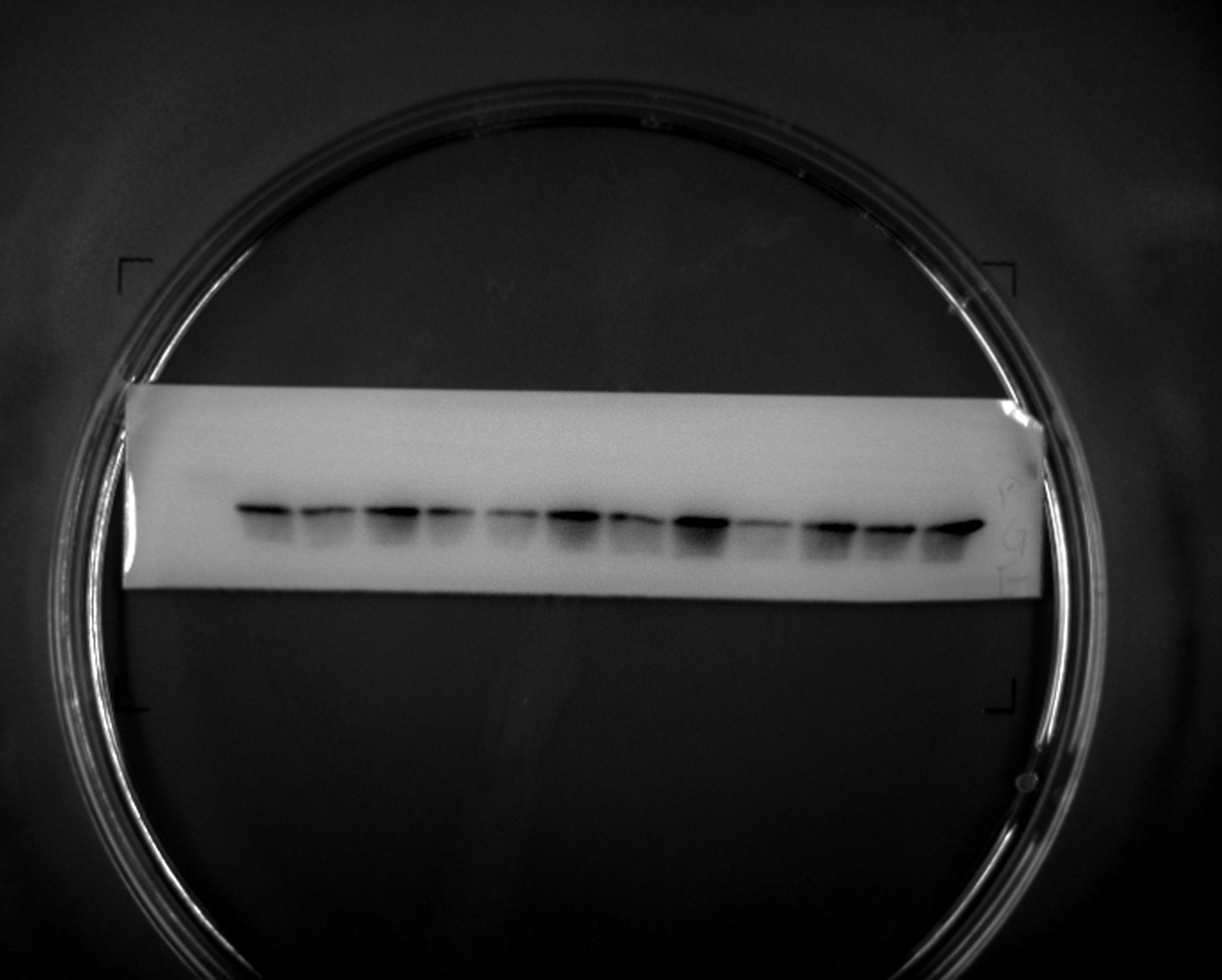

Supplement: Figure 7—source data 1. [file elife-82970-fig7-data1.zip › Figure_7-source_data_1/Figure_7-source_data_1_Figure_7E_PGES.jpg]

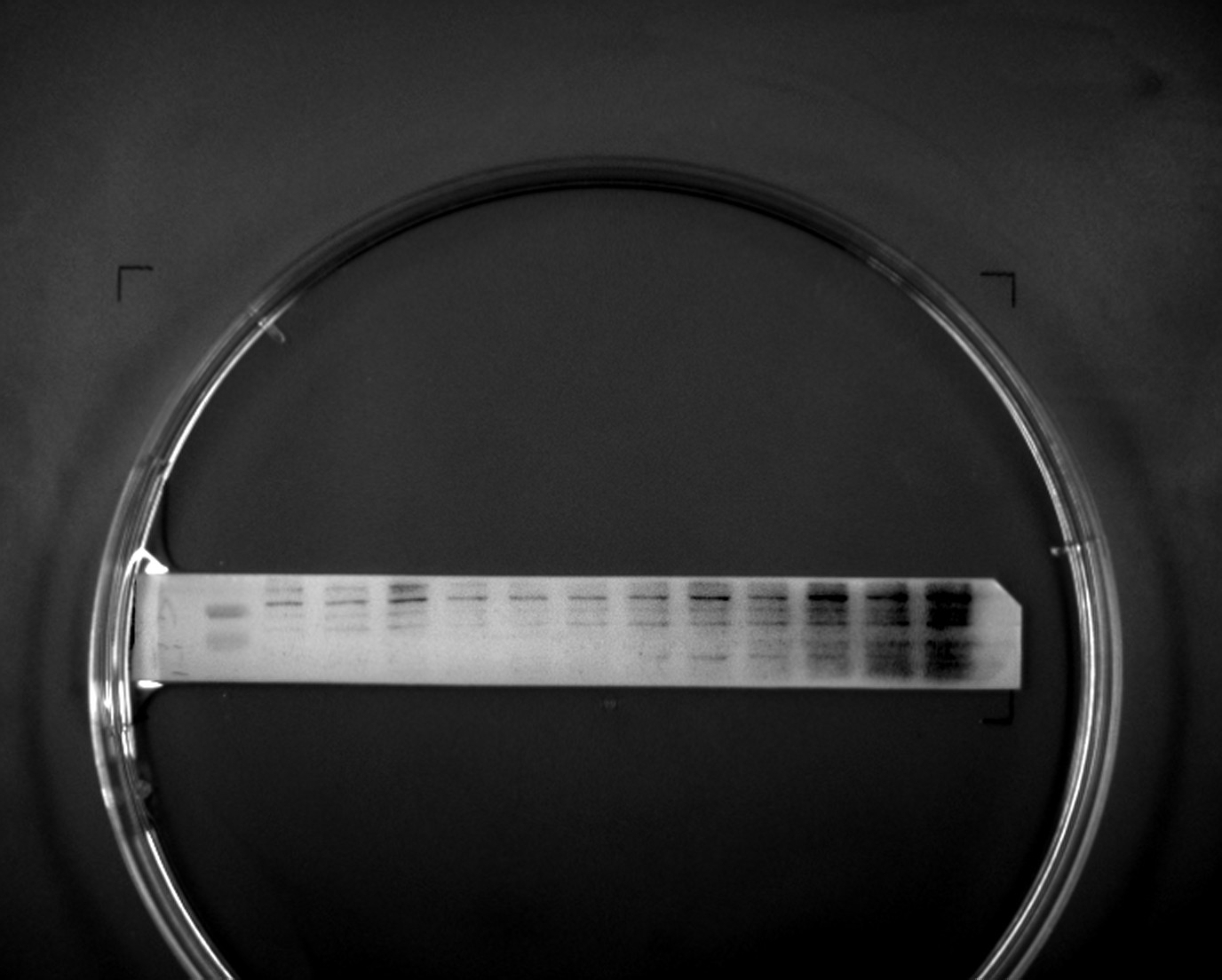

Supplement: Figure 7—source data 1. [file elife-82970-fig7-data1.zip › Figure_7-source_data_1/Figure_7-source_data_1_Figure_7E_PGIS.jpg]

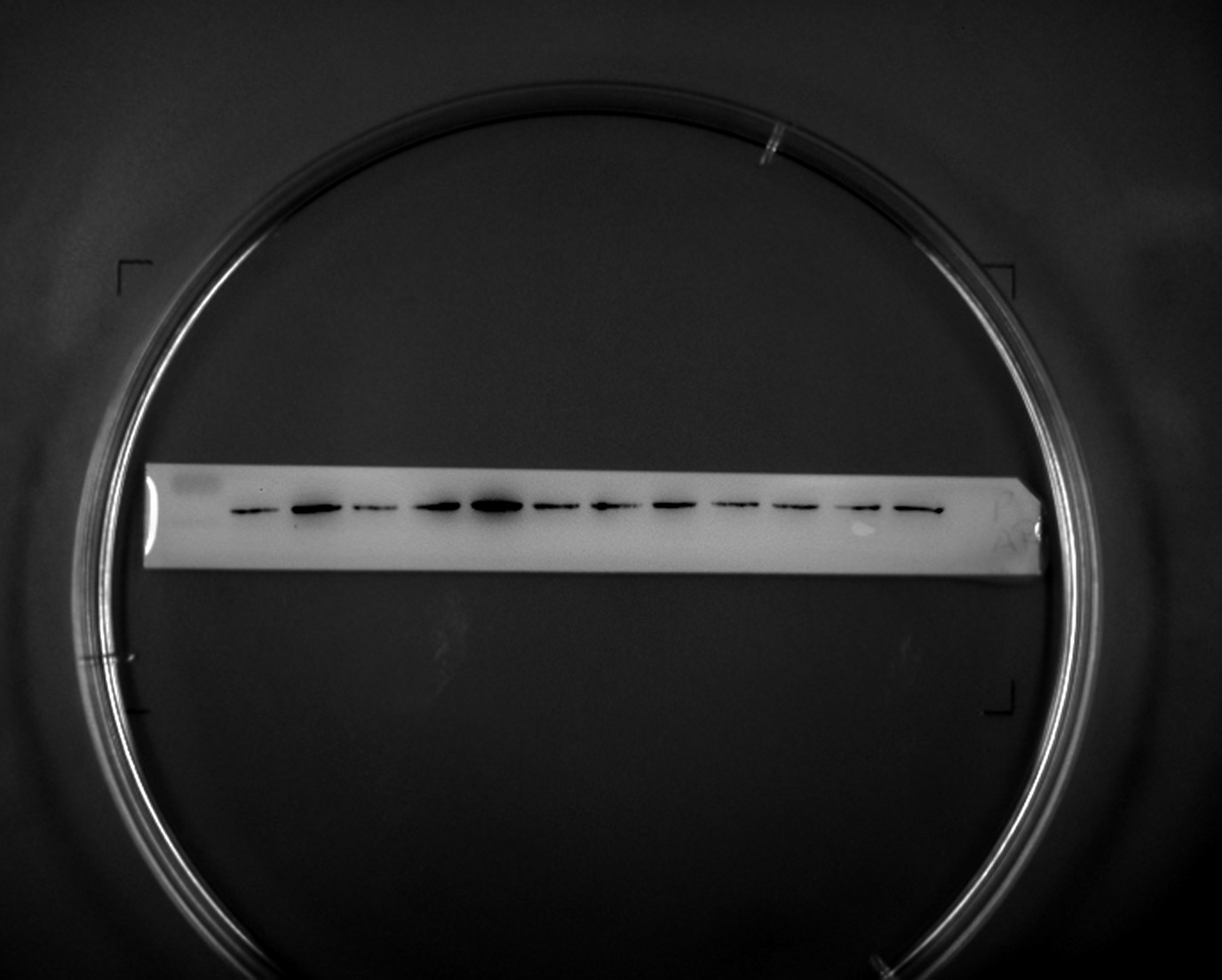

Supplement: Figure 7—source data 1. [file elife-82970-fig7-data1.zip › Figure_7-source_data_1/Figure_7-source_data_1_Figure_7E_PPARa─.jpg]

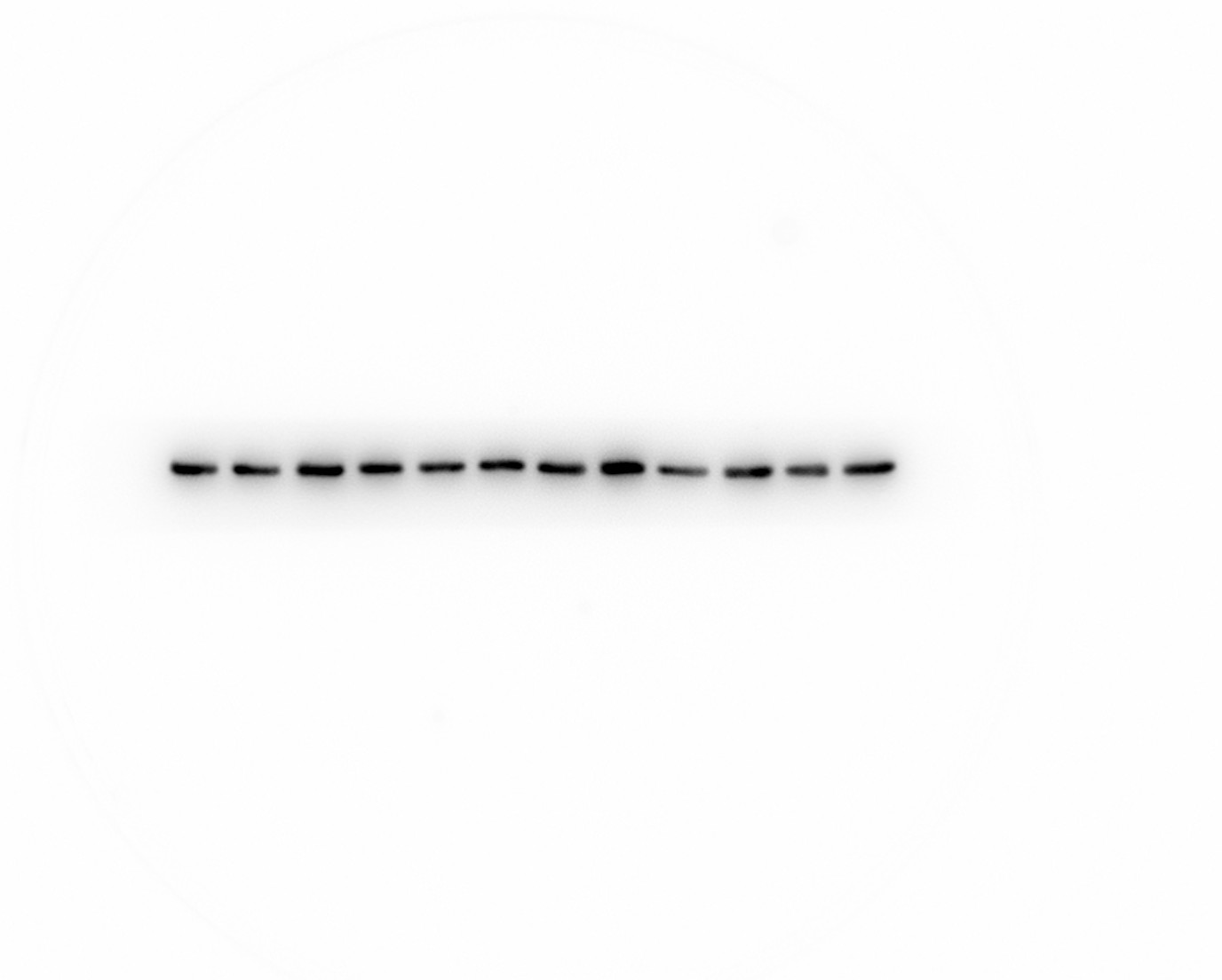

Supplement: Figure 7—source data 1. [file elife-82970-fig7-data1.zip › Figure_7-source_data_1/Figure_7-source_data_1_Figure_7E_TUBULIN.jpg]

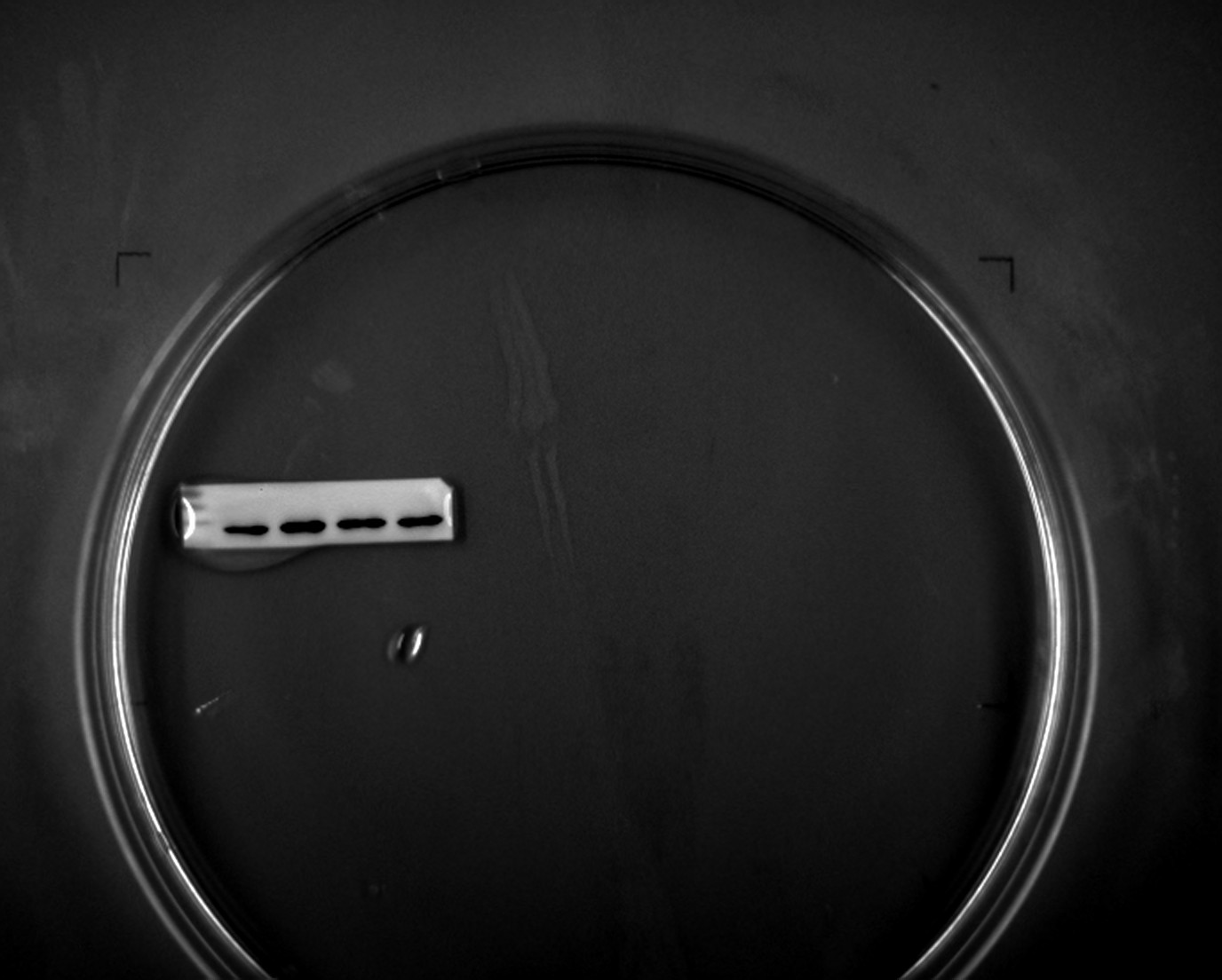

Supplement: Figure 7—source data 1. [file elife-82970-fig7-data1.zip › Figure_7-source_data_1/Figure_7-source_data_1_Figure_7F_SPARC.jpg]

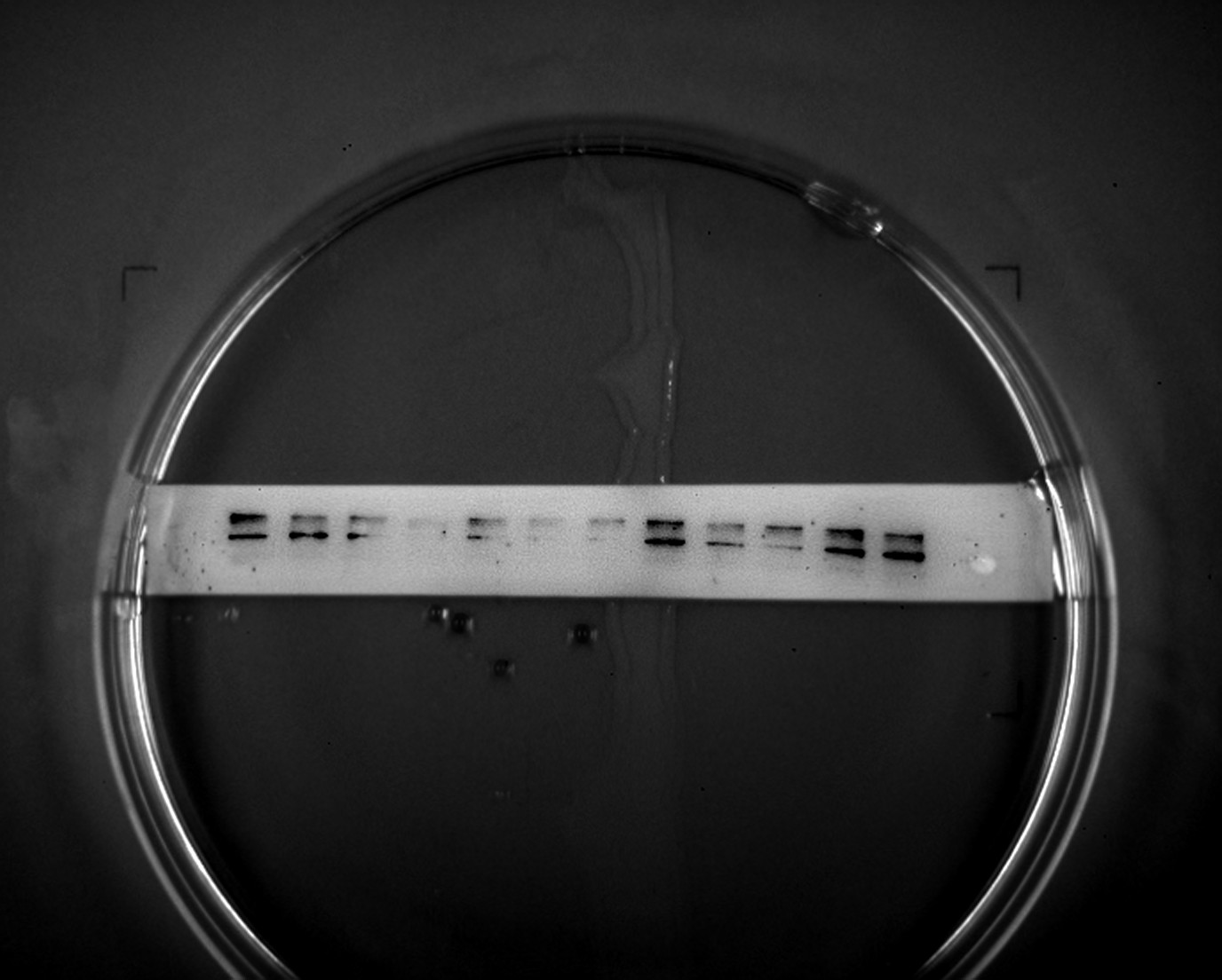

Supplement: Figure 7—source data 1. [file elife-82970-fig7-data1.zip › Figure_7-source_data_1/Figure_7-source_data_1_Figure_7F_TNC.jpg]

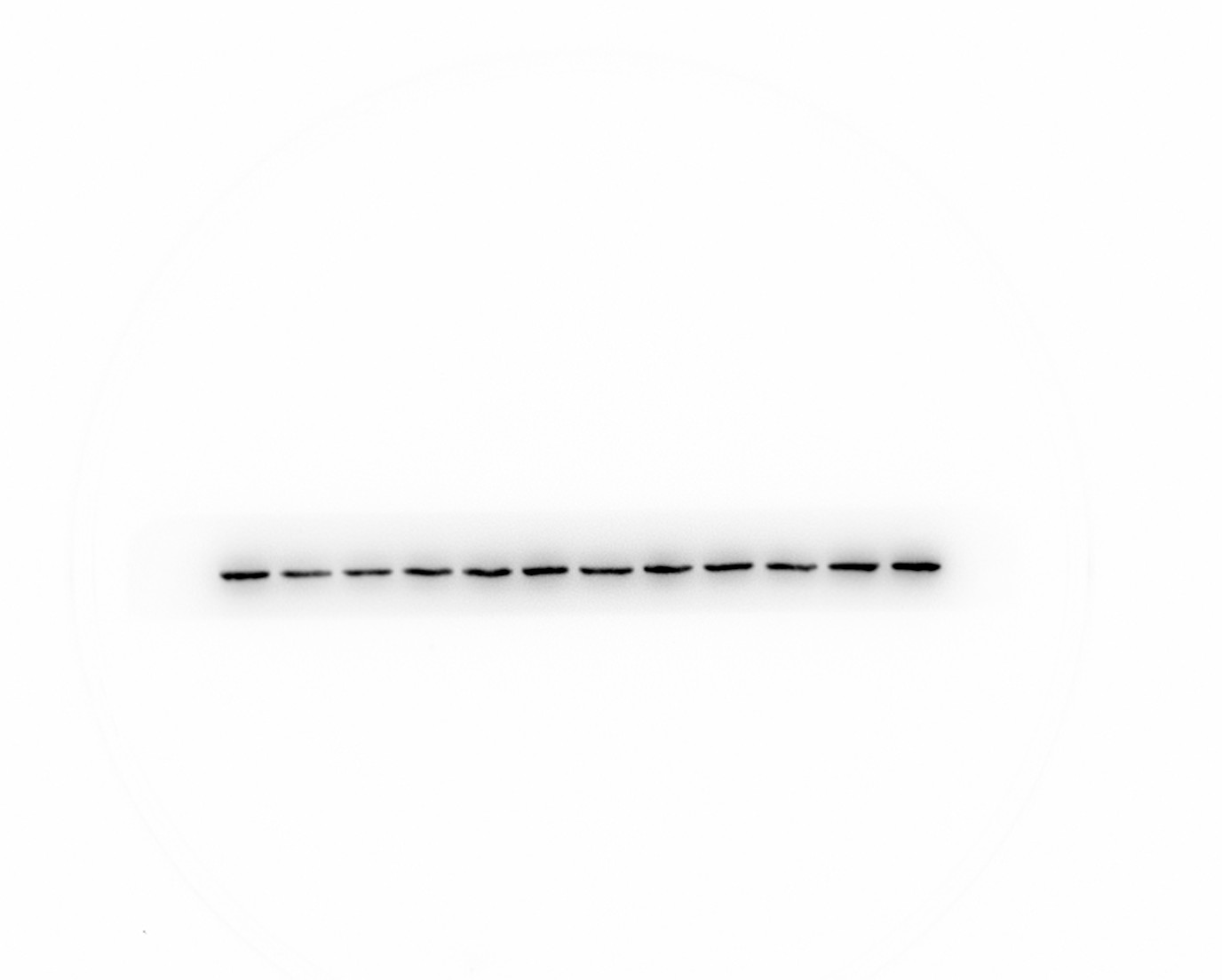

Supplement: Figure 7—source data 1. [file elife-82970-fig7-data1.zip › Figure_7-source_data_1/Figure_7-source_data_1_Figure_7F_TUBULIN.jpg]

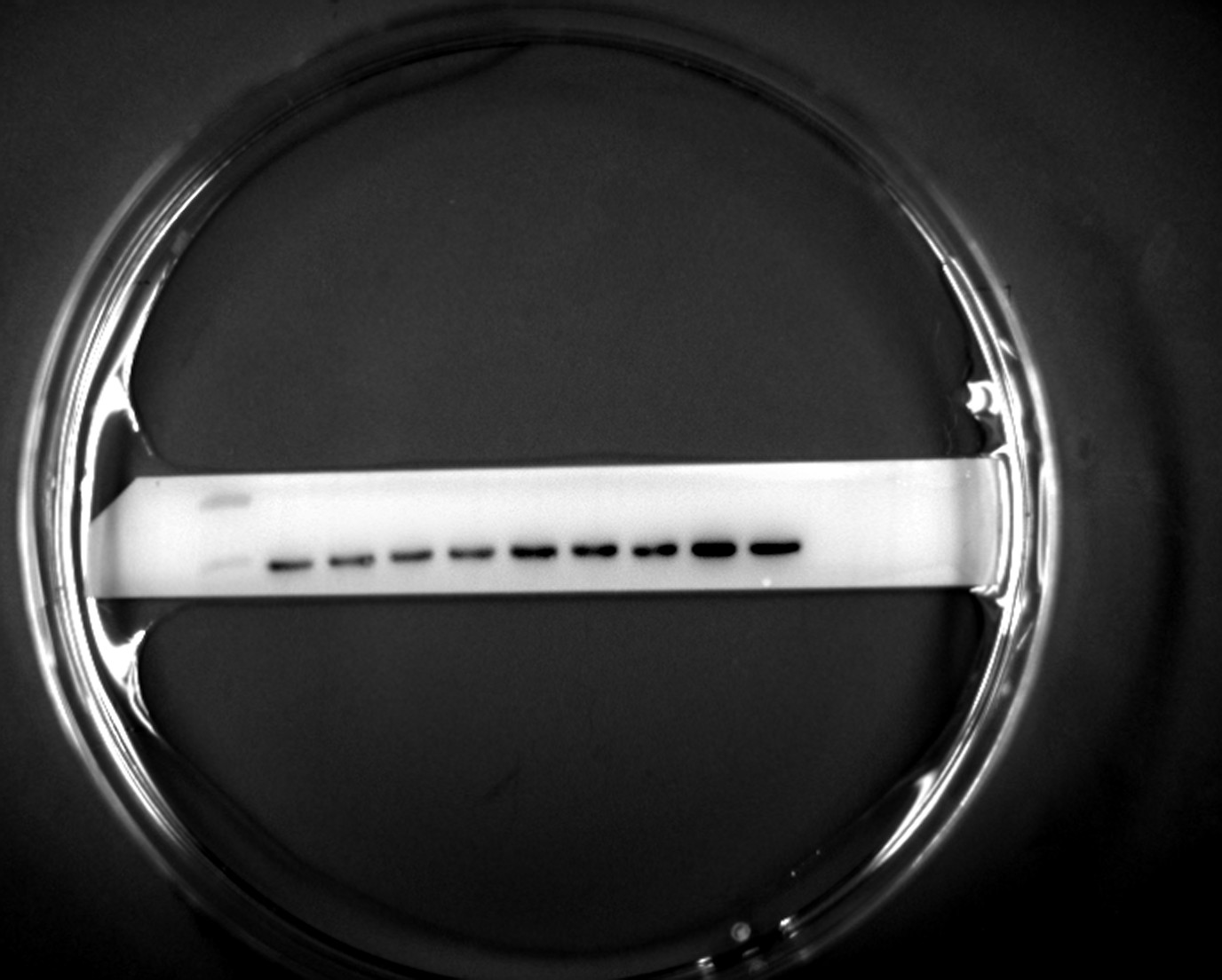

Supplement: Figure 7—source data 1. [file elife-82970-fig7-data1.zip › Figure_7-source_data_1/Figure_7-source_data_1_Figure_7F_a┴-SMA.jpg]

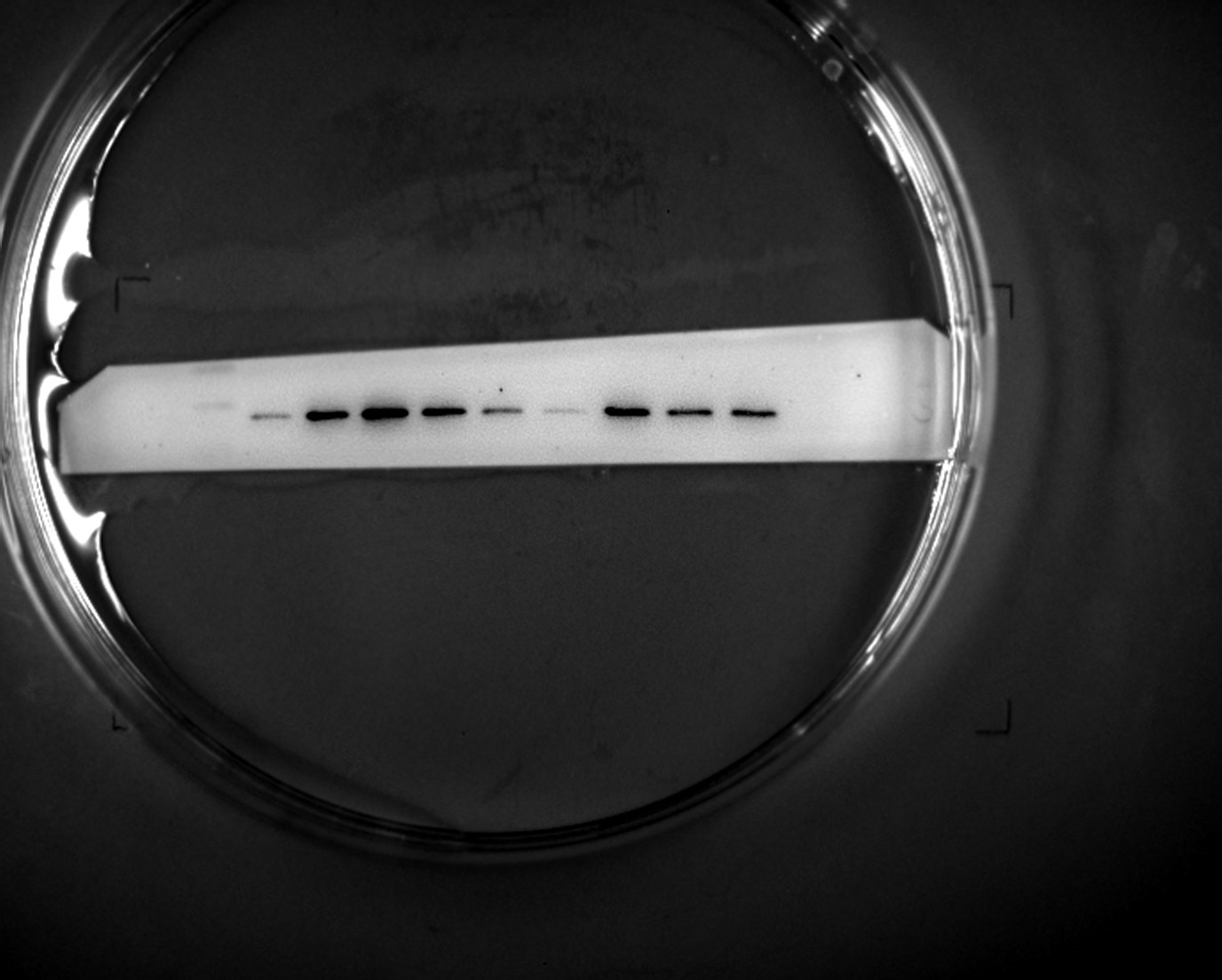

Supplement: Figure 7—source data 1. [file elife-82970-fig7-data1.zip › Figure_7-source_data_1/Figure_7-source_data_1_Figure_7G_SPARC.jpg]

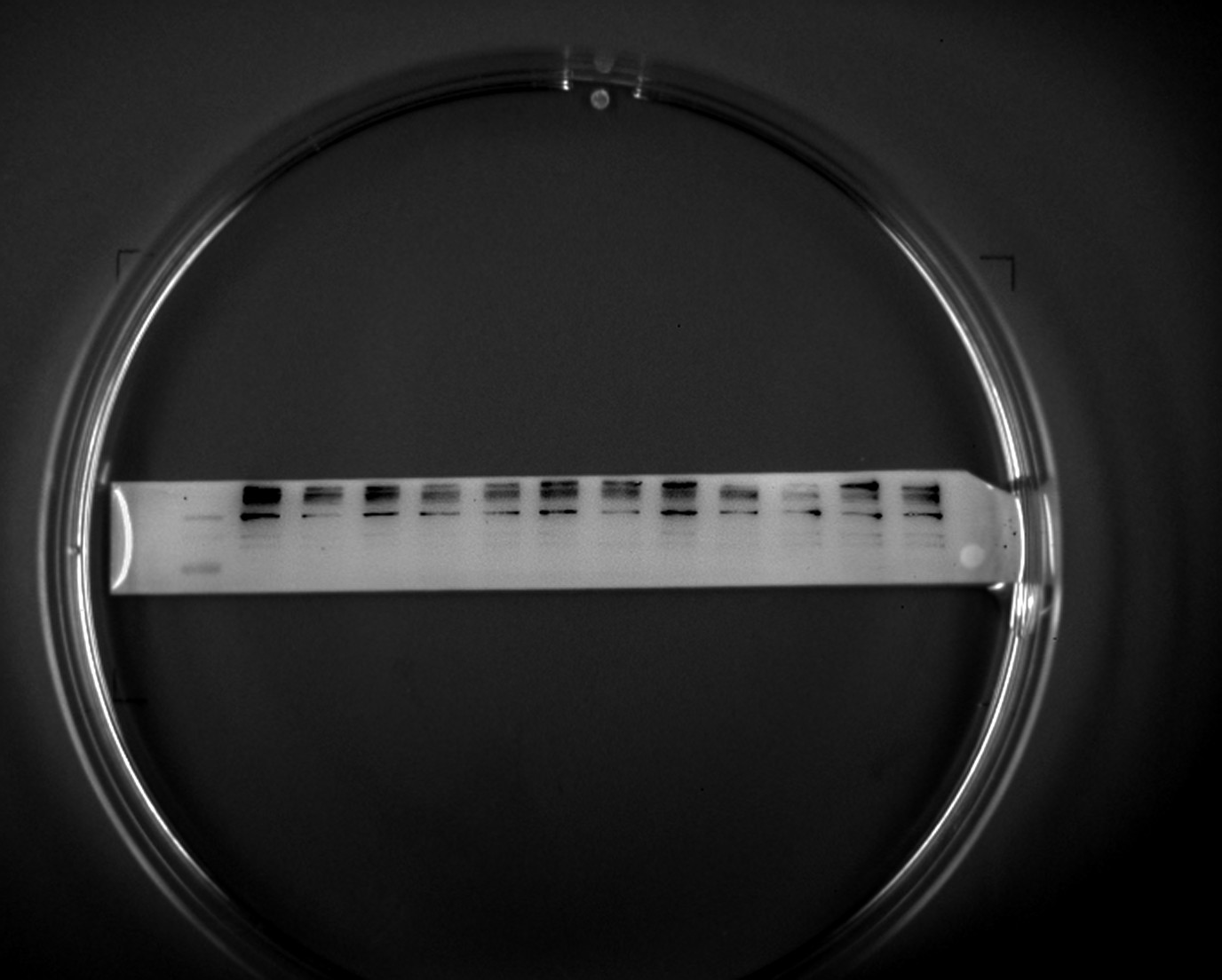

Supplement: Figure 7—source data 1. [file elife-82970-fig7-data1.zip › Figure_7-source_data_1/Figure_7-source_data_1_Figure_7G_TNC.jpg]

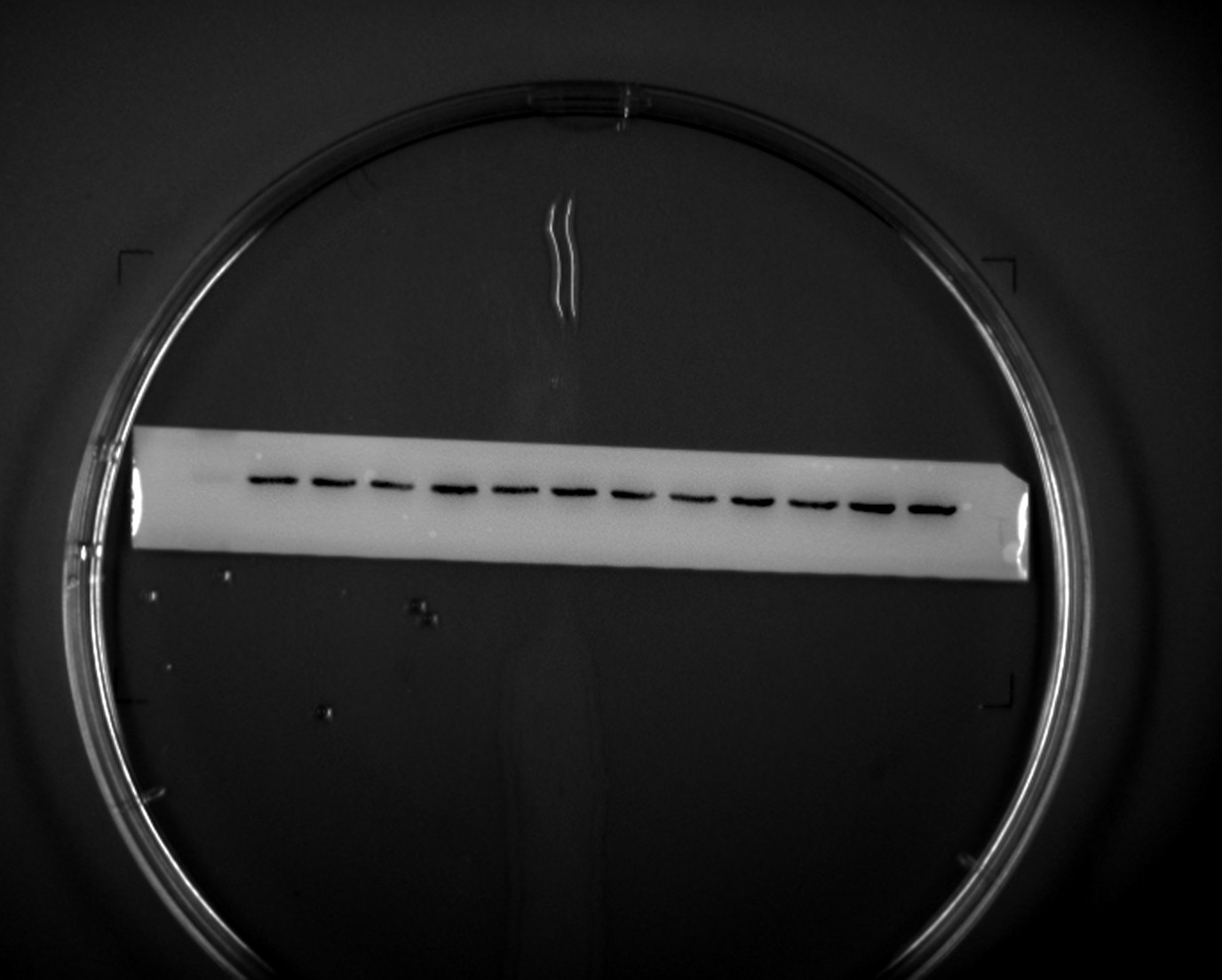

Supplement: Figure 7—source data 1. [file elife-82970-fig7-data1.zip › Figure_7-source_data_1/Figure_7-source_data_1_Figure_7G_TUBULIN.jpg]

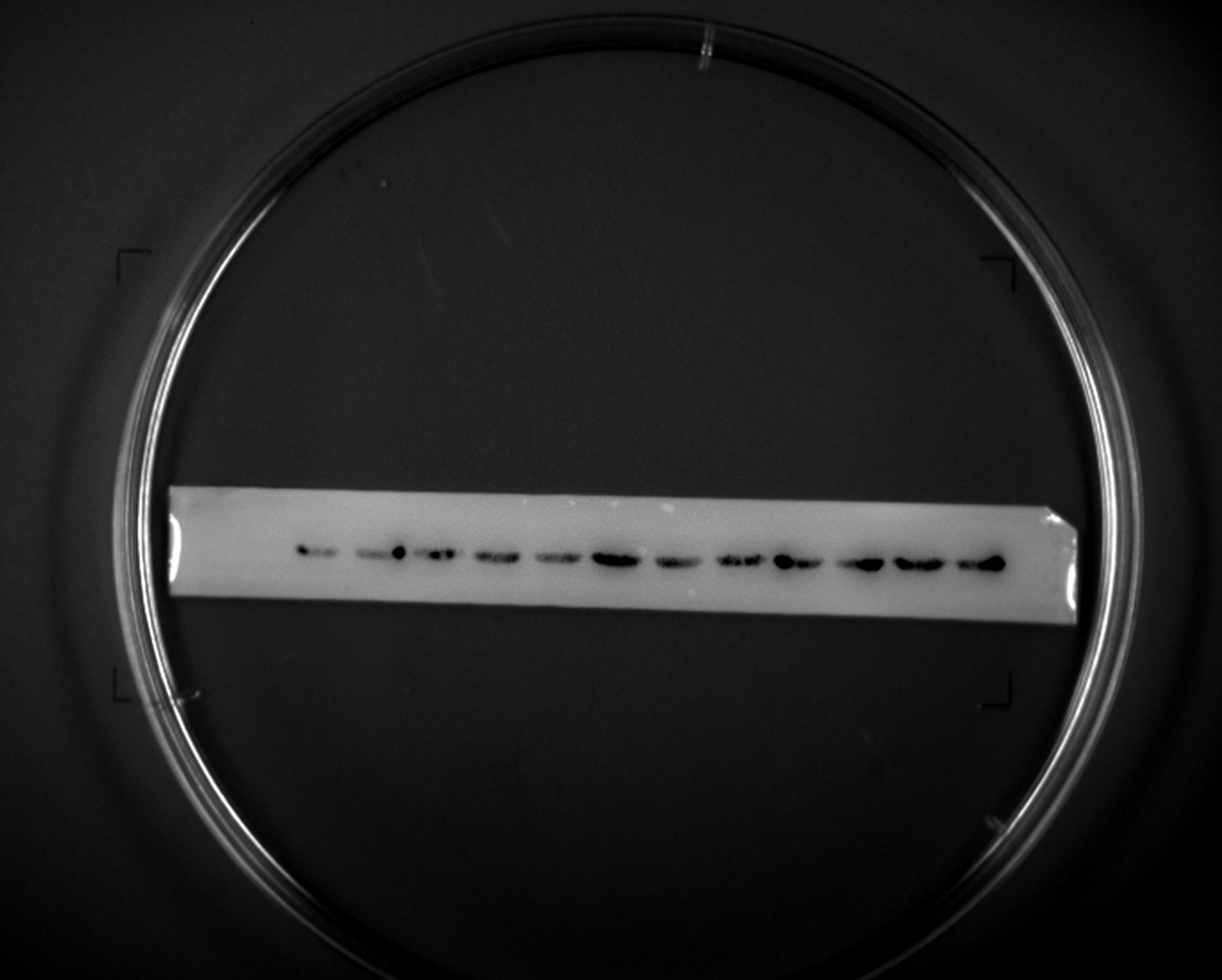

Supplement: Figure 7—source data 1. [file elife-82970-fig7-data1.zip › Figure_7-source_data_1/Figure_7-source_data_1_Figure_7G_a┴-SMA.jpg]

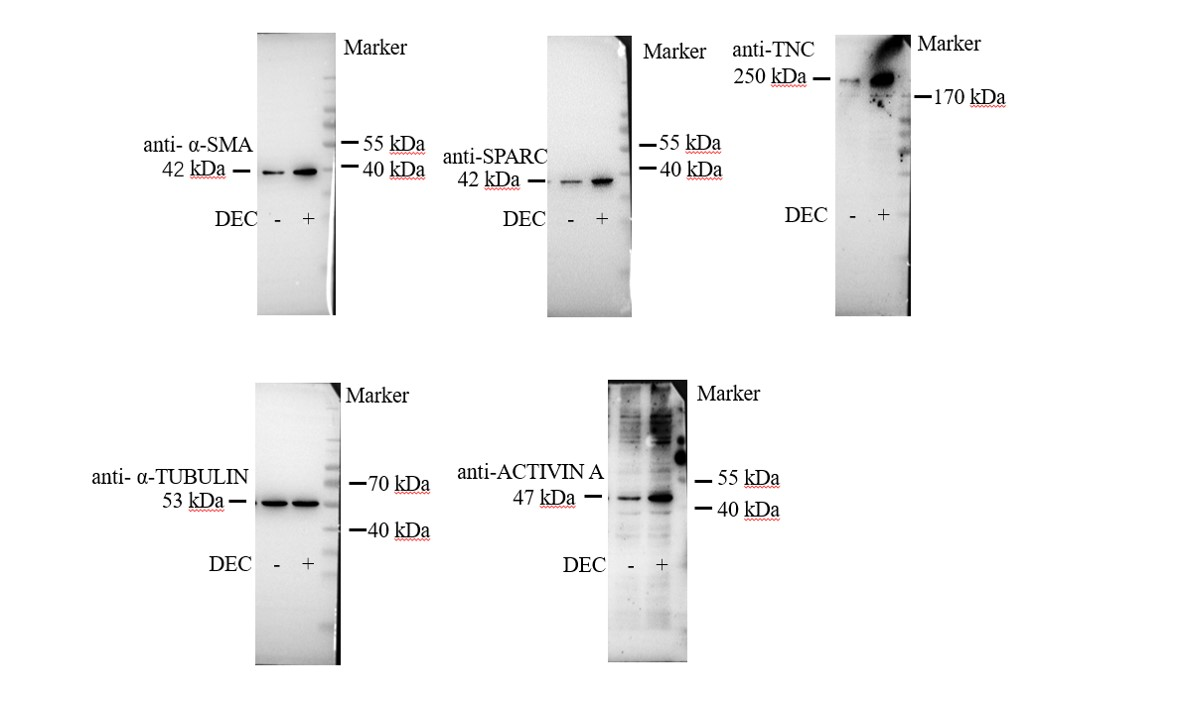

Supplement: Figure 7—source data 2. [file elife-82970-fig7-data2.zip › Figure_7-source_data_2-7A.png]

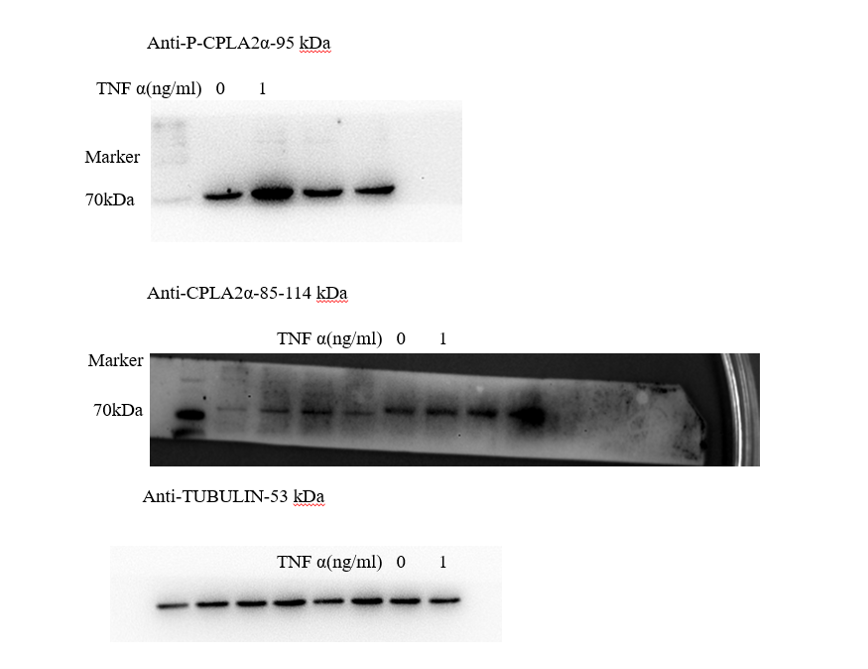

Supplement: Figure 7—source data 2. [file elife-82970-fig7-data2.zip › Figure_7-source_data_2-7B.png]

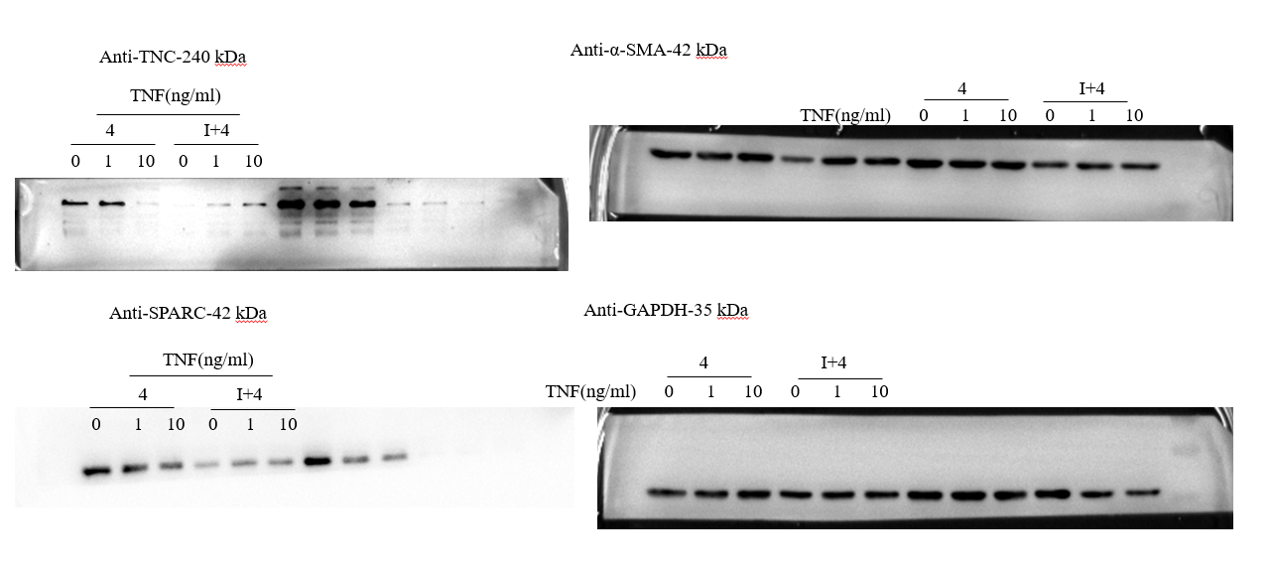

Supplement: Figure 7—source data 2. [file elife-82970-fig7-data2.zip › Figure_7-source_data_2-7C.png]

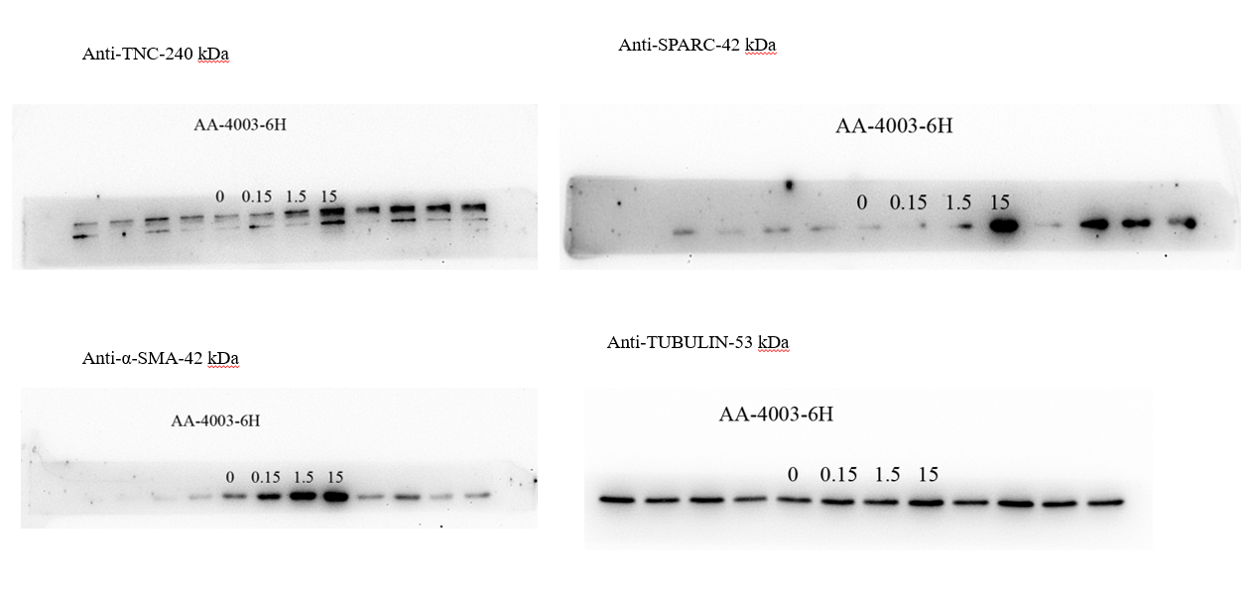

Supplement: Figure 7—source data 2. [file elife-82970-fig7-data2.zip › Figure_7-source_data_2-7D.png]

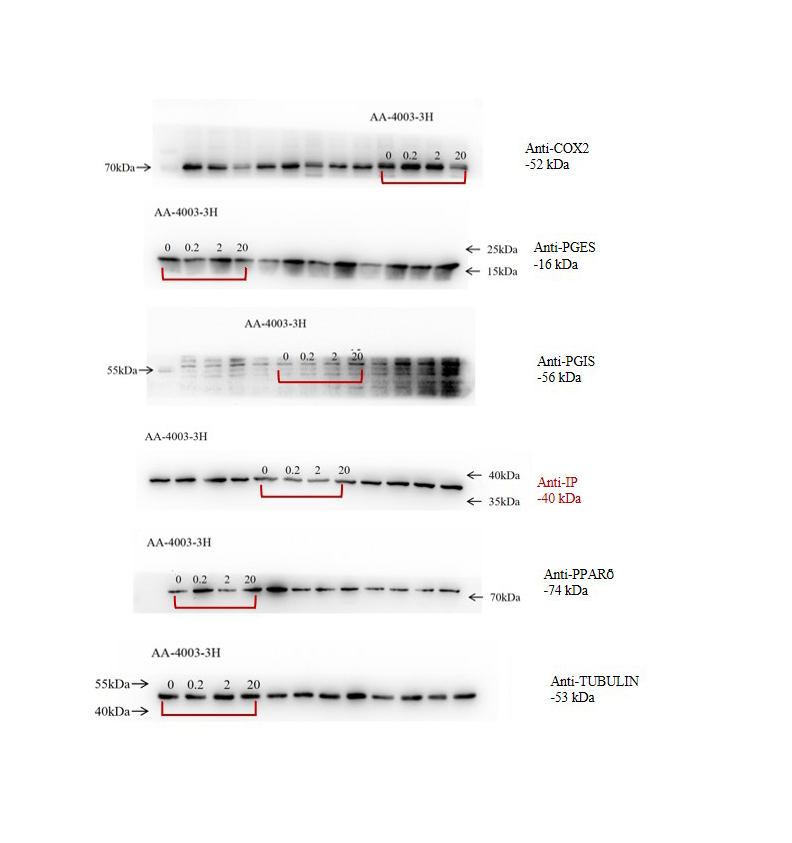

Supplement: Figure 7—source data 2. [file elife-82970-fig7-data2.zip › Figure_7-source_data_2-7E.jpg]

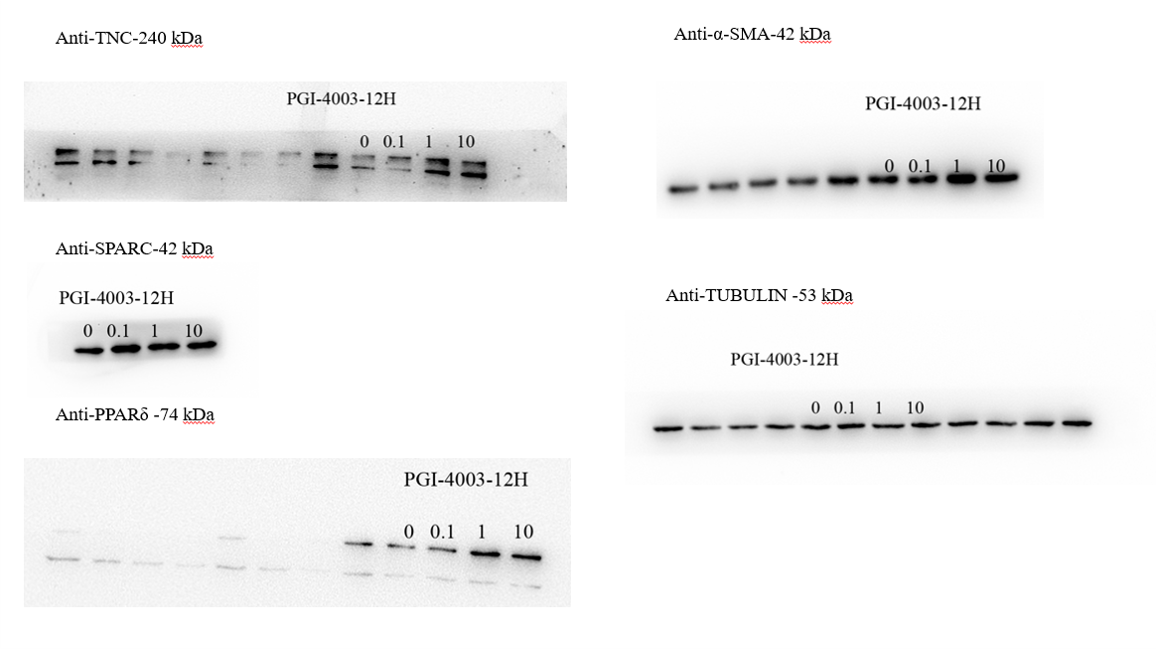

Supplement: Figure 7—source data 2. [file elife-82970-fig7-data2.zip › Figure_7-source_data_2-7F.png]

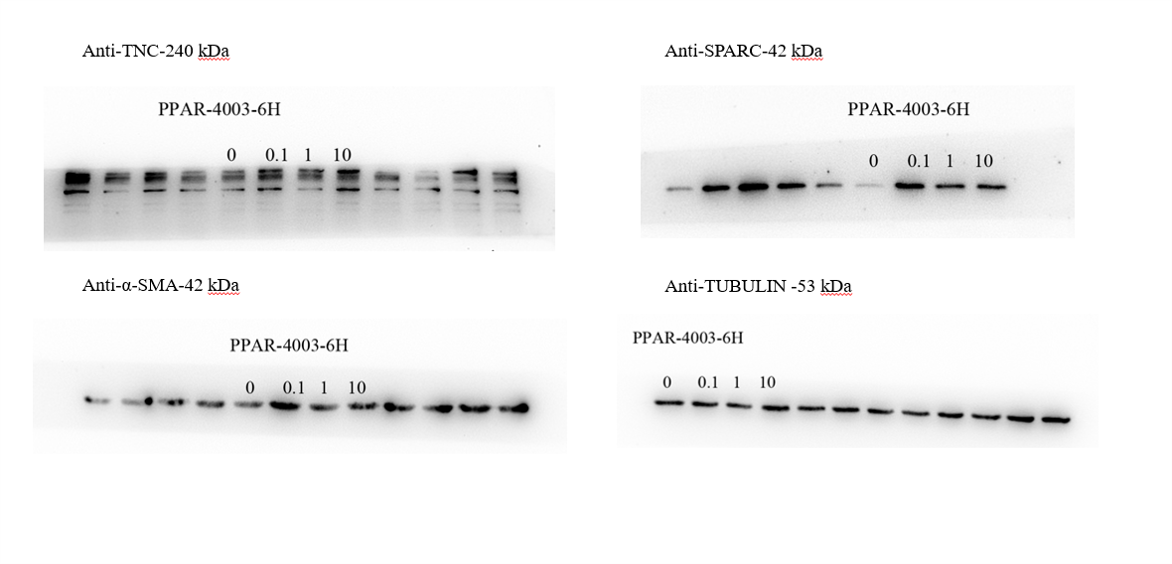

Supplement: Figure 7—source data 2. [file elife-82970-fig7-data2.zip › Figure_7-source_data_2-7G.png]
