# Supplementary material for: Embryo-derive TNF promotes decidualization via fibroblast activation
Source: eLife. 2023 Jul 17;12:e82970. doi: 10.7554/eLife.82970 (PMC10374279; doi:10.7554/eLife.82970)
Supplement: Source data 1. [file elife-82970-data1.docx]

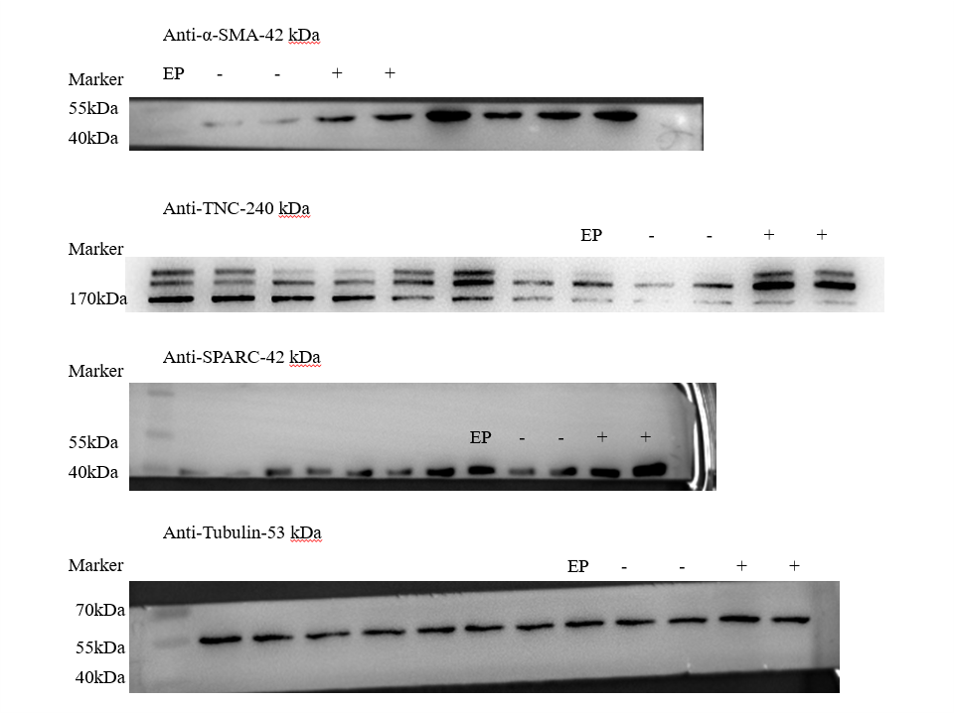


Fig. 1B-source data-1. Western blot analysis of α-SMA, SPARC, TNC protein level under in vitro decidualization (EP) for 24 h.


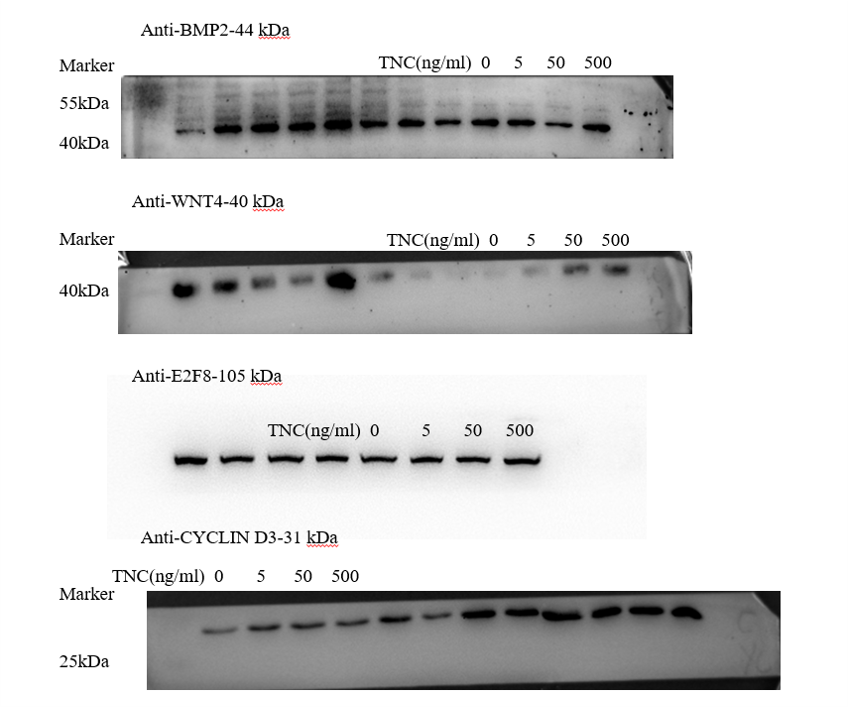


Fig. 2A-source data-1. Western blot analysis on the effects of TNC on decidualization markers (BMP2, WNT4, E2F8 and CYCLIN D3) after stromal cells were treatment with TNC for 72 h.


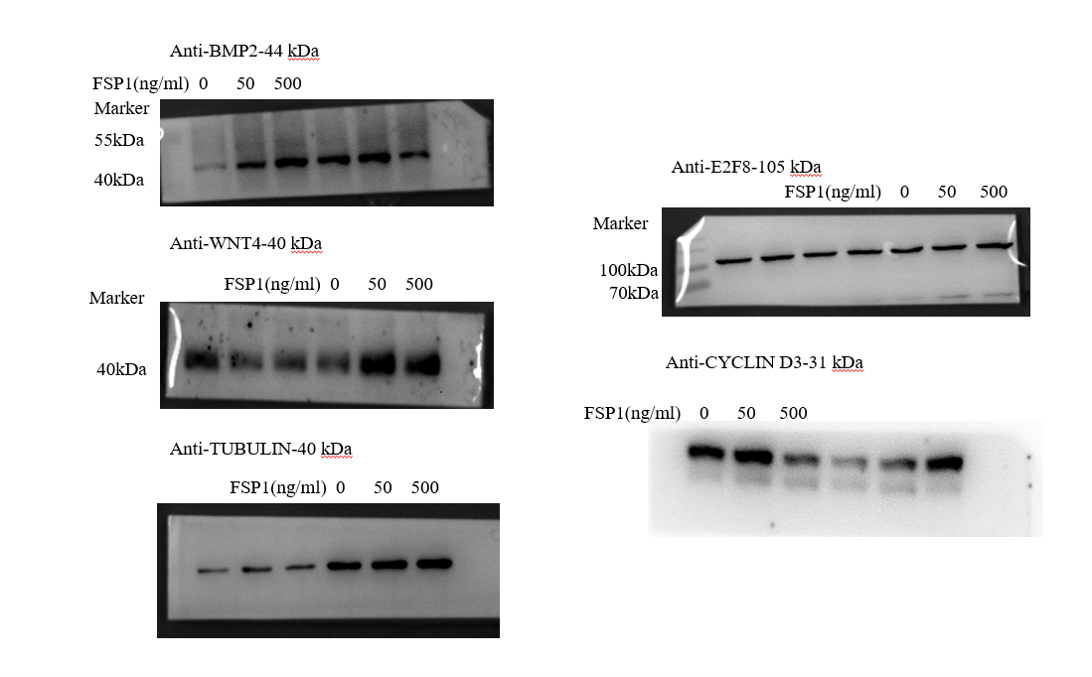


Fig. 2B-source data-2. Western blot analysis of the effects of FSP1 on decidualization markers after stromal cells were treated with FSP1 for 72 h.


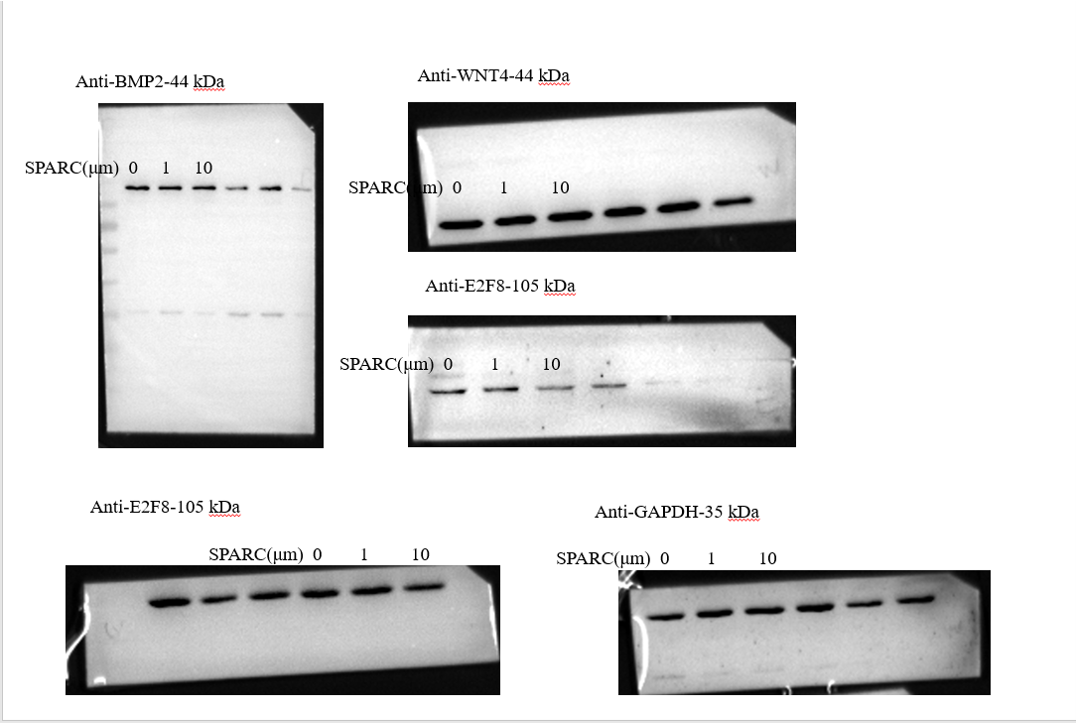


Fig. 2C-source data-3. Western blot analysis and QPCR analysis of *Prl8a2* mRNA level on the effects after stromal cells were treated with SPARC for 72 h.


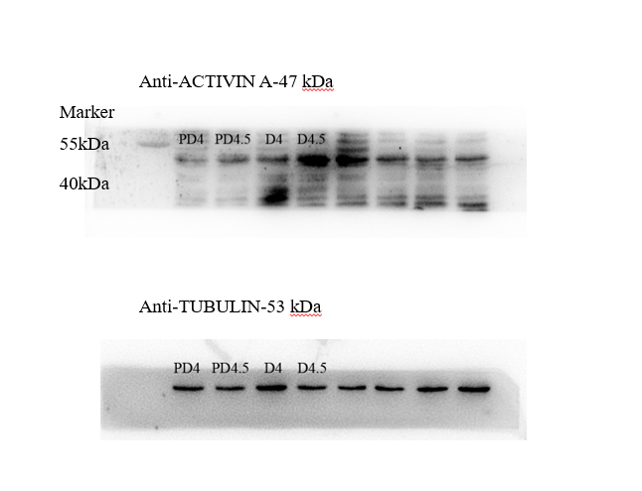


Fig. 2D-source data-4. Western blot analysis on ACTIVIN A protein levels in mouse uteri on day 4 0900 and day 4 2400 of pregnancy and pseudopregnancy, respectively.


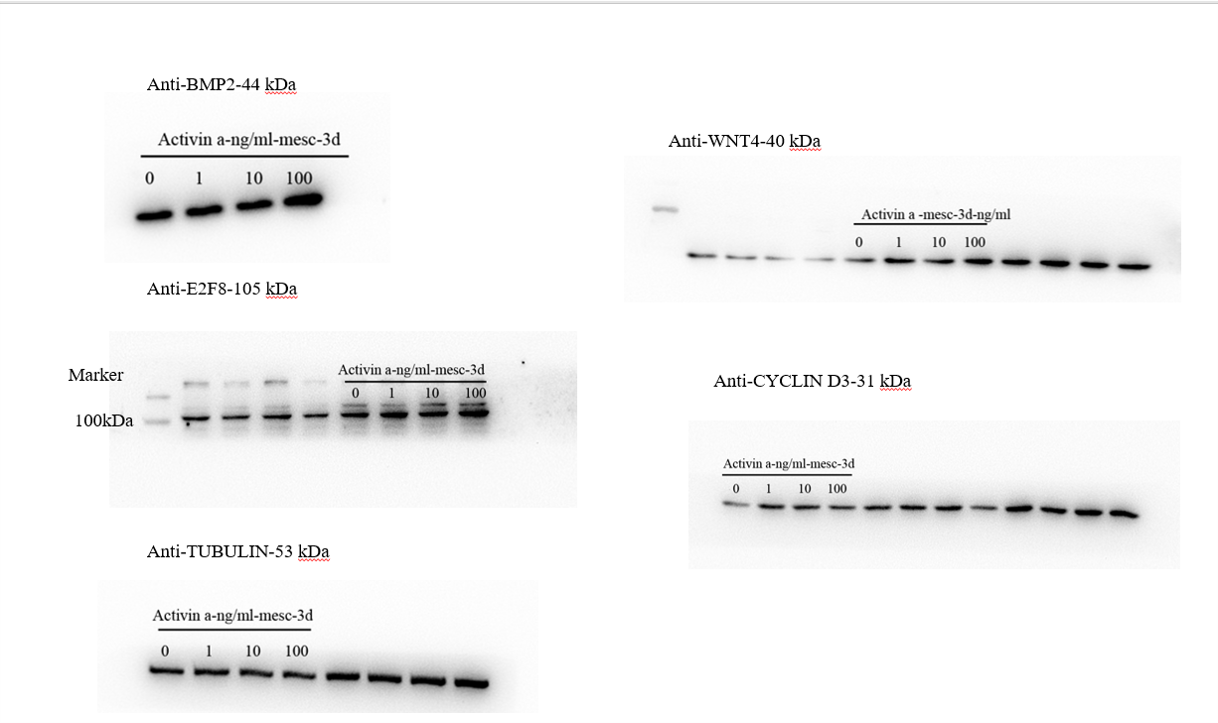


Fig. 2E-source data-5. Western blot analysis on the effects of ACTIVIN A on decidualization markers after stromal cells were treated with ACTIVIN A for 72 h.


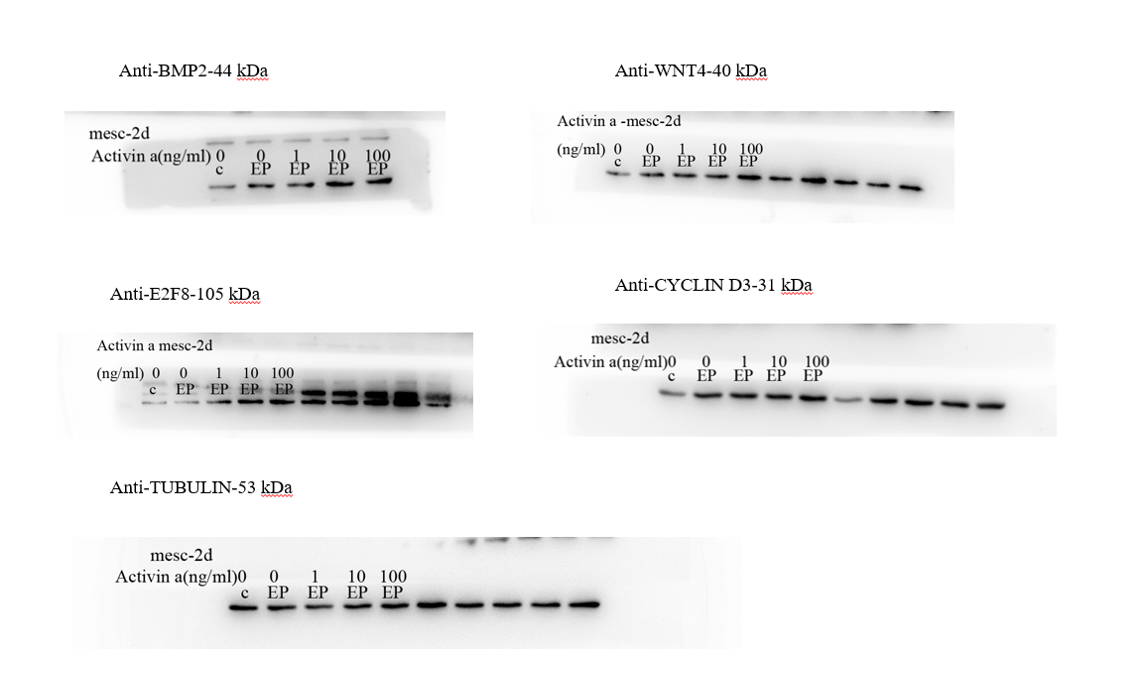


Fig. 2F-source data-6. Western blot analysis on the effects of ACTIVIN A on decidualization markers after stromal cells were treated with ACTIVIN A for 48 h under in vitro decidualization.


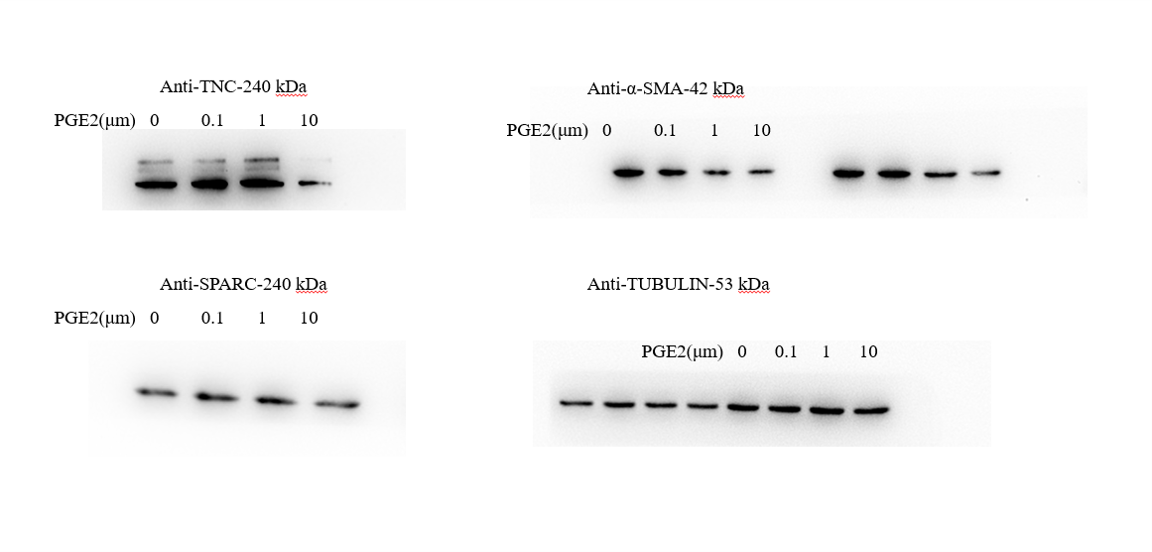


Fig. 3A-source data-1. The effects of PGE2 on markers of fibroblast activation.


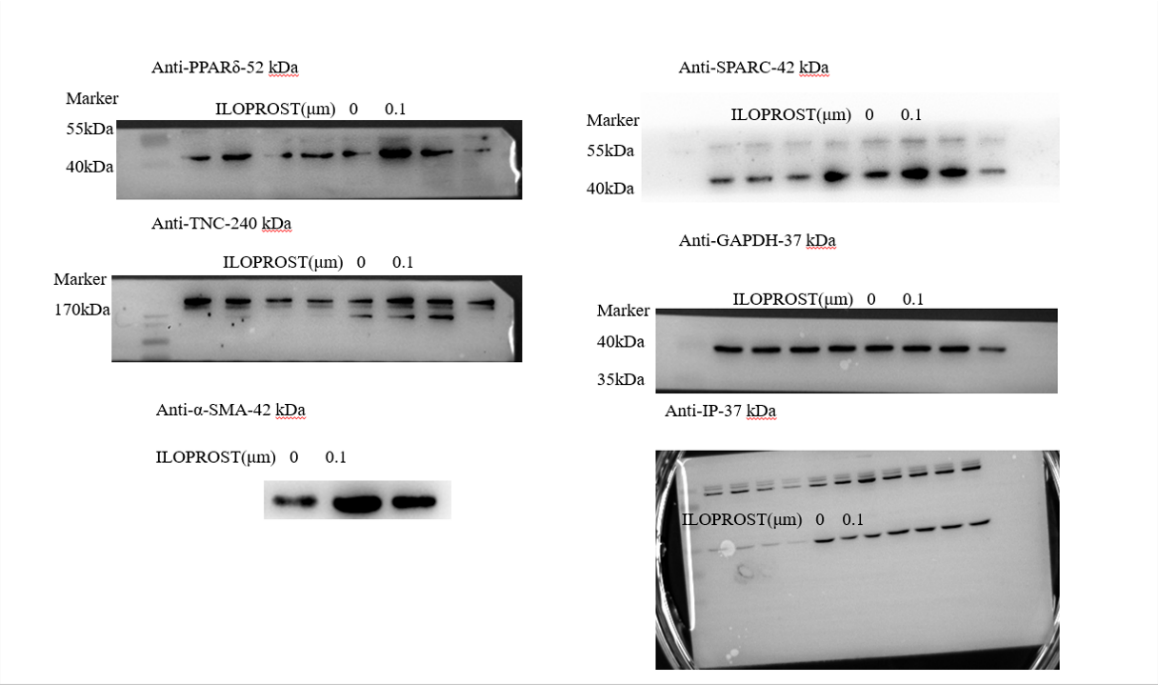


Fig. 3B-source data-2. The effects of ILOPROST, PGI2 analog, on markers of fibroblast activation after stromal cells was treated with PGI2 for 12 h.


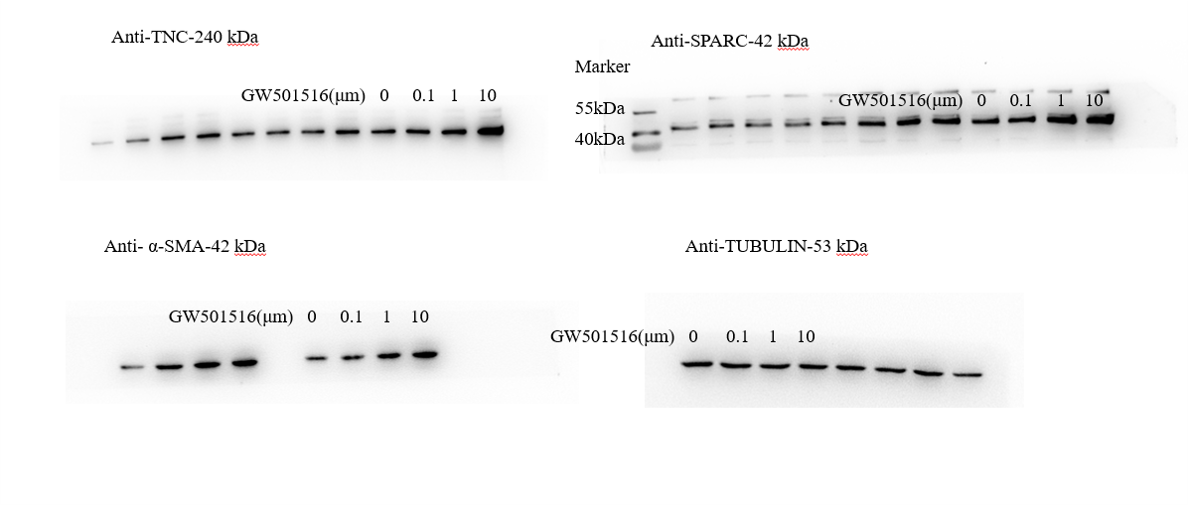


Fig. 3C-source data-3. The effects of GW501516, PPARδ agonist, on markers of fibroblast activation.


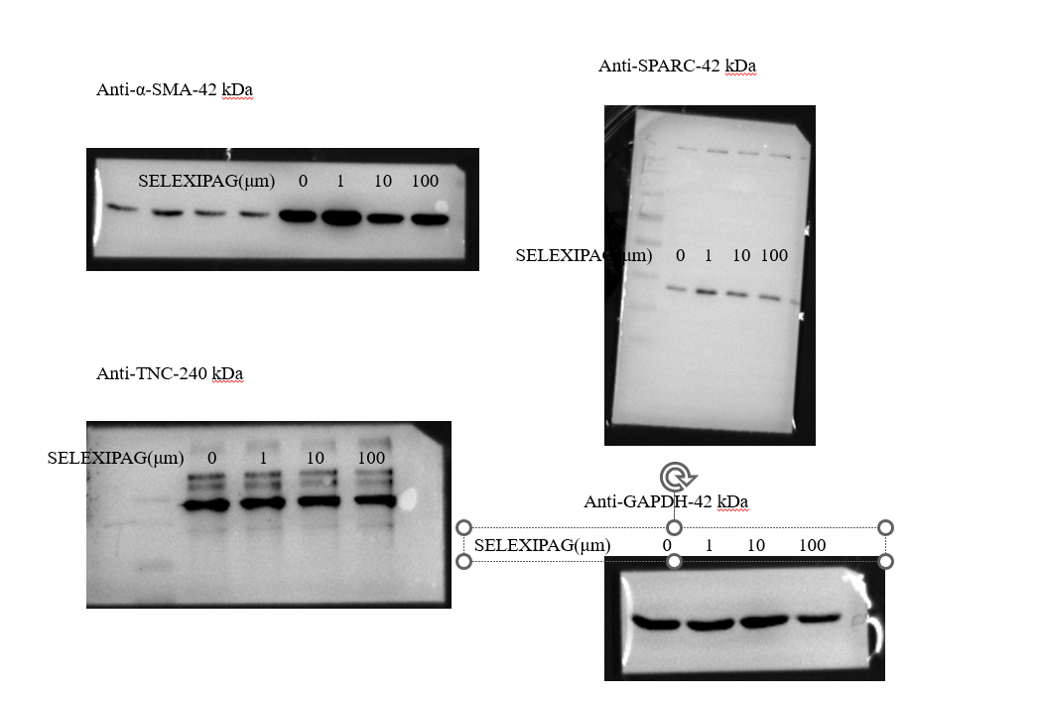


Fig. 3D-source data-4. The effects of SELEXIPA, IP analog, on markers of fibroblast activation.


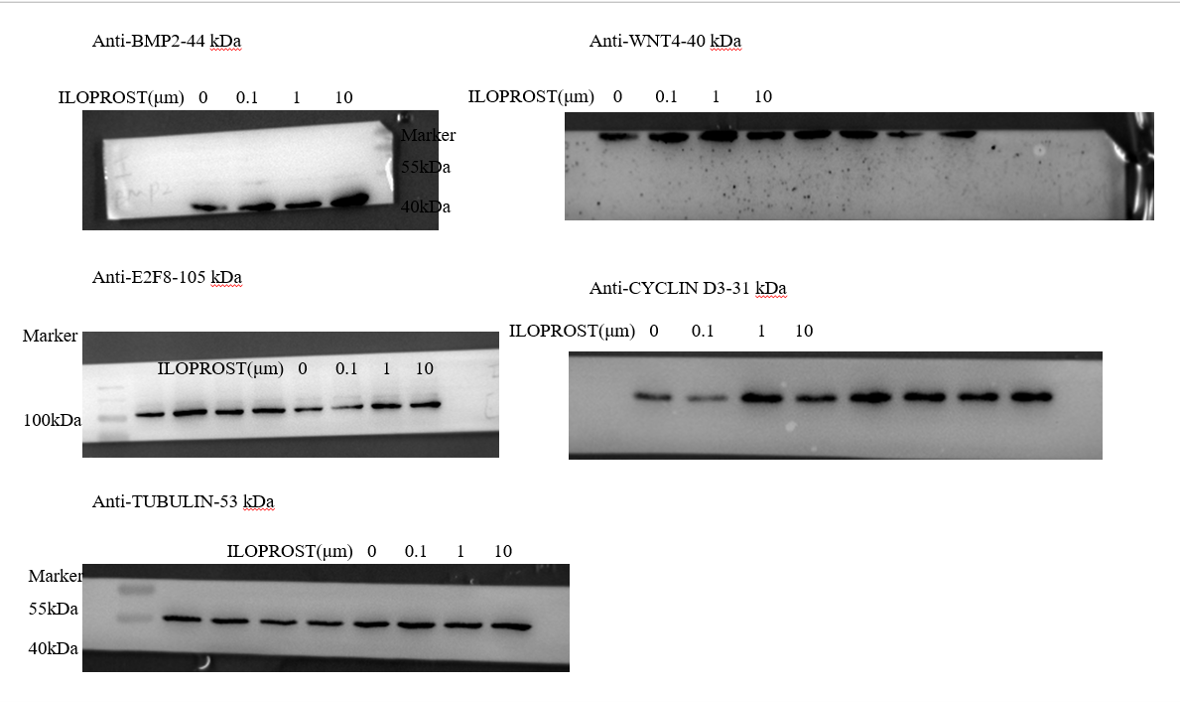


Fig. 3E-source data-5. The effects of ILOPROST on decidualization markers.


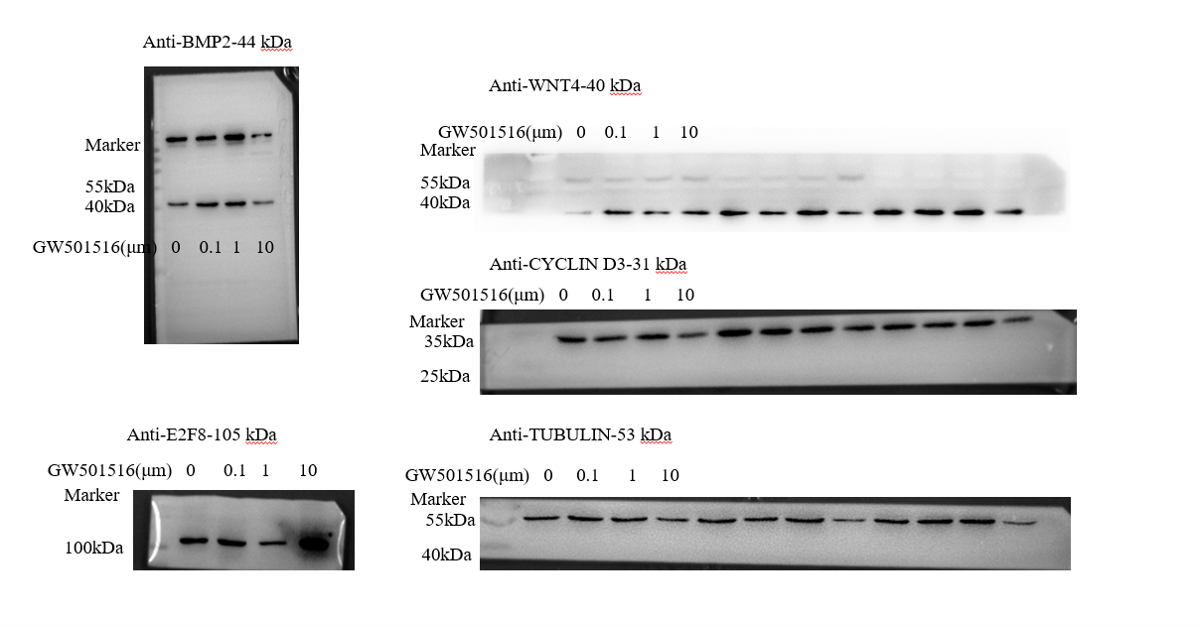


Fig. 3F-source data-6. The effects of GW501516 on decidualization markers.


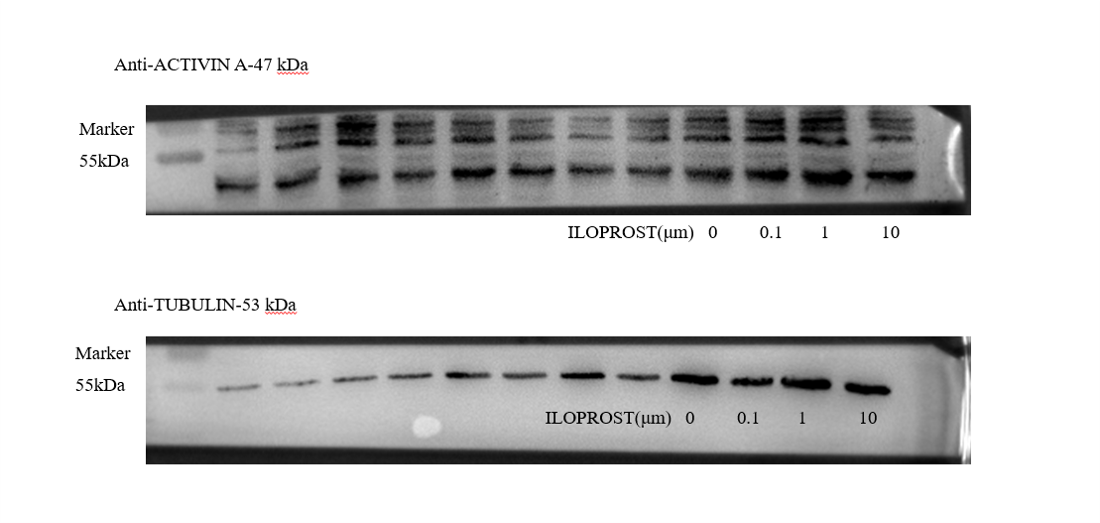


Fig. 3G-source data-7. The effects of ILOPROST on ACTIVIN A protein levels after stromal cells were treated with ILOPROST for 24 h.


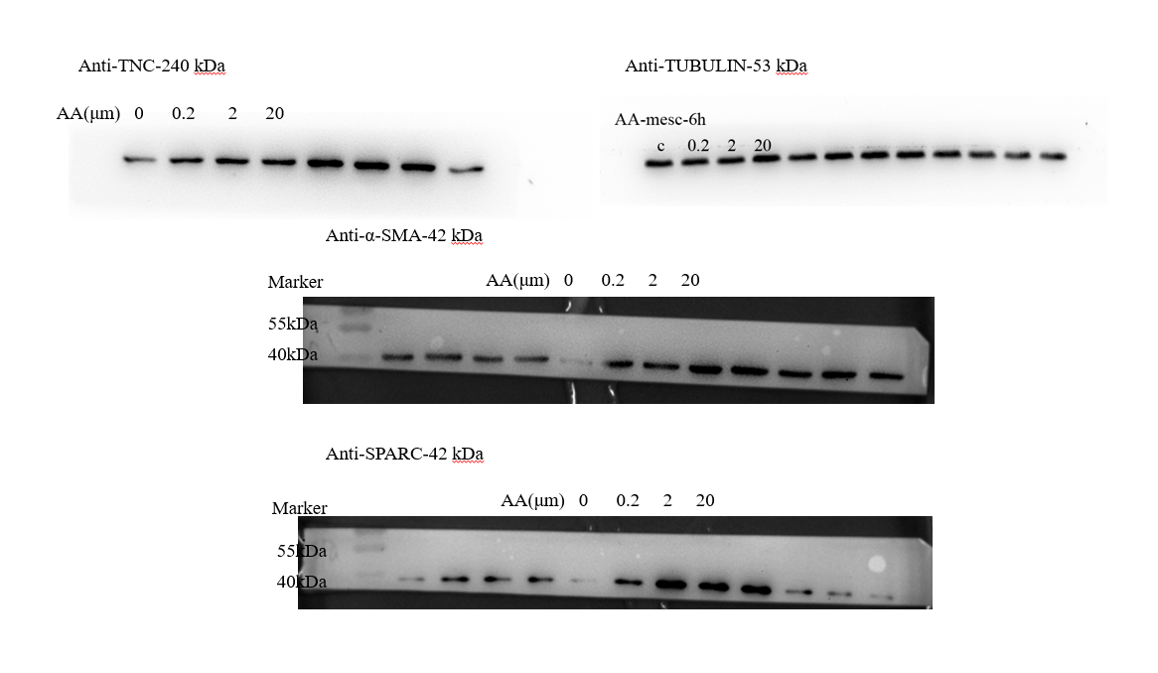


Fig. 4A-source data-1. Western blot analysis on effects of arachidonic acid on markers of fibroblast activation after stromal cells were treated with arachidonic acid for 6 h.


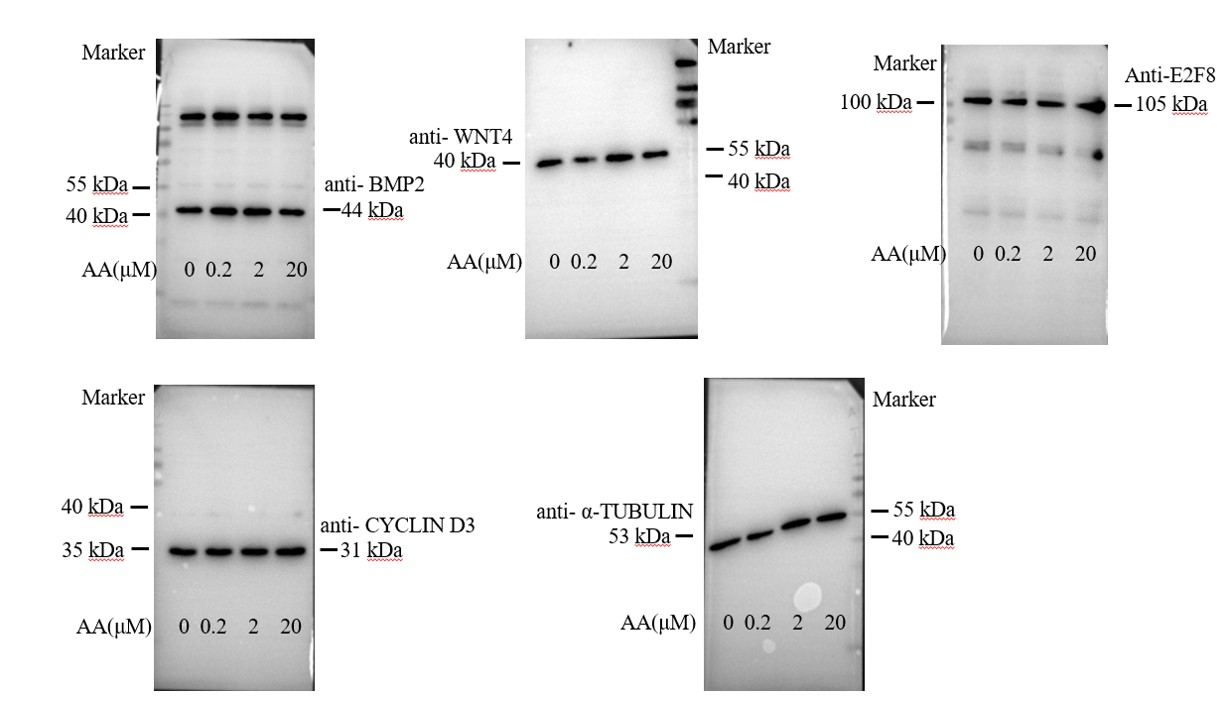


Fig. 4B-source data-2. Western blot analysis on effects of arachidonic acid on decidualization markers after stromal cells were treated with arachidonic acid for 48 h.


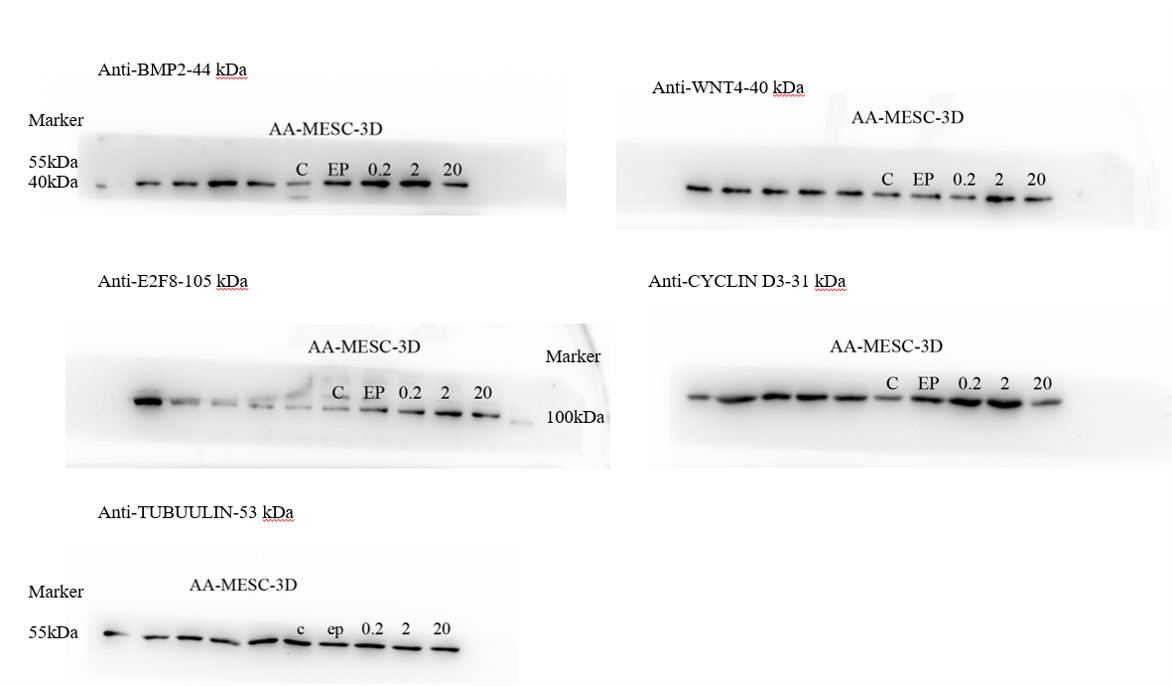


Fig. 4C-source data-3. Western blot analysis on effects of arachidonic acid on decidualization markers after stromal cells were treated with arachidonic acid for 48 h under in vitro decidualization. EP, 17β-estradiol + progesterone.


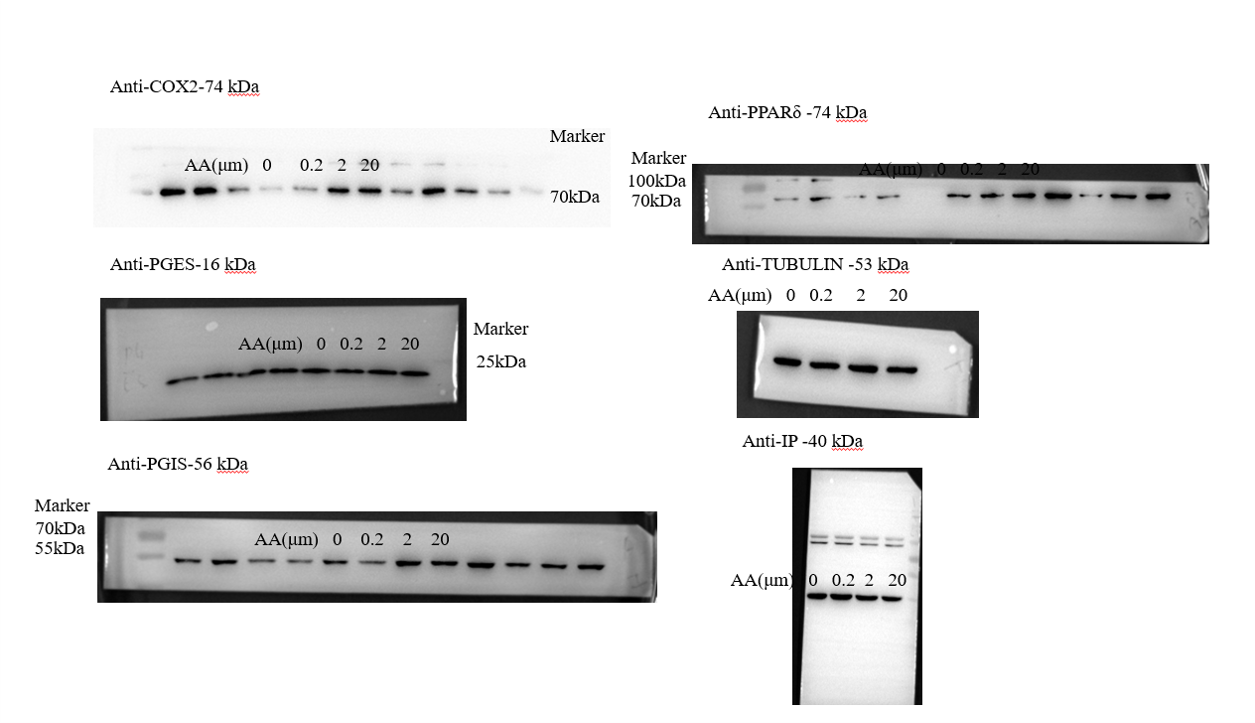


Fig. 4E-source data-4. Western blot analysis on effects of arachidonic acid on COX2, PGES, PGIS and PPARδ protein levels after stromal cells were treated with arachidonic acid for 6 h.


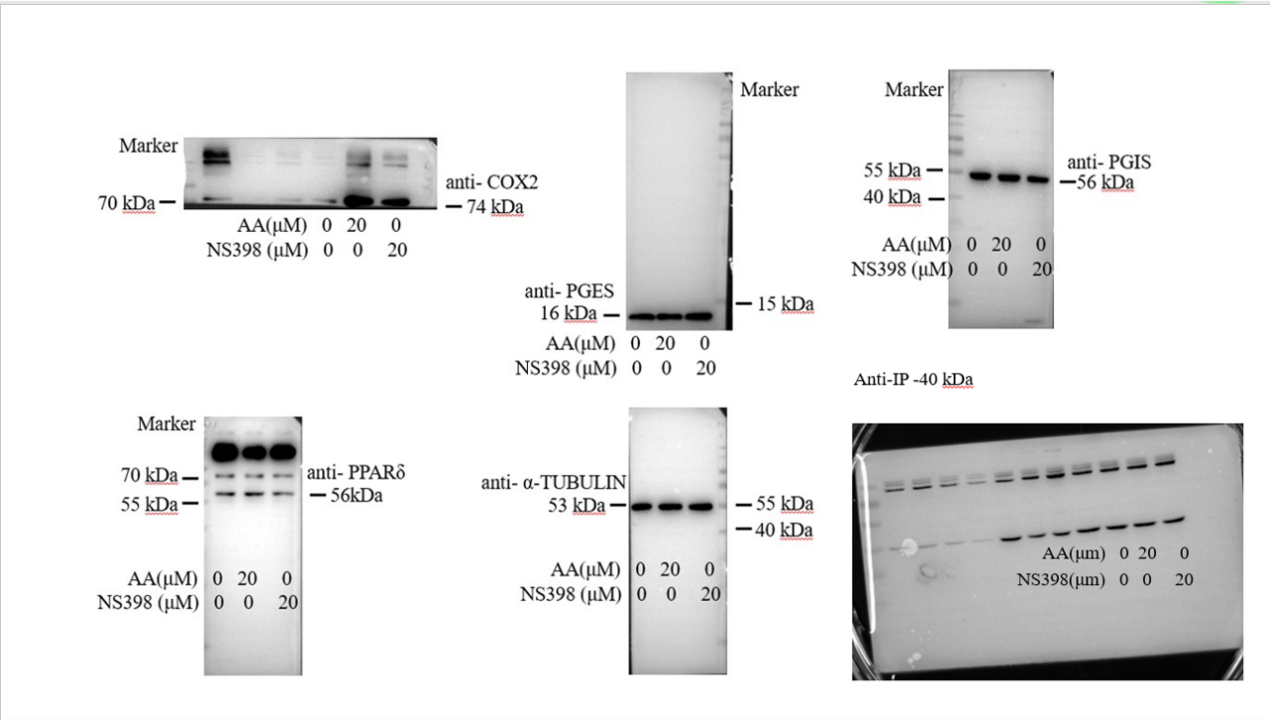


Fig. 4F-source data-5. Western blot analysis on effects of NS398 (COX-2 inhibitor) on arachidonic acid induction of COX2, PGES, PGIS and PPARδ protein levels after stromal cells were treated with arachidonic acid for 48 h in the absence or presence of NS398.


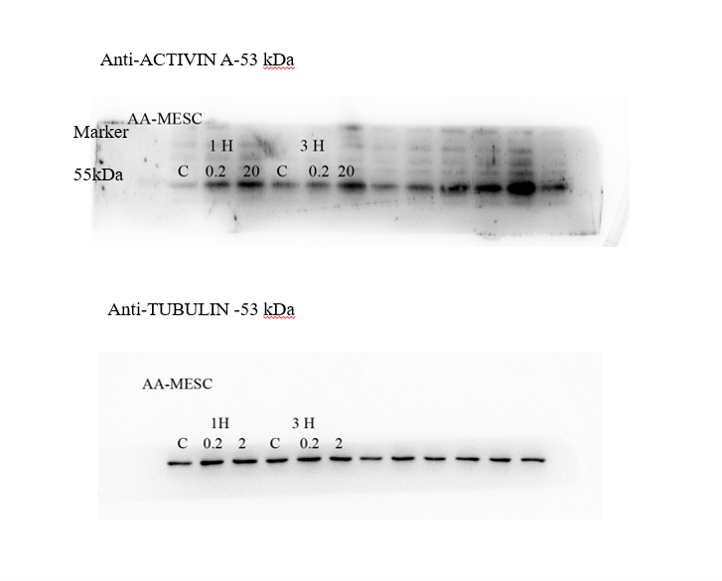


Fig. 4J-source data-6. Western blot analysis on effects of arachidonic acid on ACTIVIN A protein level after stromal cells were treated with AA for 24 h.


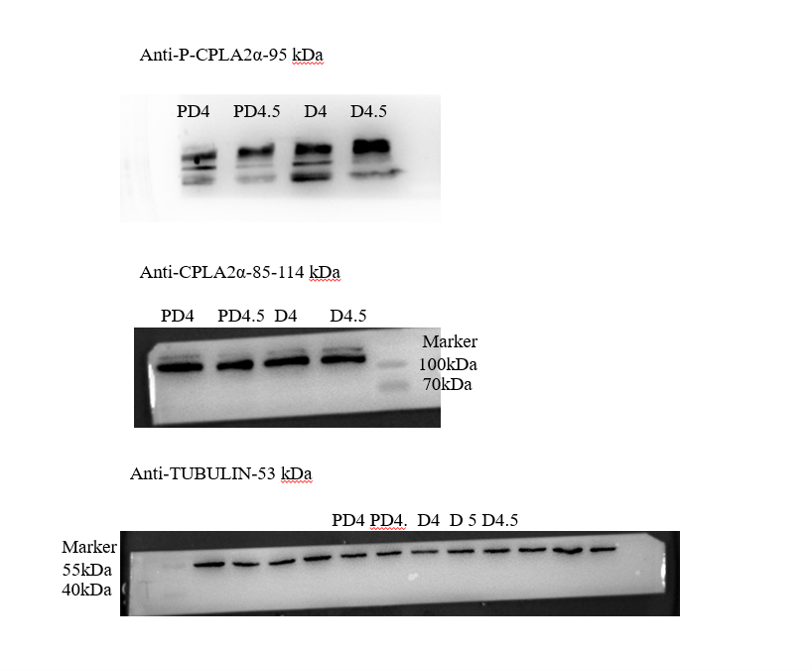


Fig. 5E-source data-1. Western blot analysis of cPLA2α and p-cPLA2α protein levels in mouse uteri on day 4 and day 4 midnight of pregnancy, and day 4 and day 4 midnight of pseudopregnancy, respectively.


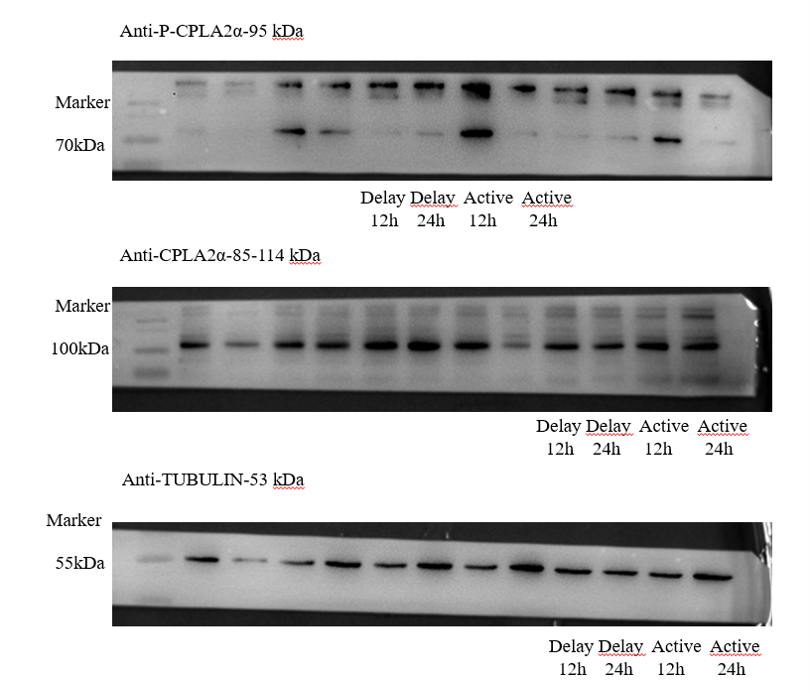


Fig. 5F-source data-2. Western blot analysis of cPLA2α and p-cPLA2α protein levels in mouse uteri 12 and 24 h after delayed implantation was activated by estrogen treatment.


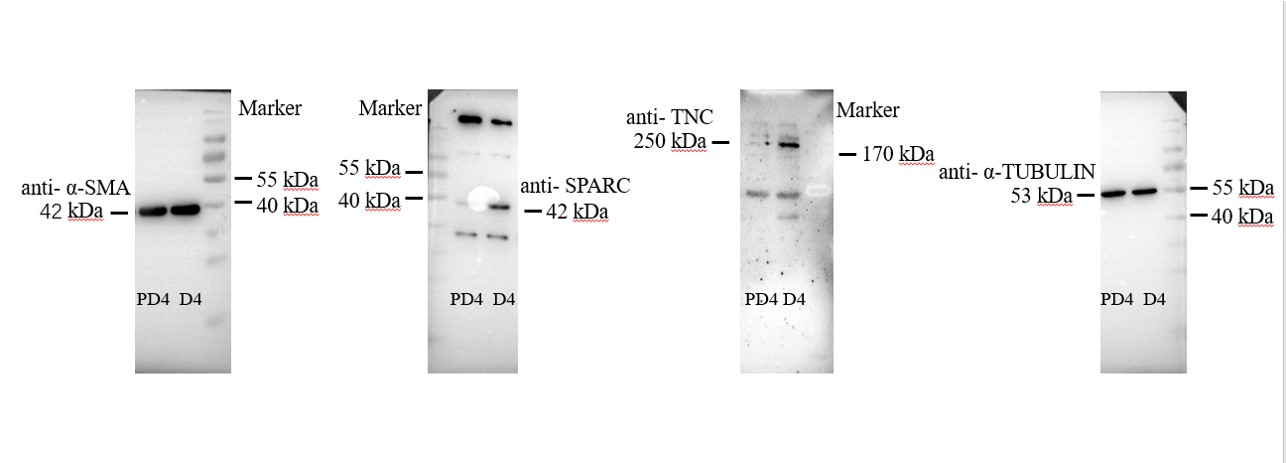


Fig. 5H-source data-3. Western blot analysis α-SMA, TNC, and SPARC protein levels in mouse uteri on day 4 of pregnancy and day4 of pseudopregnancy.


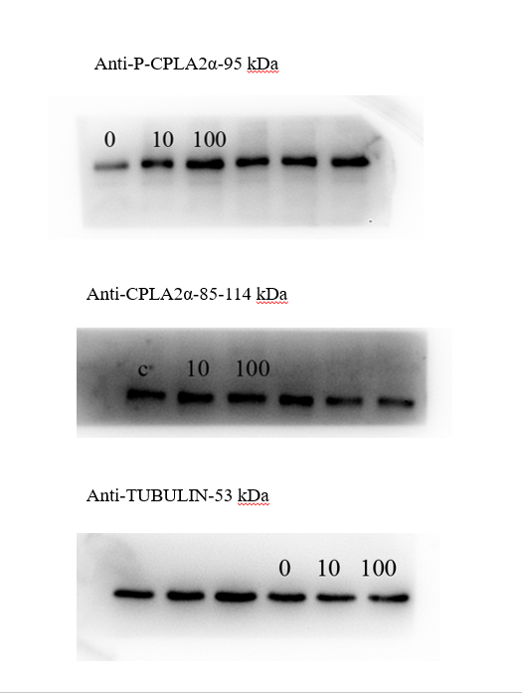


Fig. 6E-source data-1. Western blot analysis of cPLA2α and p-cPLA2α protein levels after cultured epithelial cells were treated with TNFα for 3 h.


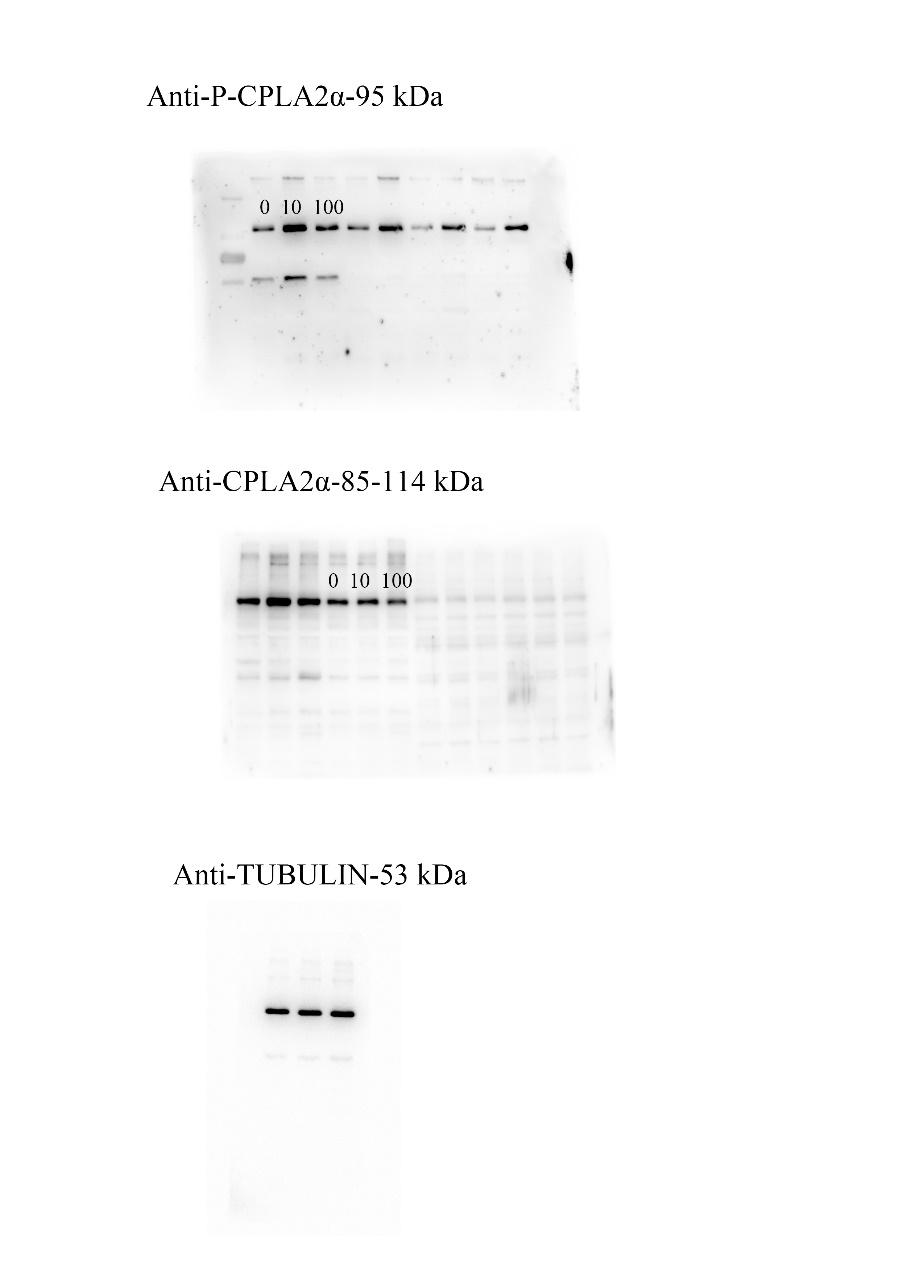


Fig. 6F-source data-2. Western blot analysis of cPLA2α and p-cPLA2α protein levels after cultured epithelial organoids were treated with TNF for 6h.


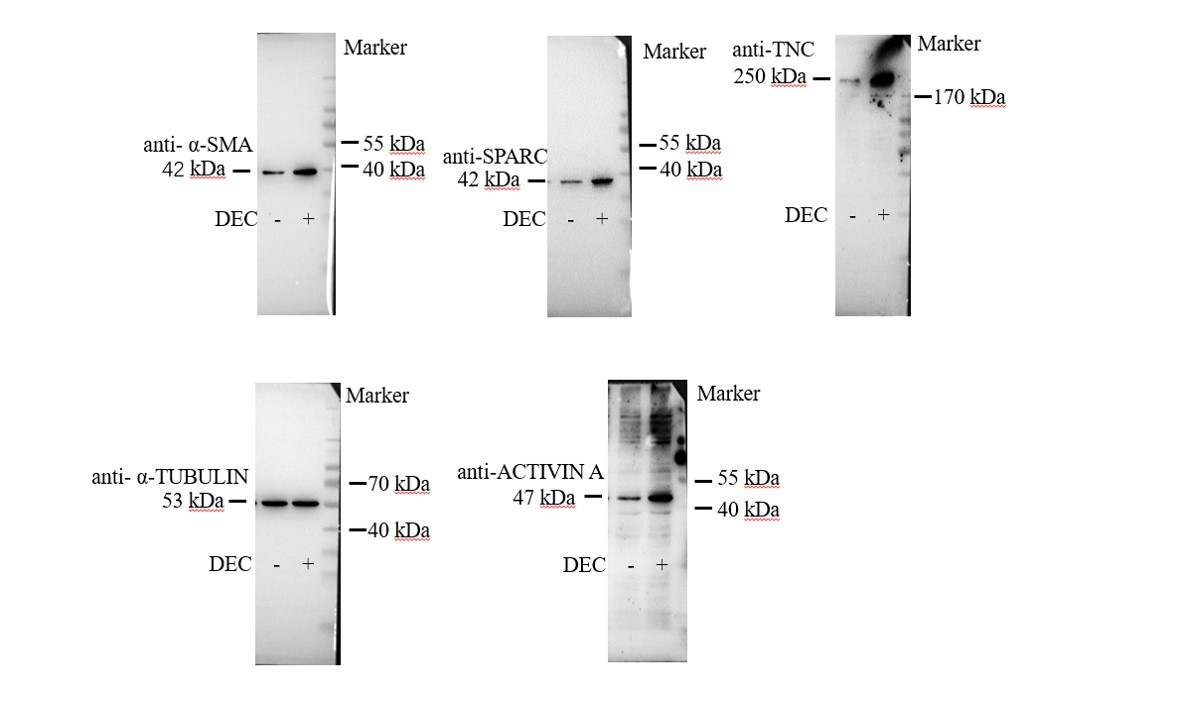


Fig. 7A-source data-1. Western blot analysis of α-SMA, TNC, SPARC and ACTIVIN A protein levels after human stromal cells were induced for decidualization for 24 h.


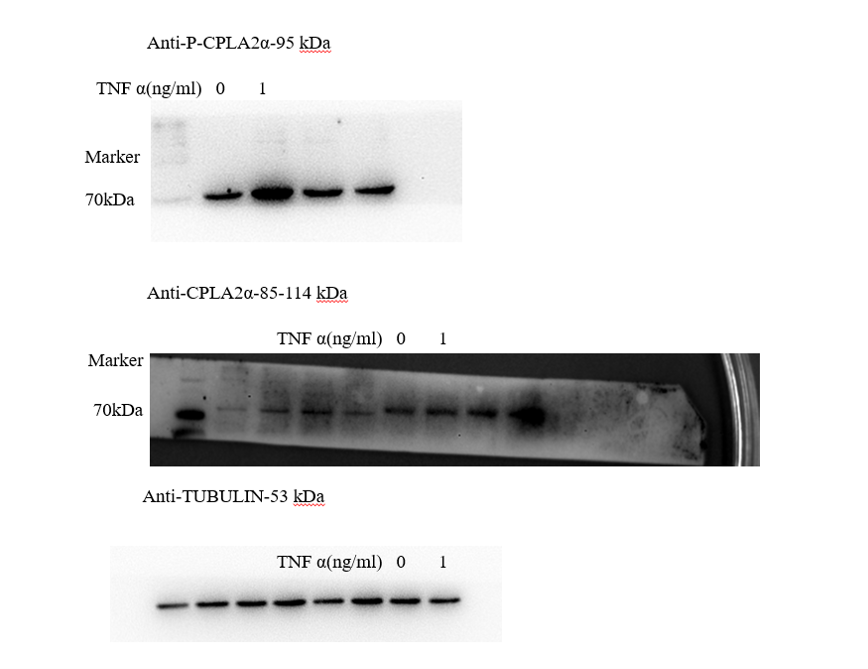


Fig. 7B-source data-2. Western blot analysis of cPLA2α and p-cPLA2α protein levels after human ISHIKAWA cells were treated with TNFα for 3 h.


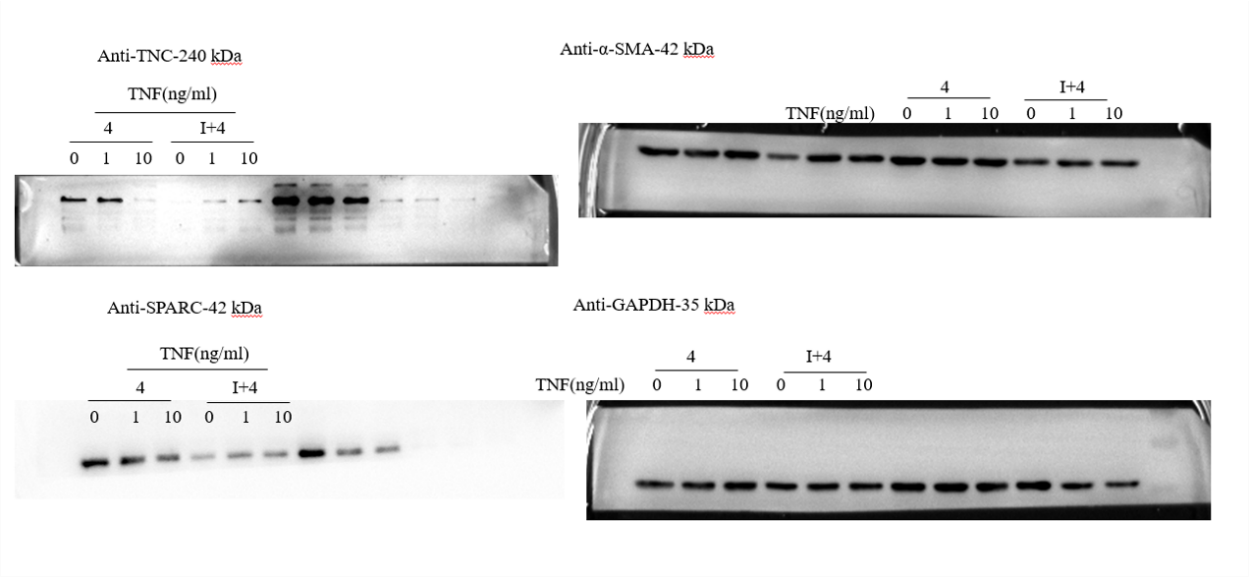


Fig. 7C-source data-3. Western blot analysis of TNC, α-SMA, SPARC and ACTIVIN A protein levels in stromal 4003 cells after the co-culture of ISHIKAWA cells and stromal cells were treated with TNFα for 3 h.


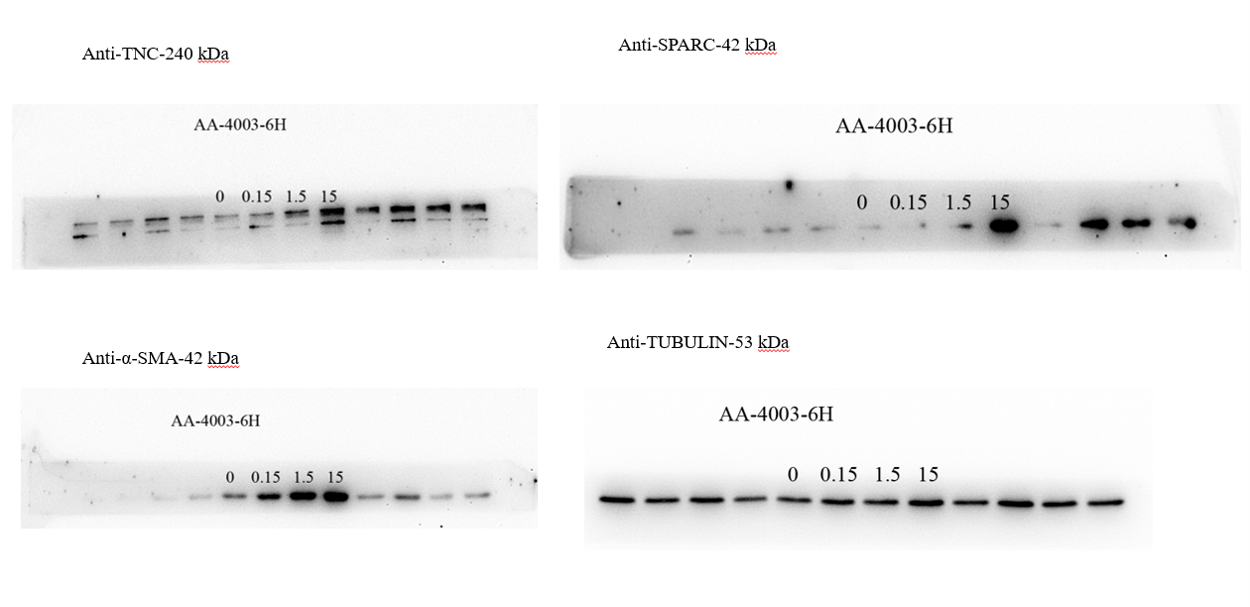


Fig. 7D-source data-4. Western blot analysis of TNC, α-SMA and SPARC protein levels after stromal 4003 cells were treated with AA for 6 h.


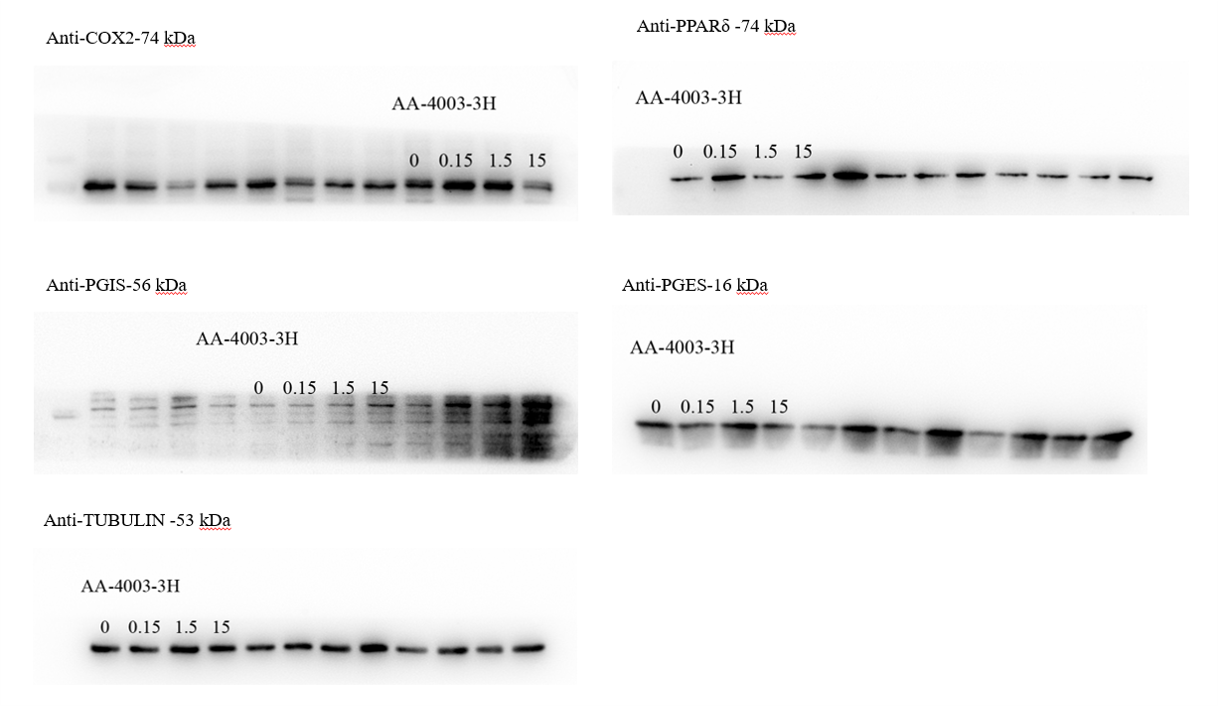


Fig. 7E-source data-5. Western blot analysis of COX-2, PGES, PGIS, and PPARδ protein levels after stromal 4003 cells were treated with AA for 3 h.


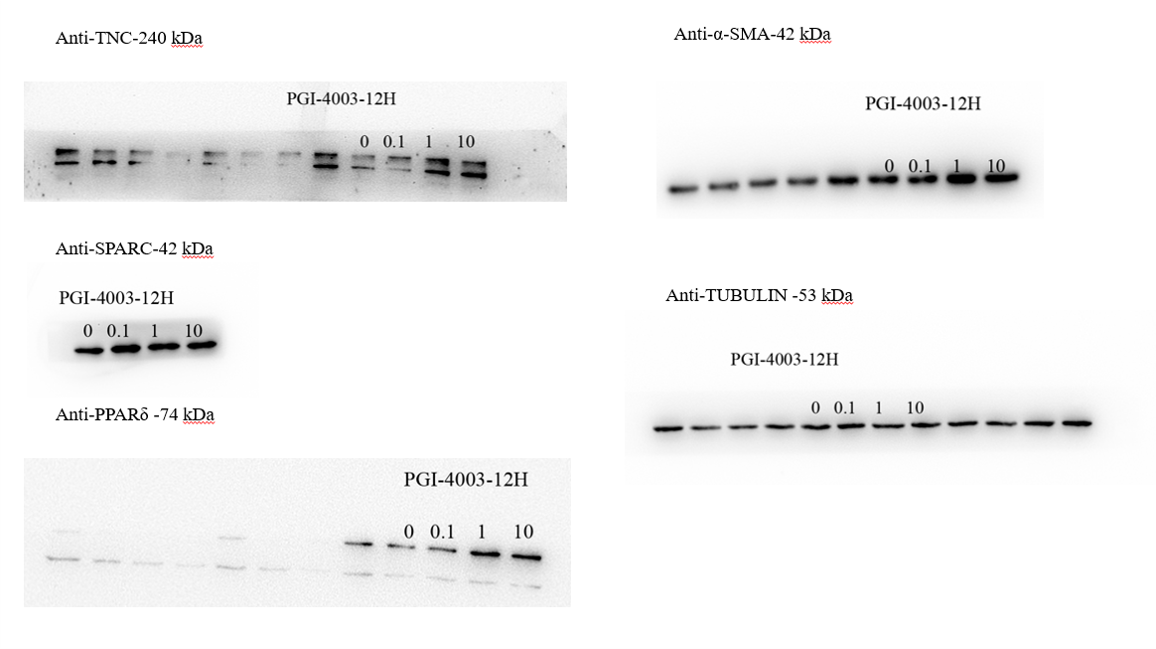


Fig. 7F-source data-6. Western blot analysis of PPARδ, TNC, α-SMA and SPARC protein levels after stromal 4003 cells were treated with ILOPROST for 12 h.


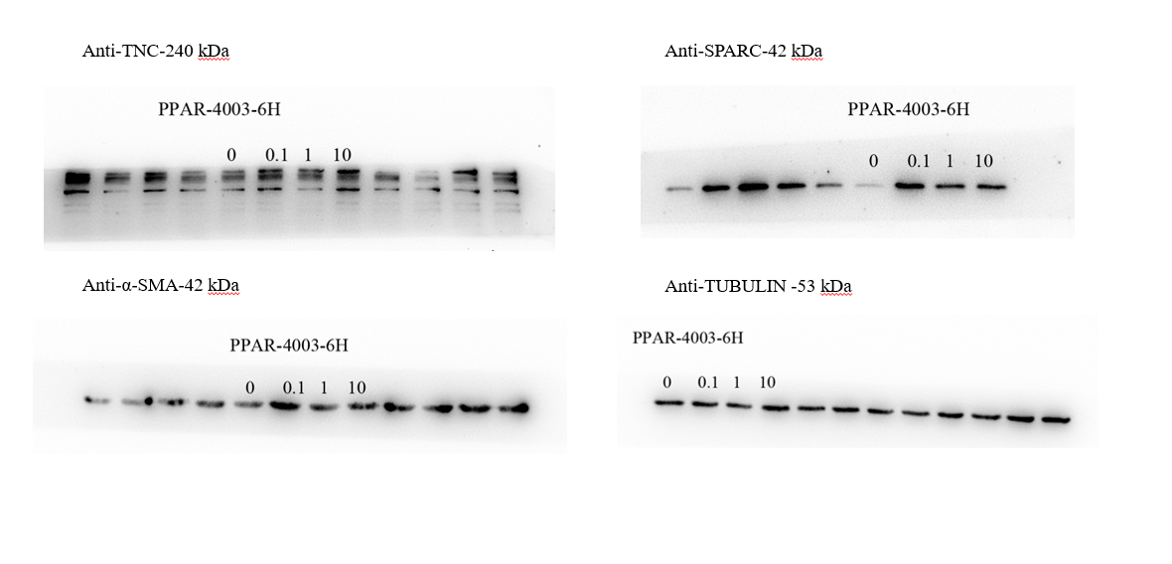


Fig. 7G-source data-7. Western blot analysis of TNC, α-SMA and SPARC protein levels after stromal cells 4003 cells were treated with GW501516 for 6 h.
